# Stewartiacids A–N, C-23 carboxylated triterpenoids from Chinese Stewartia and their inhibitory effects against ATP-citrate lyase and NF-κB†‡

**DOI:** 10.1039/c9ra09542j

**Published:** 2020-01-21

**Authors:** Jiang Wan, Yi Zang, Dao-An Xiao, Na Li, Junmin Li, Ze-Xin Jin, De-Lei Chen, Juan Xiong, Jia Li, Jin-Feng Hu

**Affiliations:** Institute of Natural Medicine and Health Products, School of Advanced Study, Zhejiang Provincial Key Laboratory of Plant Ecology and Conservation, Taizhou University Taizhou 318000 Zhejiang PR China; School of Pharmacy, Fudan University No. 826 Zhangheng Road Shanghai 201203 PR China jfhu@fudan.edu.cn +86-21-51980172 +86-21-51980172; State Key Laboratory of Drug Research, Shanghai Institute of Materia Medica, Chinese Academy of Sciences Shanghai 201203 PR China; College of Chemistry and Bioengineer, Yichun University Yichun 336000 PR China; School of Life Science, Hefei Normal University Hefei 230601 PR China

## Abstract

Fourteen previously undescribed naturally occurring C-23 carboxylated triterpenoids, stewartiacids A–N (1–14), were isolated and characterized from the twigs and leaves of the ornamental and medicinal plant *Stewartia sinensis* (Chinese Stewartia), a ‘vulnerable’ species endemic to China. The new structures were elucidated on the basis of spectroscopic data, single crystal X-ray diffraction, and electronic circular dichroism (ECD) analyses. Stewartiacids A (1) and B (2) are isoursenol derivatives. Stewartiacid C (3) is a 12-oxo-γ-amyrin analogue. Both isoursenol and γ-amyrin derivatives are quite rare in nature. Stewartiacids D (4) and E (5) are 13,27-cycloursane-type compounds. Stewartiacids K (11) and L (12) are ursane-type triterpene and phenylpropanol adducts built through a 1,4-dioxane ring, which are also seldom reported in the literature. The rest are common C-23 carboxylated ursane-type (6–10) and oleanane-type (13, 14) pentacyclic triterpenoids. Stewartiacids G (7), K (11), and L (12) showed moderate inhibitory effects against ATP-citrate lyase (ACL), with IC_50_ values of 12.5, 2.8, and 10.6 μM, respectively. Stewartiacid K (11) also exhibited moderate inhibition (IC_50_: 16.8 μM) of NF-κB.

## Abstract 2

Fourteen new C-23 carboxylated triterpenoids (stewartiacids A–N, 1–14) were obtained from the ‘vulnerable’ plant *Stewartia sinensis*. 11 and 12 showed inhibitory effects against ACL.

## Graphical abstract

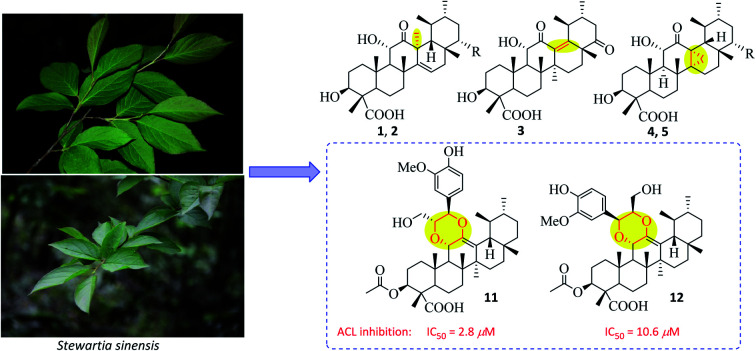

## Introduction

The small plant genus *Stewartia*, one member of the family Theaceae, comprises about twenty species worldwide and three quarters are distributed in China.^1^ Several species of *Stewartia* are not only grown as ornamental plants but also utilized as folk medicines.^2^ There are three well-known *Stewartia* species, which are mainly distributed in East Asia, *i.e.*, Chinese Stewartia (*S. sinensis* Rehd. et Wils.), Korean Stewartia (*S. koreana* Nakai), and Japanese Stewartia (*S. pseudocamellia* Maxim.^3^). Phenolic compounds (*e.g.*, flavonoids) and sterols have been reported from the leaves/twigs^4–6^ and stems^7^ of Korean Stewartia with anti-inflammatory,^4,7^ anti-photoaging,^5^ and antioxidative^6*a*^ effects. Phenolic derivatives have also been obtained from the leaves/twigs,^8^ flowers,^9^ and stems^10^ of Japanese Stewartia with anti-melanogenic,^8*a*,*c*^ antioxidative,^8*d*^ and anti-allergic effects.^9^ It is uncertain that both *S. pseudocamellia* and *S. koreana* have been regarded as the same plant species by some Korean researchers.^8*a*,9^ In addition, a few triterpenoids have been encountered from some *Stewartia* species.^8*a*,11^

Chinese Stewartia is a flowering camellia plant endemic to central and eastern China. This relict species is highly regarded horticulturally for the combination of its beautiful, buff/tan colour bark—carried high up into the crown—and absolutely smooth with little or no flaking, cup-shaped/fragrant white single flowers in midsummer, elliptical papery leaves, and intensely red fall foliage.^1,12^ The population of *S. sinensis* is dwindling due to the destruction of vegetation and the poor ability of natural regeneration. This ornamental plant has been recorded as a ‘vulnerable’ species in the China Plant Red Data Book (CPRDB) published in 1992.^13^ It is also on the International Union for Conservation of Nature (IUCN) Red List.^14^ Its stems and roots have been used as a folk medicine to treat rheumatic and traumatic injury.^13,15^ However, *S. sinensis* has never been phytochemically and pharmacologically investigated. Recently, several statistical surveys unveiled that plant-originated natural products (NPs) and their intricate molecular frameworks still offer medicinal chemists a range of uncharted chemotypes for drug discovery,^16^ among which the rare and endangered plants (REPs) could serve as better sources than other botanic sources.^17^ A pioneering phylogenetic study of the terrestrial plants showed that NPs-derived drugs are mainly produced by specific drug-productive plant families, and most REPs species are distinctly in drug-producing families.^17*a*^ Therefore, there is a tremendous need to prioritize protection and utilization of these REPs species at extinction risk. Since 2013, a special program has been launched to systematically identify bioactive/novel NPs from REPs. For examples, a number of structurally diverse sesquiterpenoids, triterpenoids, and diterpenoids with protein tyrosine phosphatase 1B (PTP1B) inhibitory and cytotoxic effects were isolated and characterized from the leaves and twigs of the endangered ornamental plants *Michelia shiluensis*,^18^*Camellia crapnelliana* (Crapnell's camellia, also in the family Theaceae),^19^ and the shed trunk barks of the endangered plant *Pinus dabeshanensis*,^20^ respectively. In the course of our continuous interest in identifying bioactive compounds from REPs endemic to China,^21^ the chemical constituents of the EtOAc-soluble fraction of the 90% MeOH extract of the twigs and leaves of *S. sinensis* have been investigated. It is worth mentioning that the Chinese Stewartia is generally a 6–16 m tall tree,^1,12^ making the plant samples (the renewable leaves and twigs) easier to harvest. As a result, a total of 18 triterpenoids were isolated and characterized, including 14 previously undescribed C-23 carboxylated ones (stewartiacids A–N, 1–14, resp.). Reported herein are their isolation, structure determination, and inhibitory effects against ATP-citrate lyase (ACL) and NF-κB.

## Results and discussion

The 90% MeOH extract of the twigs and leaves of *S*. *sinensis* (3.5 kg, air-dried) was suspended in H_2_O and then partitioned successively with petroleum ether, EtOAc, and *n*-BuOH. The entire EtOAc-soluble fraction was subjected repeatedly to column chromatography (CC) over silica gel, MCI gel, Sephadex LH-20, and semi-preparative HPLC to afford eighteen triterpenoids (1–18). By comparing the observed and reported spectroscopic data and physicochemical properties, the known compounds 15–18 were identified as urs-12-en-3β-ol (α-amyrin, 15),^22^ ursolic acid (16),^23^ olean-12-en-3β-ol (β-amyrin, 17),^22^ and oleanic acid (18),^23^ respectively.

Stewartiacid A (1) was obtained as colorless crystals from MeOH. Its molecular formula, C_30_H_46_O_6_, was determined by the HRESIMS at *m*/*z* 525.3182 [M + Na]^+^ (calcd 525.3187) and ^13^C NMR data (Table 1) with eight indices of hydrogen deficiency (IHD). In the upfield region of the ^1^H NMR spectrum (Table 2), resonances for five tertiary methyl groups at *δ*_H_ 0.83 (3H, s, Me-28), 0.86 (3H, s, Me-26), 1.03 (3H, s, Me-25), 1.09 (3H, s, Me-24), and 1.46 (3H, s, Me-27), and two secondary methyl groups at *δ*_H_ 0.92 (3H, d, *J* = 6.4 Hz, Me-29) and 0.98 (3H, d, *J* = 6.1 Hz, Me-30) were observed. In addition, signals for three oxymethine resonances at *δ*_H_ 3.51 (1H, dd, *J* = 10.6, 3.4 Hz, H-22), 3.96 (1H, dd, *J* = 10.1, 5.9 Hz, H-3), and 4.05 (1H, d, *J* = 9.1 Hz, H-11), and an olefinic proton at *δ*_H_ 5.68 (1H, dd, *J* = 8.3, 2.4 Hz, H-15) were also readily distinguished. Its ^13^C NMR spectrum resolved 30 carbon resonances (Table 1), which were classified as seven methyl, seven methylene, nine methine, five quaternary carbons, and two carbonyls with the assistance of DEPT and HSQC NMR experiments. As evidenced from the aforementioned data, the presence of one ketone carbonyl [*δ*_C_ 214.2 (C-12)], one carboxyl group [*δ*_C_ 181.3 (C-23)], and a double bond [*δ*_C_ 119.9 (C-15) and 157.1 (C-14)] corresponded to three IHDs, thus requiring 1 to feature a pentacyclic skeleton. The NMR data of 1 were found to closely related to those of 3β,6α-dihydroxy-urs-14-en-12-one,^24^ an unusual isoursenol derivative previously isolated from the roots of *Rubia schumanniana*. Unlike common ursane-type triterpenoids (*e.g.*, 15 (ref. 22) and 16 (ref. 23)), the Me-27 group migrates from C-14 to C-13 in the isoursenol derivatives, which is quite rare in nature.^24,25^ The major differences between 1 and the aforementioned known structure^24^ were that, the 5-OH group was absent in 1, while instead two hydroxy groups were attached to C-11 and C-22 as confirmed by the ^1^H–^1^H COSY motifs of H-9/H-11 and H-18/H-19 (H_3_-29)/H-20 (H_3_-30)/H-21/H-22, and the HMBC correlations of H-9/C-11 and H_3_-28/C-22 (Fig. 2). In addition, a carboxyl group rather than a methyl was assigned at C-23 from the distinct HMBC correlations from H-3 and H_3_-24 to this carboxyl signal at *δ*_C_ 181.3 (Fig. 2).

**Table 1:** ^13^C NMR data^a^ (*δ* in ppm, 150 MHz) of 1–10, 13, and 14

No.	1^b^	2^c^	3^b^	4^c^	5^c^	6^c^	7^c^	8^b^	9^b^	10^b^	13^b^	14^c^
1	40.6	40.2	39.9	39.5	39.5	39.6	39.7	40.3	40.2	40.2	40.1	41.4
2	27.4	27.8	27.8	27.7	27.7	28.1	28.1	27.4	28.1	27.4	27.9	28.3
3	76.2	75.3	76.0	75.4	75.4	75.1	75.2	76.1	76.1	76.1	76.1	75.3
4	54.8	54.5	55.4	54.3	54.3	54.8	54.9	55.0	55.0	54.9	55.0	55.2
5	52.5	51.7	52.7	51.9	51.9	51.8	51.9	51.8	51.9	52.1	51.9	51.9
6	22.4	22.0	22.0	21.7	21.7	21.7	21.8	21.4	21.4	21.5	21.4	21.4
7	41.6	41.0	35.5	37.3	37.3	33.9	34.1	33.6	33.7	34.9	33.6	34.4
8	40.3	39.3	42.6	39.3	38.4	43.4	40.7	46.8	46.5	44.2	47.3	43.9
9	58.7	58.1	57.8	60.5	60.4	48.4	48.5	61.3	61.2	60.7	62.0	58.2
10	38.9	38.3	41.7	37.9	37.9	38.0	38.1	37.6	37.8	37.7	37.9	39.6
11	75.3	74.9	78.4	73.3	73.3	77.1	77.1	196.2	196.1	196.2	196.0	73.2
12	214.1	213.0	209.8	207.4	207.4	145.1	145.1	146.3	146.6	144.2	144.8	212.9
13	53.6	52.3	141.9	38.5	39.2	116.5	116.8	133.9	130.7	128.8	134.6	81.6
14	157.1	156.7	47.2	45.1	44.5	41.4	43.4	43.3	42.7	45.6	42.6	45.5
15	119.9	118.7	26.0	21.1	21.5	27.2	27.7	28.1	27.3	28.0	26.3	21.8
16	28.8	37.5	30.2	19.0	27.7	20.9	27.9	21.3	27.4	31.2	27.5	25.6
17	41.6	35.0	49.3	37.0	30.9	39.2	33.5	39.7	49.2	49.0	40.3	49.5
18	51.7	48.4	147.9	40.7	40.3	47.3	47.4	49.9	48.9	130.7	47.5	47.2
19	38.4	36.8	38.5	41.1	41.1	40.9	41.3	41.7	41.9	138.6	42.1	41.4
20	38.2	36.6	33.9	36.2	38.5	37.8	40.0	38.8	40.2	39.0	35.4	33.6
21	38.9	28.7	46.2	40.1	31.1	40.3	31.5	40.1	47.0	43.9	51.9	48.9
22	78.9	37.7	216.5	78.6	42.1	78.5	42.2	79.5	218.0	217.0	218.8	217.4
23	181.3	180.4	181.5	180.3	180.3	180.5	180.6	181.1	181.2	180.1	181.0	180.1
24	11.4	12.0	11.5	11.8	11.8	12.2	12.3	11.5	11.5	11.6	11.4	12.1
25	18.0	17.7	17.2	18.0	18.0	17.0	17.1	17.5	17.5	17.9	17.5	16.9
26	25.8	25.3	19.5	19.3	19.3	18.1	18.2	18.9	18.9	18.9	19.1	21.1
27	23.2	21.4	21.2	18.4	18.3	23.9	23.9	21.6	21.0	18.3	23.1	17.0
28	31.8	33.2	25.8	25.4	28.4	25.4	28.8	25.8	21.7	23.7	22.2	26.5
29	24.2	23.3	21.9	16.8	17.1	17.0	17.2	16.9	16.5	19.6	32.1	31.3
30	22.1	22.1	20.9	20.5	20.7	21.2	21.4	21.2	21.2	20.1	25.4	28.5
*C̲*H_3_O–						52.4	52.4					

a Assignments were made by a combination of 1D and 2D NMR experiments.

b Measured in CD_3_OD.

c Measured in C_5_D_5_N.

**Table 2:** ^1^H NMR data^a^ (*δ* in ppm, *J* in Hz) of 1–3

No.	1^b^	1^c^	2^d^	3^b^
1α	1.36, m	1.96, m	1.96, m	1.21, m
1β	1.93, ddd (14.0, 3.1, 2.9)	2.38, br d (13.3)	2.38, ddd (13.7, 3.4, 3.0)	2.61, br d (13.5)
2a	1.63, m	2.06, m	2.05, m	1.67, m
2b	1.61, m	2.01, m	1.97, m	1.65, m
3	3.99, dd (10.1, 5.9)	4.78, dd (7.8, 6.6)	4.77, dd (10.0, 6.0)	3.95, dd (11.5, 5.2)
5	1.62, br d (11.0)	2.29, br d (9.4)	2.28, br d (11.9)	1.57, br d (12.0)
6α	1.23, br d (10.9)	1.73, m	1.85, m	1.66, m
6β	1.63, m	1.86, m	1.73, m	1.18, m
7α	2.01, dd (11.6, 10.2)	2.02, m	2.03, m	1.52, m
7β	1.49, br d (10.2)	1.71, m	1.65, m	1.61, m
9	1.99, d (9.1)	2.50, d (8.6)	2.41, d (8.8)	1.67, d (11.5)
11	4.05, d (9.1)	4.64, d (8.6)	4.62, d (8.8)	4.38, d (11.5)
15	5.68, dd (8.3, 2.4)	5.76, dd (8.4, 2.4)	5.61, dd (7.9, 2.6)	1.49, m; 1.72, m
16α	2.06, dd (15.5, 2.4)	2.57, dd (15.4, 2.4)	2.18, dd (15.3, 2.6)	1.23, m
16β	1.85, dd (15.5, 8.3)	2.31, dd (15.4, 8.4)	1.55, dd (15.3, 7.9)	1.87, m
18	2.14, d (6.9)	2.62, d (7.7)	2.29, d (8.4)	
19	1.43, m	1.83, m	1.46, m	3.28, m
20	1.34, m	1.47, m	1.31, m	1.82, m
21α	1.28, ddd (11.4, 11.2, 10.6)	1.62, q-like (11.0)	1.05, m	2.13, dd (15.7, 11.9)
21β	1.61, ddd (11.4, 3.4, 2.1)	1.99, m	1.48, m	2.45, dd (15.7, 3.4)
22	3.51, dd (10.6, 3.4)	3.90, dd (8.9, 2.4)	1.23, ddd (13.8, 4.6, 3.7)	
			1.64, m	
24	1.09, s	1.69, s	1.71, s	1.13, s
25	1.03, s	1.16, s	1.16, s	1.18, s
26	0.86, s	1.04, s	1.01, s	1.24, s
27	1.46, s	1.89, s	1.77, s	0.92, s
28	0.83, s	1.17, s	0.88, s	1.33, s
29	0.92, d (6.4)	1.15, d (7.5)	1.08, d (6.5)	1.12, d (7.1)
30	0.98, d (6.1)	0.97, d (6.5)	0.89, d (6.9)	1.03, d (6.5)

a Assignments were made by a combination of 1D and 2D NMR experiments.

b Measured in CD_3_OD, 400 MHz.

c Measured in C_5_D_5_N, 600 MHz.

d Measured in C_5_D_5_N, 400 MHz.

**Fig. 1:**
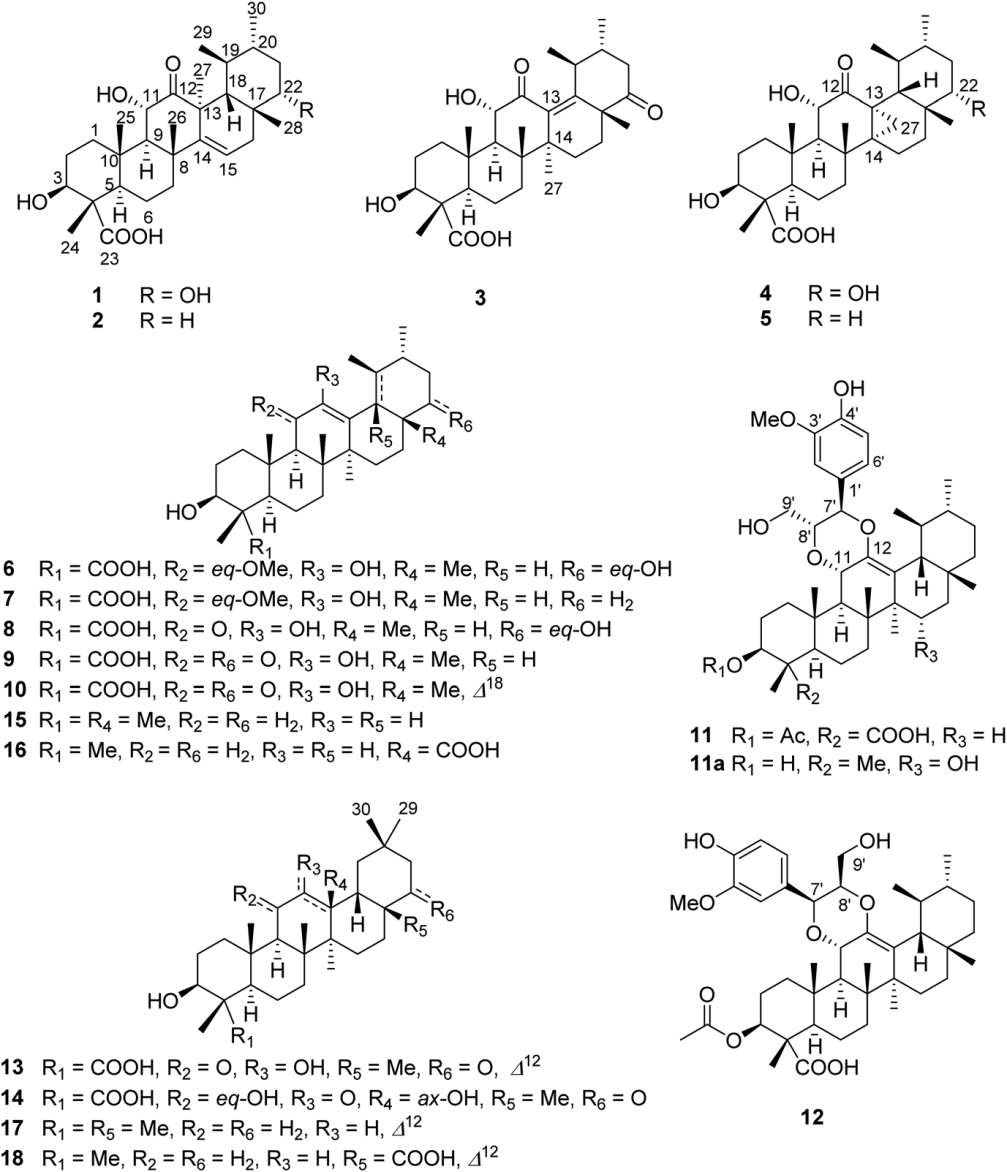
Triterpenoids 1–18 from *Stewartia sinensis*.

**Fig. 2:**
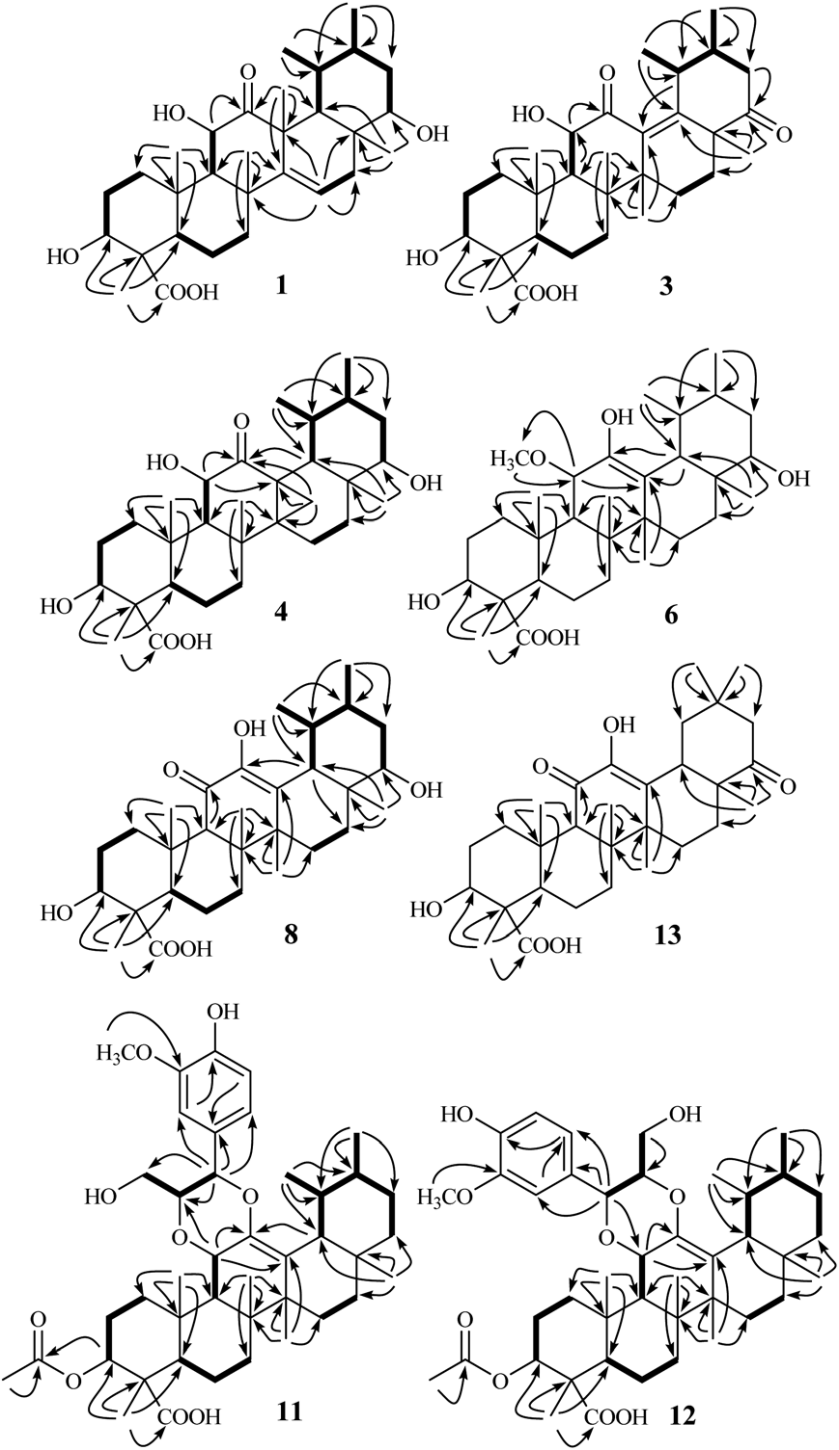
^1^H–^1^H COSY and observed key HMBC correlations of triterpenoids 1, 3, 4, 6, 8, and 11–13.

**Fig. 3:**
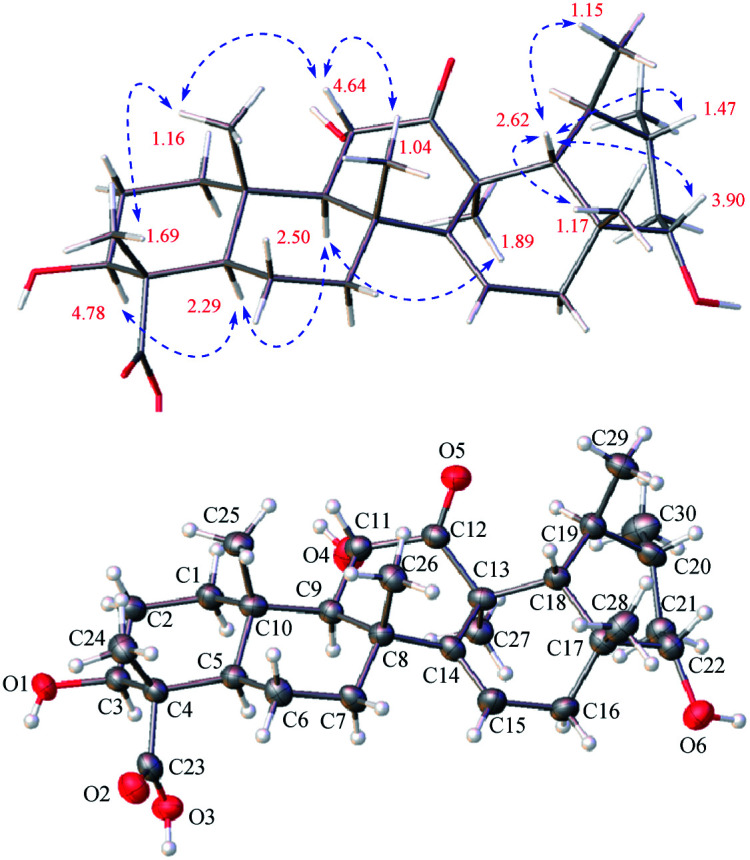
Observed key ROE correlations and the ORTEP drawing of 1.

The relative configuration of 1 was assigned by analysis of the key proton–proton coupling constants and ROESY interactions (Fig. 3). The magnitudes of *J*_H-2β/H-3_ (10.1 Hz), *J*_H-5/H-6β_ (11.0 Hz), *J*_H-9/H-11_ (11.0 Hz), and *J*_H-21α/H-22_ (10.6 Hz) indicated that H-3, H-5, H-9, H-11, and H-22 were in axial positions. In the ROESY spectrum, the correlations between H-3/H-5, H-5/H-9, H-9/H_3_-27, and H_3_-27/H_3_-30 revealed their cofacial relationship and were arbitrarily assigned as α-oriented. In turn, the ROE interactions of H_3_-24/H_3_-25, H_3_-25/H-11, H-11/H_3_-26, H-18/H-20, H-18/H-22, H-18/H_3_-28, and H-18/H_3_-29 indicated their β-orientation. Finally, a single-crystal X-ray diffraction experiment with Ga Kα radiation for 1 defined its (3*S*,4*S*,5*R*,8*R*,9*R*,10*S*,11*S*,13*S*,17*R*,18*R*,19*S*,20*R*,22*S*) absolute configuration [absolute structure parameter: 0.19 (17)]. Based on the above findings, the structure of compound 1 was characterized as (3*S*,4*S*,5*R*,8*R*,9*R*,10*S*,11*S*,13*S*,17*R*,18*R*,19*S*,20*R*,22*S*)-3β,11α,22α-trihydroxy-12-oxo-isours-14-en-23-oic acid.

Stewartiacid B (2) was obtained as a white powder, and its molecular formula, C_30_H_46_O_5_, was deduced from the deprotonated ion at *m*/*z* 485.3665 [M − H]^−^ in its HRESIMS and the ^13^C NMR data (Table 1). The close similarity of the ^1^H and ^13^C NMR spectroscopic data of 2 with those of 1 indicated an isoursane-type triterpenoid nucleus. The difference of sixteen mass units between 1 and 2, and the absence of the signals from an oxymethine group [*δ*_H_ 3.74 (1H, dd, *J* = 11.8, 3.7 Hz, H-22), *δ*_C_ 78.6 (C-22)] when compared with those of 1, suggested the disappearance of 22-OH group in 2. This was confirmed by the HMBC cross-peak from H_3_-28 to C-22 (Fig. S1, ESI‡). The relative configuration of compound 2 was found to be the same as that of 1 by *J*-based configuration analysis and ROESY data (Fig. S2, ESI‡). Likewise, the absolute configurations of C-11 and C-13 in 2 were elucidated to be the same as those of 1, based on a similiar negative Cotton effect around 316 nm for the keto carbonyl group at C-12 in their electronic circular dichroism (ECD) curves (Fig. S23, ESI‡). Accordingly, the structure of 2 was defined as (3*S*,4*S*,5*R*,8*R*,9*R*,10*S*,11*S*,13*S*,17*R*,18*R*,19*S*,20*R*)-3β,11α-dihydroxy-12-oxo-isours-14-en-23-oic acid.

**Fig. 4:**
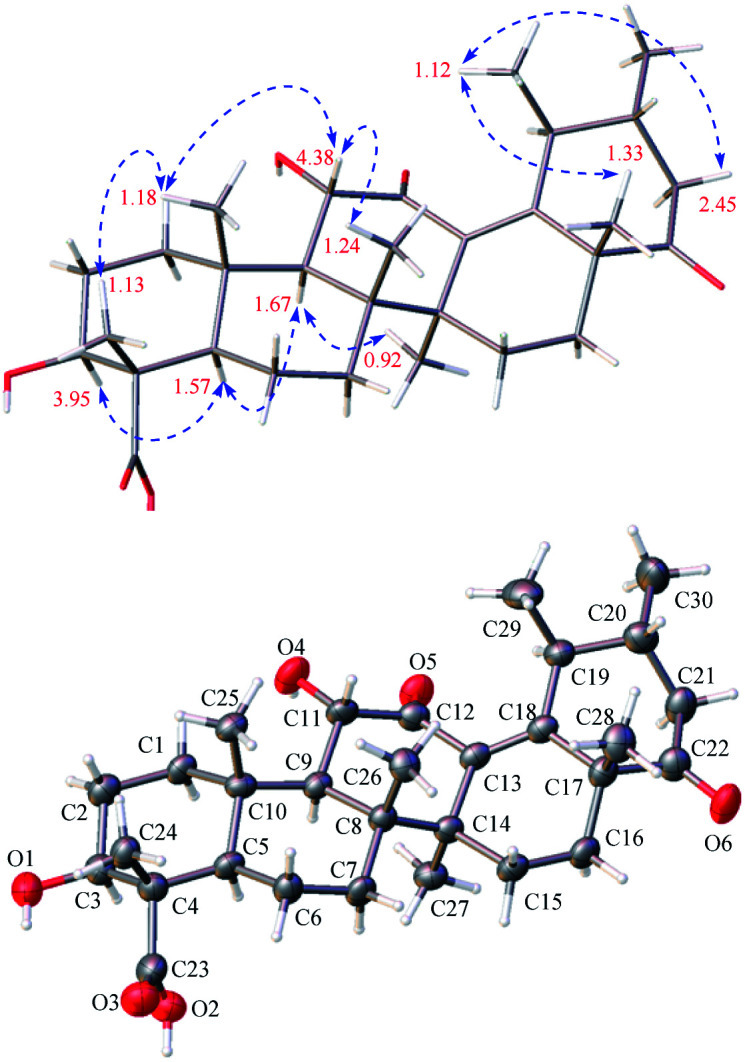
Observed key ROE correlations and the ORTEP drawing of 3.

Stewartiacid C (3) was obtained as colorless crystals from MeOH, with a molecular formula of C_30_H_44_O_6_ as deduced from the deprotonated ion peak at *m*/*z* 499.3069 [M − H]^−^ in its HRESIMS and the ^13^C NMR data (Table 1). Inspection of the ^1^H NMR spectroscopic data (Table 2) indicated the presence of five tertiary methyl groups [*δ*_H_ 0.92 (3H, s, Me-27), 1.13 (3H, s, Me-24), 1.18 (3H, s, Me-25), 1.24 (3H, s, Me-26), and 1.33 (3H, s, Me-28)] two secondary methyl groups [*δ*_H_ 1.03 (3H, d, *J* = 6.5 Hz, Me-30) and 1.12 (3H, d, *J* = 7.1 Hz, Me-29)], and two oxymethine resonances at *δ*_H_ 3.95 (1H, dd, *J* = 11.5, 5.2 Hz, H-3) and 4.38 (1H, d, *J* = 11.5 Hz, H-11). A total of 30 carbon signals, including two ketone carbonyls at *δ*_C_ 209.8 (C-12) and 216.5 (C-22), a carboxyl carbon at *δ*_C_ 181.5 (C-23), two olefinic carbons at *δ*_C_ 141.9 (C-13) and 147.9 (C-18), and two oxymethine carbons at *δ*_C_ 76.0 (C-3) and 78.4 (C-11), were displayed in its ^13^C NMR spectrum. Further HMBC correlations from H_3_-27 to C-8/C-13/C-14/C-15, and from H_3_-28/H_3_-29 to C-18 revealed that compound 3 is a γ-amyrin analogue featuring an uncommon Δ^13(18)^ double bond. The two oxymethine groups were anchored at C-3 and C-11 based on the HMBC correlations from H_3_-24 to C-3 and from H-9 to C-11 (Fig. 2). Similarly, the HMBC cross-peaks from H-11 to C-12 and from H_3_-28 to C-22 positioned the two ketone groups at C-11 and C-22, respectively. In addition, as with compounds 1 and 2, the carboxyl group at C-23 was evident from the distinct HMBC cross-peak from H_3_-24 to C-23 (*δ*_C_ 181.5). The relative configuration of 3 was determined *via* analysing the coupling constants and ROESY data. The large *J* values between H-2β and H-3 (11.5 Hz) and between H-9 and H-11 (11.5 Hz) were indicative of their *trans*-diaxial relationship. The ROE correlations (Fig. 4) of H-3/H-5, H-5/H-9, and H-9/H_3_-27 indicated that H-3, H-5, H-9, and H_3_-27 assumed α-axial orientations. Meanwhile, the ROE correlations of H_3_-24/H_3_-25, H_3_-25/H-11, H-11/H_3_-26, and H_3_-28/H_3_-29 suggested these protons to be β-oriented in 3. Thus, the structure of 3 was defined as 3β,11α-dihydroxy-12,22-dioxo-urs-13(18)-en-23-oic acid. Finally, the absolute configuration (3*S*,4*S*,5*R*,8*R*,9*R*,10*S*,11*S*,14*S*,17*R*,19*S*,20*R*) of 3 was unequivocally established by a single-crystal X-ray diffraction analysis [Flack parameter = −0.01 (19)] using Ga Kα radiation (Fig. 4 and Table S2 in ESI‡).

**Fig. 5:**
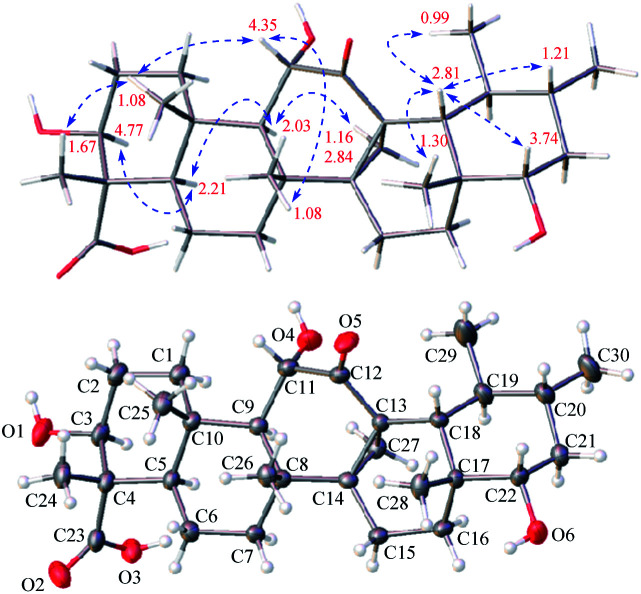
Observed key ROE correlations and the ORTEP drawing of 4.

Stewartiacid D (4) was obtained as colorless crystals (in MeOH), with the same molecular formula, C_30_H_46_O_6_, as 1 based on the HRESIMS and ^13^C NMR data (Table 1). Its ^1^H (Table 3) and ^13^C (Table 1) NMR data resembled those of 1, with major differences being observed for C-13 through C-16, C-18, and C-27. Unlike compound 1, signals for the 27-Me group was absent in the NMR spectra of 4, while instead, those typical for a methylene group [*δ*_H_ 2.84, 1.16 (ABq, *J* = 5.6 Hz, H_2_-27), *δ*_C_ 18.4 (C-27)] in a cyclopropyl ring appeared. This implied that 4 possesses a 13,27-cycloursane-type hexacyclic triterpenoid framework. This deduction was confirmed by the HMBC cross-peaks from H_2_-27 to C-12, C-13, and C-14 (Fig. 2). As shown in Fig. 5, the ROE correlation of H_b_-27 (*δ*_H_ 1.16) with H-9 positioned the cyclopropyl ring in the α-face of the molecule. The absolute configuration of 4 was established to be (3*S*,4*S*,5*R*,8*R*,9*R*,10*S*,11*S*,13*S*,14*R*,17*R*,18*R*,19*S*,20*R*,22*S*) by a single-crystal X-ray diffraction experiment using Ga Kα radiation (Fig. 5 and Table S3 in ESI‡). Taken together, the structure of compound 4 was identified as (3*S*,4*S*,5*R*,8*R*,9*R*,10*S*,11*S*,13*S*,14*R*,17*R*,18*R*,19*S*,20*R*,22*S*)-3β,11α,22α-trihydroxy-12-oxo-13α,27-cycloursan-23-oic acid.

**Table 3:** ^1^H NMR data^a^ (*δ* in ppm, *J* in Hz, 400 MHz) of 4–8

No.	4^b^	5^b^	6^b^	7^b^	8^c^
1α	1.99, m	1.99, m	1.67, m	1.69, m	1.16, m
1β	2.09, m	2.09, m	2.50, br d (13.3)	2.54, br d (13.5)	2.80, br d (13.6)
2a	2.00, m	2.01, m	1.99, m	2.02, m	1.72, m
2b	2.04, m	2.07, m	2.05, m	2.04, m	1.65, m
3	4.77, dd (9.4, 5.6)	4.77, dd (9.7, 6.0)	4.76, dd (10.6, 5.1)	4.79, dd (11.0, 5.1)	3.98, dd (10.0, 5.8)
5	2.21, br d (12.0)	2.20, br d (11.4)	2.13, br d (11.2)	2.15, br d (11.2)	1.52, br d (11.1)
6α	1.68, m	1.68, m	1.60, m	1.64, m	1.16, m
6β	1.80, m	1.81, m	1.77, m	1.78, m	1.63, m
7α	1.46, m	1.43, m	1.69, m	1.72, m	1.43, m
7β	1.64, m	1.64, m	1.29, m	1.30, m	1.74, m
9	2.03, d (7.4)	2.00, d (7.3)	2.21, d (9.7)	2.21, d (9.6)	2.58, s
11	4.35, d (7.4)	4.32, d (7.3)	4.52, d (9.7)	4.51, d (9.6)	
15α	1.62, m	1.52, br dd (13.8, 5.0)	1.02, m	0.92, m	1.23, m
15β	1.79, m	1.76, m	1.79, m	1.76, m	1.84, m
16α	1.24, m	1.40, m	1.79, m	0.79, m	1.82, m
16β	1.86, m	0.69, br dd (13.5, 5.4)	1.94, m	2.02, m	1.45, m
18	2.81, d (9.3)	2.68, d (10.7)	2.89, d (11.2)	2.80, d (11.1)	2.49, d (11.2)
19	0.97, m	0.87, m	1.60, m	1.48, m	1.51, m
20	1.21, m	2.01, m	1.24, m	1.07, m	1.18, m
21α	1.42, m	1.00, m	1.69, m	1.37, m	1.41, m
21β	1.79, m	1.29, m	1.90, m	1.29, m	1.64, m
22α	3.74, dd (11.8, 3.7)	1.35, m	3.70, dd (11.5, 3.9)	1.44, m	3.37, dd (11.7, 4.2)
22β		1.37, m		1.38, m	
24	1.67, s	1.67, s	1.68, s	1.73, s	1.11, s
25	1.08, s	1.07, s	1.22, s	1.23, s	1.17, s
26	1.08, s	1.03, s	1.16, s	1.14, s	1.16, s
27	2.84, d (5.6)	2.80, d (5.6)	1.32, s	1.29, s	1.41, s
	1.16, d (5.6)	1.06, d (5.6)			
28	1.30, s	0.90, s	1.32, s	0.93, s	0.94, s
29	0.99, br s	0.97, d (6.2)	1.23, d (7.7)	1.24, d (4.7)	0.81, d (6.2)
30	0.85, d (6.4)	0.81, d (5.6)	0.97, d (6.3)	0.94, d (4.8)	0.98, d (6.2)
OMe			3.42, s	3.41, s	

a Assignments were made by a combination of 1D and 2D NMR experiments.

b Measured in C_5_D_5_N.

c Measured in CD_3_OD.

Stewartiacid E (5) was obtained as a white powder, and the close similarity of its UV, ^1^H and ^13^C NMR spectroscopic data with those of 4 indicated a common 12-oxo-13α,27-cycloursan-23-oic acid nucleus. The molecular formula of 5, C_30_H_46_O_5_, was obtained *via* its HRESIMS (*m*/*z* 509.3238 [M + Na]^+^) and ^13^C NMR data (Table 1). The difference of sixteen mass units between 4 and 5, and the disappearance of resonances from the oxymethine group at C-22 in 5 suggested the absence of OH-22, which was confirmed by the HMBC correlations (Fig. S1, ESI‡). The relative configuration of compound 5 was found to be the same as that of 4 by *J*-based configuration analysis and ROESY data (Fig. S2, ESI‡). Its absolute configuration was then determined by comparing the ECD data (Fig. S52, ESI‡) with those of compound 4. The ECD curve of 5, showing a positive Cotton effect around 292 nm for the carbonyl group, was in good agreement with that of 4. This suggested the absolute configurations of C-11 (*S*) and C-13 (*S*) in 5 to be the same as 4. Accordingly, the structure of 5 was defined as (3*S*,4*S*,5*R*,8*R*,9*R*,10*S*,11*S*,13*S*,14*R*,17*R*,18*R*,19*S*,20*R*)-3β,11α-dihydroxy-12-oxo-13α,27-cycloursan-23-oic acid.

**Fig. 6:**
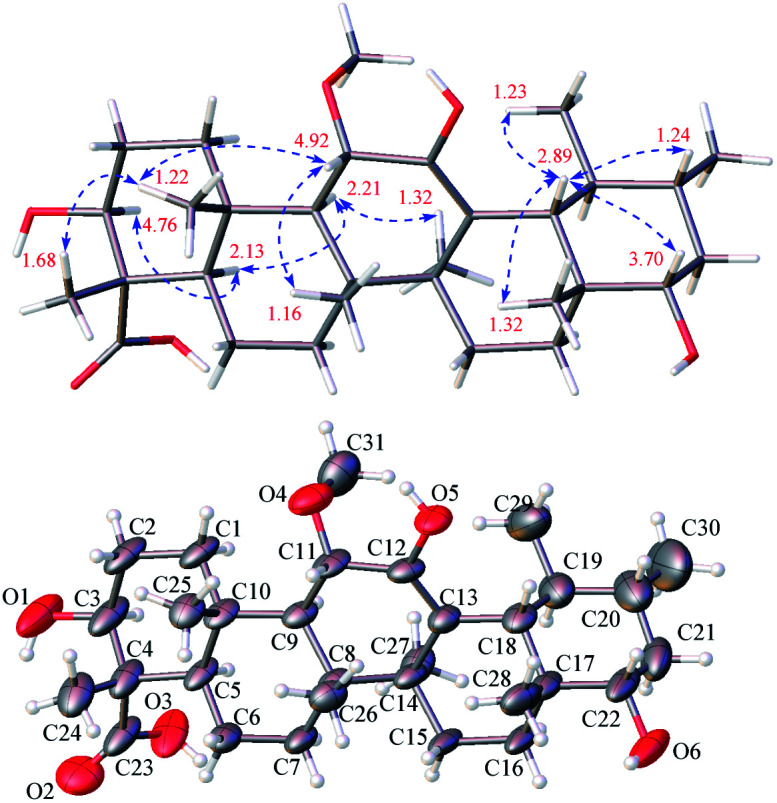
Observed key ROE correlations and the ORTEP drawing of 6.

Stewartiacid F (6) was obtained as colorless crystals from MeOH. The HRESIMS spectrum displayed a deprotonated ion at *m*/*z* 517.3665 [M − H]^−^, establishing the molecular formula C_31_H_50_O_6_ for 6. Its ^1^H and ^13^C NMR data (Tables 1 and 3) were similar to those of compounds 15 (ref. 22) and 16,^23^ suggesting a common ursane-type skeleton for 6. Detailed comparisons suggested that the NMR data of 6 were closely related to those of 11α-methoxy-urs-12-ene-3β,12-diol, which was previously isolated from *Siphonodon celastrineus*.^26^ The differences between 6 and this known compound were related to the presence of additional signals ascribed to a secondary hydroxy group at C-22 (*δ*_C_ 78.5; *δ*_H_ 3.70) and a carboxyl group at C-23 (*δ*_C_ 180.5) based on the key HMBC correlations from H_3_-28 to C-22 and from H_3_-24 to C-23 in 6 (Fig. 2). The α*-*orientation of H-22 and 23-COOH groups were then confirmed by analyses of the vicinal proton coupling constants (for H-22) and ROE correlations (Fig. 6). Hence, the structure of compound 6 was concluded as 11α-methoxy-3β,12,22α-trihydroxy-urs-12-en-23-oic acid. Finally, the absolute configuration (3*S*,4*S*,5*R*,8*R*,9*R*,10*S*,11*S*,14*S*,17*R*,18*R*,19*S*,20*R*,22*S*) of 6 was established by a single-crystal X-ray diffraction experiment using Ga Kα radiation [Flack parameter = 0.10 (8)] (Fig. 6 and Table S4 in ESI‡).

On the basis of an HRESIMS ion at *m*/*z* 501.3577 [M − H]^−^, the molecular formula of stewartiacid G (7) was determined to be C_31_H_50_O_5_, with one *O*-atom less than that of 6. Inspection of its NMR data (Tables 1 and 3) implied that its structure resembled that of 6, with the only difference being the absence of OH-22 group in 7. This change was corroborated by the observation of methylene resonances for CH_2_-22 (*δ*_H_ 1.44 m, 1.38 m; *δ*_C_ 42.1) and the HMBC cross-peak from H_3_-28 to C-22. The relative configuration of 7 was identical to that of 6, as evidenced by the ROESY experiment (Fig. S2, ESI‡). Furthermore, the ECD curve of 7, showing a positive Cotton effect around 207 nm for the enol group, was in good agreement with 6 (Fig. S71, ESI‡). This suggested the absolute configurations of C-11 (*S*), C-14 (*S*), and C-18 (*R*) in 7 to be the same as 6. Therefore, the structure of compound 7 was defined as (3*S*,4*S*,5*R*,8*R*,9*R*,10*S*,11*S*,14*S*,17*R*,18*R*,19*S*,20*R*)-11α-methoxy-3β,12-dihydroxy-urs-12-en-23-oic acid.

**Fig. 7:**
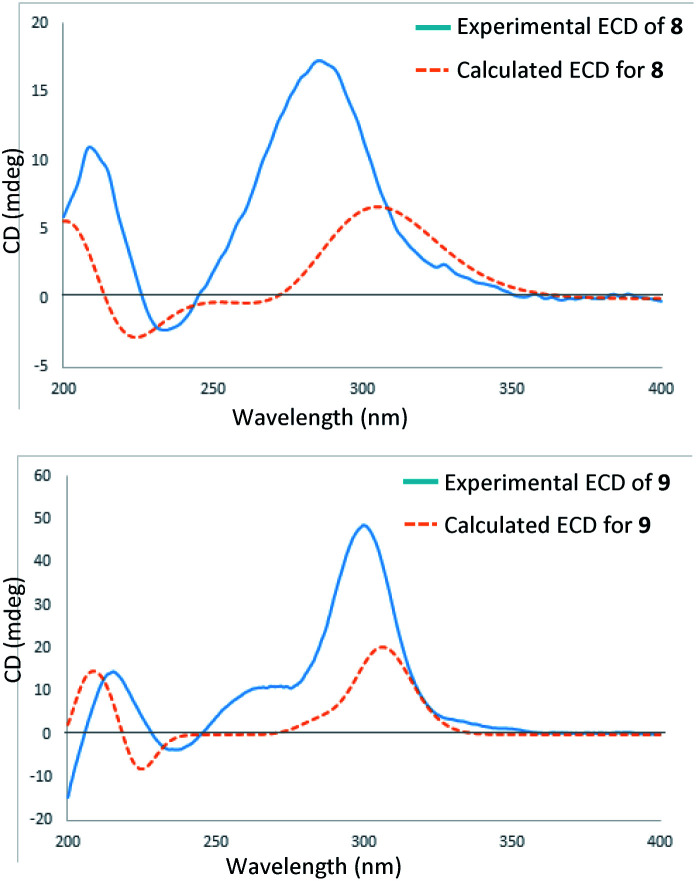
Experimental and calculated ECD spectra of 8 and 9.

In the HRESIMS spectrum of stewartiacid H (8), the [M − H]^−^ ion peak at *m*/*z* 501.3222 established its molecular formula to be C_30_H_46_O_6_. The ^1^H and ^13^C NMR spectroscopic data (Tables 1 and 3) closely resembled those of 6, indicating that they were structurally related. The difference lies in the substituent at C-11, where the carbonyl group in 8 replaced the methoxy group in 6, which was inferred from the observation of a conjugated carbonyl carbon (*δ*_C_ 196.2) in 8 along with the absence of methoxy signal at *δ*_H_ 3.42 (3H, s). Indeed, the presence of such a Δ^12,13^-11-one moiety in 8 was in accordance with the UV absorption at 287 nm. Further HMBC correlations of H-9/C-11, H-18/C-12, and H_3_-27/C-13 confirmed the above deduction (Fig. 2). The relative configuration of 8 was consistent with that of 6 based on their similar ROESY data (Fig. S2, ESI‡), and comparable coupling constants for the key protons H-3, H-5, H-18, and H-22 (Table 3). Accordingly, the structure of compound 8 was defined as 3β,12,22α-trihydroxy-11-oxo-urs-12-en-23-oic acid. The time-dependent density functional theory (TDDFT) ECD calculation at the CAM-B3LYP/def2-TZVP level (for details, please see Experimental section) was performed to elucidate the absolute configuration of 8. The calculated ECD spectrum of 8 gave a positive Cotton effect at *ca*. 300 nm (Fig. 7), well matching its experimental ECD spectrum (Fig. 7). Thus, the absolute configuration of 8 was finally assigned as (3*S*,4*S*,5*R*,8*R*,9*R*,10*S*,14*S*,17*R*,18*R*,19*S*,20*R*,22*S*).

Stewartiacid I (9) possesses a molecular formula of C_30_H_44_O_6_, as indicated by the ^13^C NMR data (Table 1) and the [M − H]^−^ ion at *m*/*z* 499.3065 in its HRESIMS. Its UV, ^1^H and ^13^C NMR spectroscopic data indicated that 9 was highly similar to 8, with the only distinction being the presence of a carbonyl group at C-22 in 9 rather than a hydroxy group in 8. This was confirmed by the chemical shift of C-22 deshielded to *δ*_C_ 218.0, in conjunction with the HMBC correlation (Fig. S1, ESI‡) from H_3_-28 to C-22. The relative configuration of 9 was assessed by the ROESY data (Fig. S2, ESI‡) and comparison of its NMR data to those of 8 and 9. Like 8, the absolute configuration of 9 was determinated as (3*S*,4*S*,5*R*,8*R*,9*R*,10*S*,14*S*,17*R*,18*R*,19*S*,20*R*) from the overlaid experimental and calculated ECD curves (Fig. 7). Therefore, the structure of 9 was defined as (3*S*,4*S*,5*R*,8*R*,9*R*,10*S*,14*S*,17*R*,18*R*,19*S*,20*R*)-3β,12-dihydroxy-11,22-dioxo-urs-12-en-23-oic acid.

The molecular formula of stewartiacid J (10) was determined as C_30_H_42_O_6_ by analysis of HRESIMS and ^13^C NMR data (Table 1), with two protons less than that of 9. The NMR spectroscopic data of 10 were comparable to those of 9, except for the presence of the Δ^18(19)^ double bond [*δ*_H_ 1.59, s (H_3_-29); *δ*_C_ 130.7 (C-18), 138.6 (C-19)]. This was corroborated by the HMBC correlations from H_3_-29/H_3_-30 to C-19 and from H_3_-28 to C-18 (Fig. S1, ESI‡). The relative configuration of 10 was consistent with 9 based on the ROE correlations of H_3_-24/H_3_-25, H_3_-25/H_3_-26, H-18/H_3_-29, H-18/H_3_-28, H-3/H-5, H-5/H-9, and H-9/H_3_-27 (Fig. S2, ESI‡). Thus, compound 10 was identified as 3β,12-dihydroxy-11,22-dioxo-urs-12,18-dien-23-oic acid.

**Fig. 8:**
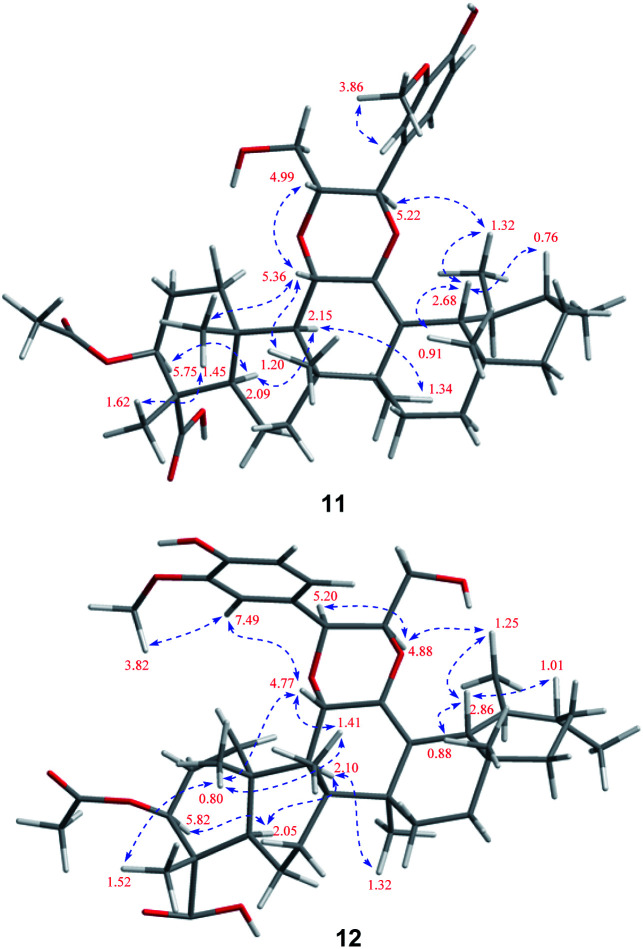
Observed key ROE correlations of 11 and 12.

Stewartiacid K (11) was isolated as a white powder and its molecular formula, C_42_H_60_O_9_, was determined by HRESIMS (*m*/*z* 731.4111 [M + Na]^+^, calcd for C_42_H_60_O_9_Na, 731.4130) and ^13^C NMR data (Table 5), corresponding to 13 IHDs. Interpretation of the 1D NMR data suggested that 11 possesses a typical urs-23-oic acid fragment as in compounds 1–10 conjugated with an aromatic moiety. In addition to the NMR signals assignable to the triterpenoid moiety along with an acetyl substituent [*δ*_H_ 1.91, 3H, s; *δ*_C_ 169.9, 21.3], the remaining resonances were typical for a phenylpropanoid unit featuring a 1,3,4-trisubstituted benzene ring [*δ*_H_ 7.37 (br s, H-2′), 7.28 (d, *J* = 7.9 Hz, H-5′), 7.23 (br d, *J* = 7.9 Hz, H-6′)]. The aforementioned data were closely related to those of 11α,12-[2-(hydroxymethyl)-3-(4-hydroxy-3-methoxyphenyl)ethane-1,2-dioxy]-urs-12-en-3β,15α-diol (11a), which was previously isolated from *Siphonodon celastrineus*.^26^ As with 11a, compound 11 is a hybrid of ursane-type triterpenoid and phenylpropanol by forming a unique 1,4-dioxane ring. The oxygen bridge from C-11 to C-8′ was verified by the HMBC cross-peak between H-11 and C-8′ (Fig. 2). Another oxygen bridge from C-12 to C-7′, without the observation of clear HMBC cross-peak between H-7′ and C-12, was evidenced based on the molecular formula, from which the IHDs required the formation of an additional heterocyclic ring in the structure of 11. However, the main differences between 11 and 11a were the substitution patterns at C-3 and C-15 as well as the methyl at C-23 being oxidized to a carboxyl group in 11. The highly downfield shifted H-3 (*δ*_H_ 5.75) in 11 and the HMBC correlation from H-3 to the acetyl carbonyl carbon suggested that an acetoxy moiety was located at C-3 in 11. Differing from 11a the 15-OH was absent in 11, which was corroborated by the HMBC correlation from H_3_-27 to C-15. In addition, as with compounds 1–10, the carboxyl group at C-23 was confirmed by the distinct HMBC correlations from H-3/H_3_-24 to C-23. The relative configuration of 11 was determined by analysing the vicinal coupling constants of key protons (Table 5) and ROE correlations (Fig. 8). The large *J* values between H-2β and H-3 (12.0 Hz), between H-9 and H-11 (8.8 Hz), and between H-18 and H-19 (10.7 Hz) were indicative of their *trans*-diaxial relationship. The ROE correlations of H-3/H-5, H-5/H-9, and H-9/H_3_-27 indicated that H-3, H-5, H-9, and H_3_-27 assumed α-axial orientations. In turn, the ROE correlations of H_3_-24/H_3_-25, H_3_-25/H-11, H_3_-26/H-11, H_3_-28/H-18, and H_3_-29/H-18 suggested these protons to be β-oriented in 11. Meanwhile, the magnitude of *J*_H-7′/H-8′_ (10.4 Hz) indicated a *trans* configuration of the dioxane ring,^27^ and the ROE correlation between H-8′ and H-11 supported their β-orientation. The ROE correlation between H-2′ and the methoxy group also confirmed the latter to be located at C-3′. Thus, the structure of 11 was defined as 11α,12-[2-(hydroxymethyl)-3-(4-hydroxy-3-methoxyphenyl)ethane-1,2-dioxy]-3β-acetoxy-urs-12-en-23-oic acid.

**Fig. 9:**
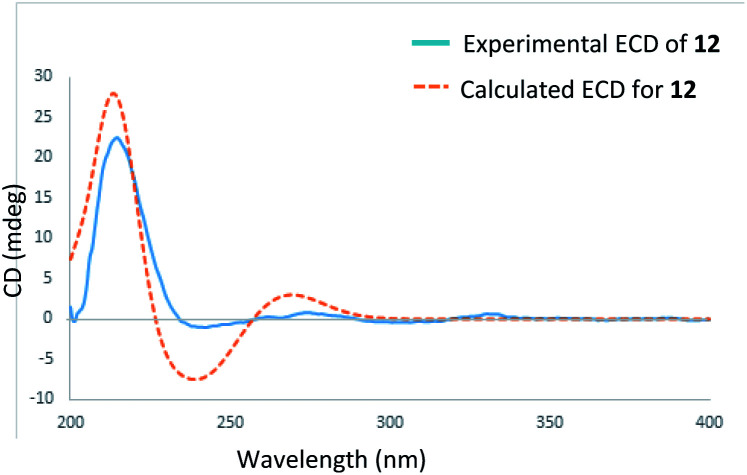
Experimental and calculated ECD spectra of 12.

Stewartiacid L (12) was found to possess the same molecular formula (C_42_H_60_O_9_) as that of 11 based on the HRESIMS ion at *m*/*z* 731.4125 ([M + Na]^+^, calcd for C_42_H_60_O_9_Na, 731.4130). Its ^1^H and ^13^C NMR data resembled those of 11, with noticeable difference being observed around the 1,4-dioxane ring (Table 5). Further HMBC NMR data (Fig. 2) showed a clear correlation from H-7′ to C-11, suggesting that the two oxygen bridges in 12 were formed between C-7′ and C-11, and between C-8′ and C-12. The small coupling constant (*J*_H-7′/H-8′_ = 3.7 Hz) was indicative of a 7′,8′-*cis* relative configuration. H-7′ and H-8′ were then assigned as α-orientation from the ROE correlations of H-2′/H-6′ with H-11, and of H′-8 with H_3_-29, as well as the absence of correlation between H-11 and H-7′ (Fig. 8). Similar to compounds 8 and 9, the absolute configuration of 12 was also assessed by TDDFT ECD calculation at the CAM-B3LYP/def2-TZVP level. From the overlaid experimental and calculated ECD curves of 12 (Fig. 9), its absolute configuration was determined as (3*S*,4*S*,5*R*,8*R*,9*R*,10*S*,11*S*,14*S*,17*R*,18*R*,19*S*,20*R*,7′*S*,8′*R*). Accordingly, the structure of 12 was established as (3*S*,4*S*,5*R*,8*R*,9*R*,10*S*,11*S*,14*S*,17*R*,18*R*,19*S*,20*R*,7′*S*,8′*R*)-11α,12-[3-(hydroxymethyl)-2-(4-hydroxy-3-methoxyphenyl) ethane-1,2-dioxy]-3β-acetoxy-urs-12-en-23-oic acid. Interestingly, the formation of the 1,4-dioxane rings in compounds 11 and 12 has been considered to be resulted *via* a free radical coupling reaction.^26^

Stewartiacid M (13) has the same molecular formula (C_30_H_44_O_6_) as compound 9 based on the HRESIMS and ^13^C NMR data. Comparsion of the 1D NMR data of 13 (Tables 1 and 6) and 9 revealed their structural similarity, with the only distinction being the presence of a pair of geminal methyl groups at C-20 (*δ*_H_ 1.03, s; 0.90, s) in 13 rather than two secondary methyl groups in 9. This implied that, unlike compounds 1–12 with an ursane-type skeleton, 13 is an oleanane-type triterpenoid, as with the co-occurring compounds 17 (ref. 22) and 18.^23^ Further HMBC NMR experiment confirmed the planar structure of 13 as depicted in Fig. 2. As for its relative configuration, the large *J* values between H-2β and H-3 (11.0 Hz), H-5 and H-6β (11.2 Hz), and between H-18 and H-19α (12.2 Hz) were indicative of their axial orientations. The ROE correlations (Fig. S2, ESI‡) of H-3/H-5, H-5/H-9, and H-9/H_3_-27 implied that these protons assumed the α-axial orientation. Meanwhile, the ROE correlations of H_3_-24/H_3_-25, H_3_-25/H_3_-26, H-18/H_3_-28, and H-18/H_3_-30 were indicative of their β-orientation. Thus, the structure of 13 was defined as 3β,12-dihydroxy-11,22-dioxo-olean-12-en-23-oic acid.

**Fig. 10:**
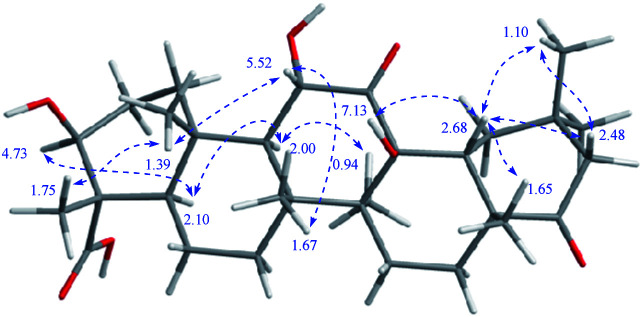
Observed key ROE correlations of 14.

Stewartiacid N (14) has a molecular formula of C_30_H_46_O_7_, by its HRESIMS and ^13^C NMR data (Table 1). The NMR spectroscopic data of 14 showed a high similarity to camellisin C, which has been previously reported from the roots of *Camellia sinensis*.^28^ The main differences between 14 and camellisin C being observed for C-3, C-4, C-5, and C-24 (14: *δ*_C_ 75.3, 55.2, 51.9, and 11.6, resp.; camellisin C:^28^*δ*_C_ 78.1, 50.0, 57.1, and 24.9, resp.). This implied that they should be a pair of C-4 epimers. As for 14, a clear ROE correlation between H_3_-24 (*δ*_H_ 1.75) and H_3_-25 (*δ*_H_ 1.39) undoubtedly allowed Me-24 to be β-axially oriented (Fig. 10). Consistent with this, the chemical shifts assigned to ring A in 14 were closely related to those of the other co-occurring triterpenoids 1–13 featuring a common α-oriented carboxyl group. As with camellisin C,^28^ the 12-OH was concluded to adopt the β-orientation as evident from the ROE correlations of 12-OH (*δ*_H_ 7.13) with H-18 (*δ*_H_ 2.68) and H_3_-26 (*δ*_H_ 1.67). Thus, the configuration at C-4 in camellisin C^28^ seems to be wrongly assigned, and its structure should be revised as 3β,11α,13β-trihydroxy-11,22-dioxo-olean-24-oic acid. Herein, the ^1^H and ^13^C NMR data for 3β,11α,13β-trihydroxy-11,22-dioxo-olean-23-oic acid (14) are accurately assigned in this study.

The ATP-citrate lyase (ACL) is a promising target for the treatment of metabolic disorders and cancer,^29^ since it affects nutrient catabolism and cholesterol and fatty acid biosynthesis. Recently, a series of diterpenoids and norditerpenoids from the endangered conifers *Pseudotsuga sinensis*^21*b*^ and *Picea brachytyla*^21*c*^ were found to have significant ACL inhibitory effects. In the present study, all the isolated compounds (1–18) were evaluated for their ACL inhibitory activities. Among them, compound 11 displayed the most potent inhibitory effect, with an IC_50_ value of 2.8 μM. Compounds 7 and 12 showed moderate inhibition against ACL, with IC_50_ values of 12.5 and 10.6 μM, respectively (Table 7). The rest isolates were inactive (inhibition ration < 50% at 20 μM). The known inhibitor BMS 303141 was used as the positive control (IC_50_: 0.4 ± 0.1 μM).^30^ To our knowledge, this is the first report on the naturally occurring ursane-type triterpenoids as ACL inhibitors. In addition, compounds 1–18 were also tested for their inhibitory activities against NF-κB, and only 11 showed moderate inhibition (IC_50_: 16.8 μM). Bortezomib (PS-341) was used as the positive control (IC_50_: 0.06 ± 0.01 μM) (Table 7).^31^

## Conclusions

In the present study, 14 new C-23 carboxylated triterpenoid derivatives, stewartiacids A–N, were isolated from the twigs and leaves of the endangered ornamental plant *Stewartia sinensis*. This is the first phytochemical and pharmacological investigation on this plant. Unlike 28-carboxylated ursane-/oleanane-type triterpenoids (*e.g.*, compounds 16 and 18) that are widely distributed in the plant kingdom, the ursane-/oleanane-type triterpenoids featuring a 23-COOH group are quite rare in nature. To our knowledge, only a dozen C-23 monocarboxylated derivatives have been so far reported (data from The Dictionary of Natural Products on DVD). From a chemical point of view, stewartiacids A (1) and B (2) are rare isoursenol derivatives featuring the 27-methyl group at C-13,^24,25^ whereas stewartiacid C (3) is a rare 12-oxo-γ-amyrin analogue.^25*a*^ Stewartiacids D (4) and E (5) are two 13,27-cycloursane-type triterpenoids. Stewartiacids K (11) and L (12) are uncommon ursane-type triterpene and phenylpropanol hybrids by forming a unique 1,4-dioxane bridge.^26,32^ The absolute configurations of compounds 1–9 and 12 were well established either by single crystal X-ray diffraction or by ECD analyses. The absolute configurations of the rest new stewartiacids (*i.e.*, 10, 11, 13, and 14) are still open; however, they could be assigned as shown in both Fig. 1 and the Experimental [*e.g.*, (3S*,4S*,5R*,8R*,9R*,10S*,14S*,17R*,20R*)-10] based on biogenetic considerations.

Stewartiacids G (7), K (11), and L (12) showed inhibitory effects against ACL. Compound 11 also exhibited inhibition on NF-κB. The above findings may provide useful clues for discovery and development of new therapeutic or preventive agents for treatment of metabolic disorders and other ACL or NF-κB related diseases. Moreover, the identification of new molecules from endangered plants reveals the importance in conservation efforts to prevent species diversity loss in the control of emerging druggable targets.

## Experimental section

### General experimental procedures

Melting points were measured with a WRS-1B capillary melting point apparatus. Optical rotations were obtained with a Rudolf Autopol IV at 22 °C. UV and IR spectra were recorded on a Hitachi U-2900E UV spectrophotometer and a Thermo Scientific Nicolet Is5 FT-IR spectrometer, respectively. ECD spectra were recorded on a JASCO-810 spectropolarimeter. ESI-MS and HRESIMS were acquired on an Agilent 1100 LC/MSD mass spectrometer and an AB Sciex Triple TOF 5600 spectrometer, respectively. X-ray crystallographic data were measured on a Bruker Apex Duo Diffractometer (Ga Kα). 1D and 2D NMR spectra were performed on a Bruker Avance III 400 MHz or a Bruker Avance III 600 MHz spectrometers using the residual solvent signals as the internal standard. All chemical shifts were expressed in ppm. Semi-preparative HPLC was performed on a Waters e2695 system coupled with a 2998 photodiode array (PDA) detector and a 2424 evaporative light-scattering detector (ELSD). A SunFire C18 column (5 μM, 10 × 250 mm; flow rate: 3.0 mL min^−1^) and a X-bridge C18 column (5 μM, 10 × 250 mm; flow rate: 3.0 mL min^−1^) were utilized. Thin-layer chromatography (TLC) was performed on pre-coated plates (GF_254_, 0.25 mm, Kang-Bi-Nuo Silysia Chemical Ltd., Yantai, China). TLC spots were visualized under UV light (254 or 365 nm) and by spraying with 5% H_2_SO_4_/vanillin followed by heating to 120 °C.

### Plant material

The twigs and leaves of *Stewartia sinensis* (family Theaceae) were collected by one of the authors (Mr Dao-An Xiao) from Mingyue Mountain in Yichun, Jiangxi Province of China, in October 2017. The plant was also identified by Mr Xiao (College of Chemistry and Bioengineer, Yichun University, Jiangxi Province, PR China). A voucher specimen (no. 20171007) was deposited at the Herbarium of the School of Pharmacy at Fudan University.

### Extraction and isolation

The air-dried, powdered twigs and leaves (3.5 kg) of *S. sinensis* were extracted with 90% MeOH (5 × 6 L, each time for 24 h) at room temperature. The resultant dark green residue (315 g, semidry) was suspended in H_2_O (1.5 L) and extracted successively with petroleum ether (PE, 3 × 1.5 L), EtOAc (3 × 1.5 L), and *n*-BuOH (3 × 1.5 L). The EtOAc-soluble extract (52.2 g) was subjected to a silica gel column with a stepwise gradient-elution, employing a mixture of PE–EtOAc (30 : 1 → 20 : 1 → 10 : 1 → 5 : 1 → 1 : 1 → 1 : 5 → neat EtOAc) as solvents, to afford nine fractions (Fr.1–Fr.9), according to TLC analysis. Fr.2 (910 mg) was separated by gel permeation chromatography (GPC) on Sephadex LH-20 (CH_2_Cl_2_–MeOH, 2 : 1), followed by semi-preparative HPLC (MeOH–H_2_O, 100 : 0) to afford compounds 15 (2.1 mg, *t*_R_ = 24.8 min) and 17 (3.1 mg, *t*_R_ = 27.5 min). Fr.3 (1.05 g) was fractioned by Sephadex LH-20 (MeOH), and five fractions (Fr.3A-Fr.3E) were obtained. Fr.3B (127 mg) was purified by semi-preparative HPLC (MeOH–H_2_O, 90 : 10) to afford compounds 16 (2.4 mg, *t*_R_ = 30.1 min) and 18 (3.4 mg, *t*_R_ = 31.6 min). Fr.4 (410 mg) was fractioned on a MCI column with a step gradient elution of MeOH–H_2_O (50 : 50 → 70 : 30 → 85 : 15 → 100 : 0) and six fractions (Fr.4A–Fr.4F) were collected. Separation of Fr.4C (63 mg) over Sephadex LH-20 (MeOH) and semi-preparative HPLC (MeOH–H_2_O, 88 : 12) afforded compounds 12 (1.7 mg, *t*_R_ = 18.8 min) and 11 (5.2 mg, *t*_R_ = 21.3 min). Fr.5 (1.9 g) was fractioned on an MCI column with a step gradient elution of MeOH–H_2_O (50 : 50 → 70 : 30 → 85 : 15 → 100 : 0) and seven fractions (Fr.5A–Fr.5G) were obtained. Fr.5B (74.0 mg) was purified by semi-preparative HPLC to furnish compound 14 (3.0 mg, *t*_R_ = 14.7 min). Fr.5C (197.0 mg) was further separated on Sephadex LH-20 (MeOH) to give subfractions Fr.5C-1–Fr.5C-6. Fr.5C-3 (22 mg) was purified by semi-preparative HPLC (MeOH–H_2_O, 80 : 20) to afford compound 4 (1.5 mg, *t*_R_ = 12.5 min). By employing the same HPLC (MeCN–H_2_O, 47 : 53) system, compounds 9 (0.6 mg, *t*_R_ = 18.9 min) and 13 (0.4 mg, *t*_R_ = 20.8 min) were purified from Fr.5C-4 (17.4 mg), whereas compound 10 (0.5 mg, *t*_R_ = 16.2 min) was purified from Fr.5C-5 (12.6 mg). Compound 3 (4.0 mg) was obtained from Fr.5D (130 mg) by Sephadex LH-20 (MeOH) followed by HPLC purification (MeOH–H_2_O, 93 : 7, *t*_R_ = 10.9 min). Purification of fraction Fr.5E (218 mg) by semi-preparative HPLC (MeOH–H_2_O, 93 : 7) afforded compounds 2 (3.0 mg, *t*_R_ = 13.3 min) and 7 (5.0 mg, *t*_R_ = 15.4 min). Fr.7 (3.5 g) was chromatographed over a silica gel column (CH_2_Cl_2_–MeOH, 20 : 1 → 10 : 1 → 5 : 1 → 1 : 1) to give six subfractions, Fr.7A–Fr.7F. Fr.7D (650 mg) was rechromatographed by Sephadex LH-20 (MeOH), and seven subfractions (Fr.7D-1–Fr.7D-7) were obtained. Purification of subfraction Fr.7D-4 (233 mg) by semi-preparative HPLC (MeOH–H_2_O, 78 : 22) yielded compounds 6 (30.0 mg, *t*_R_ = 10.3 min) and 8 (10.0 mg, *t*_R_ = 15.0 min). Compound 1 (5.0 mg) was purified from Fr.7F by Sephadex LH-20 (MeOH) followed by semi-preparative HPLC purification (MeOH–H_2_O, 75 : 25, *t*_R_ = 10.9 min). Fr.8 (1.1 g) was fractioned by Sephadex LH-20 (MeOH), and six subfractions (Fr.8A–Fr.8F) were generated. Fr.8C (488 mg) was purified by semi-preparative HPLC (MeOH–H_2_O, 90 : 10) to afford compound 5 (7.0 mg, *t*_R_ = 11.4 min).

### Stewartiacid A [(3*S*,4*S*,5*R*,8*R*,9*R*,10*S*,11*S*,13*S*,17*R*,18*R*,19*S*,20*R*,22*S*)-3β,11α,22α-trihydroxy-12-oxo-isours-14-en-23-oic acid, 1]

Colorless crystals (MeOH), mp 322.4–323.0; [*α*]^22^_D_ −7.8 (*c* 0.2, MeOH); UV (MeOH) *λ*_max_ (log *ε*) 203 (4.62) nm; ECD (*c* 3.98 × 10^−3^ M, MeOH) *λ*_max_ (Δ*ε*): 233 (−1.7), 316 (−1.4); IR (KBr) *v*_max_ 3476, 2945, 2920, 2868, 1706, 1674, 1464, 1384 1210, 1132, 1013, 801 cm^−1^; ^1^H and ^13^C NMR data, see Tables 1 and 2; ESIMS *m*/*z* 525 [M + Na]^+^, 501 [M − H]^−^; HRESIMS *m*/*z* 525.3182 [M + Na]^+^ (calcd for C_30_H_46_O_6_Na, 525.3187, *Δ* = −0.8 ppm).

### Stewartiacid B [(3*S*,4*S*,5*R*,8*R*,9*R*,10*S*,11*S*,13*S*,17*R*,18*R*,19*S*,20*R*)-3β,11α-dihydroxy-12-oxo-isours-14-en-23-oic acid, 2]

White powder; [*α*]^22^_D_ −4.6 (*c* 0.2, MeOH); UV (MeOH) *λ*_max_ (log *ε*) 207 (1.54) nm; ECD (*c* 4.05 × 10^−3^ M, MeOH) *λ*_max_ (Δ*ε*): 233 (−0.5), 316 (−0.4); IR (KBr) *v*_max_ 3451, 2967, 2918, 2863, 1705, 1661, 1621, 1459, 1382, 1207, 1153, 1008, 868 cm^−1^; ^1^H and ^13^C NMR data, see Tables 1 and 2; ESIMS *m*/*z* 485 [M − H]^−^; HRESIMS *m*/*z* 485.3268 [M − H]^−^ (calcd for C_30_H_45_O_5_, 485.3272, *Δ* = −0.9 ppm).

### Stewartiacid C [(3*S*,4*S*,5*R*,8*R*,9*R*,10*S*,11*S*,14*S*,17*R*,19*S*,20*R*)-3β,11α-dihydroxy-12,22-dioxo-urs-13(18)-en-23-oic acid, 3]

Colorless crystals (MeOH); [*α*]^22^_D_ −5.2 (*c* 0.16, MeOH); UV (MeOH) *λ*_max_ (log *ε*) 210 (1.24), 257 (1.15) nm; ECD (*c* 3.20 × 10^−3^ M, MeOH) *λ*_max_ (Δ*ε*): 220 (+3.9), 257 (−3.5), 289 (−3.5); IR (KBr) *v*_max_ 3441, 2972, 2932, 2860, 1696 (br.), 1644, 1457, 1379, 1339, 1205, 1060, 1015, 798, 726 cm^−1^; ^1^H and ^13^C NMR data, see Tables 1 and 2; ESIMS *m*/*z* 499 [M − H]^−^; HRESIMS *m*/*z* 499.3069 [M − H]^−^ (calcd for C_30_H_43_O_6_, 499.3065, *Δ* = 0.9 ppm).

### Stewartiacid D [(3*S*,4*S*,5*R*,8*R*,9*R*,10*S*,11*S*,13*S*,14*R*,17*R*,18*R*,19*S*,20*R*,22*S*)-3β,11α,22α-trihydroxy-12-oxo-13α,27-cycloursan-23-oic acid, 4]

Colorless crystals (MeOH), mp 272.0–273.1; [*α*]^22^_D_ +59.6 (*c* 0.2, MeOH); UV (MeOH) *λ*_max_ (log *ε*) 215 (3.43) nm; ECD (*c* 3.97 × 10^−3^ M, MeOH) *λ*_max_ (Δ*ε*): 214 (+3.2), 291 (+3.6); IR (KBr) *v*_max_ 3661, 2972, 2940, 2825, 1703, 1663, 1454, 1344, 1205, 1054, 1012 cm^−1^; ^1^H and ^13^C NMR data, see Tables 1 and 3; ESIMS *m*/*z* 501 [M − H]^−^, 1003 [2M − H]^−^; HRESIMS *m*/*z* 501.3277 [M − H]^−^ (calcd for C_30_H_45_O_6_, 501.3222, *Δ* = 1.1 ppm).

### Stewartiacid E [(3*S*,4*S*,5*R*,8*R*,9*R*,10*S*,11*S*,13*S*,14*R*,17*R*,18*R*,19*S*,20*R*)-3β,11α-dihydroxy-12-oxo-13α,27-cycloursan-23-oic acid, 5]

White powder; [*α*]^22^_D_ +34.7 (*c* 0.15, MeOH); UV (MeOH) *λ*_max_ (log *ε*) 217 (2.49) nm; ECD (*c* 3.20 × 10^−3^ M, MeOH) *λ*_max_ (Δ*ε*): 233 (−2.4), 291 (+3.8); IR (KBr) *v*_max_ 3436, 2985, 2915, 2860, 1694, 1659, 1619, 1452, 1384, 1212, 1058, 1013, 866 cm^−1^; ^1^H and ^13^C NMR data, see Tables 1 and 3; ESIMS *m*/*z* 509 [M + Na]^+^, 485 [M − H]^−^, 971 [2M − H]^−^; HRESIMS *m*/*z* 509.3238 [M + Na]^+^ (calcd for C_30_H_46_O_5_Na, 509.3237, *Δ* = 0.1 ppm).

### Stewartiacid F [(3*S*,4*S*,5*R*,8*R*,9*R*,10*S*,11*S*,14*S*,17*R*,18*R*,19*S*,20*R*,22*S*)-11α-methoxy-3β,12,22α-trihydroxy-urs-12-en-23-oic acid, 6]

Colorless crystals (MeOH), mp 220.9–221.5; [*α*]^22^_D_ +23.3 (*c* 0.3, MeOH); UV (MeOH) *λ*_max_ (log *ε*) 209 (2.02) nm; ECD (*c* 2.7 × 10^−3^ M, MeOH) *λ*_max_ (Δ*ε*): 207 (+7.2); IR (KBr) *v*_max_ 3405, 2993, 2925, 2860, 2823, 1701, 1681, 1454, 1344, 1314, 1202, 1054, 1032, 1014, 721 cm^−1^; ^1^H and ^13^C NMR data, see Tables 1 and 3; ESIMS *m*/*z* 517 [M − H]^−^; HRESIMS *m*/*z* 517.3539 [M − H]^−^ (calcd for C_30_H_49_O_6_, 517.3535, *Δ* = 0.9 ppm).

### Stewartiacid G [(3*S*,4*S*,5*R*,8*R*,9*R*,10*S*,11*S*,14*S*,17*R*,18*R*,19*S*,20*R*)-11α-methoxy-3β,12-dihydroxy-urs-12-en-23-oic acid, 7]

White powder; [*α*]^22^_D_ +17.3 (*c* 0.2, MeOH); UV (MeOH) *λ*_max_ (log *ε*) 203 (1.10) nm; ECD (*c* 4.3 × 10^−3^ M, MeOH) *λ*_max_ (Δ*ε*): 206 (+17.4); IR (KBr) *v*_max_ 3681, 2920, 2841, 1701, 1681, 1642, 1522, 1457, 1053, 1036, 874, 809, 724 cm^−1^; ^1^H and ^13^C NMR data, see Tables 1 and 3; ESIMS *m*/*z* 517 [M − H]^−^; HRESIMS *m*/*z* 501.3577 [M − H]^−^ (calcd for C_31_H_49_O_5_, 501.3585, *Δ* = −1.6 ppm).

### Stewartiacid H [(3*S*,4*S*,5*R*,8*R*,9*R*,10*S*,14*S*,17*R*,18*R*,19*S*,20*R*,22*S*)-3β,12,22α-trihydroxy-11-oxo-urs-12-en-23-oic acid, 8]

White powder; [*α*]^22^_D_ +10.6 (*c* 0.2, MeOH); UV (MeOH) *λ*_max_ (log *ε*) 287 (2.67) nm; ECD (*c* 4.1 × 10^−3^ M, MeOH) *λ*_max_ (Δ*ε*): 209 (+2.6), 234 (−0.5), 285 (+4.2); IR (KBr) *v*_max_ 3663, 2970, 2868, 1702, 1671, 1452, 1347, 1205, 1058, 1005, 729 cm^−1^; ^1^H and ^13^C NMR data, see Tables 1 and 3; ESIMS *m*/*z* 501 [M − H]^−^; HRESIMS *m*/*z* 501.3225 [M − H]^−^ (calcd for C_30_H_45_O_6_, 501.3222, *Δ* = 0.7 ppm).

### Stewartiacid I [(3*S*,4*S*,5*R*,8*R*,9*R*,10*S*,14*S*,17*R*,18*R*,19*S*,20*R*)-3β,12-dihydroxy-11,22-dioxo-urs-12-en-23-oic acid, 9]

White powder; [*α*]^22^_D_ +5.8 (*c* 0.2, MeOH); UV (MeOH) *λ*_max_ (log *ε*) 285 (1.97) nm; ECD (*c* 4.8 × 10^−3^ M, MeOH) *λ*_max_ (Δ*ε*): 214 (+3.0), 236 (−0.7), 266 (+2.3), 300 (+10.1); IR (KBr) *v*_max_ 3463, 2970, 2910, 2830, 1703, 1661, 1449, 1357, 1182, 1063, 1010, 871 cm^−1^; ^1^H and ^13^C NMR data, see Tables 1 and 4; ESIMS *m*/*z* 499 [M − H]^−^; HRESIMS *m*/*z* 499.3065 [M − H]^−^ (calcd for C_30_H_43_O_6_, 499.3065, *Δ* = 0.7 ppm).

**Table 4:** ^1^H NMR data^a^ (*δ* in ppm, *J* in Hz, 400 MHz) of 9 and 10 in CD_3_OD

No.	9	10
1α	1.23, m	1.17, m
1β	2.79, br d (12.5)	2.72, br d (12.1)
2a	1.67, m	1.72, br dd (12.9, 12.7)
2b	1.63, m	1.64, m
3	3.99, dd (11.4, 4.9)	3.98, dd (11.2, 3.9)
5	1.56, br d (11.6)	1.53, br d (overlapped)
6α	1.17, m	1.14, m
6β	1.64, m	1.65, m
7α	1.44, m	1.55, m
7β	1.76, m	1.70, m
9	2.62, s	2.52, s
15α	1.25, m	1.33, br d (13.6)
15β	1.72, m	2.09, ddd (13.7, 13.6, 4.1)
16α	2.20, ddd (14.4, 14.2, 4.5)	1.54, m
16β	1.93, m	1.98, br d (13.6)
18	2.92, d (11.9)	
19	1.89, m	
20	1.56, m	2.65, m
21α	2.47, dd (14.7, 12.4)	3.11, dd (12.1, 6.0)
21β	2.38, dd (14.7, 3.6)	2.12, br d (12.1)
24	1.12, s	1.12, s
25	1.19, s	1.26, s
26	1.16, s	1.23, s
27	1.46, s	1.16, s
28	0.98, s	1.12, s
29	0.94, d (6.0)	1.59, s
30	1.04, d (6.2)	1.02, d (7.0)

a Assignments were made by a combination of 1D and 2D NMR experiments.

### Stewartiacid J [(3S*,4S*,5R*,8R*,9R*,10S*,14S*,17R*,20R*)-(3β,12-dihydroxy-11,22-dioxo-urs-12,18-dien-23-oic acid, 10]

White powder; [*α*]^22^_D_ +18.0 (*c* 0.05, MeOH); UV (MeOH) *λ*_max_ (log *ε*) 299 (2.70) nm; IR (KBr) *v*_max_ 3449, 2975, 2915, 2866, 1700, 1656, 1474, 1384, 1210, 1055, 1030, 803 cm^−1^; ^1^H and ^13^C NMR data, see Tables 1 and 4; ESIMS *m*/*z* 497 [M − H]^−^; HRESIMS *m*/*z* 497.2933 [M − H]^−^ (calcd for C_30_H_41_O_6_, 497.2909, *Δ* = 4.9 ppm).

### Stewartiacid K [(3S*,4S*,5R*,8R*,9R*,10S*,11S*,14S*,17R*, 18R*,19S*,20R*,7′R*,8′R*)*-*11α,12-[2-(hydroxymethyl)-3-(4-hydroxy-3-methoxyphenyl) ethane-1,2-dioxy]-3β-acetoxy-urs-12-en-23-oic acid, 11]

White powder; [*α*]^22^_D_ +25.0 (*c* 0.2, MeOH); UV (MeOH) *λ*_max_ (log *ε*) 203 (7.94), 279 (0.65) nm; ECD (*c* 7.06 × 10^−3^ M, MeOH) *λ*_max_ (Δ*ε*): 228 (+0.4), 278 (+0.2); IR (KBr) *v*_max_ 3439, 2980, 2925, 2860, 1715, 1671, 1619, 1454, 1384, 1267, 1055, 1010, 918 cm^−1^; ^1^H and ^13^C NMR data, see Table 5; ESIMS *m*/*z* 731 [M + Na]^+^; HRESIMS *m*/*z* 731.4111 [M + Na]^+^ (calcd for C_42_H_60_O_9_Na, 731.4130, *Δ* = −2.6 ppm).

**Table 5:** ^1^H and ^13^C NMR data^a^ (*δ* in ppm) of 11 and 12 in C_5_D_5_N

No.	11		12	
	*δ*_H_ (*J* in Hz)^b^	*δ*_C_ c	*δ*_H_ (*J* in Hz)^b^	*δ*_C_ c
1α	1.60, m	41.1	1.55, m	41.3
1β	2.70 br d (11.7)		2.75, br d (13.2)	
2	1.69, m; 1.78, m	23.7	1.74, m; 1.99, m	24.2
3	5.75, dd (12.0, 5.6)	78.0	5.82, dd (12.1, 4.5)	78.1
4		52.4		52.6
5	2.09, br d (11.3)	51.7	2.05, br d (10.0)	51.7
6	1.60, m; 1.78, m	21.3	1.58, m; 1.75, m	21.3
7	1.27, m; 1.73, m	33.9	1.25, m; 1.70, m	34.4
8		42.8		41.5
9	2.15, d (8.8)	52.5	2.10, d (10.4)	50.8
10		38.1		37.8
11	5.36, d (8.8)	68.1	4.77, d (10.4)	67.7
12		146.6		145.6
13		113.9		121.6
14		41.0		43.2
15	0.92, m; 1.75, m	27.3	0.92, m; 1.73, m	27.2
16	0.79, m; 2.02, m	27.9	0.77, m; 2.00, m	27.8
17		33.3		33.6
18	2.68, d (10.7)	46.0	2.86, d (11.1)	46.6
19	1.50, m	41.3	1.47, m	39.8
20	0.76, m	39.5	1.01, m	39.7
21	1.25, m; 1.31, m	31.6	1.28, m; 1.45, m	31.5
22	1.28, m; 1.41, m	41.9	1.29, m; 1.40, m	41.3
23		179.2		179.2
24	1.62, s	12.9	1.52, s	12.8
25	1.45, s	17.2	0.80, s	16.2
26	1.20, s	18.2	1.14, s	18.6
27	1.34, s	24.2	1.32, s	23.9
28	0.91, s	28.9	0.88, s	28.6
29	1.32, d (6.2)	17.1	1.25, d (6.5)	18.0
30	0.88, d (6.2)	21.0	0.91, d (6.4)	21.0
1′		132.1		128.0
2′	7.37, br s	112.0	7.49, br s	115.0
3′		148.6		148.2
4′		148.2		148.1
5′	7.28, d (7.9)	116.4	7.26, d (7.8)	115.8
6′	7.23, br d (7.9)	121.1	7.48, br d (7.8)	123.5
7′	5.22, d (10.4)	77.9	5.20, d (3.7)	75.2
8′	4.99, dd (10.5, 3.1)	77.6	4.88, ddd (7.1, 5.5, 3.7)	81.2
9′	3.88, dd (11.9, 4.6)	62.2	4.03, dd (12.0, 5.5)	62.7
	4.04, br d (11.9)		4.29, dd (12.0, 7.1)	
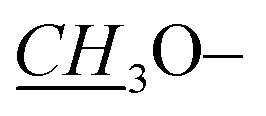	3.86, s	55.9	3.83, s	55.9
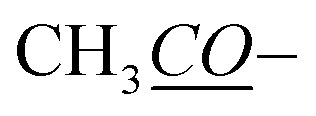		169.9		170.0
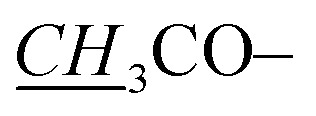	1.91, s	21.3	1.91, s	21.2

a Assignments were made by a combination of 1D and 2D NMR experiments.

b measured at 400 MHz.

c measured at 150 MHz.

### Stewartiacid L [(3*S*,4*S*,5*R*,8*R*,9*R*,10*S*,11*S*,14*S*,17*R*,18*R*,19*S*,20*R*,7′*S*,8′*R*)*-*11α,12-[3-(hydroxymethyl)-2-(4-hydroxy-3-methoxyphenyl)ethane-1,2-dioxy]-3β-acetoxy-urs-12-en-23-oic acid, 12]

White powder; [*α*]^22^_D_ +16.7 (*c* 0.1, MeOH); UV (MeOH) *λ*_max_ (log *ε*) 203 (7.30), 279 (0.72) nm; ECD (*c* 2.61 × 10^−3^ M, MeOH) *λ*_max_ (Δ*ε*): 214 (+8.6), 279 (+0.3); IR (KBr) *v*_max_ 3441, 2927, 2853, 1713, 1661, 1619, 1439, 1380, 1207, 1080, 1020, 941 cm^−1^; ^1^H and ^13^C NMR data, see Table 5; ESIMS *m*/*z* 731 [M + Na]^+^; HRESIMS *m*/*z* 731.4125 [M + Na]^+^ (calcd for C_42_H_60_O_9_Na, 731.4130, *Δ* = −0.6 ppm).

### Stewartiacid M [(3S*,4S*,5R*,8R*,9R*,10S*,14S*,17R*,18R*)-3β,12-dihydroxy-11,22-dioxo-olean-12-en-23-oic acid, 13]

White powder; [*α*]^22^_D_ +4.2 (*c* 0.05, MeOH); UV (MeOH) *λ*_max_ (log *ε*) 285 (2.02) nm; IR (KBr) *v*_max_ 3446, 2977, 2922, 2873, 1704, 1661, 1454, 1377, 1207, 1140, 1060, 1033, 1008, 948, 843, 721 cm^−1^; ^1^H and ^13^C NMR data, see Tables 1 and 6; ESIMS *m*/*z* 499 [M − H]^−^; HRESIMS *m*/*z* 499.3061 [M − H]^−^ (calcd for C_30_H_43_O_6_, 499.3065, *Δ* = −0.9 ppm).

**Table 6:** ^1^H NMR data^a^ (*δ* in ppm, *J* in Hz, 400 MHz) of 13 and 14

No.	13^b^	14^c^
1α	1.18, m	1.60, m
1β	2.85, br d (13.1)	3.24, br d (13.2)
2a	1.71, m	2.02, m
2b	1.65, m	2.08, m
3	3.98, dd (11.0, 4.4)	4.73, br d (9.0)
5	1.54, br d (11.7)	2.10, br d (12.5)
6α	1.14, m	1.63, m
6β	1.63, m	2.00, m
7α	1.44, m	1.62, m
7β	1.72, m	1.28, m
9	2.63, s	2.00, d (12.1)
11		5.52, d (12.1)
15α	1.21, m	1.08, br d (13.8)
15β	1.84, m	2.26, ddd (13.8, 13.5, 3.0)
16α	2.19, ddd (13.5, 13.3, 3.0)	1.99, m
16β	1.84, m	1.83, br d (15.4)
18	3.19, dd (12.2, 3.7)	2.68, br d (12.3)
19α	1.93, dd (13.4, 12.2)	1.22, dd (13.4, 13.1)
19β	1.28, m	2.34, br d (13.4)
21α	2.61, br d (14.1)	2.48, d (12.4)
21β	2.05, dd (14.1, 2.4)	1.92, d (12.4)
24	1.11, s	1.75, s
25	1.17, s	1.39, s
26	1.15, s	1.67, s
27	1.49, s	0.94, s
28	0.99, s	1.65, s
29	1.03, s	0.88, s
30	0.90, s	1.10, s
13-OH		7.13, s

a Assignments were made by a combination of 1D and 2D NMR experiments.

b Measured in CD_3_OD.

c Measured in C_5_D_5_N.

**Table 7:** Inhibitory activities of indicated compounds against ACL and NF-κB

Compound	IC_50_^a^ (μM)	
	ACL	NF-κB
7	12.5 ± 5.1	>50
11	2.8 ± 0.9	16.8 ± 1.1
12	10.6 ± 0.1	>50
BMS 303141^b^	0.4 ± 0.1	NT^d^
PS-341^c^	NT^d^	0.06 ± 0.01

a These data are expressed as the mean ± SEM of triplicated experiments.

b Positive control for the ACL assay.

c Positive control for the NF-κB assay.

d NT: not tested.

### Stewartiacid N [(3S*,4S*,5R*,8R*,9R*,10S*,13S*,14S*,17R*, 18R*)-3β,11α,13β-trihydroxy-11,22-dioxo-olean-23-oic acid, 14]

White powder; [*α*]^22^_D_ +22.0 (*c* 0.2, MeOH) {camellisin C:^28^ [*α*]^24^_D_ +10.0 (*c* 0.1, C_5_H_5_N)}; UV (MeOH) *λ*_max_ (log *ε*) 210 (0.66) nm; IR (KBr) *v*_max_ 3446, 2977, 2922, 2873, 1703, 1661, 1454, 1377, 1207, 1060, 1033, 843 cm^−1^; ^1^H and ^13^C NMR data, see Tables 1 and 6; ESIMS *m*/*z* 517 [M − H]^−^, 541 [M + Na]^+^; HRESIMS *m*/*z* 541.3140 [M + Na]^+^ (calcd for C_30_H_46_O_7_Na, 541.3136, *Δ* = 0.9 ppm).

### X-ray crystallographic data of stewartiacids A (1), C (3), D (4), and F (6)

Detailed data are included in the ESI.‡ The structures were solved with the SheIXT^33^ structure solution program using Intrinsic Phasing and refined with the SheIXT^34^ refinement using Least Squares minimization. Crystallographic data of compounds 1, 3, 4, and 6 have been deposited in the Cambridge Crystallographic Data Centre as CCDC-1959096, CCDC-1972126, CCDC-1959098, and CCDC-1959097, respectively.

### ECD calculations of stewartiacids H (8), I (9), and L (12)

The Monte Carlo conformational searches were carried out by Spartan's 10 software (Wavefunction, Inc., Irvine, CA.) using Merck Molecular Force Field (MMFF). The conformers with Boltzmann-population of over 7% were chosen for ECD calculations, and then the conformers were initially optimized at B3LYP/6-31g (d,p) level in MeOH using the conductor-like polarizable calculation model (CPCM). The theoretical calculation of ECD was conducted in MeOH using time-dependent density functional theory (TDDFT) at the CAM-B3LYP/def2-TZVP level^35^ for all conformers of stewartiacids H (8), I (9), and L (12). Rotatory strengths for a total of 30 excited states were calculated. ECD spectra were generated using the program SpecDis 1.6 (University of Würzbrg, Würzburg, Germany) and GraphPad Prism 5 (University of California San Diego, USA) from dipole-length rotational strengths by applying Gaussian band shapes with sigma = 0.3 eV.

### ATP-citrate lyase inhibitory assay

The assay was performed using ADP-Glo™ luminescence assay reagents. It measures ACL activity by quantification of the amount of ADP generated by the enzymatic reaction. The luminescent signal from the assay is correlated with the amount of ADP generated and is proportionally correlated with the amount of ACL activity.^30^ The kinase assay was carried out in a 384-well plate (ProxiPlateTM-384 Plus, PerkinElmer) in a volume of 5 μL reaction mixture containing 2.0 μL of ACL, 2.0 μL of ATP, and 1.0 μL of the tested compound with different concentrations. Reactions in each well were kept going for 30 min under 37 °C. After the enzymatic reaction, 2.5 μL of ADP-Glo™ reagent was added to each well to terminate the kinase reaction and deplete the unconsumed ATP within 60 min at room temperature. In the end, 5.0 μL of kinase detection reagent (reagent 2) was added to each well and incubated for 1 h to simultaneously convert ADP to ATP. The luminescence signal was measured using a PerkinElmer EnVision reader. The known inhibitor BMS 303141 (ref. 30) (CAS no. 943962-47-8) was used as the positive control.

### NF-κB inhibitory assay

The HEK293 with stable NF-κB expression cell line was used for the luciferase assay.^36^ Cells were seeded into 96-well plates and incubated for 24 h, and then treated with different concentrations of the tested compound followed by stimulation with 20 ng mL^−1^ TNF-α. The luciferase substrate was added to each well after incubation for 6 h, and then the released luciferin signal was detected using an EnVision microplate reader. The IC_50_ value was derived from a nonlinear regression model (curve-fit) based on a sigmoidal dose–response curve (variable slope) and computed using Graphpad Prism 5 (Graphpad Software). Bortezomib (PS-341, CAS no. 179324-69-7) was used as the positive control.^31^

## Conflicts of interest

There are no conflicts to declare.

## Supplementary Material

RA-010-C9RA09542J-s001

RA-010-C9RA09542J-s002
