# Supplementary material for: Stewartiacids A–N, C-23 carboxylated triterpenoids from Chinese Stewartia and their inhibitory effects against ATP-citrate lyase and NF-κB
Source: RSC Adv. 2020 Jan 21;10(6):3343–56. doi: 10.1039/c9ra09542j (PMC9048753; doi:10.1039/c9ra09542j)
Supplement: RA-010-C9RA09542J-s001 [file RA-010-C9RA09542J-s001.pdf]

Electronic Supplementary Material (ESI) for RSC Advances.
This journal is © The Royal Society of Chemistry 2020

Supporting Information for

Stewartiacids A–N, C-23 carboxylated triterpenoids from the Chinese Stewartia and their inhibitory effects against ATP-citrate lyase and NF-*κ*B

Jiang Wan,[a,b] Yi Zang,[c] Dao-An Xiao,[d] Na Li,[a] JunminLi,[a] Ze-Xin Jin,[a] De-Lei Chen,[e] Juan Xiong,*[b] Jia Li,*[c] and Jin-Feng Hu*[a,b]

[a] Institute of Natural Medicine and Health Products, School of Advanced Study, Zhejiang Provincial Key Laboratory of Plant Ecology and Conservation, Taizhou University, Taizhou 318000, Zhejiang, PR China.

[b] School of Pharmacy, Fudan University, No. 826 Zhangheng Road, Shanghai 201203, PR China. E-mail: jfhu@fudan.edu.cn; Telephone/Fax: +86-21-51980172.

[c] State Key Laboratory of Drug Research, Shanghai Institute of MateriaMedica, Chinese Academy of Sciences, Shanghai 201203, PR China.

[d] College of Chemistry and Bioengineer, Yichun University, Yichun 336000, PR China.

[e] School of Life Science, Hefei Normal University, Hefei 230601, PR China.

---

* Corresponding authors. Tel/fax: +86 21 51980172.

E-mail addresses: jxiong@fudan.edu.cn (J. Xiong); jli@simm.ac.cn (J Li); jfhu@fudan.edu.cn (J.-F. Hu).

Contents	page
Fig. S1. Key HMBC correlation of compounds **2**, **5**, **7**, **9**, **10**, and **14**.	1
Fig. S2. Diagnostic ROE correlations of compounds **2**, **5**, **7**–**10**, and **13**.	1
Figs. S3–S13: 1D/2D NMR and HRESIMS spectra for compound **1**	2−7
Figs. S14–S22: 1D/2D NMR and HRESIMS spectra for compound **2**	8−12
Fig. S23: ECD spectra of compounds **1** and **2**	12
Figs. S24–S31: 1D/2D NMR and HRESIMS spectra for compound **3**	13−16
Fig. S32: ECD spectrum of compound **3**	17
Figs. S33–S43: 1D/2D NMR and HRESIMS spectra for compound **4**	17−23
Figs. S44–S51: 1D/2D NMR and HRESIMS spectra for compound **5**	23−26
Fig. S52: ECD spectra of compounds **4** and **5**	27
Figs. S53–S61: 1D/2D NMR and HRESIMS spectra for compound **6**	27−31
Figs. S62–S70: 1D/2D NMR and HRESIMS spectra for compound **7**	32−36
Fig. S70: ECD spectra of compounds **6** and **7**	36
Figs. S71–S81: 1D/2D NMR and HRESIMS spectra for compound **8**	37−41
Figs. S82–S89: 1D/2D NMR and HRESIMS spectra for compound **9**	43−46
Figs. S90–S97: 1D/2D NMR and HRESIMS spectra for compound **10**	47−50
Figs. S98–S108: 1D/2D NMR and HRESIMS spectra for compound **11**	51−56
Figs. S109–S116: 1D/2D NMR and HRESIMS spectra for compound **12**	56−60
Figs. S117–S124: 1D/2D NMR and HRESIMS spectra for compound **13**	60−64
Figs. S125–S132: 1D/2D NMR and HRESIMS spectra for compound **14**	64−68
Table S1: Crystal data and structure refinement for **1**	69
Table S2: Crystal data and structure refinement for **3**	70
Table S3: Crystal data and structure refinement for **4**	71
Table S4: Crystal data and structure refinement for **6**	72

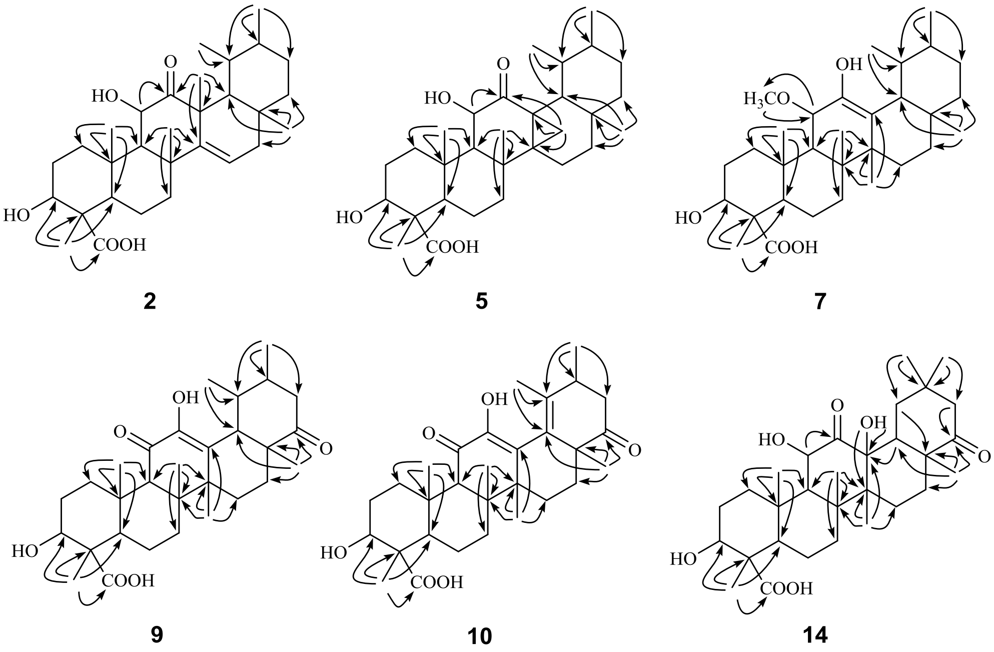

Fig. S1. HMBC correlations of compounds **2**, **5**, **7**, **9**, **10**, and **14**.

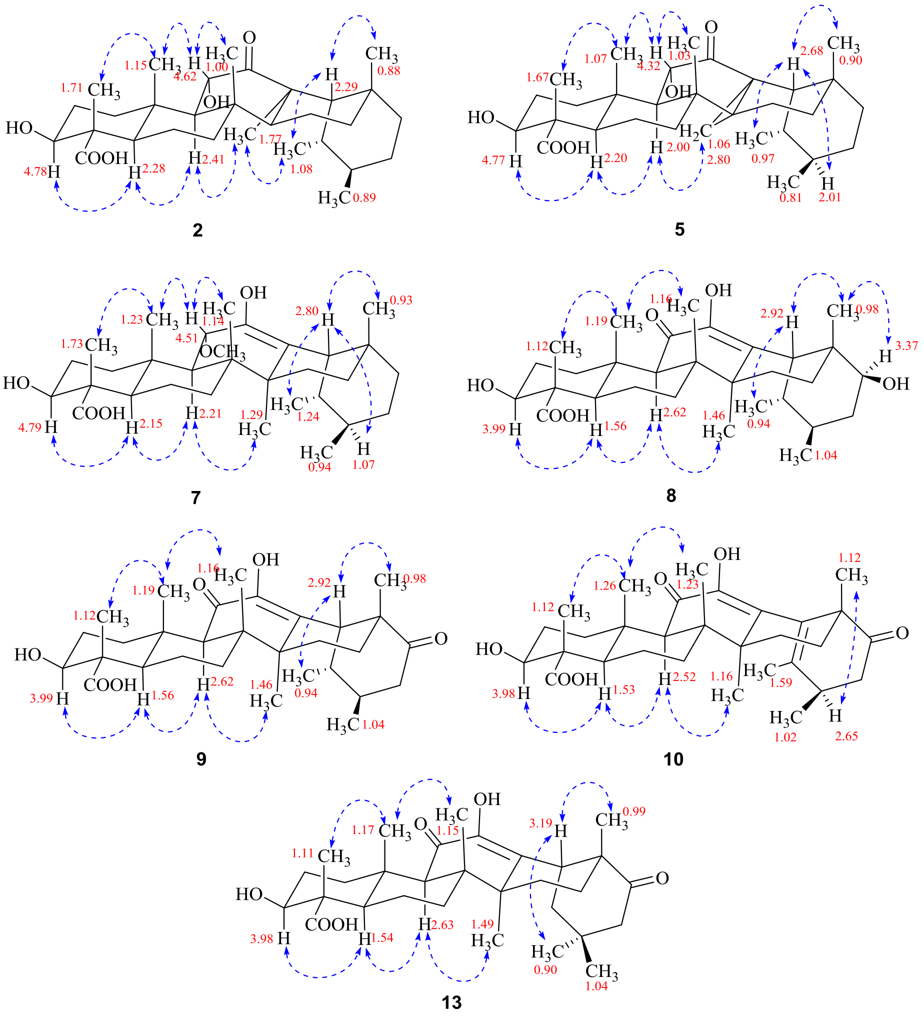

Fig.S2. Diagnostic ROE correlations of compounds **2**, **5**, **7**−**10**, and **13**.

Fig. S3. ^{1}H NMR spectrum of compound **1** in CD_3OD (400 MHz).

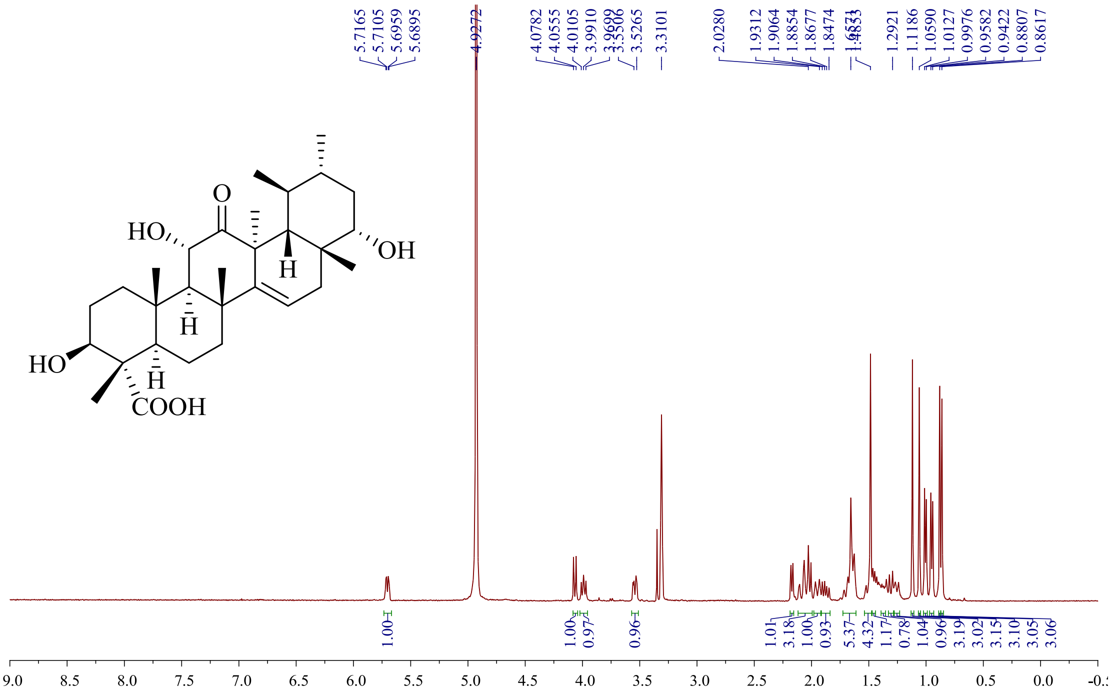

Fig. S4. ^{1}H NMR spectrum of compound **1** in CD_3OD–expansion.

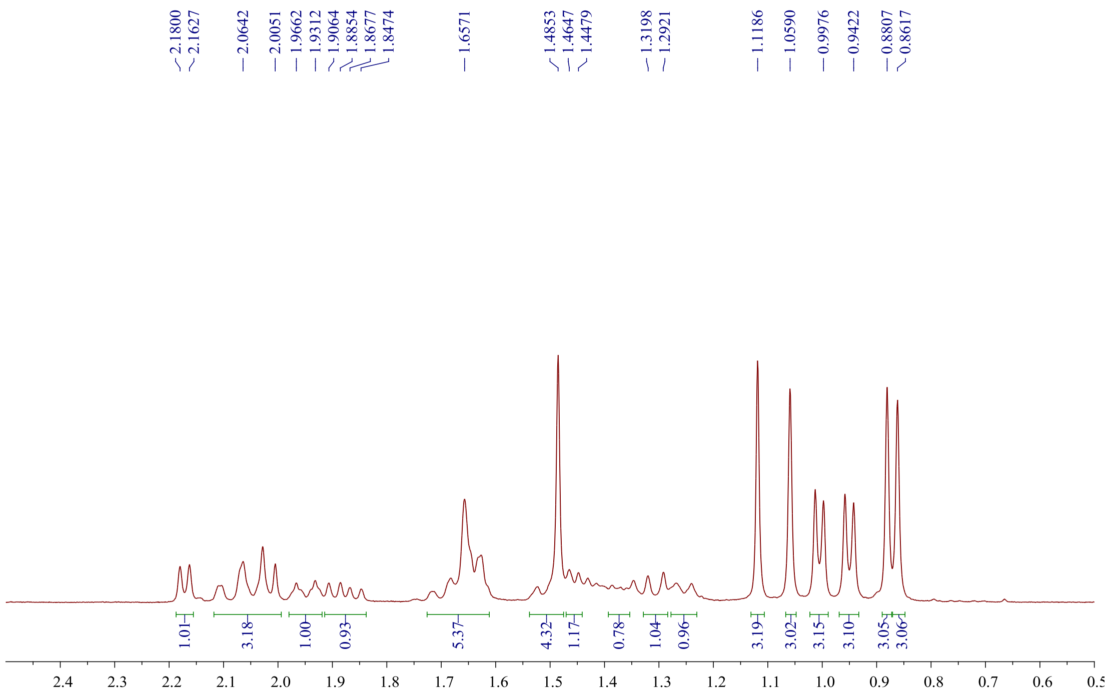

Fig. S5. ^{13}C NMR and DEPT 135 spectra of compound **1** in CD_3OD (150 MHz).

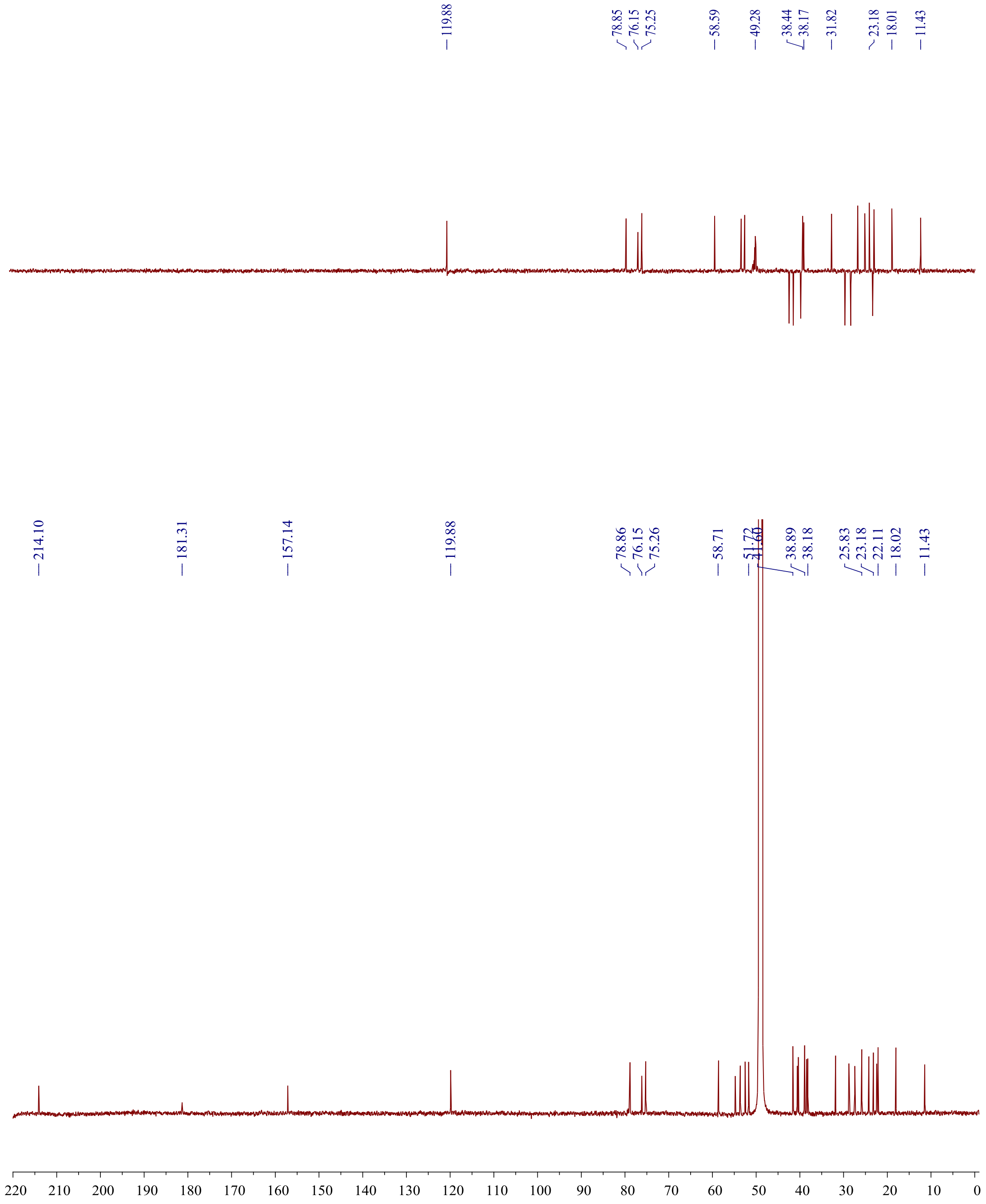

Fig. S6.^{1}H-^{1}H COSY spectrum of compound **1** in CD_3OD (600 MHz).

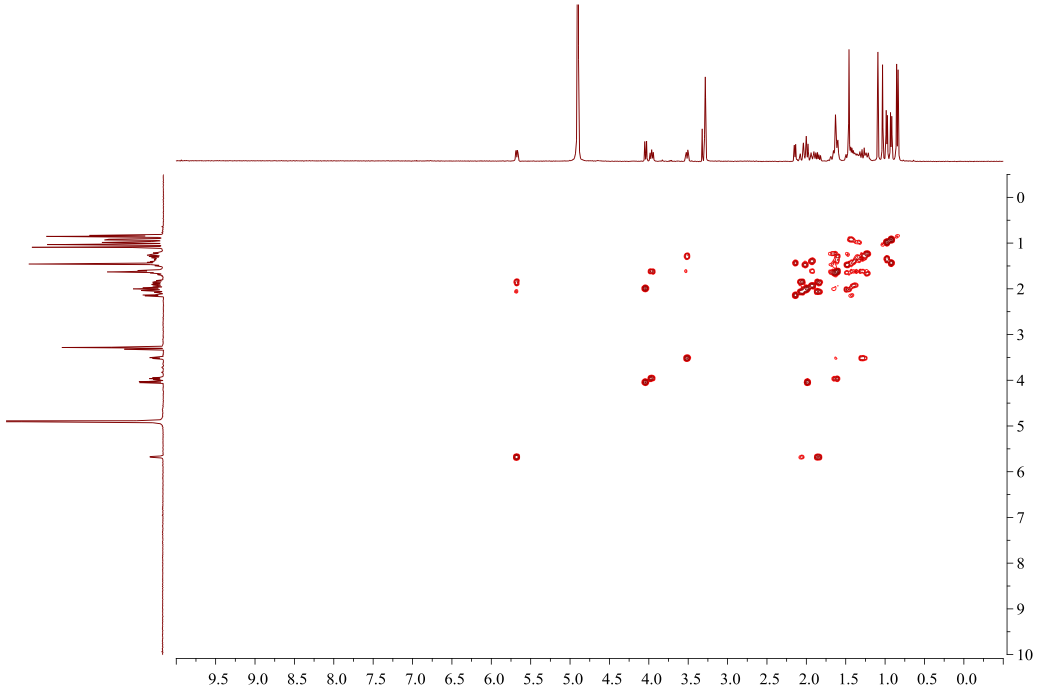

Fig. S7.^{1}H-^{1}H COSY spectrum of compound **1**-expansion.

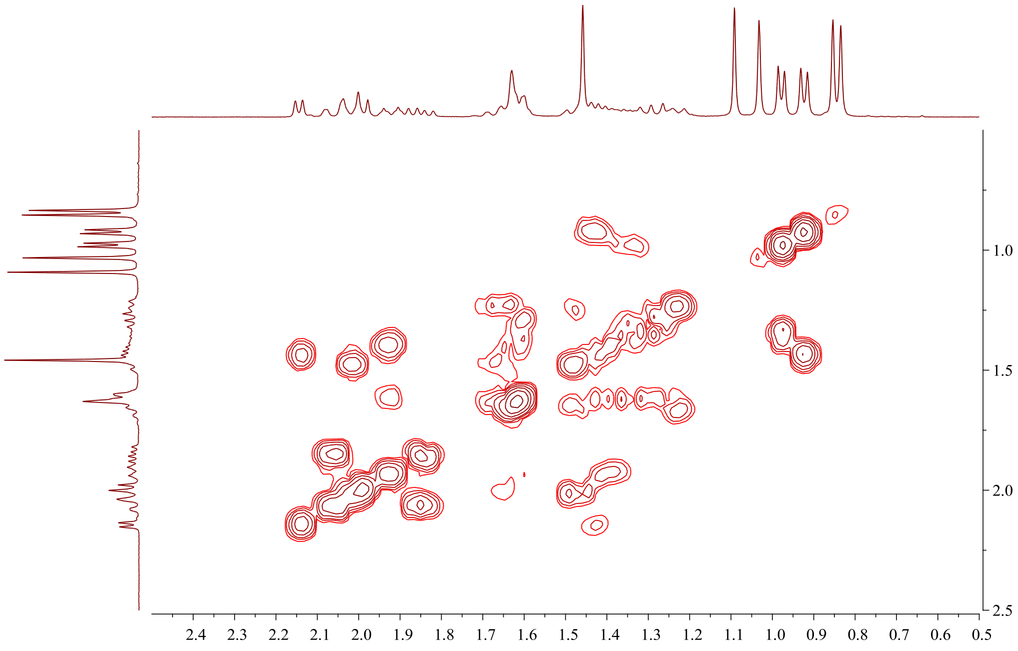

Fig. S8. HMBC spectrum of compound **1** in CD_3OD (600 MHz).

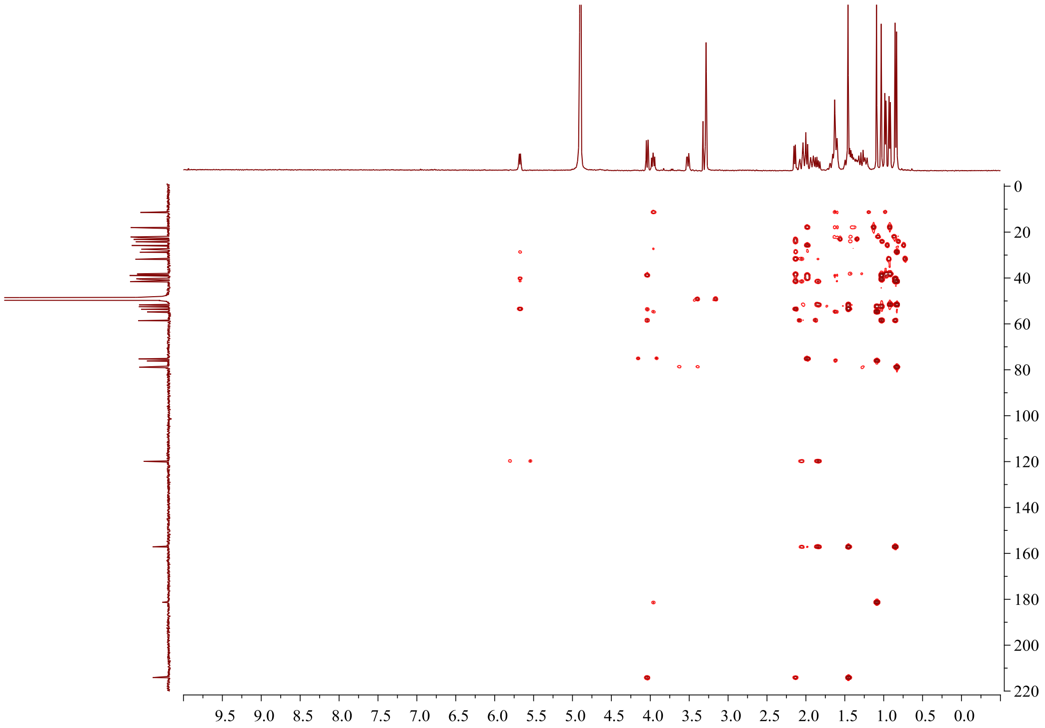

Fig. S9. HMBC spectrum of compound **1**-expansion.

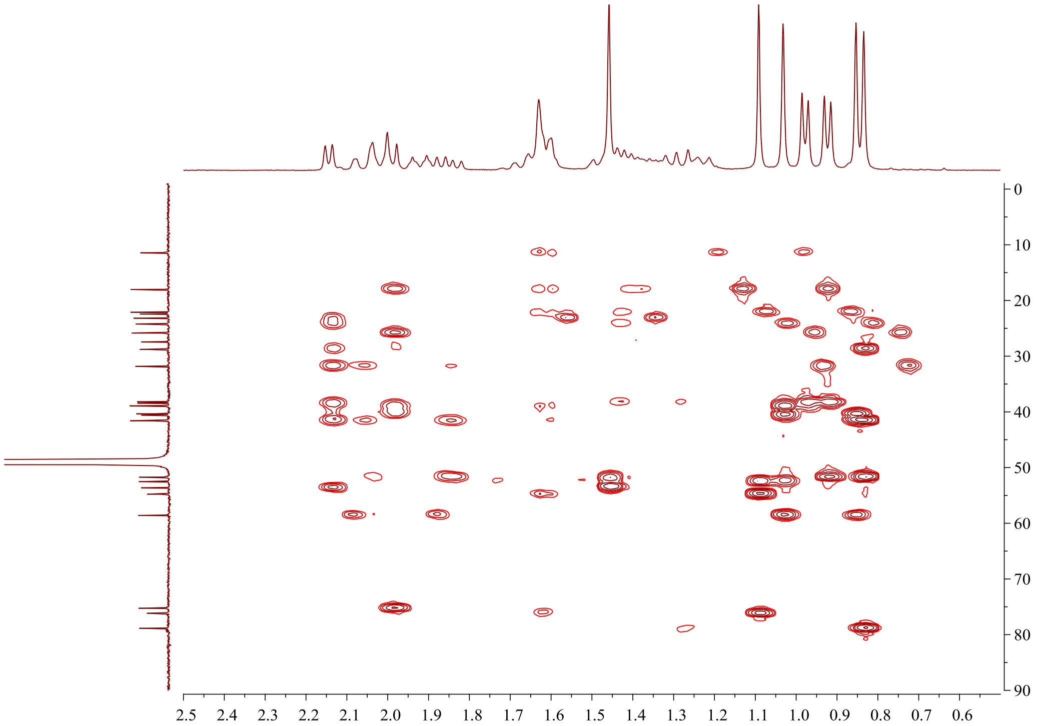

Fig. S10. ^{1}H NMR spectrum of compound **1** in C_5D_5N (600 MHz).

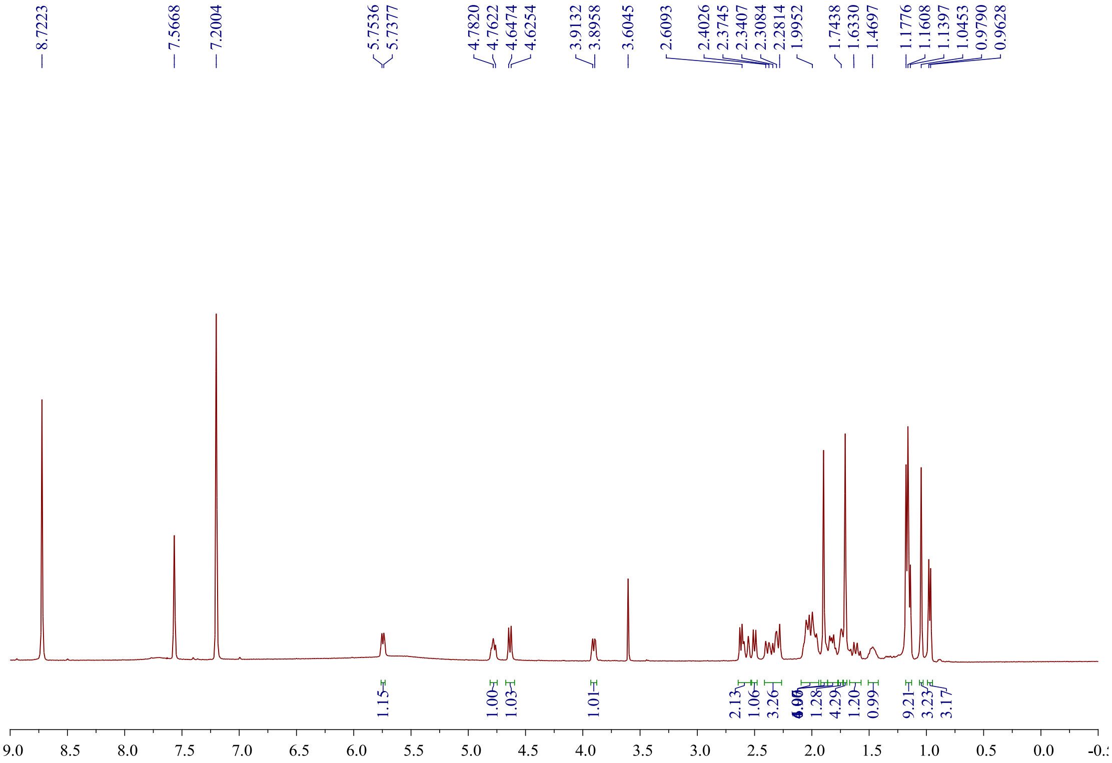

Fig. S11. ROESY spectrum of compound **1** inC_5D_5N (600 MHz).

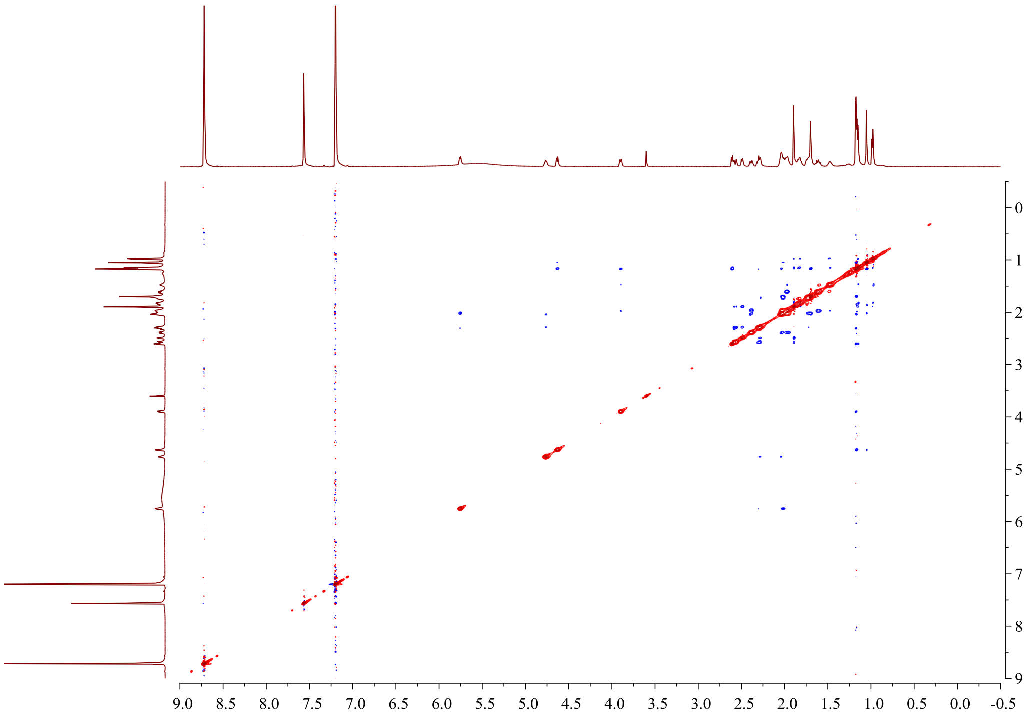

Fig. S12. ROESY spectrum of compound **1**–expansion.

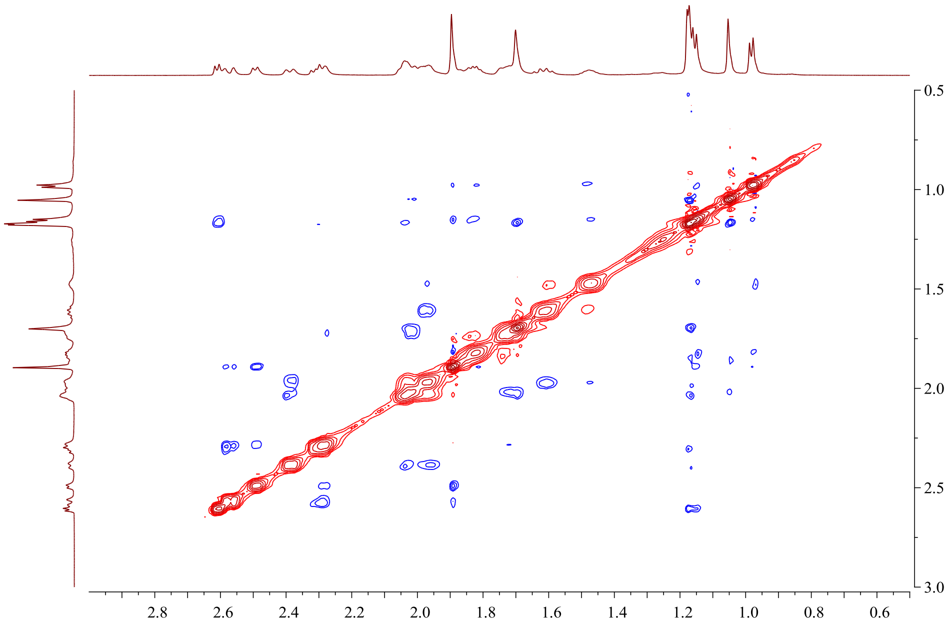

Fig. S13. HRESIMS report of compound **1**.

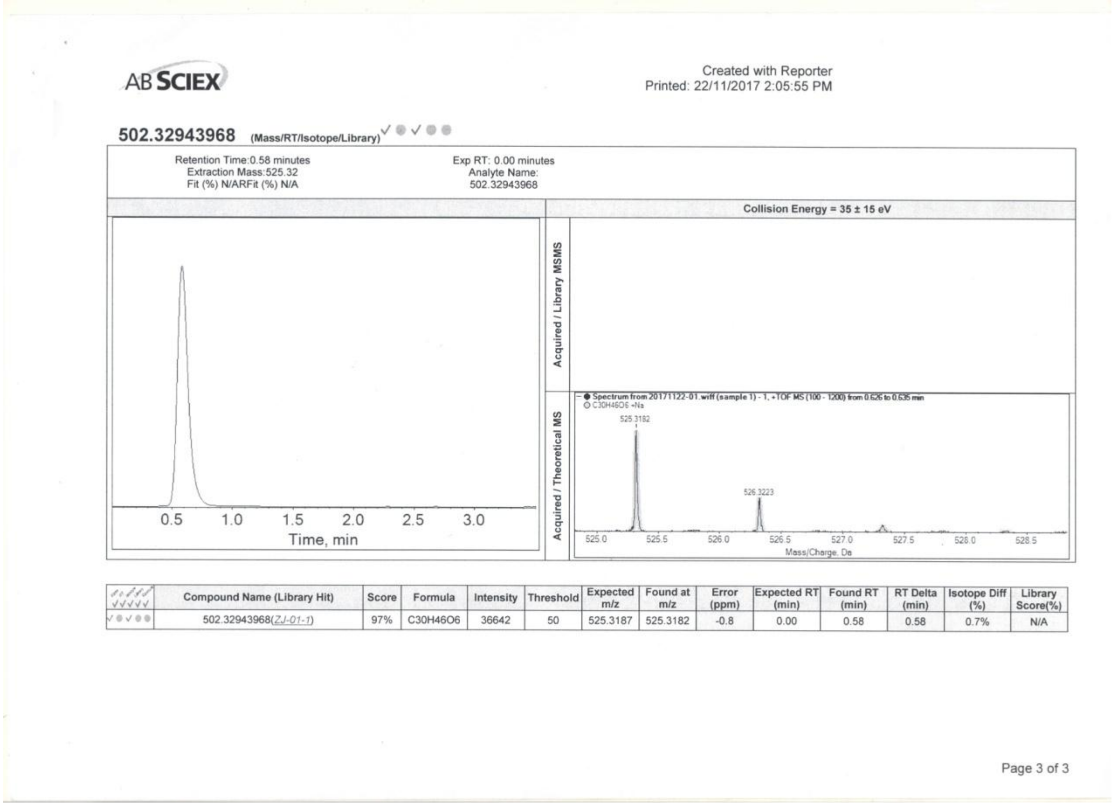

AB SCIEX

Created with Reporter
Printed: 22/11/2017 2:05:55 PM

502.32943968 (Mass/RT/Isotope/Library)

Retention Time:0.58 minutes
Extraction Mass:525.32
Fit (%) N/ARFit (%) N/A

Exp RT: 0.00 minutes
Analyte Name:
502.32943968

Collision Energy = 35 ± 15 eV

Acquired / Library MSMS

Acquired / Theoretical MS

Spectrum from 20171122-01.wiff (sample 1) - 1, +TOF MS (100 - 1200) from 0.626 to 0.635 min
C30H46O6 +Na

525.3182
526.3223

525.0 525.5 526.0 526.5 527.0 527.5 528.0 528.5
Mass/Charge, Da

0.5 1.0 1.5 2.0 2.5 3.0
Time, min

	Compound Name (Library Hit)	Score	Formula	Intensity	Threshold	Expected m/z	Found at m/z	Error (ppm)	Expected RT (min)	Found RT (min)	RT Delta (min)	Isotope Diff (%)	Library Score(%)
	502.32943968(ZJ-01-1)	97%	C30H46O6	36642	50	525.3187	525.3182	-0.8	0.00	0.58	0.58	0.7%	N/A

Page 3 of 3

Fig. S14. ^{1}H NMR spectrum of compound **2** in C_5D_5N (400 MHz).

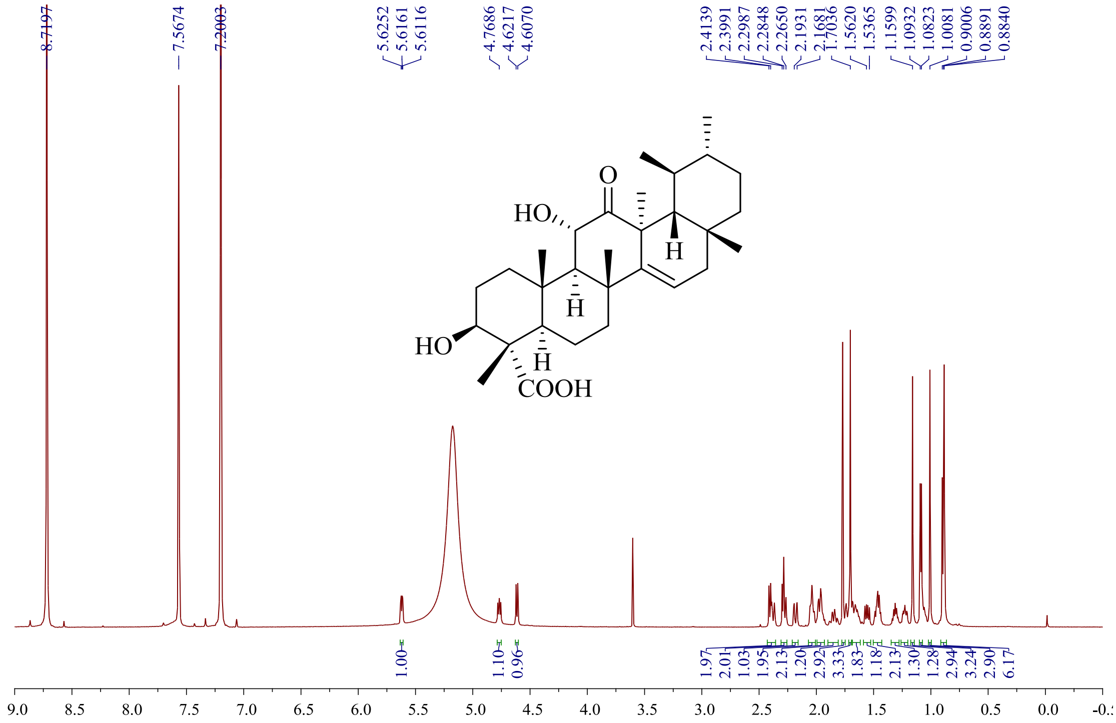

Fig. S15. ^{1}H NMR spectrum of compound **2**-expansion.

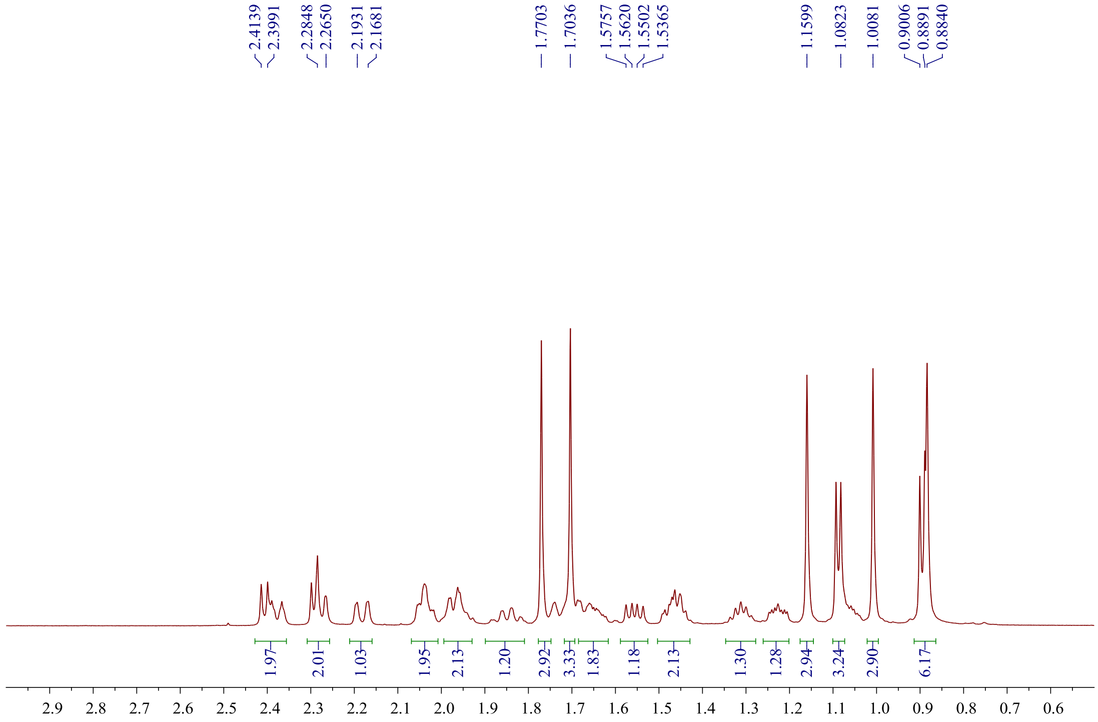

Fig. S16. ^{13}C NMR spectrum of compound **2** in C_5D_5N (150 MHz).

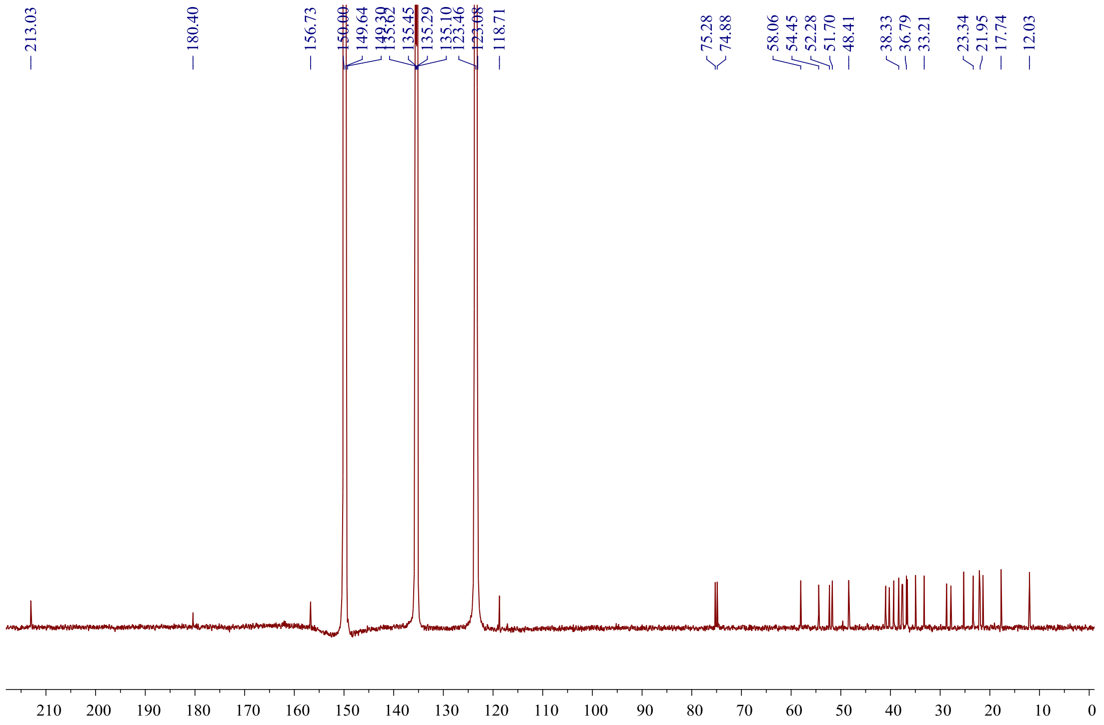

Fig. S17. ^{13}C NMR spectrum of compound **2** –expansion.

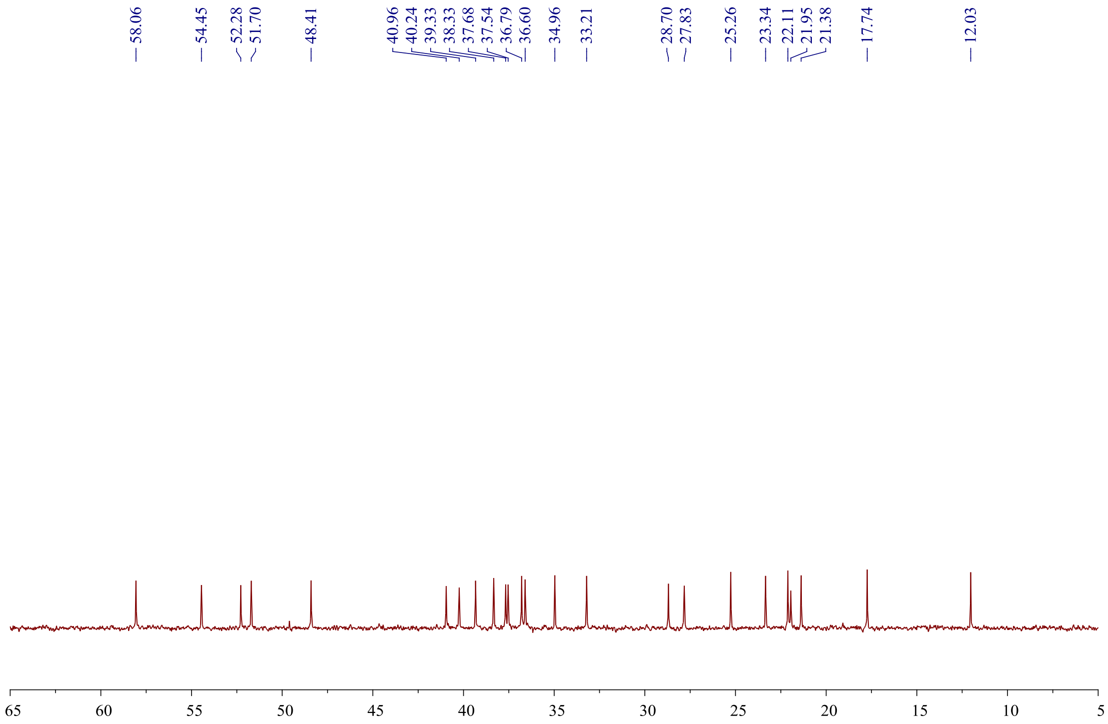

Fig. S18. HMBC spectrum of compound **2** in C_5D_5N (600 MHz).

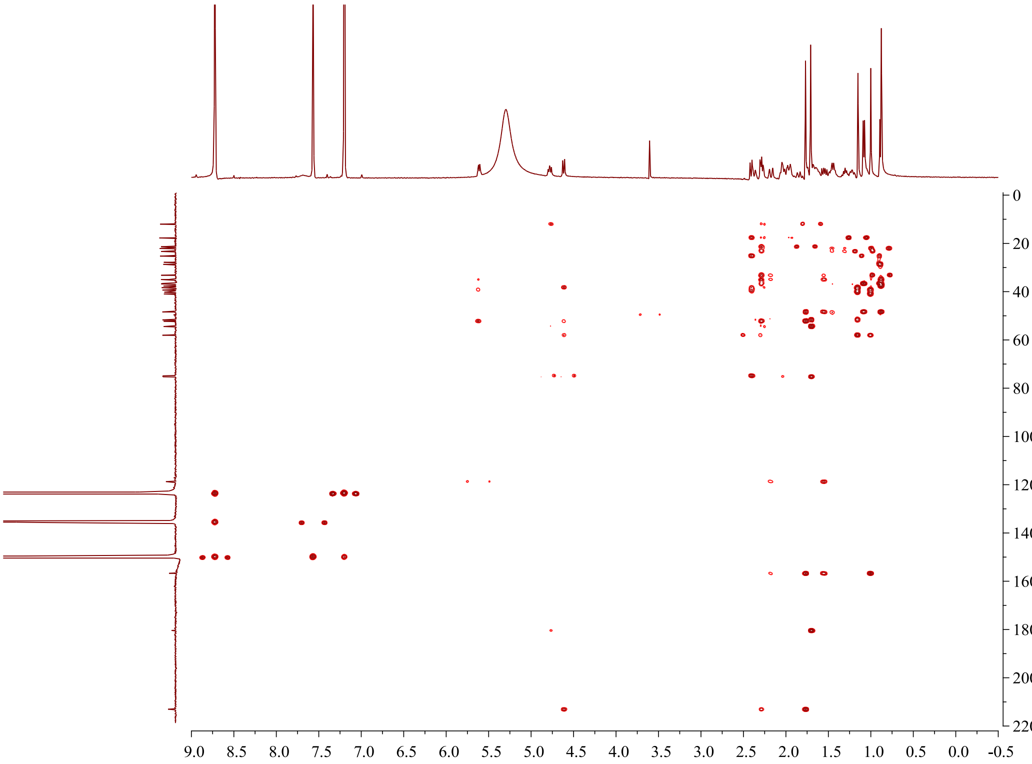

Fig. S19. HMBC spectrum of compound **2**-expansion.

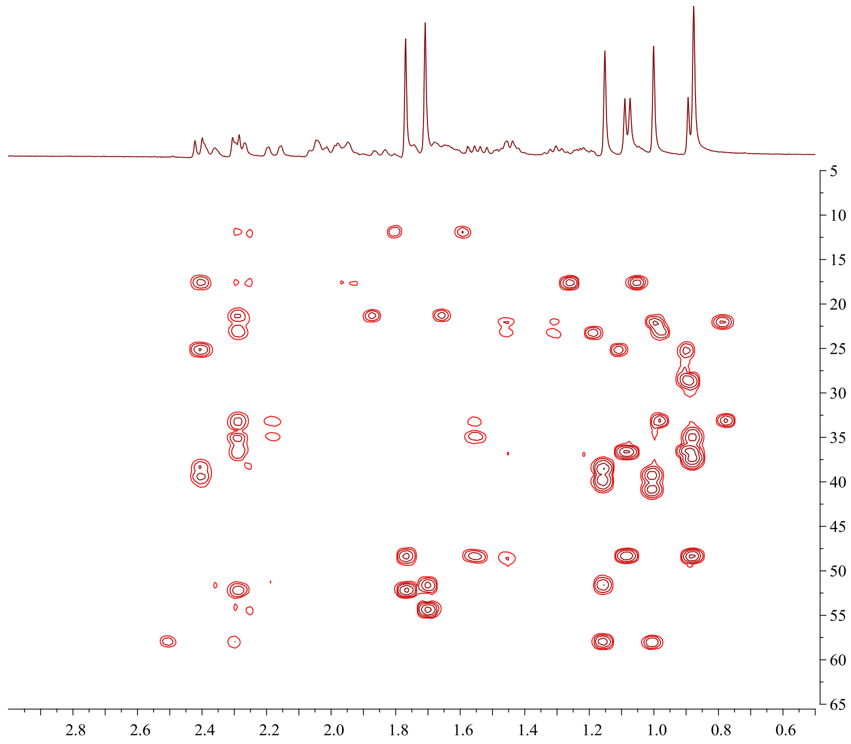

Fig. S20. ROESY spectrum of compound **2** in C_5D_5N (600 MHz).

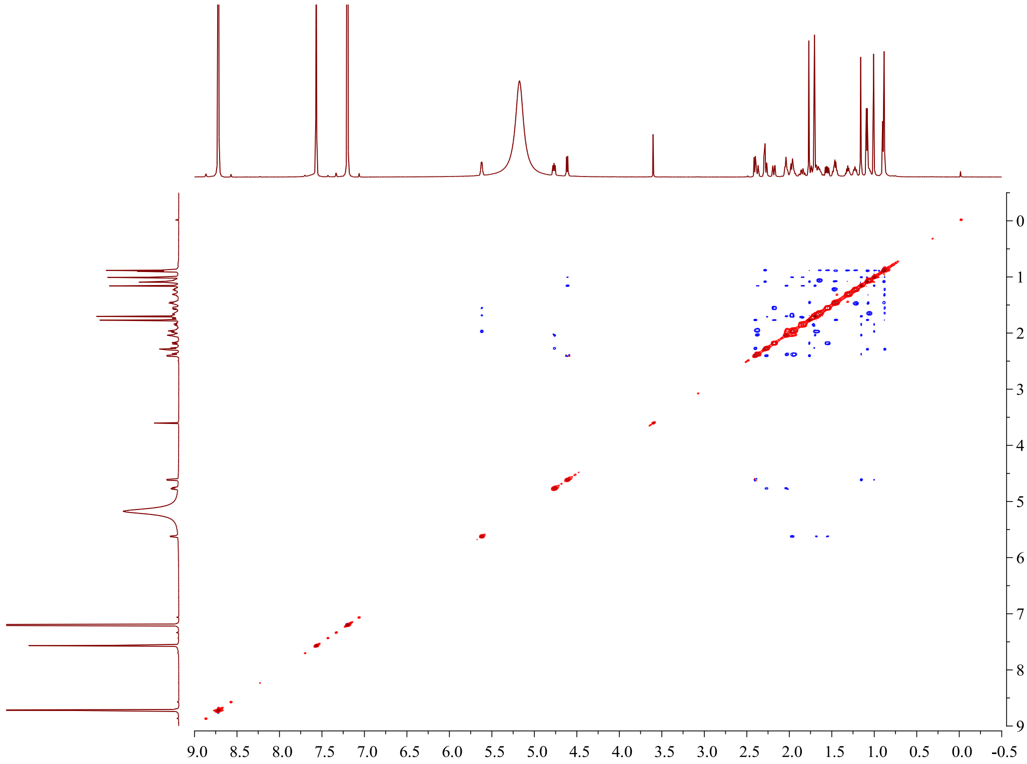

Fig. S21. ROESY spectrum of compound **2**-expansion.

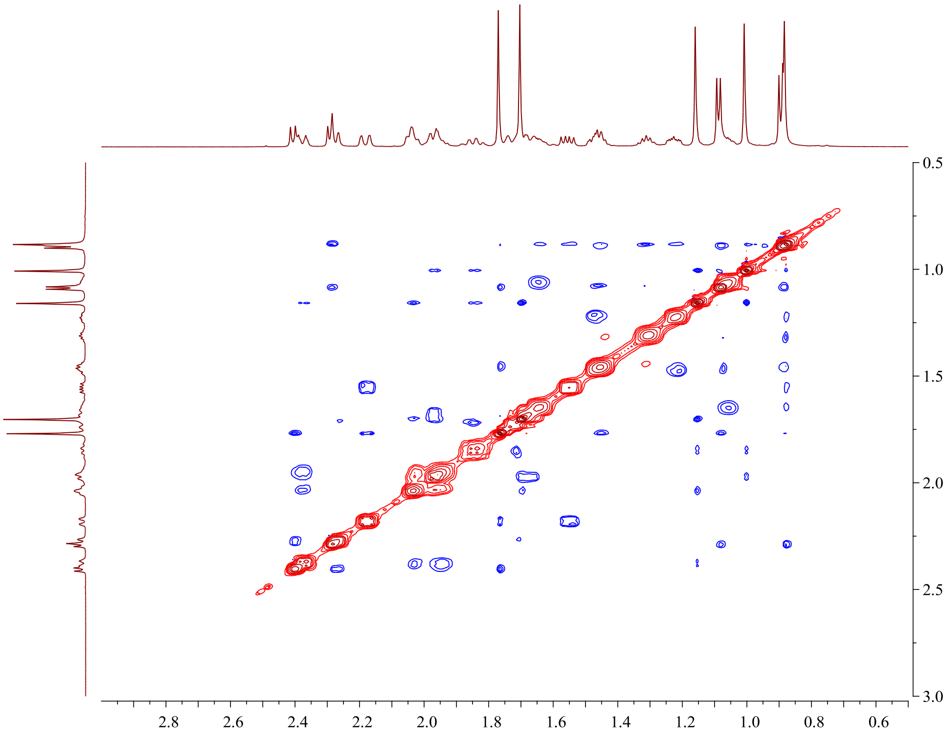

Fig. S22. HRESIMS report of compound **2**.

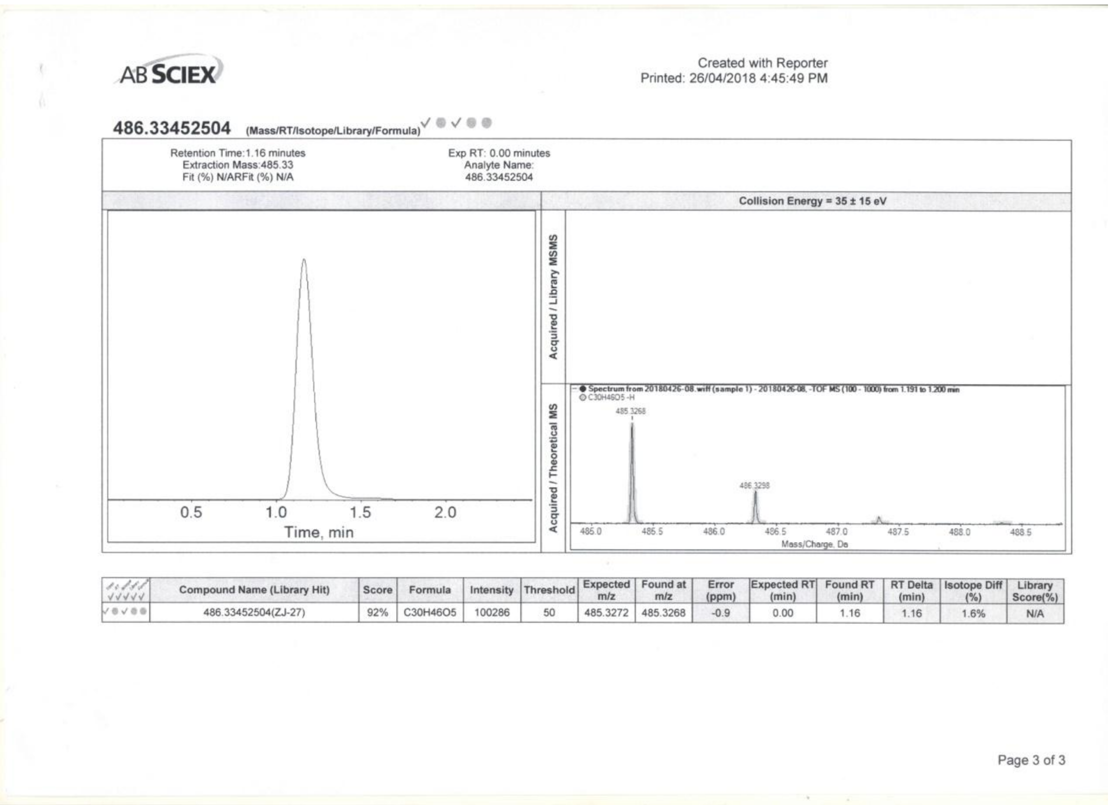

	Compound Name (Library Hit)	Score	Formula	Intensity	Threshold	Expected m/z	Found at m/z	Error (ppm)	Expected RT (min)	Found RT (min)	RT Delta (min)	Isotope Diff (%)	Library Score(%)
	486.33452504(ZJ-27)	92%	C30H46O5	100286	50	485.3272	485.3268	-0.9	0.00	1.16	1.16	1.6%	N/A

Fig. S23. Experimental ECD spectra of **1** (red curve) and **2** (black curve) in MeOH.

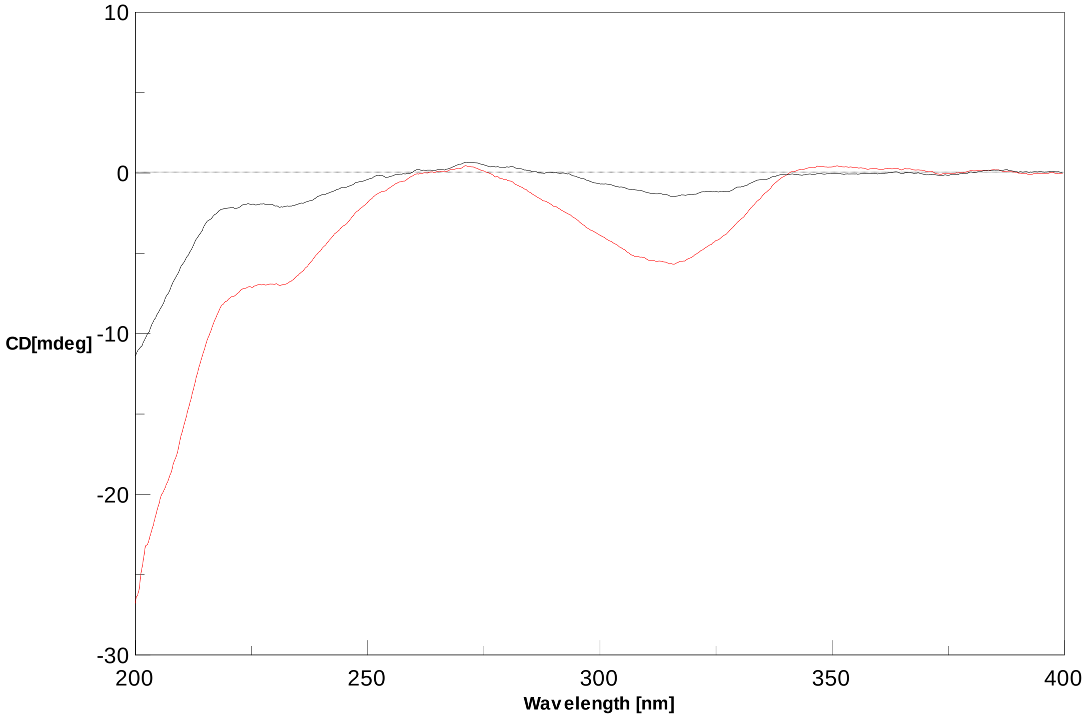

Fig. S24. ^{1}H NMR spectrum of compound **3** in CD3OD (400 MHz).

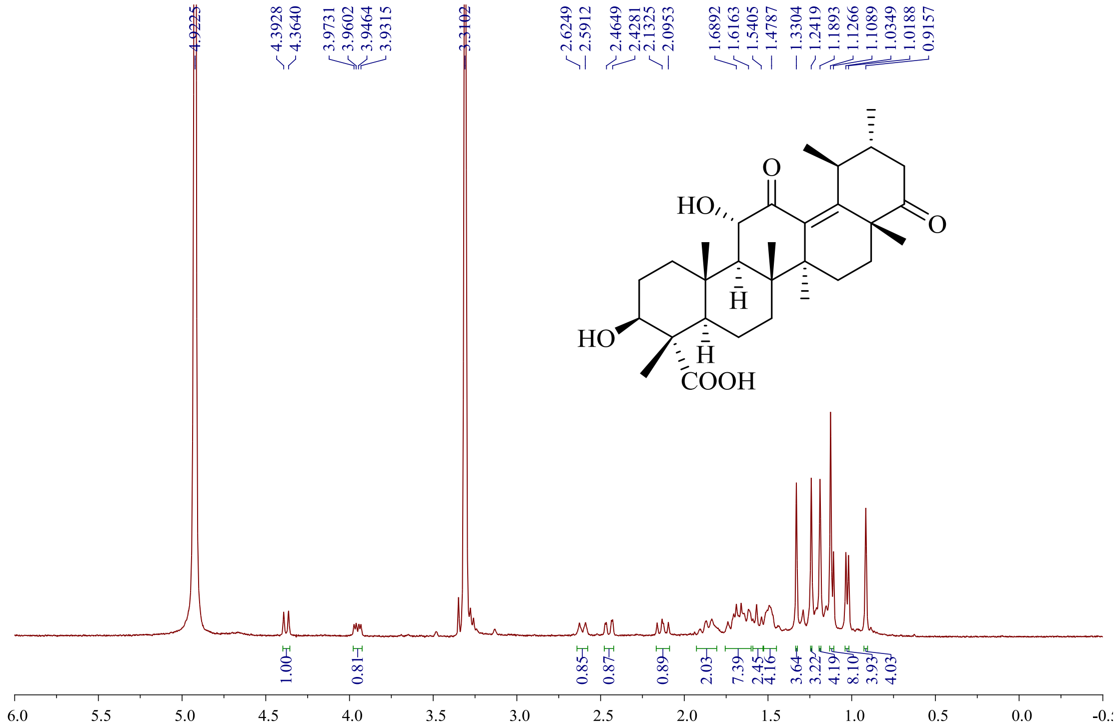

Fig. S25. ^{1}H NMR spectrum of compound **3**–expansion.

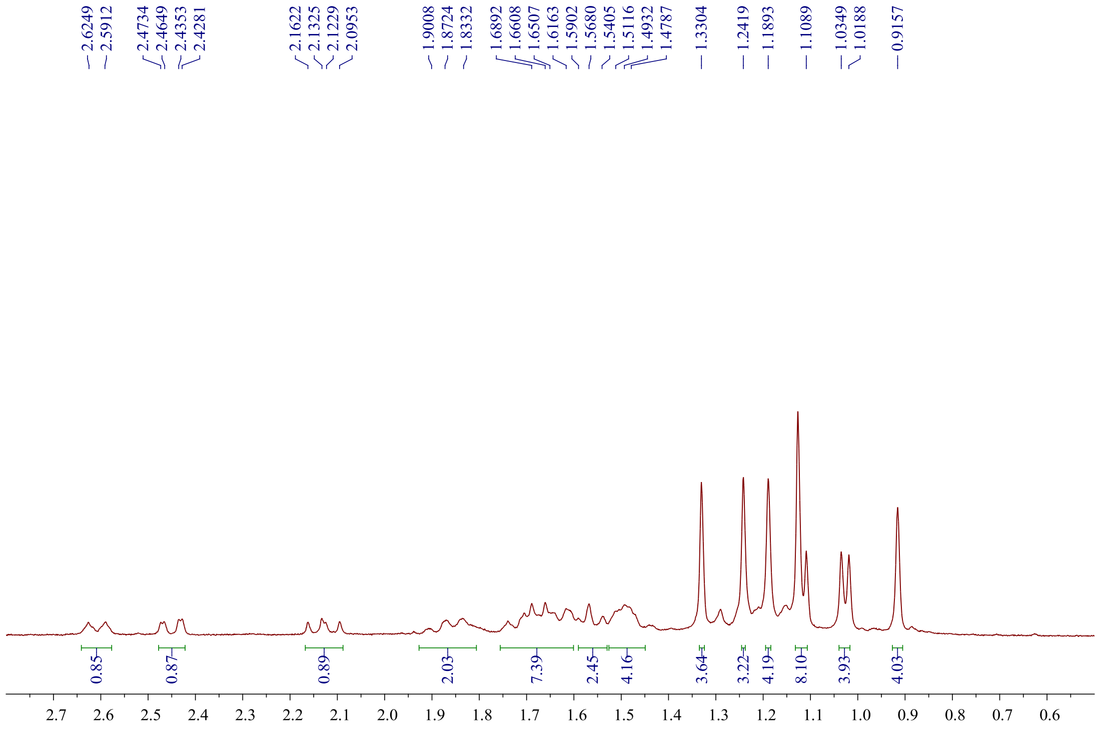

Fig. S26. ^{13}C NMR spectrum of compound **3** in CD3OD (150 MHz).

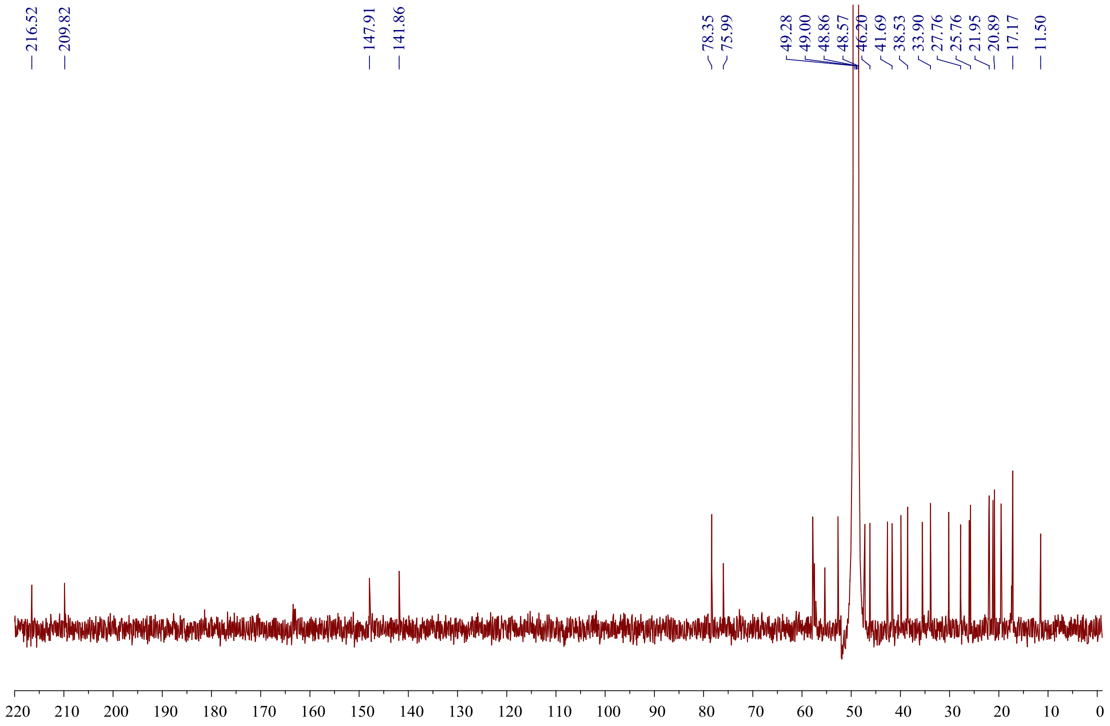

Fig. S27. ^{13}C NMR spectrum of compound **3**-expansion.

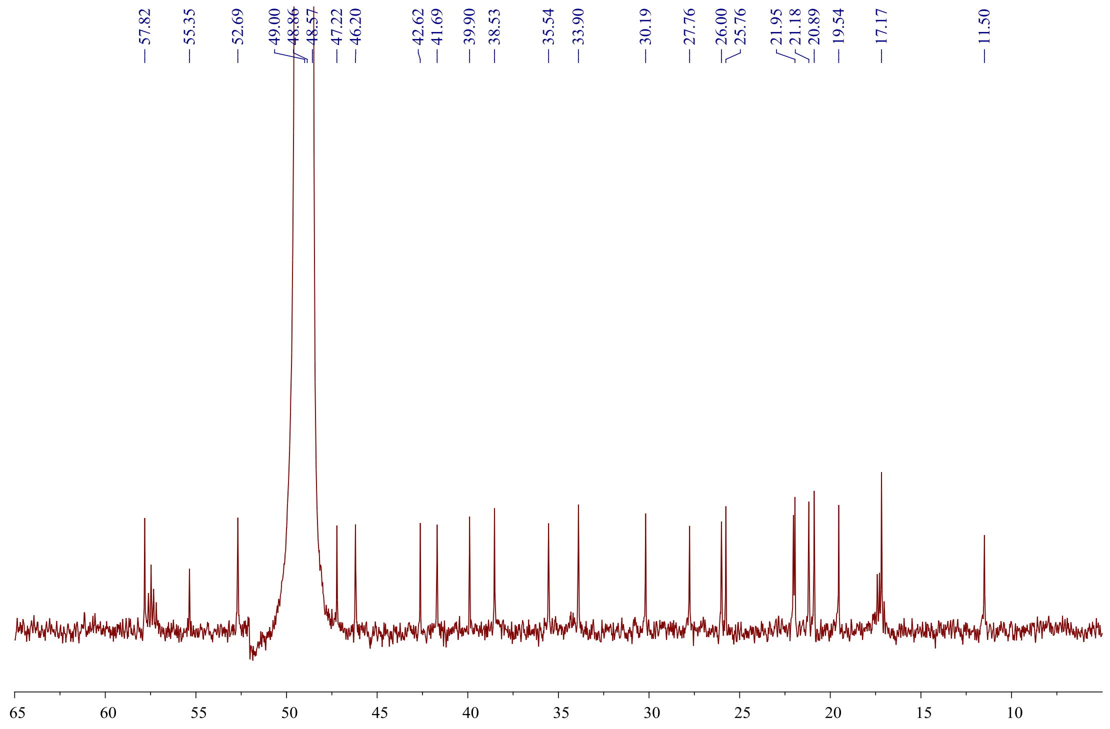

Fig. S28. HMBC spectrum of compound **3** in CD_3OD (600 MHz).

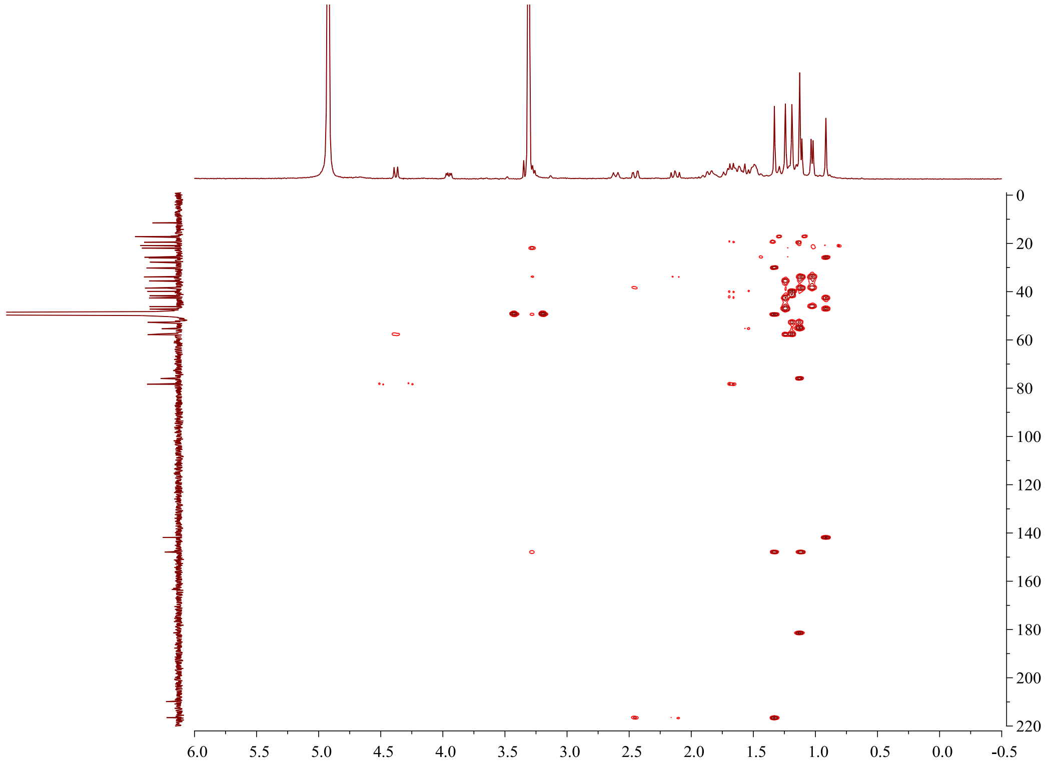

Fig. S29. HMBC spectrum of compound **3**–expansion.

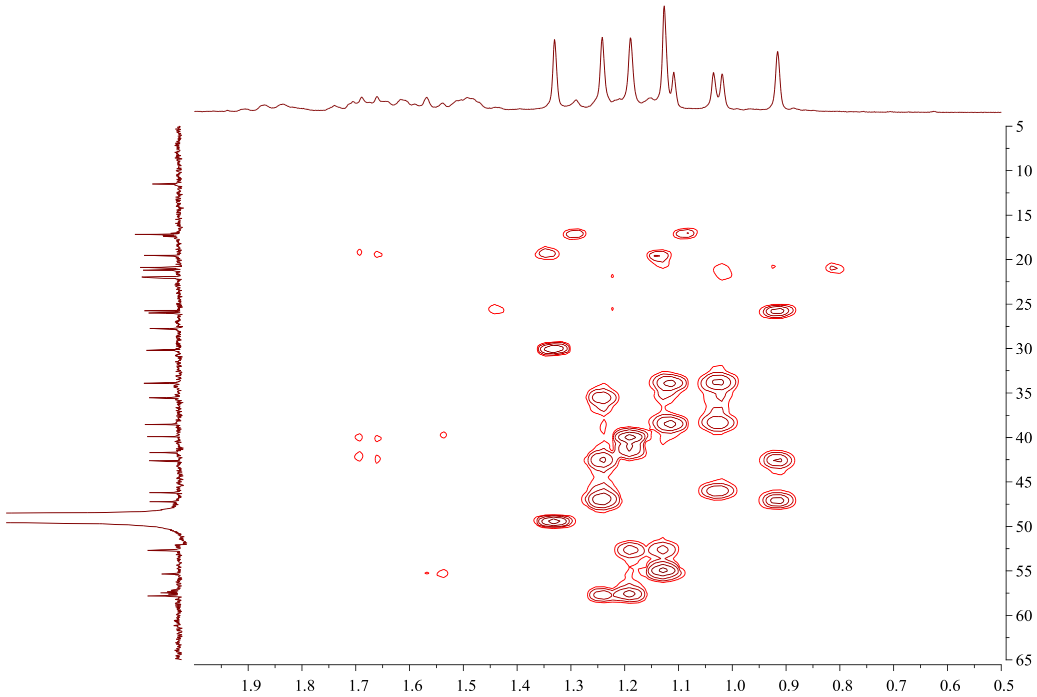

Fig. S30. ROESY spectrum of compound **3** in CD_3OD (600 MHz).

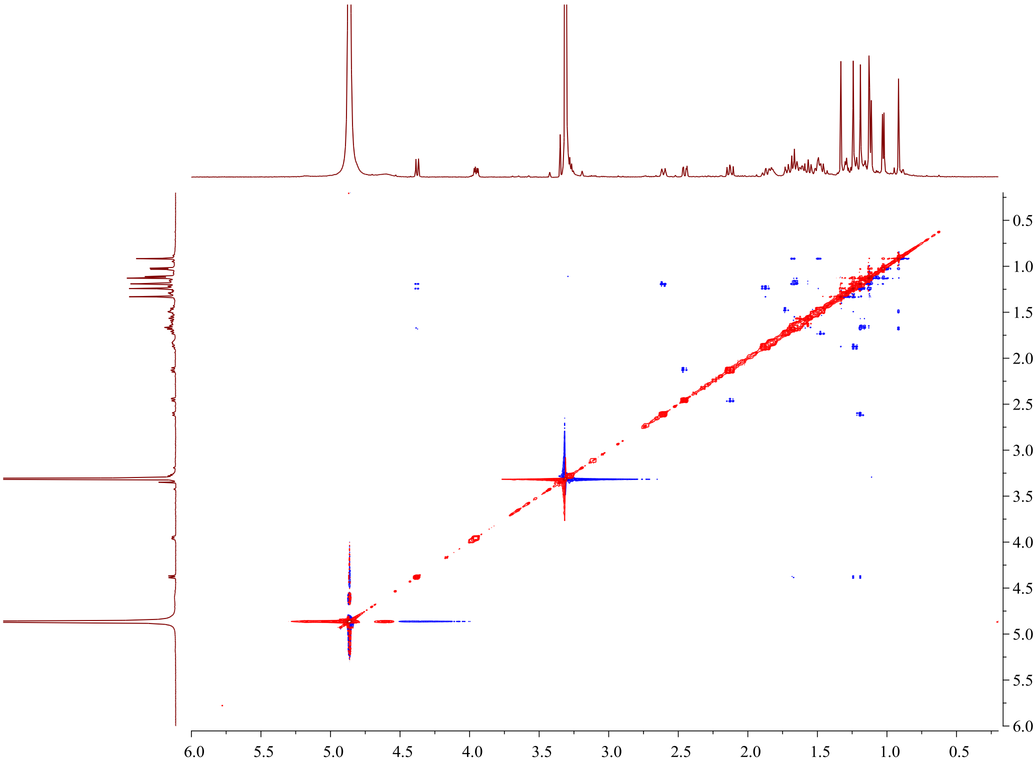

Fig. S31. HRESIMS report of compound **3**.

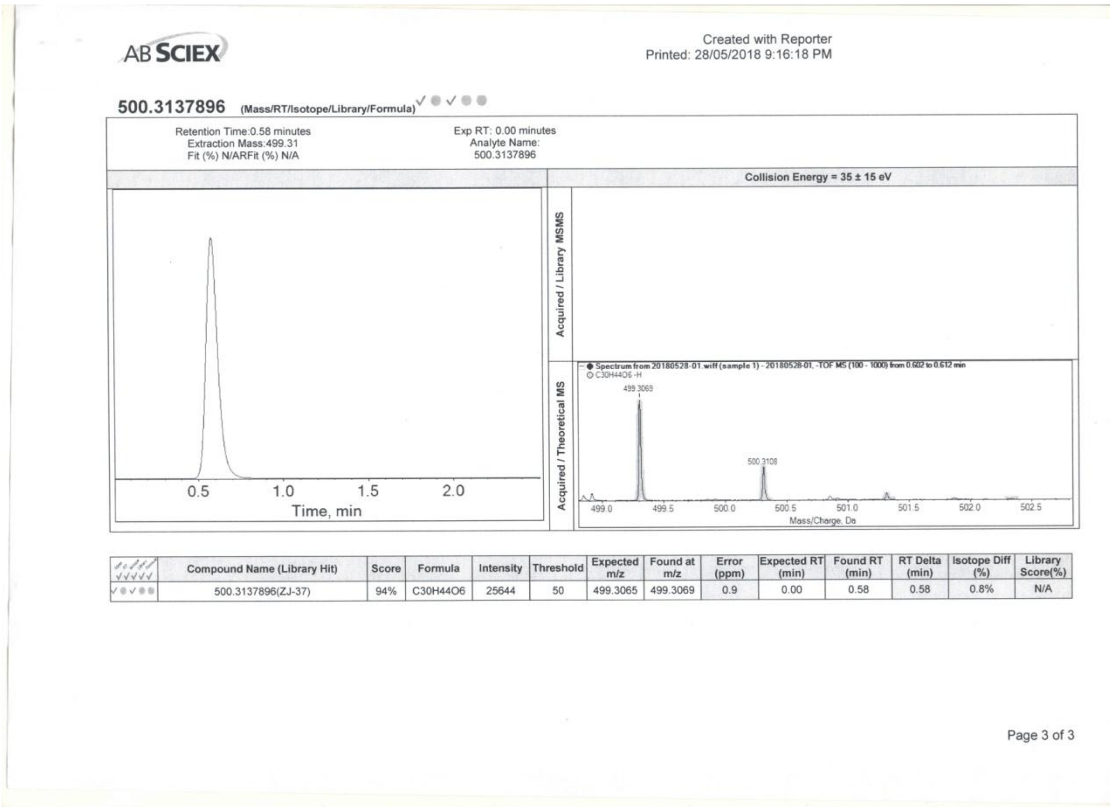

	Compound Name (Library Hit)	Score	Formula	Intensity	Threshold	Expected m/z	Found at m/z	Error (ppm)	Expected RT (min)	Found RT (min)	RT Delta (min)	Isotope Diff (%)	Library Score(%)
	500.3137896(ZJ-37)	94%	C30H44O6	25644	50	499.3065	499.3069	0.9	0.00	0.58	0.58	0.8%	N/A

Fig. S32. Experimental ECD spectrum of **3** in MeOH.

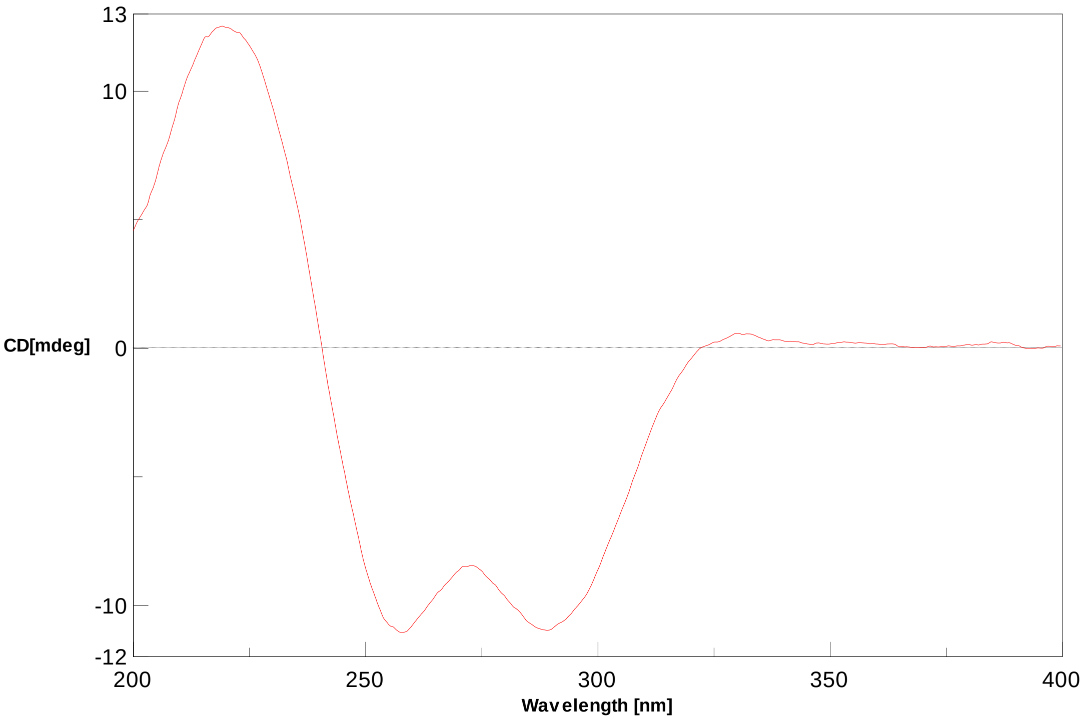

Fig. S33. ^{1}H NMR spectrum of compound **4** in C_5D_5N (400 MHz).

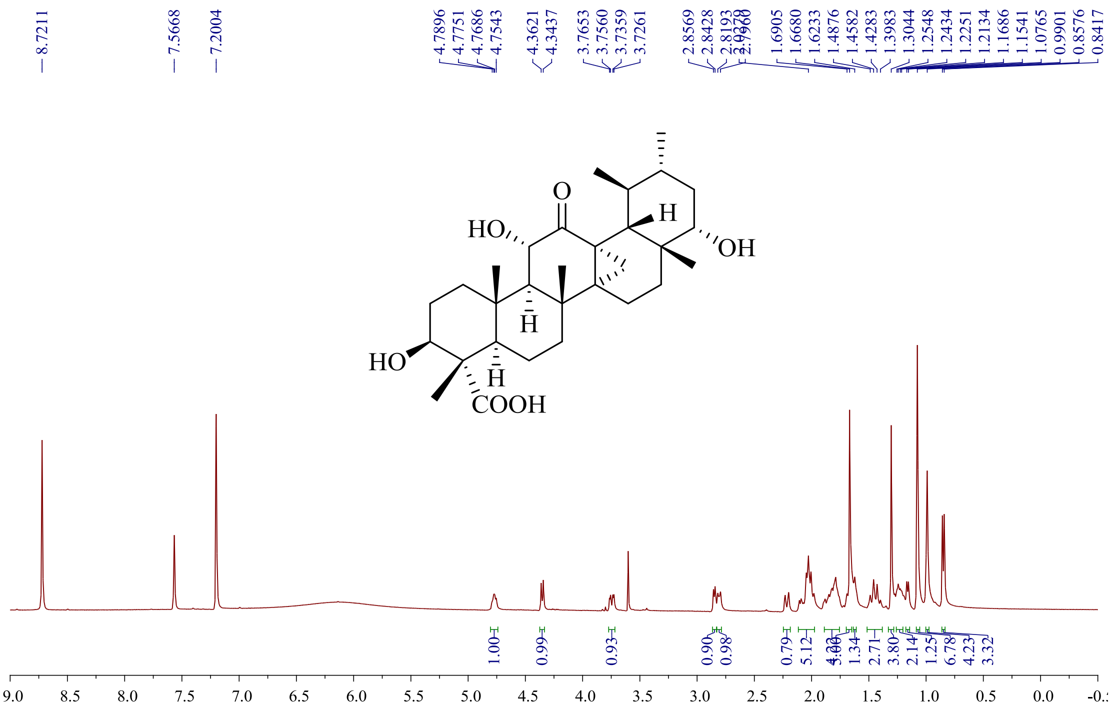

Fig. S34.^{1}H NMR spectrum of compound **4**-expansion.

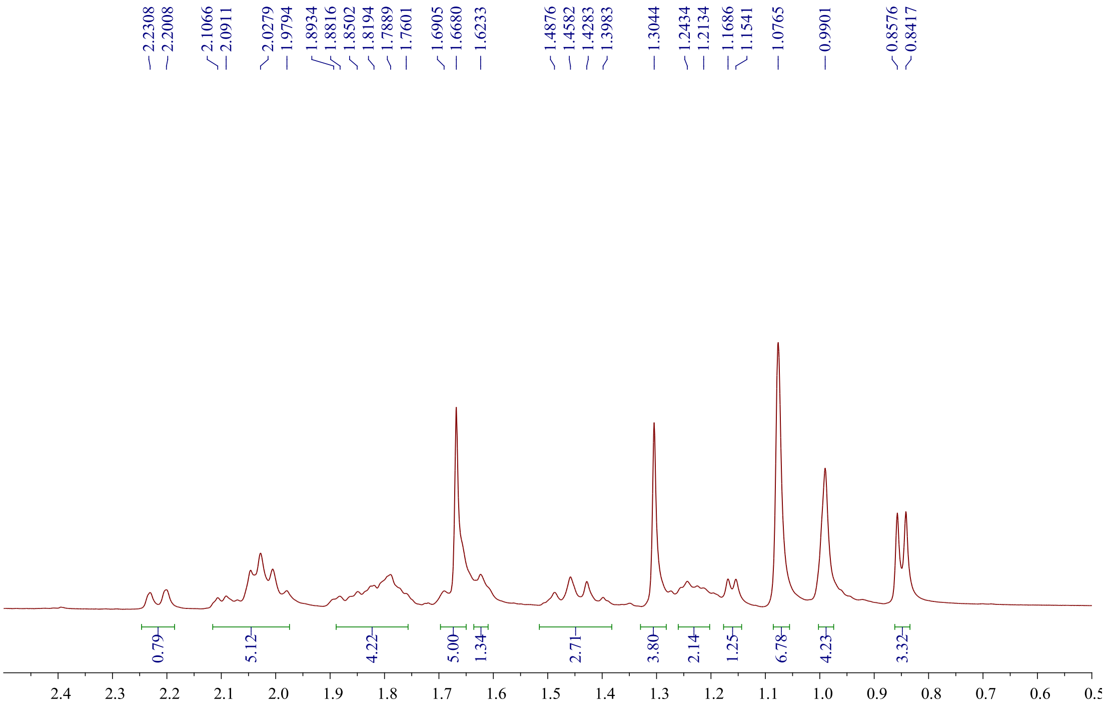

Fig. S35. ^{13}C NMR spectra of compound **4** in C_5D_5N (150 MHz).

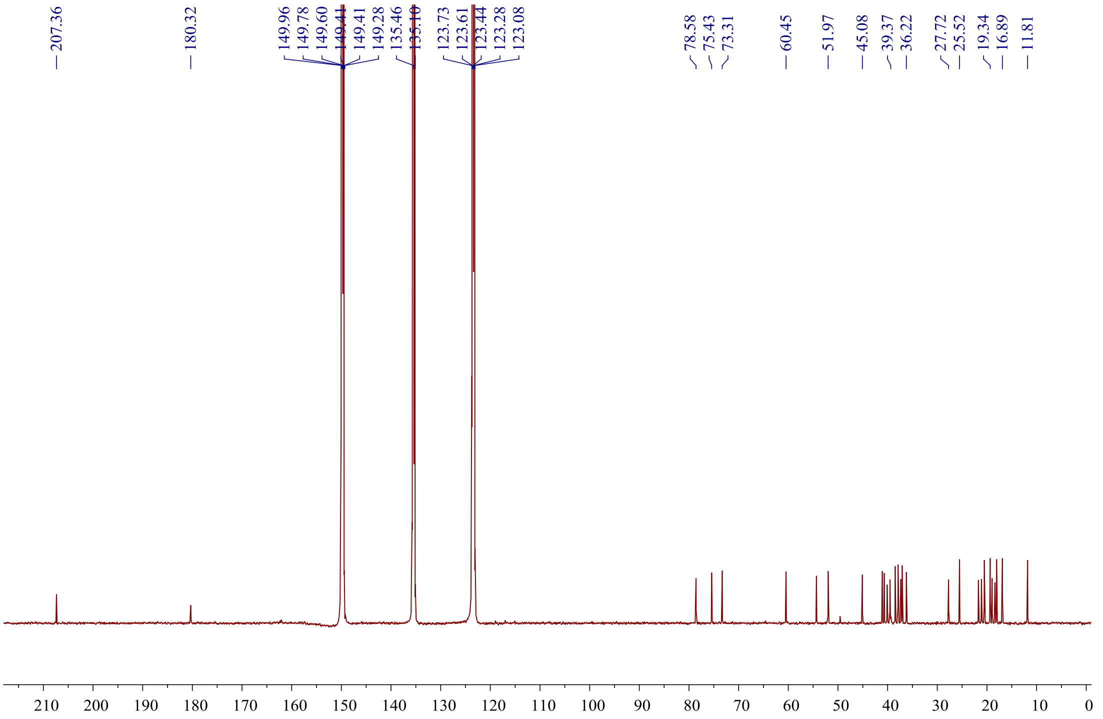

Fig. S36. ^{13}C NMR spectra of compound **4**-expansion.

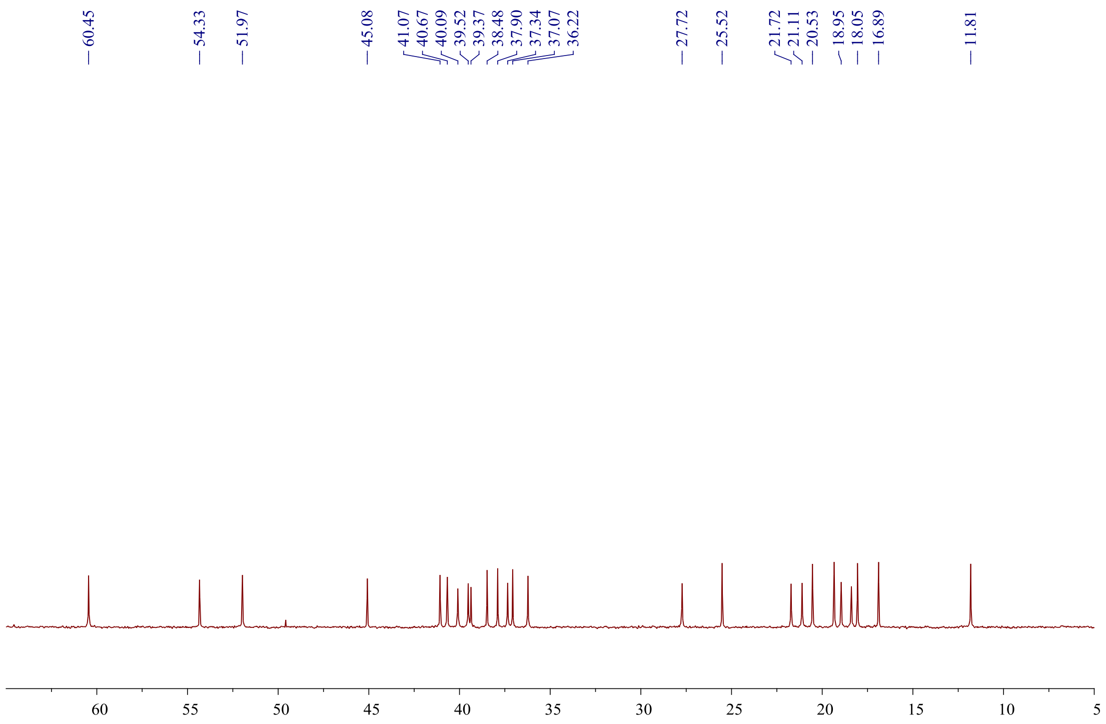

Fig. S37. DEPT 135 spectra of compound **4**.

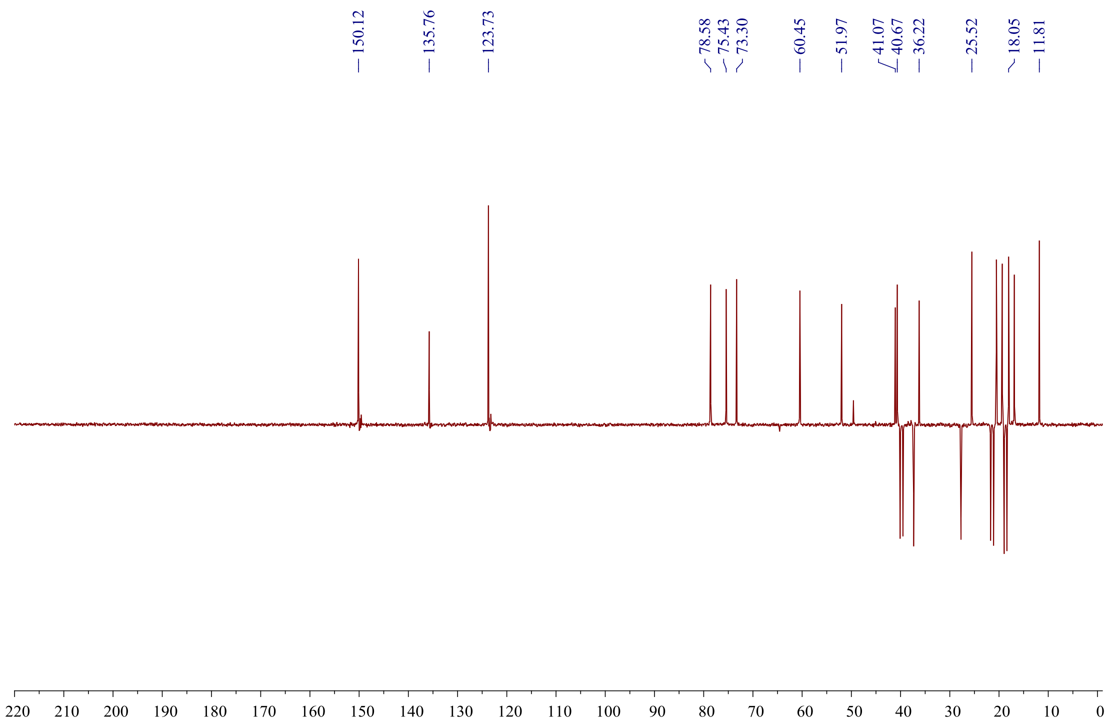

Fig. S38. HSQC spectrum of compound **4** in C_5D_5N (600 MHz).

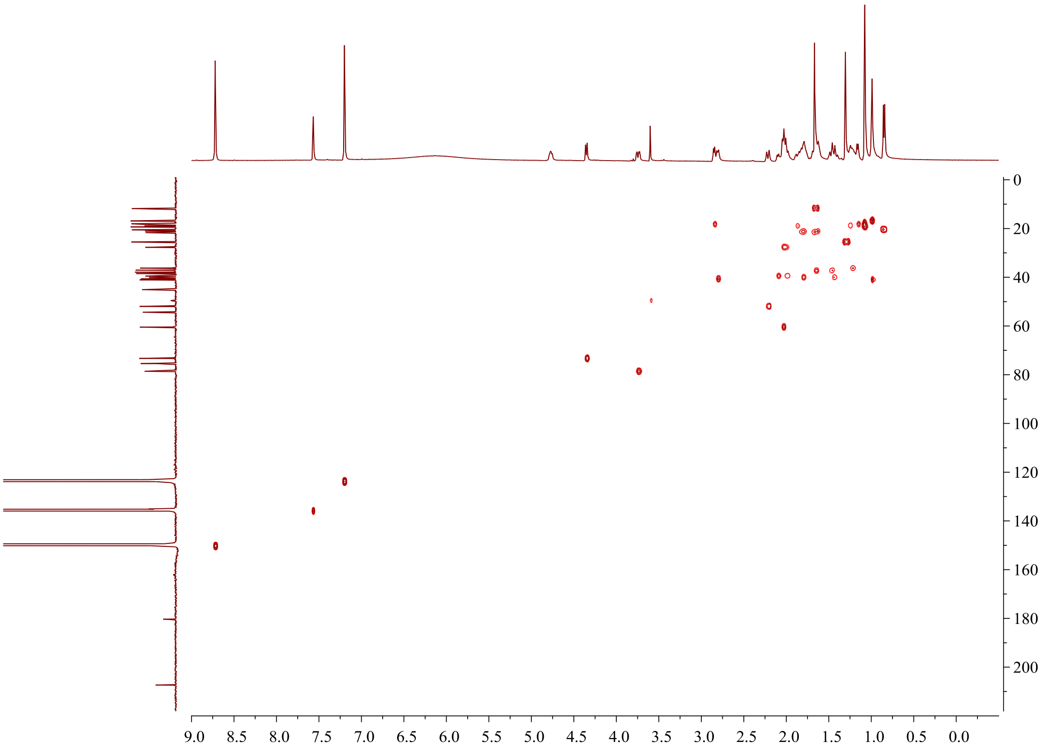

Fig. S39.^{1}H-^{1}H COSY spectrum of compound **4** in C_5D_5N (600 MHz).

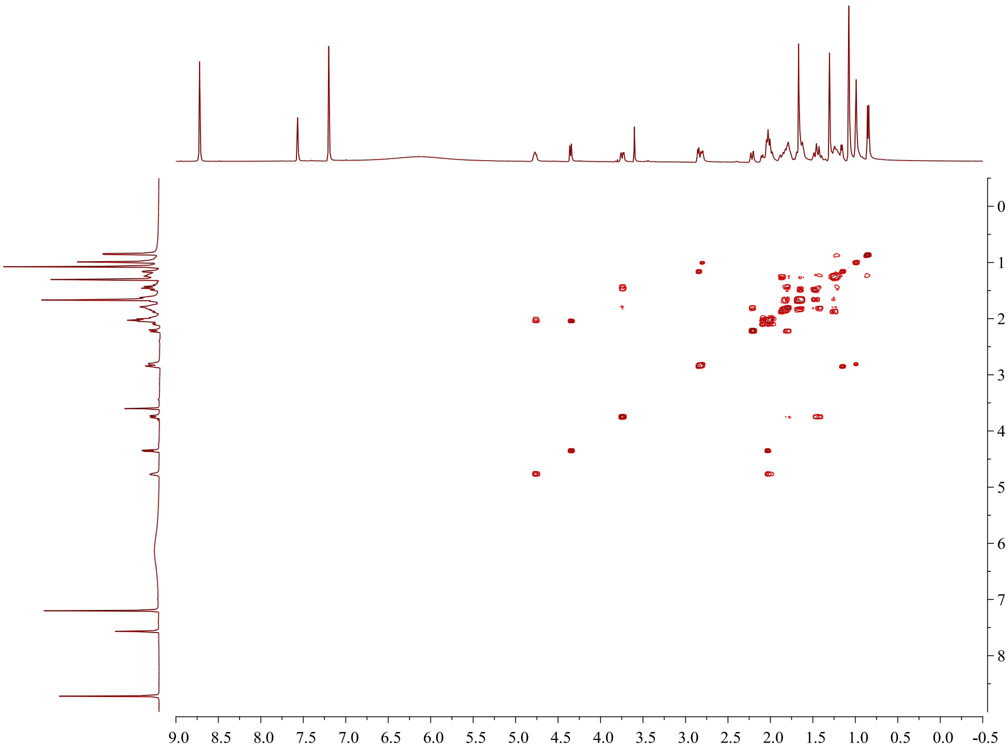

Fig. S40. HMBC spectrum of compound **4** in C_5D_5N (600 MHz).

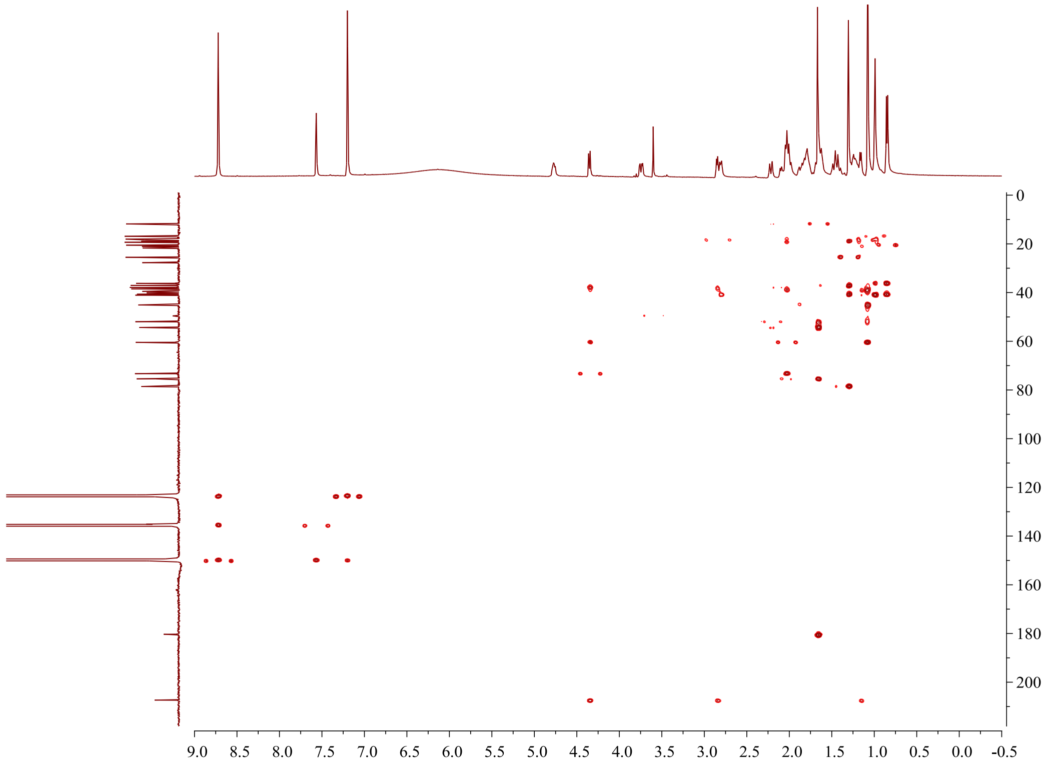

Fig. S41. HMBC spectrum of compound **4**-expansion.

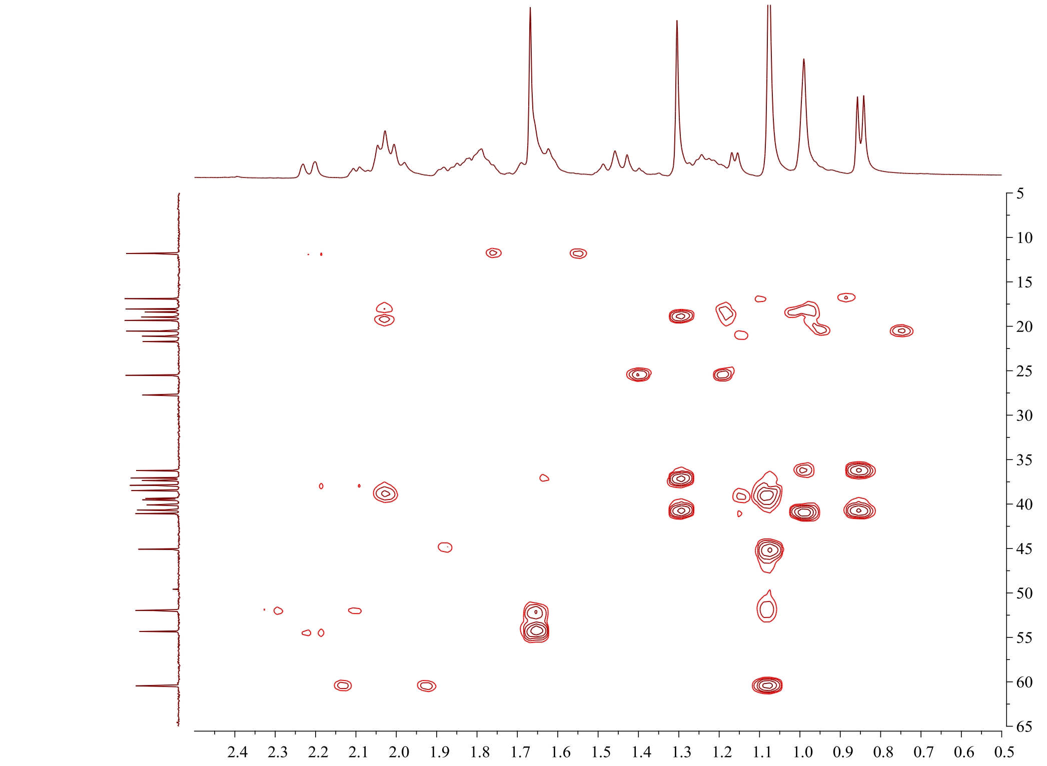

Fig. S42. ROESY spectrum of compound **4** in C_5D_5N (600 MHz).

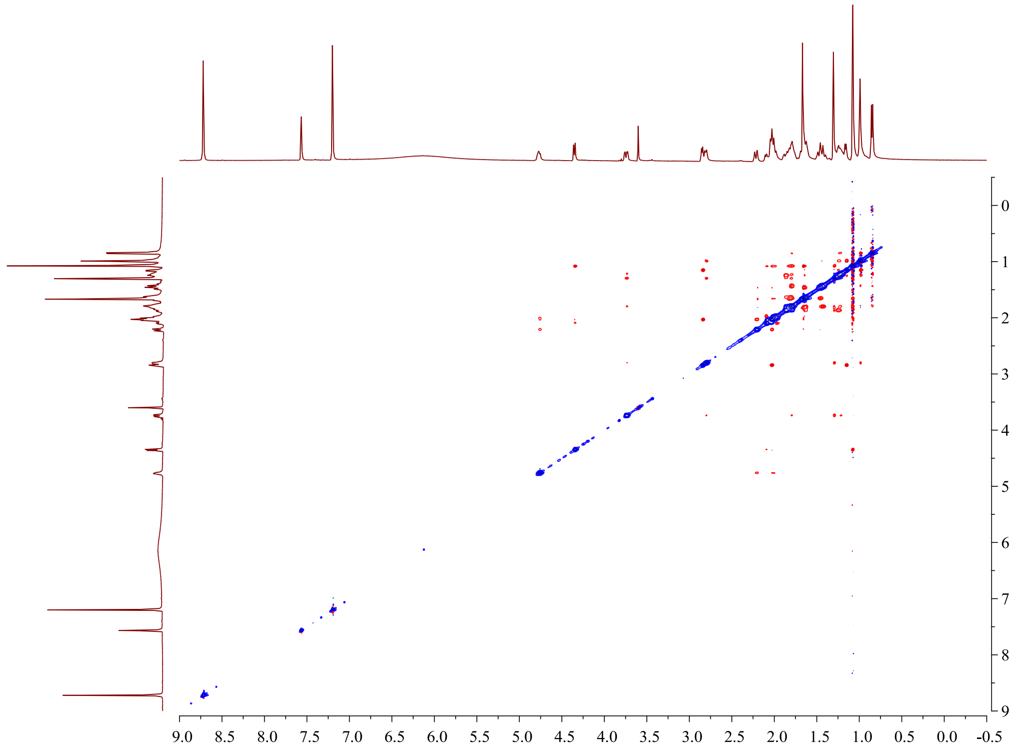

Fig. S43. HRESIMS report of compound **4**.

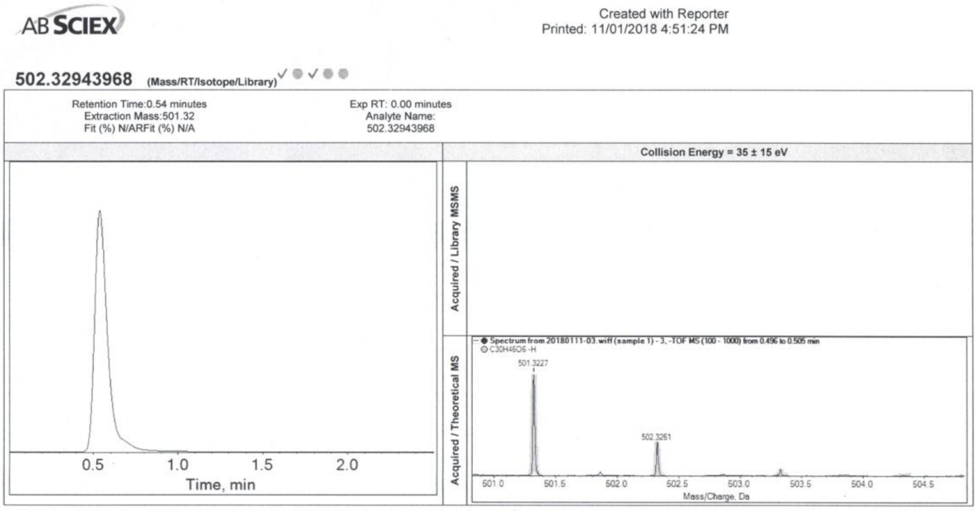

AB SCIEX

Created with Reporter
Printed: 11/01/2018 4:51:24 PM

502.32943968 (Mass/RT/Isotope/Library)

Retention Time:0.54 minutes
Extraction Mass:501.32
Fit (%) N/ARFit (%) N/A

Exp RT: 0.00 minutes
Analyte Name:
502.32943968

Collision Energy = 35 ± 15 eV

	Compound Name (Library Hit)	Score	Formula	Intensity	Threshold	Expected m/z	Found at m/z	Error (ppm)	Expected RT (min)	Found RT (min)	RT Delta (min)	Isotope Diff (%)	Library Score(%)
✓●✓●●	502.32943968(ZJ-12)	92%	C30H46O6	58463	100	501.3222	501.3227	1.1	0.00	0.54	0.54	1.1%	N/A

Page 3 of 3

Fig. S44. ^{1}H NMR spectrum of compound **5** in C_5D_5N (400 MHz).

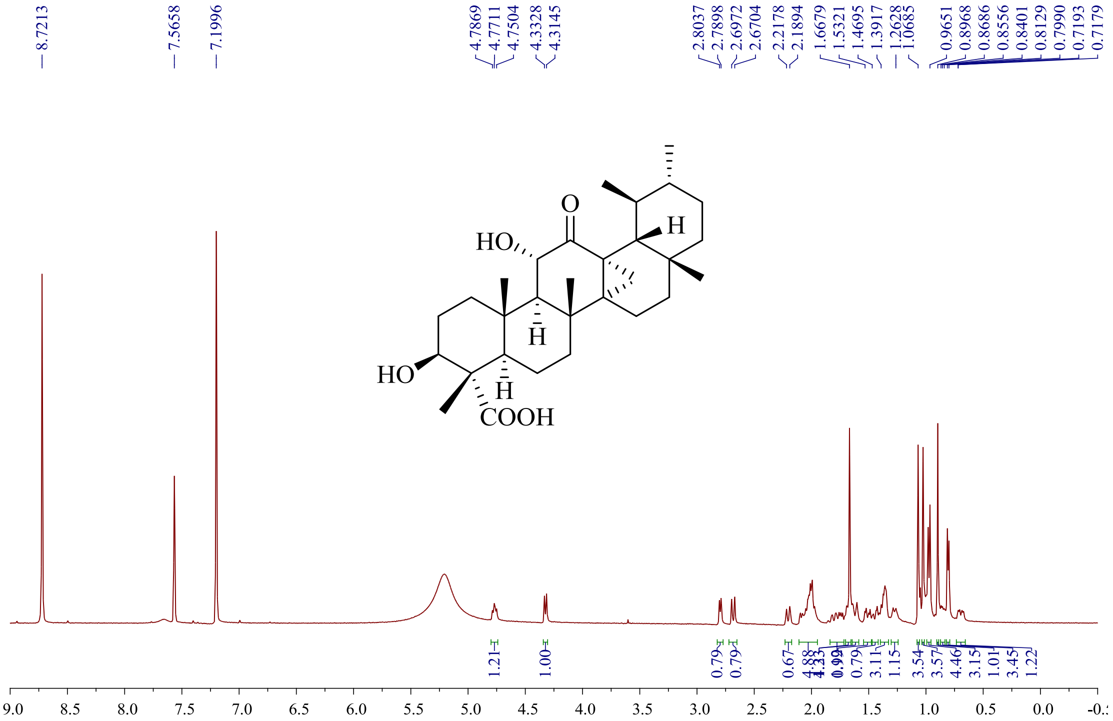

Fig. S45. ^{1}H NMR spectrum of compound **5**–expansion.

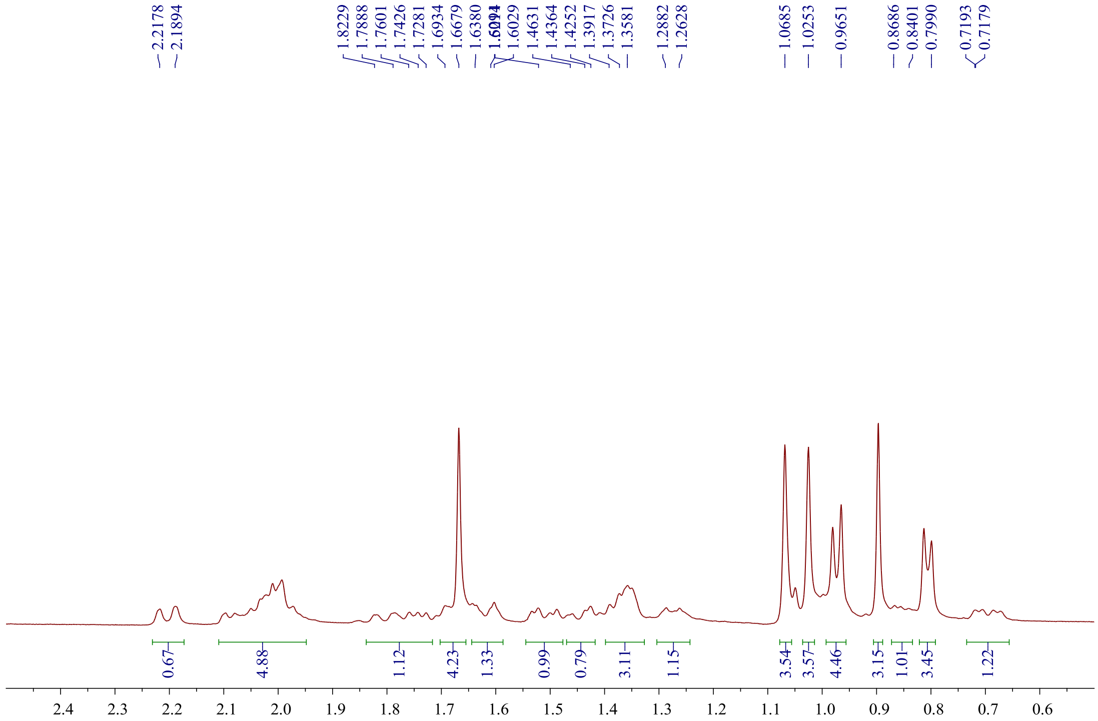

Fig. S46. ^{13}C NMR spectrum of compound **5** in C_5D_5N (150 MHz).

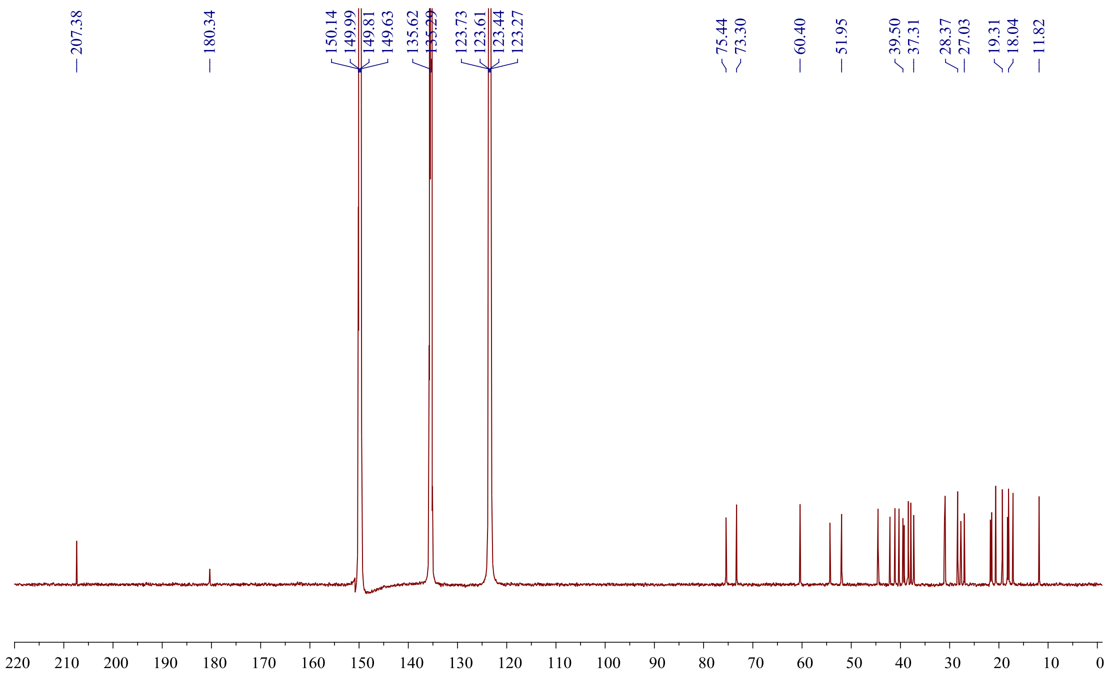

Fig. S47. ^{13}C NMR spectrum of compound **5**–expansion.

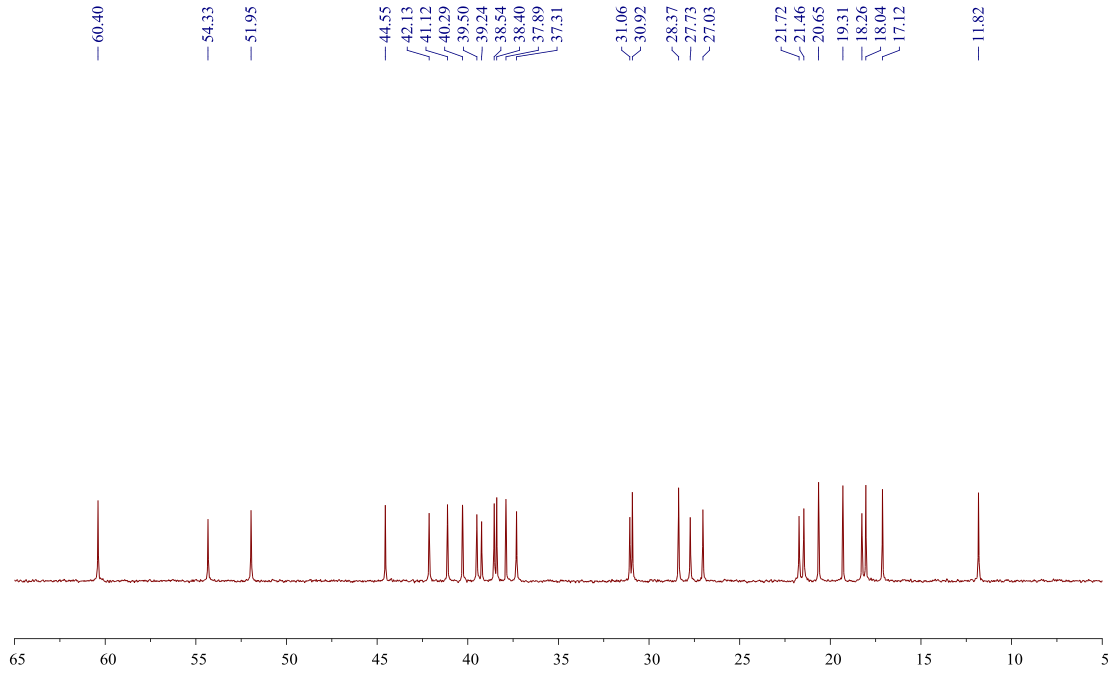

Fig. S48. HMBC spectrum of compound **5** in C_5D_5N (600 MHz).

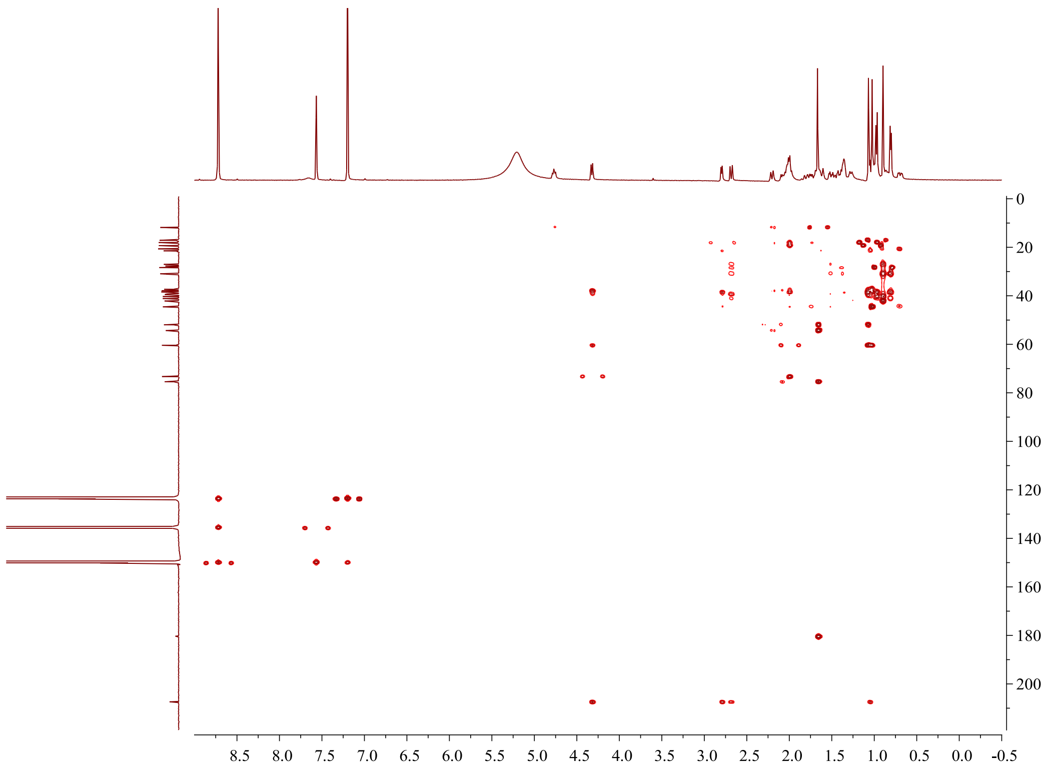

Fig. S49. HMBC spectrum of compound **5**–expansion.

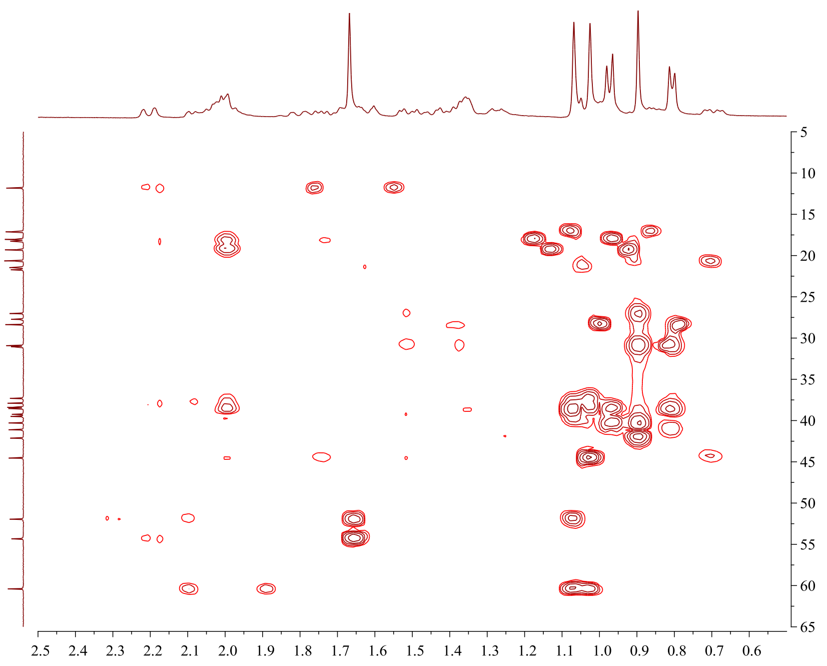

Fig. S50. ROESY spectrum of compound **5** in C_5D_5N (600 MHz).

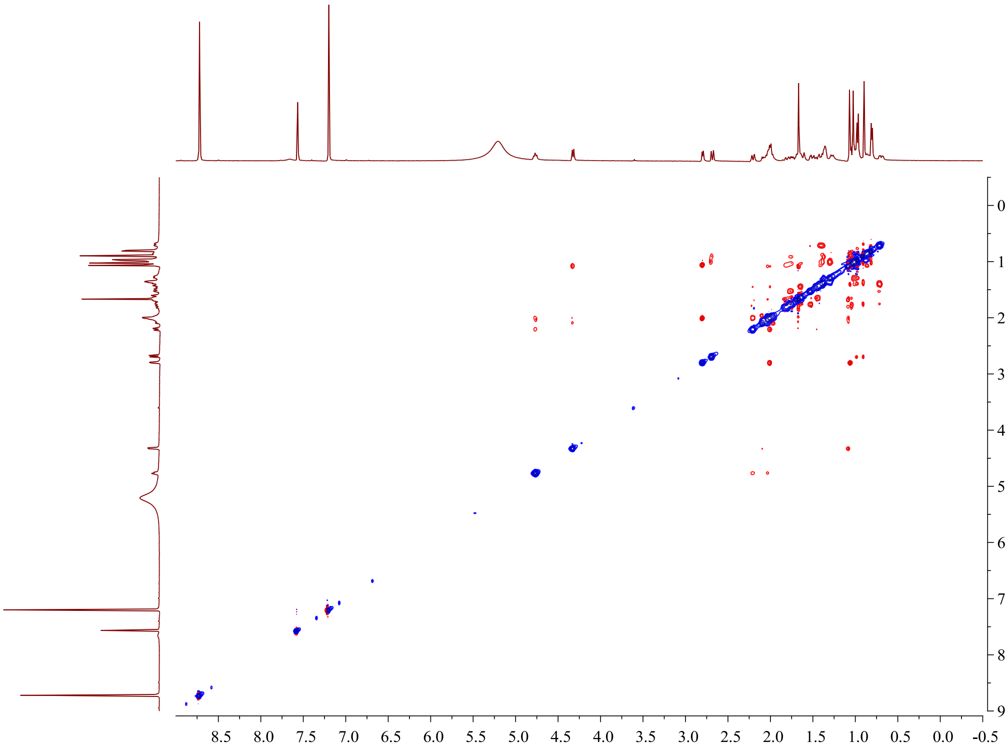

Fig. S51. HRESIMS report of compound **5**.

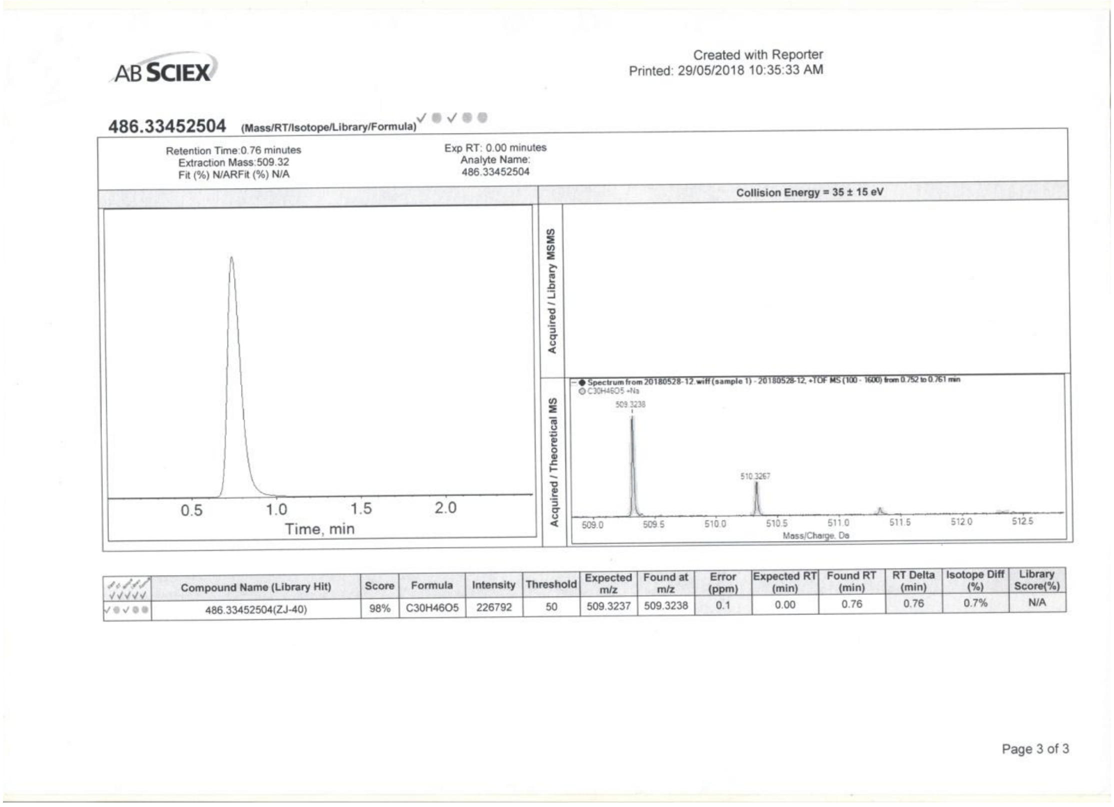

AB SCIEX

Created with Reporter
Printed: 29/05/2018 10:35:33 AM

486.33452504 (Mass/RT/Isotope/Library/Formula)

Retention Time:0.76 minutes
Extraction Mass:509.32
Fit (%) N/ARFit (%) N/A

Exp RT: 0.00 minutes
Analyte Name:
486.33452504

Collision Energy = 35 ± 15 eV

	Compound Name (Library Hit)	Score	Formula	Intensity	Threshold	Expected m/z	Found at m/z	Error (ppm)	Expected RT (min)	Found RT (min)	RT Delta (min)	Isotope Diff (%)	Library Score(%)
	486.33452504(ZJ-40)	98%	C30H46O5	226792	50	509.3237	509.3238	0.1	0.00	0.76	0.76	0.7%	N/A

Page 3 of 3

Fig. S52. Experimental ECD spectra of **4** (red curve) and **5** (black curve) in MeOH..

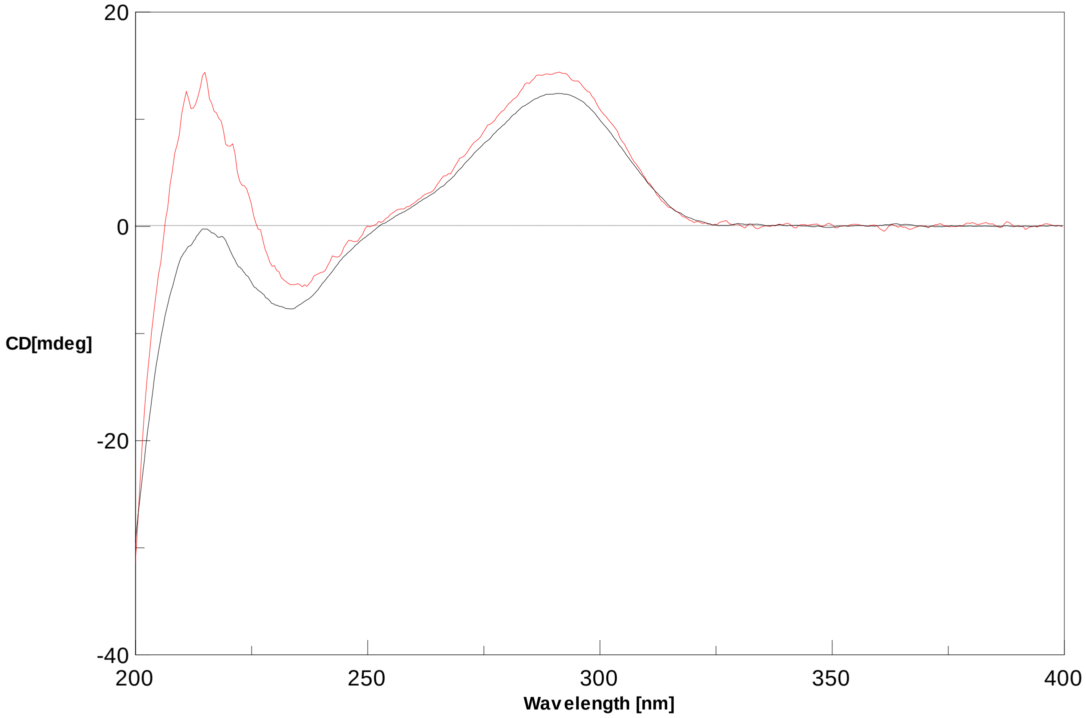

Fig. S53. ^{1}H NMR spectrum of compound **6** in C_5D_5N (400 MHz).

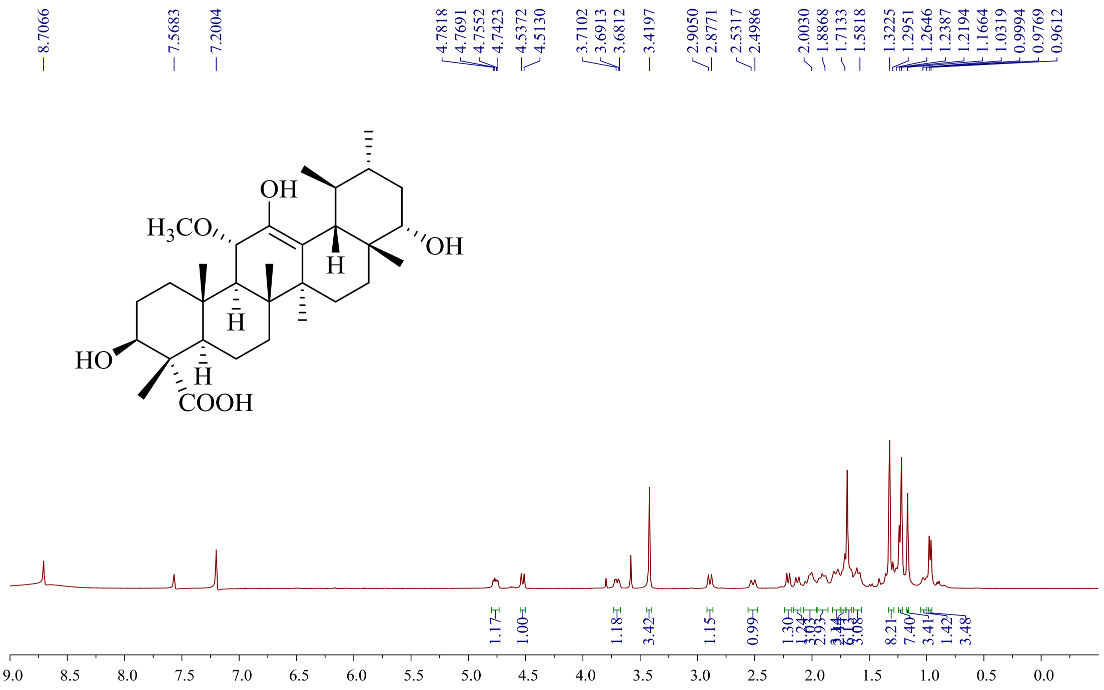

Fig. S54. ^{1}H NMR spectrum of compound **6**–expansion.

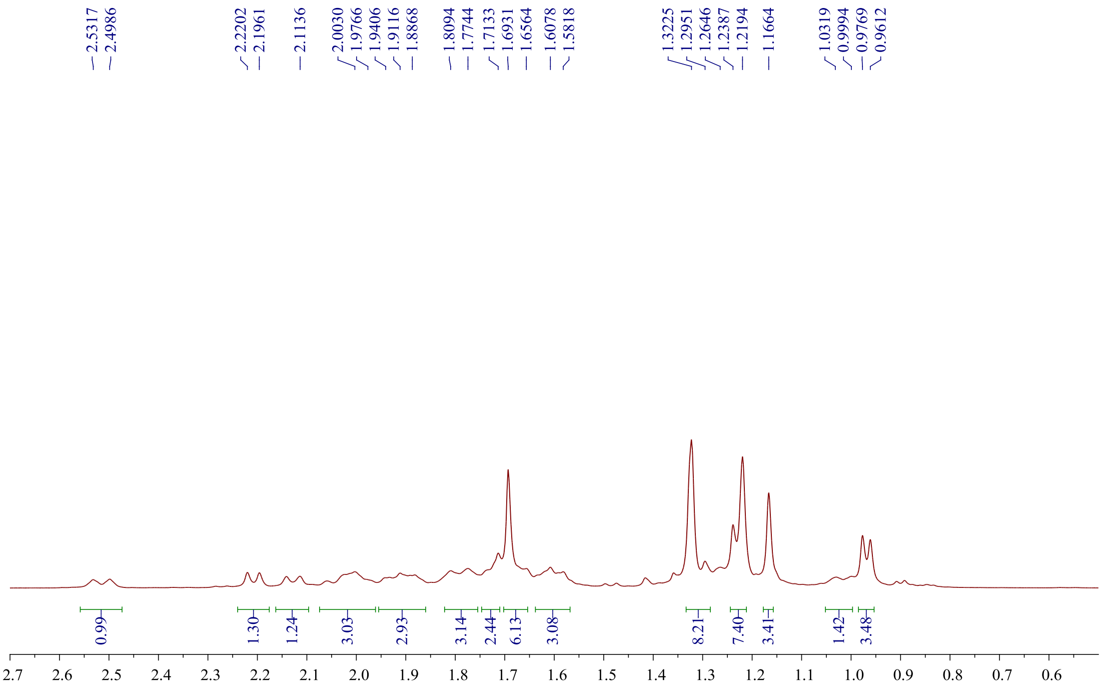

Fig. S55. ^{13}C NMR spectrum of compound **6** in C_5D_5N (150 MHz).

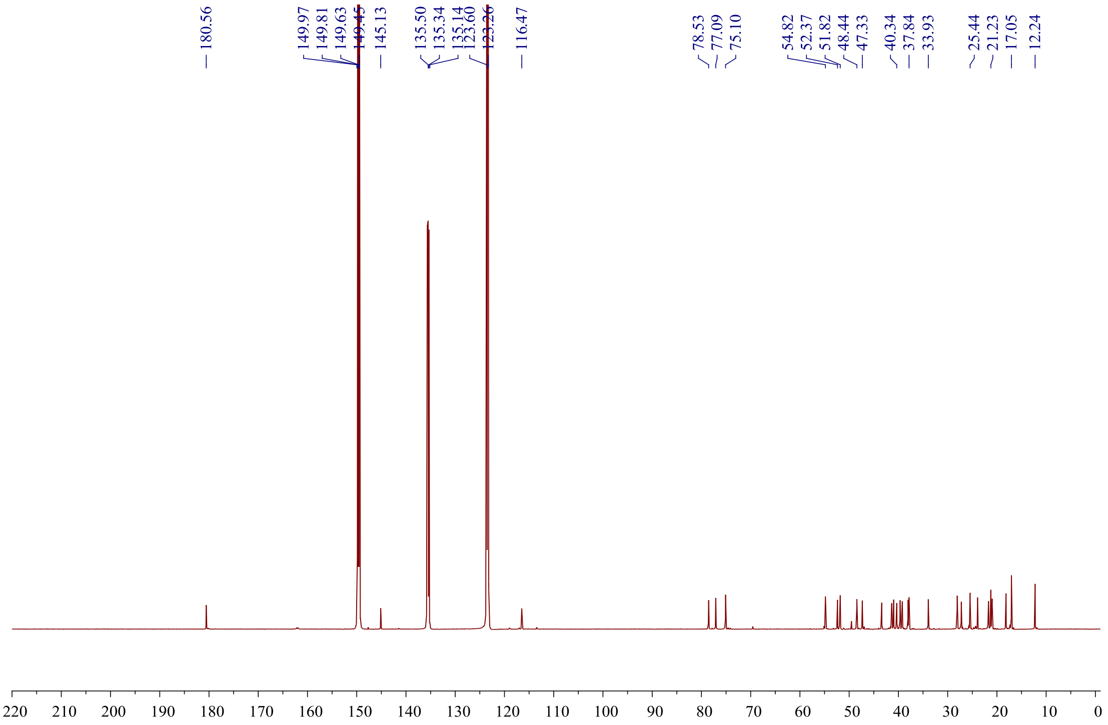

Fig. S56. ^{13}C NMR spectrum of compound **6**–expansion.

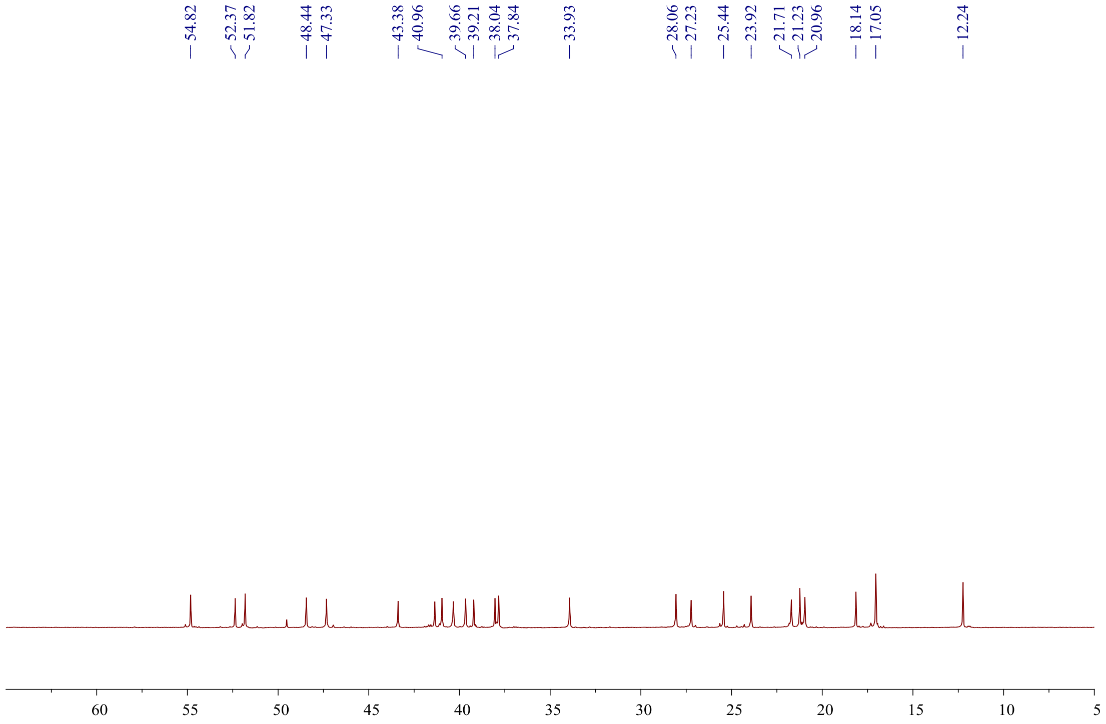

Fig. S57. HSQC spectrum of compound **6** in C_5D_5N (600 MHz).

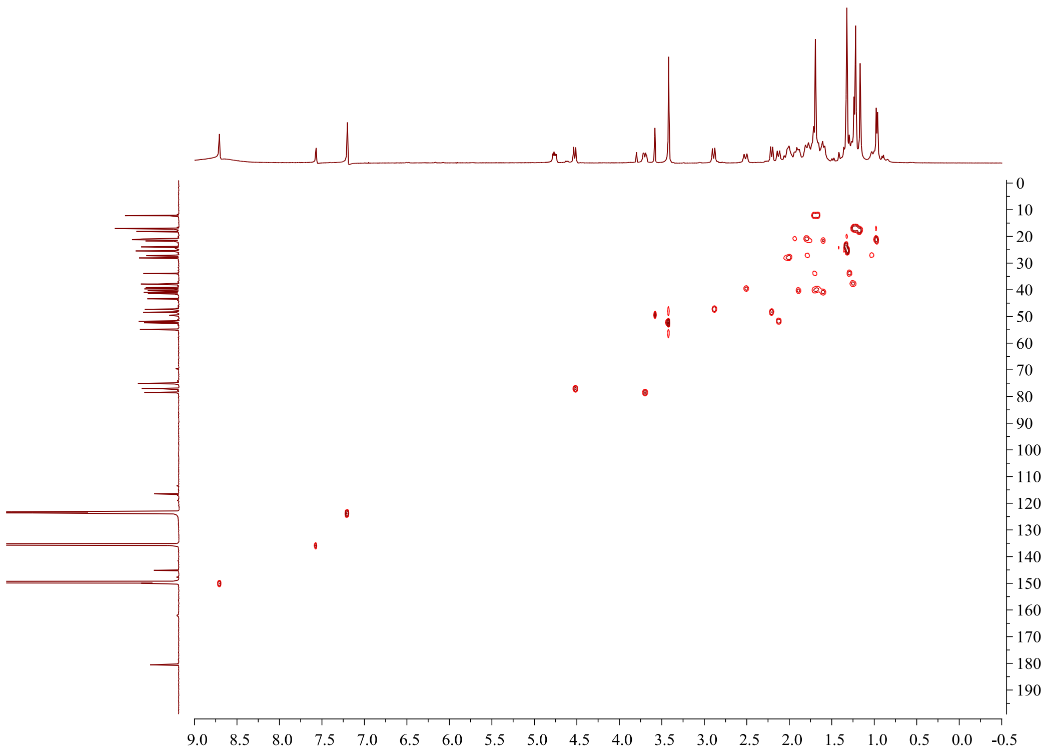

Fig. S58. HMBC spectrum of compound **6** in C_5D_5N (600 MHz).

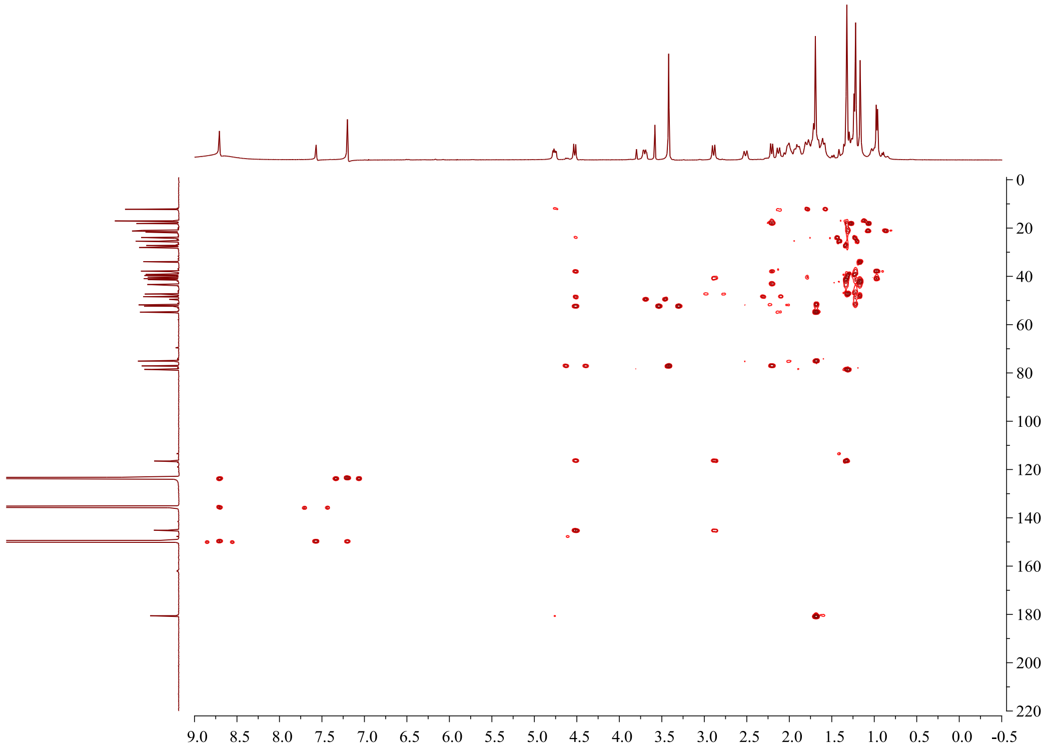

Fig. S59. HMBC spectrum of compound **6**–expansion.

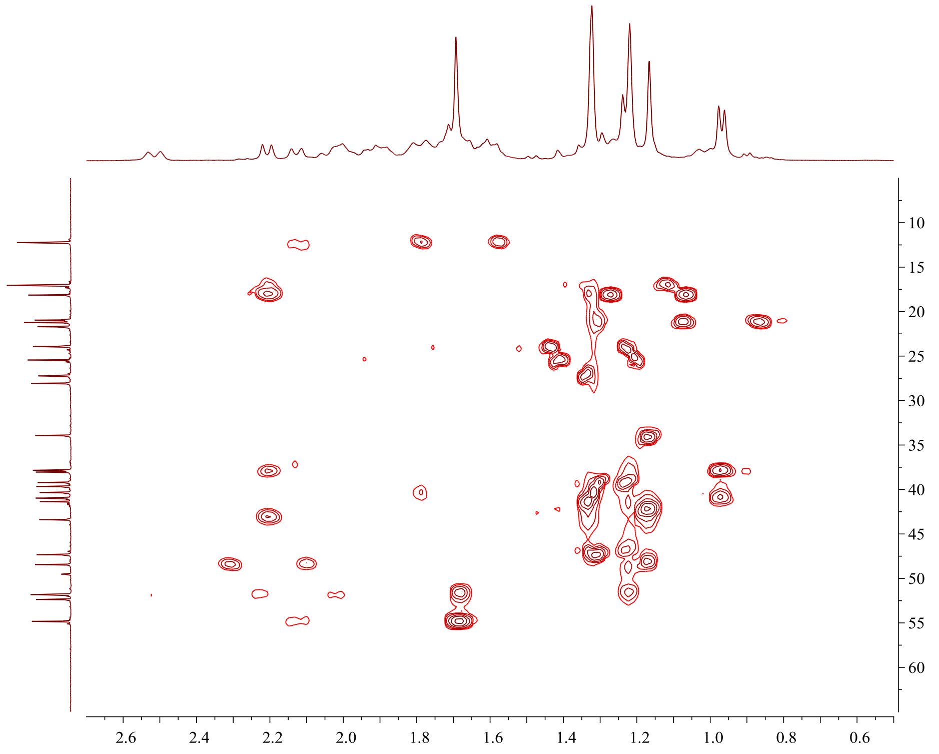

Fig. S60. ROESY spectrum of compound **6** in C_5D_5N (600 MHz).

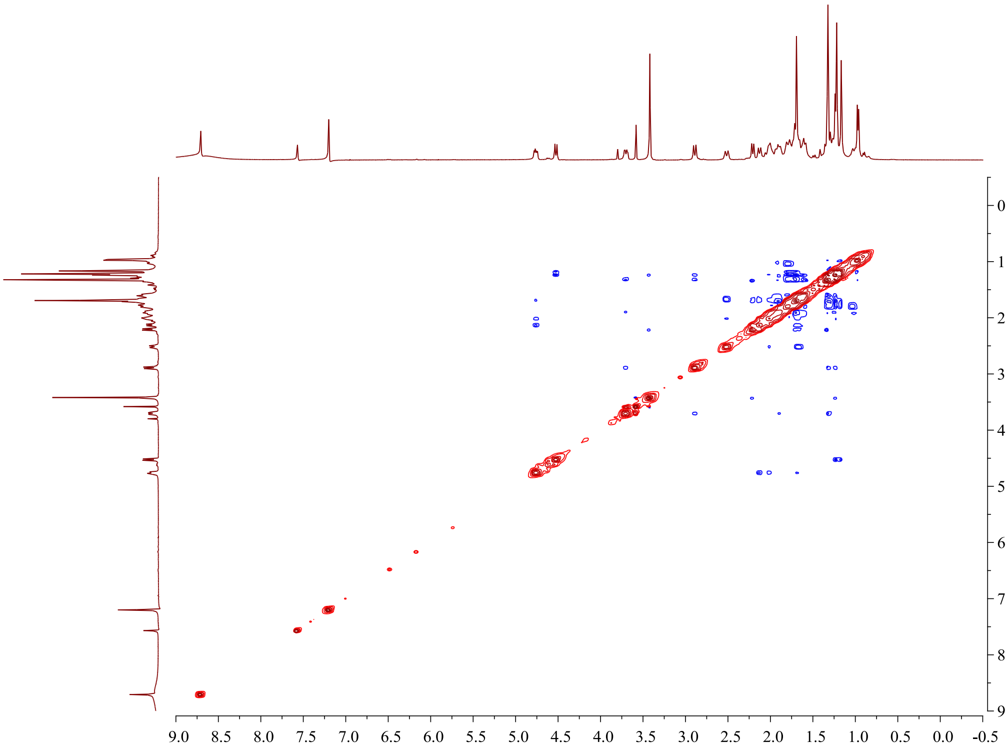

Fig. S61. HRESIMS report of compound **6**.

0

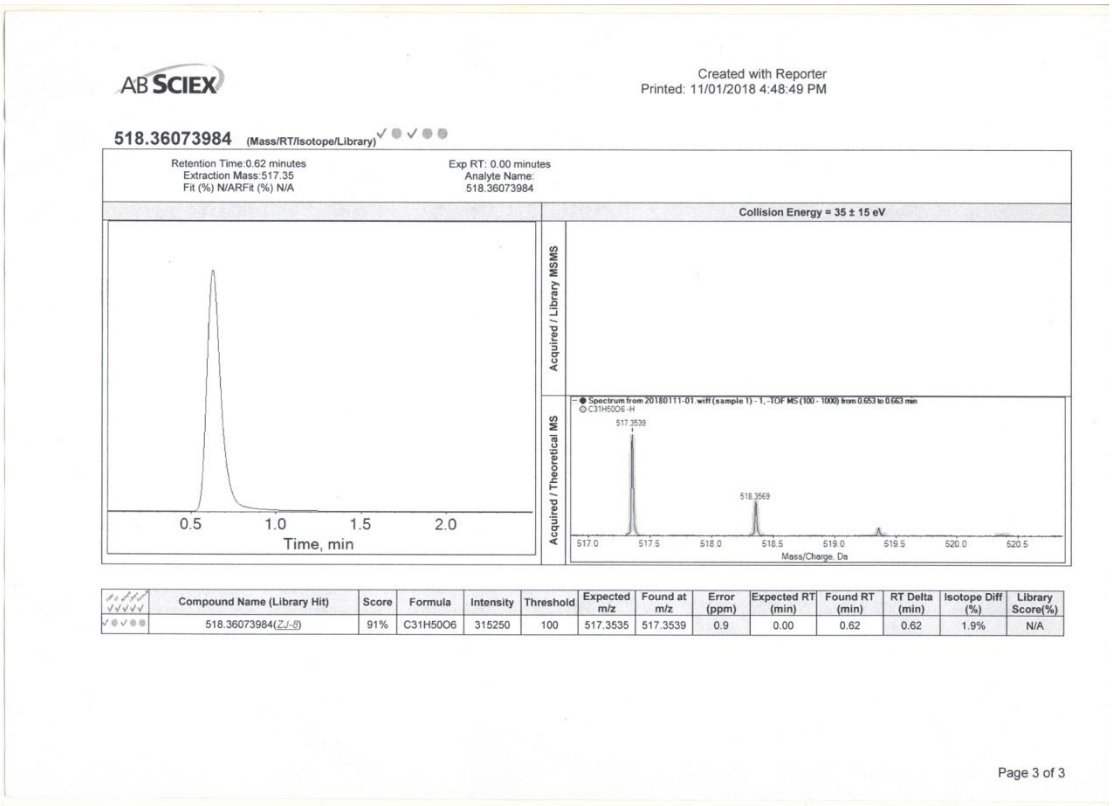

AB SCIEX

Created with Reporter
Printed: 11/01/2018 4:48:49 PM

518.36073984 (Mass/RT/Isotope/Library)

Retention Time:0.62 minutes
Extraction Mass:517.35
Fit (%) N/ARFit (%) N/A

Exp RT: 0.00 minutes
Analyte Name:
518.36073984

Collision Energy = 35 ± 15 eV

	Compound Name (Library Hit)	Score	Formula	Intensity	Threshold	Expected m/z	Found at m/z	Error (ppm)	Expected RT (min)	Found RT (min)	RT Delta (min)	Isotope Diff (%)	Library Score(%)
	518.36073984(ZJ-8)	91%	C31H50O6	315250	100	517.3535	517.3539	0.9	0.00	0.62	0.62	1.9%	N/A

Page 3 of 3

Fig. S62. ^{1}H NMR spectrum of compound **7** in C_5D_5N (400 MHz).

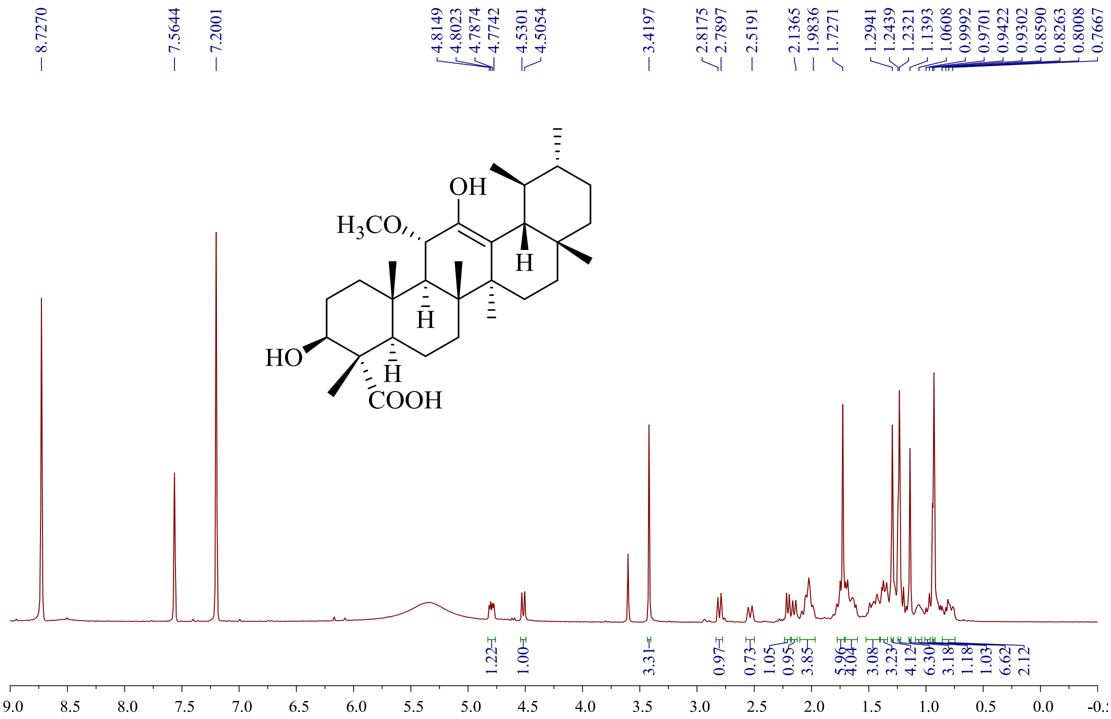

Fig. S63. ^{1}H NMR spectrum of compound **7**-expansion.

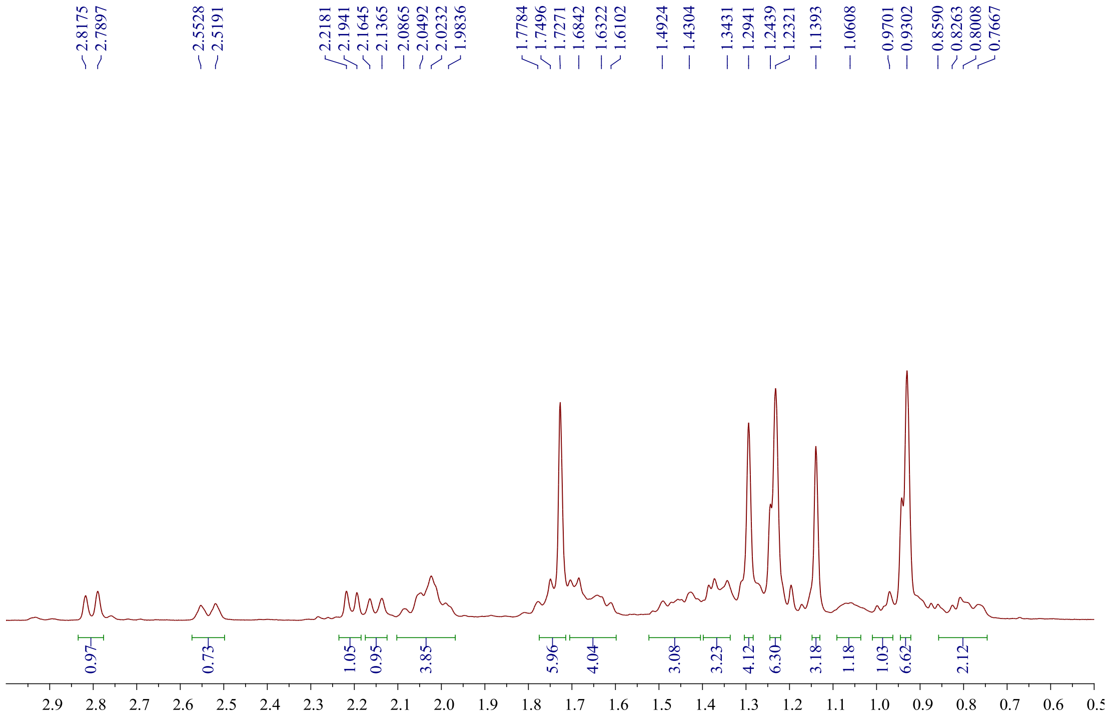

Fig. S64. ^{13}C NMR spectrum of compound **7** in C_5D_5N (150 MHz).

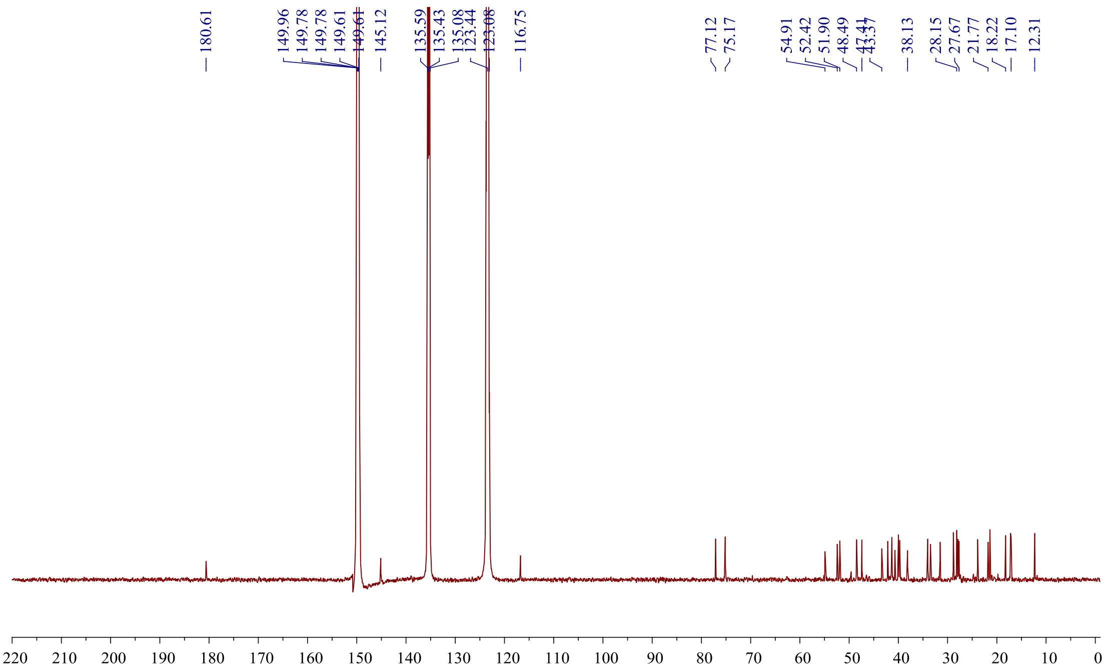

Fig. S65. ^{13}C NMR spectrum of compound **7** –expansion.

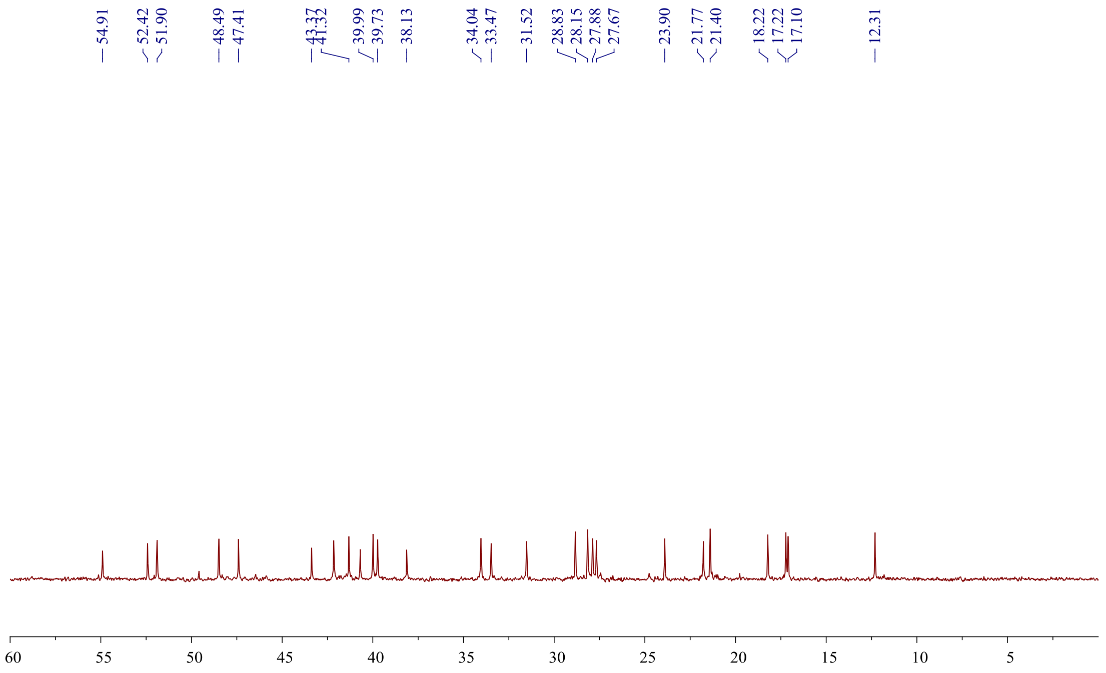

Fig. S66. HSQC spectrum of compound **7** in C_5D_5N (600 MHz).

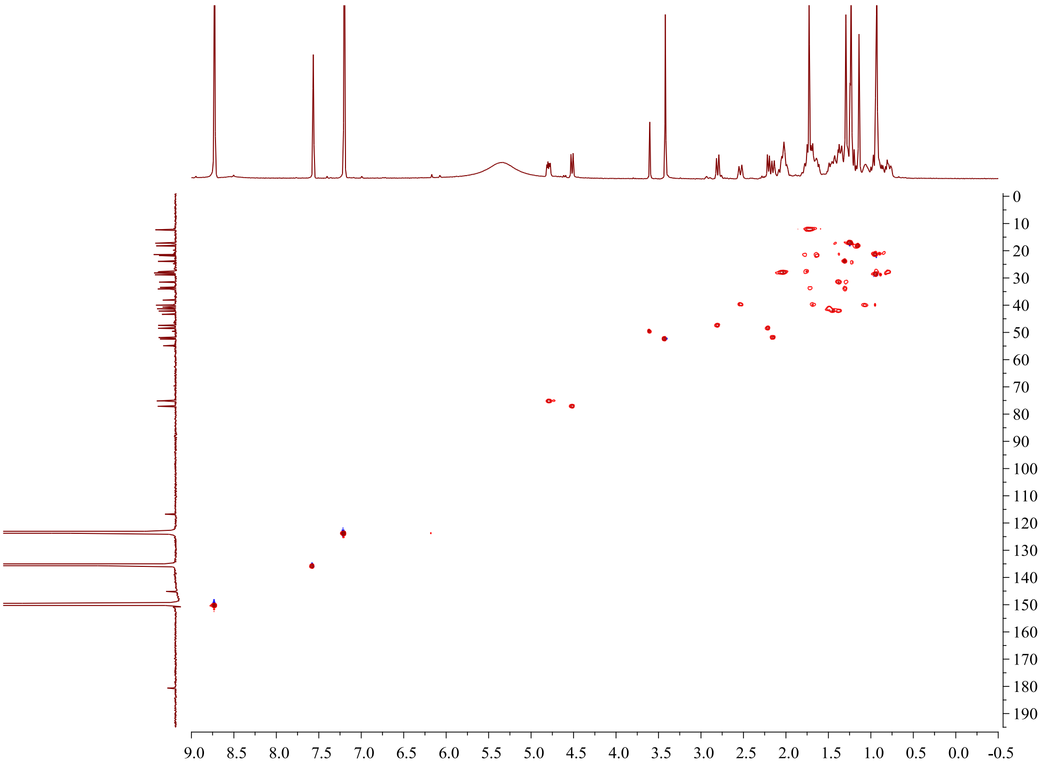

Fig. S67. HMBC spectrum of compound **7** in C_5D_5N (600 MHz).

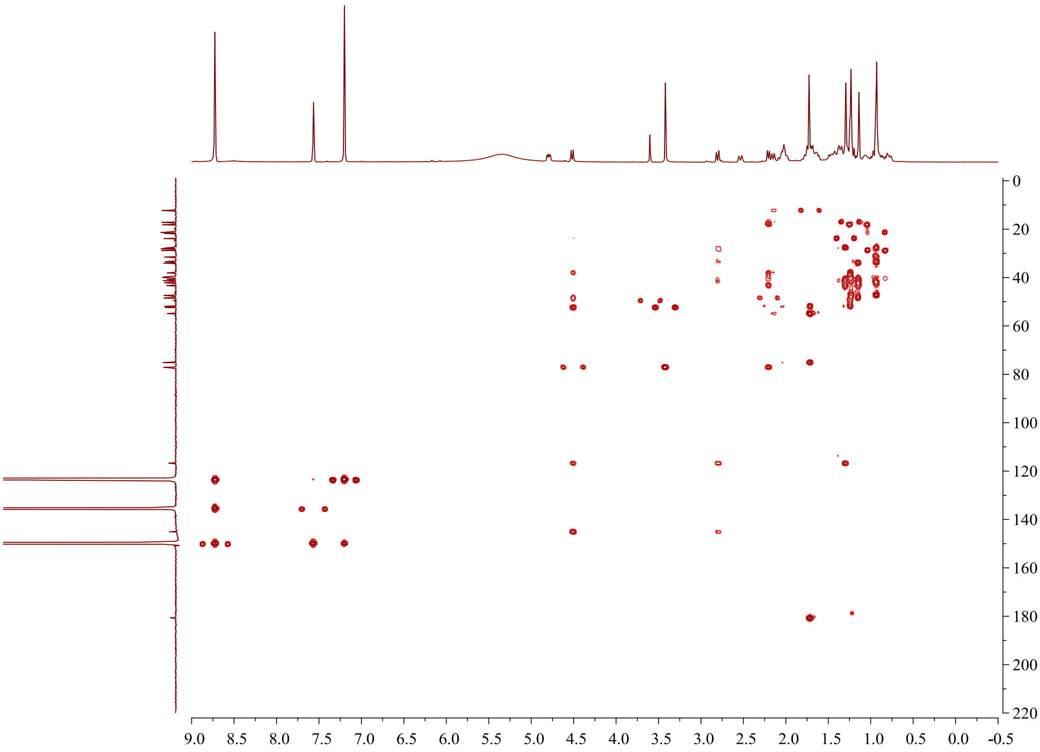

Fig. S68. ROESY spectrum of compound **7** in C_5D_5N (600 MHz).

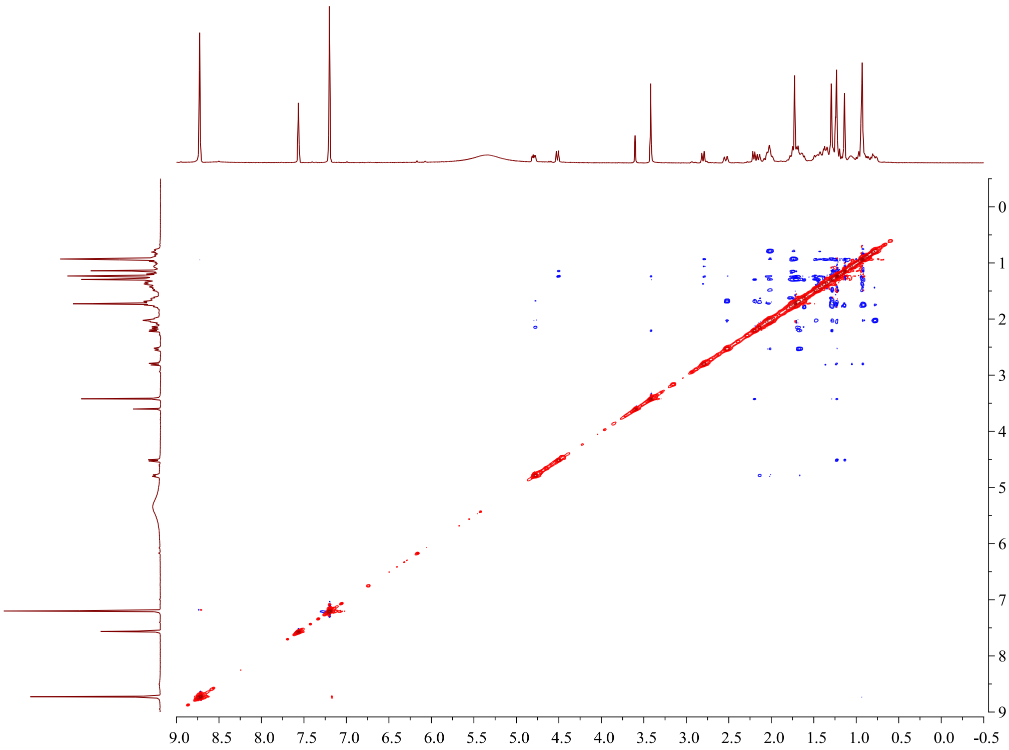

Fig. S69. ROESY spectrum of compound **7**-expansion.

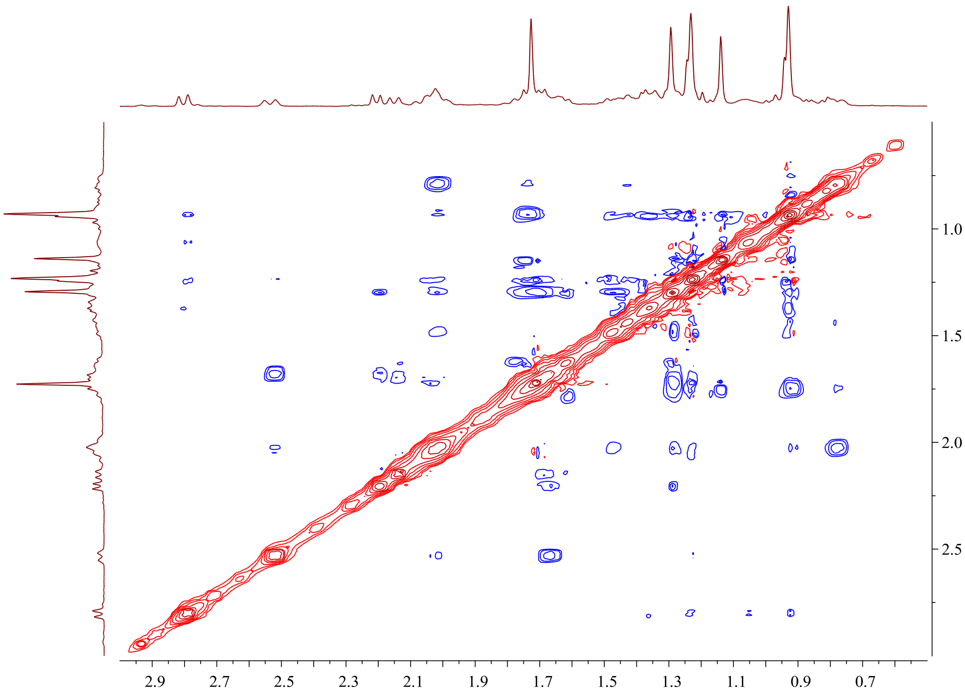

Fig. S70. HRESIMS report of compound **7**.

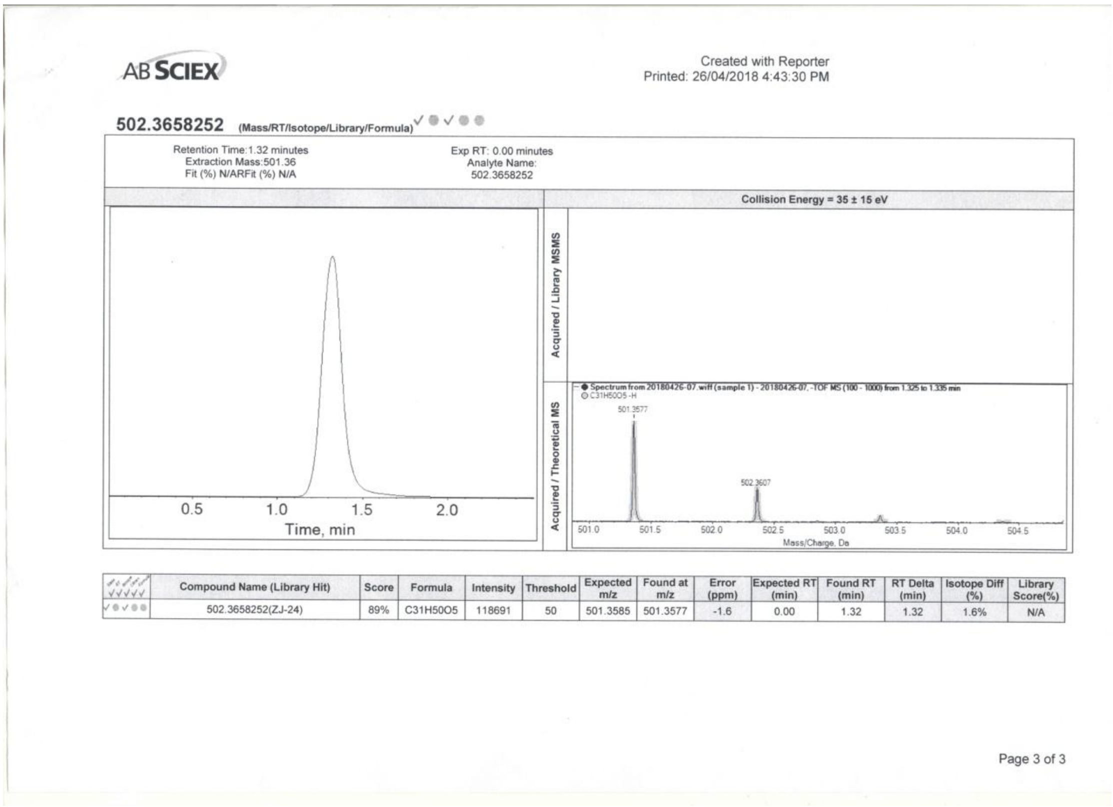

AB SCIEX

Created with Reporter
Printed: 26/04/2018 4:43:30 PM

502.3658252 (Mass/RT/Isotope/Library/Formula)

Retention Time:1.32 minutes
Extraction Mass:501.36
Fit (%) N/ARFit (%) N/A

Exp RT: 0.00 minutes
Analyte Name:
502.3658252

Collision Energy = 35 ± 15 eV

	Compound Name (Library Hit)	Score	Formula	Intensity	Threshold	Expected m/z	Found at m/z	Error (ppm)	Expected RT (min)	Found RT (min)	RT Delta (min)	Isotope Diff (%)	Library Score(%)
	502.3658252(ZJ-24)	89%	C31H50O5	118691	50	501.3585	501.3577	-1.6	0.00	1.32	1.32	1.6%	N/A

Page 3 of 3

Fig. S71. Experimental ECD spectra of **6** (red curve) and **7** (black curve) in MeOH.

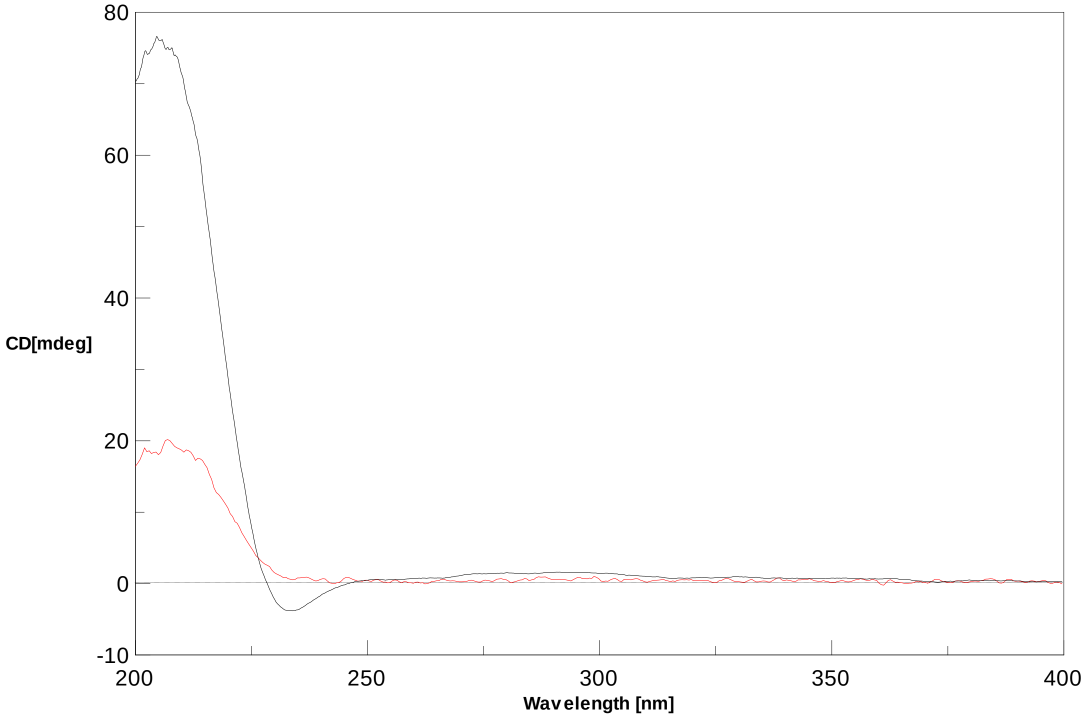

Fig. S72. ^{1}H NMR spectrum of compound **8** in CD3OD (400 MHz).

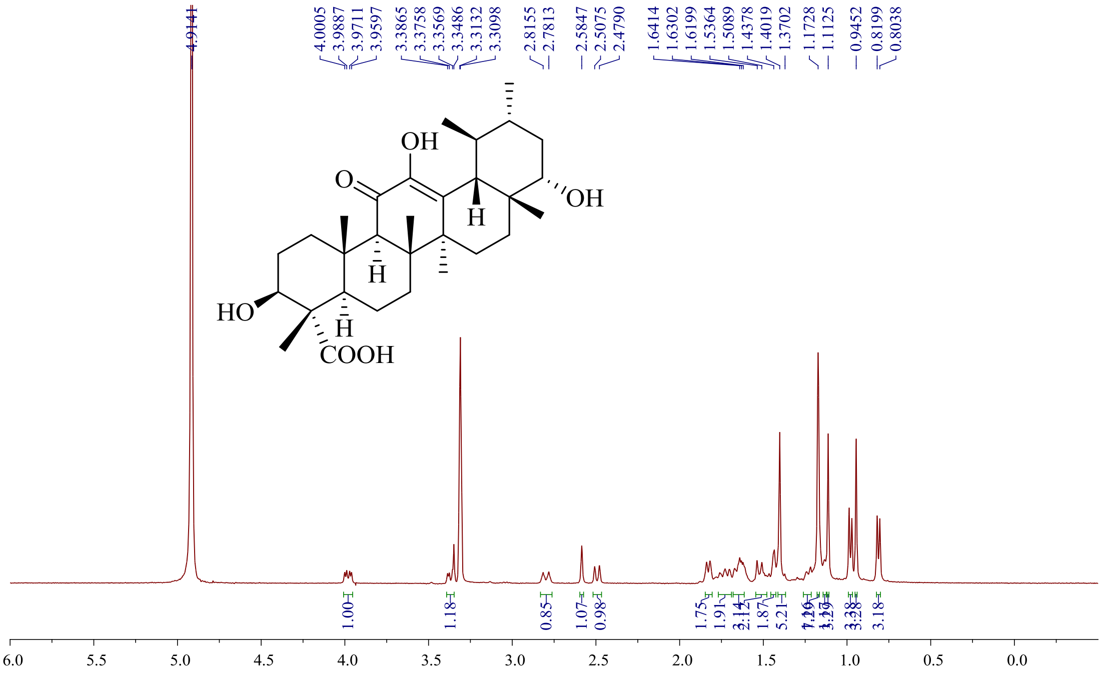

Fig. S73. ^{1}H NMR spectrum of compound **8**-expansion.

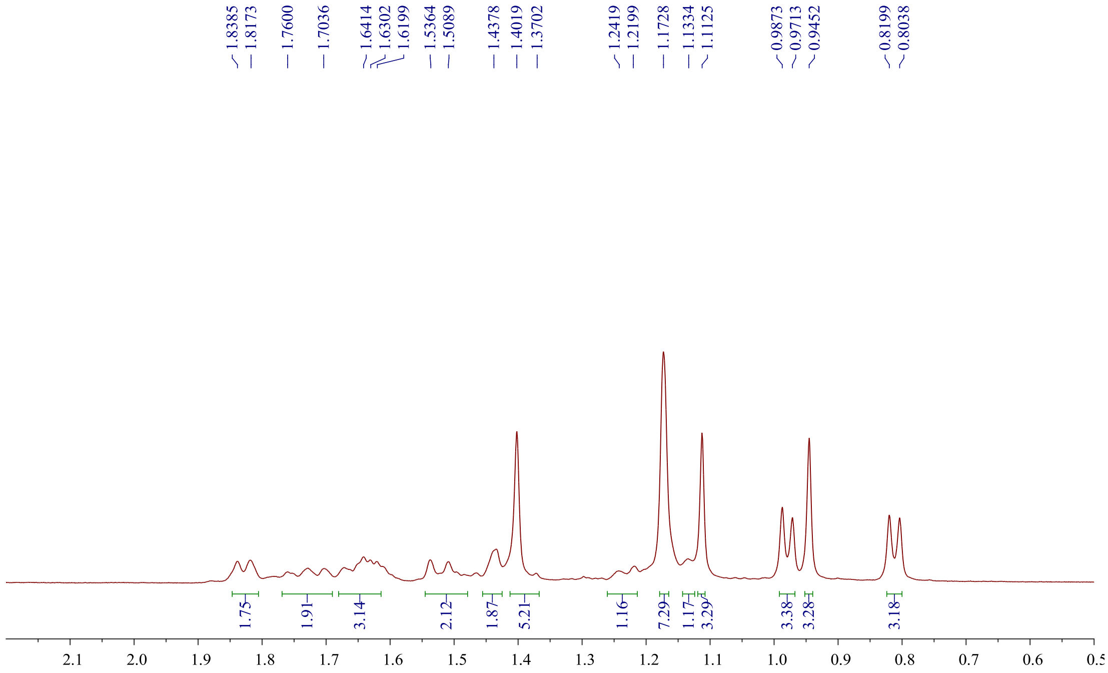

Fig. S74. ^{13}C NMR spectrum of compound **8** in CD_3OD (150 MHz).

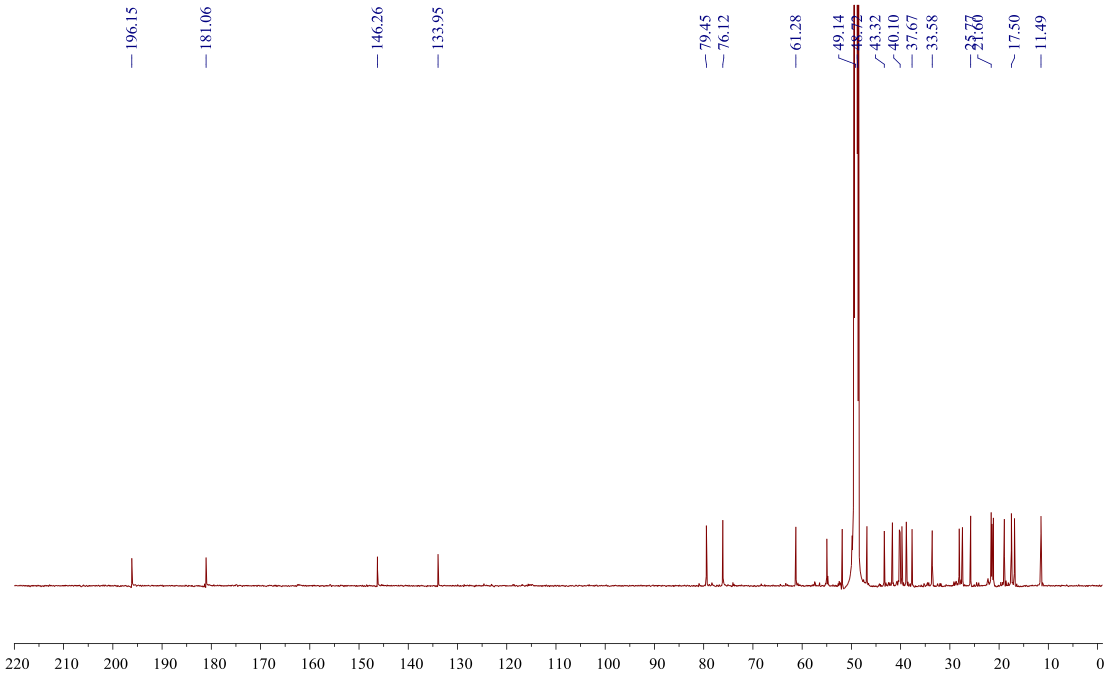

Fig. S75. ^{13}C NMR spectrum of compound **8**-expansion.

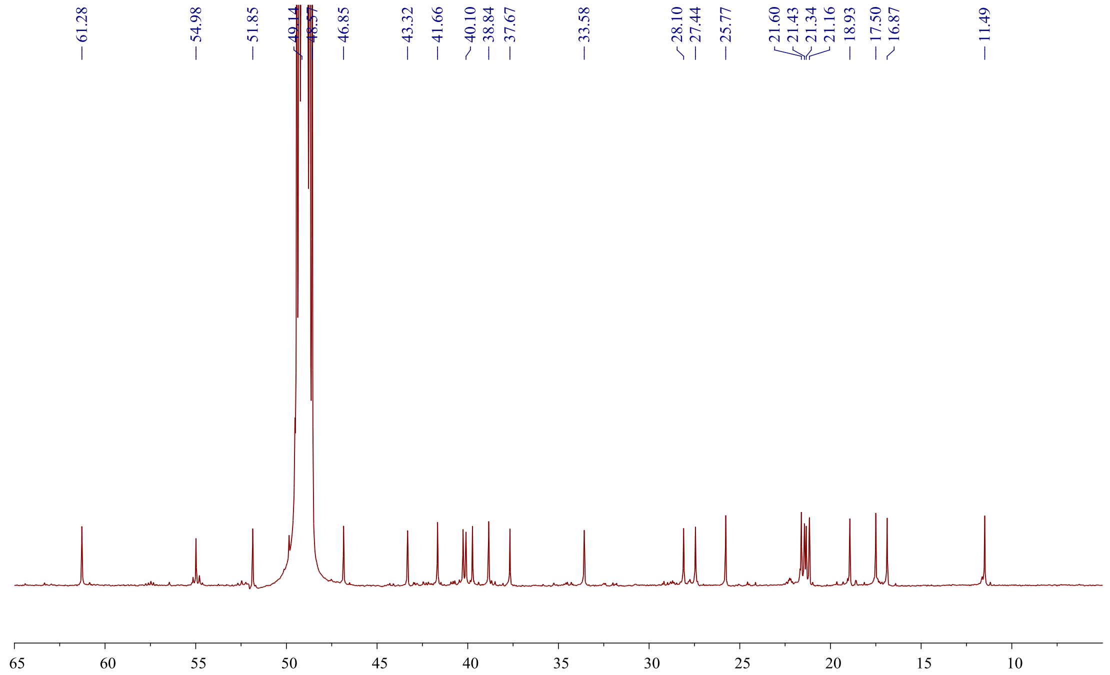

Fig. S76. ^{1}H-^{1}H COSY spectrum of compound **8** in CD3OD (600 MHz).

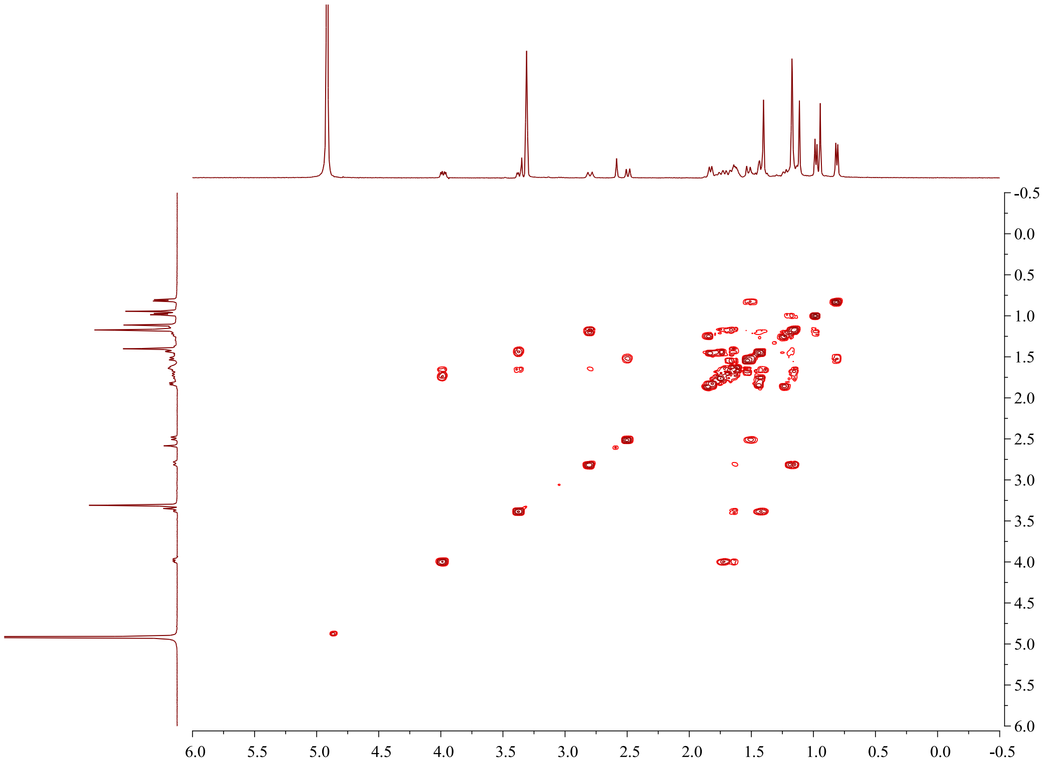

Fig. S77. HMBC spectrum of compound **8** in CD3OD (600 MHz).

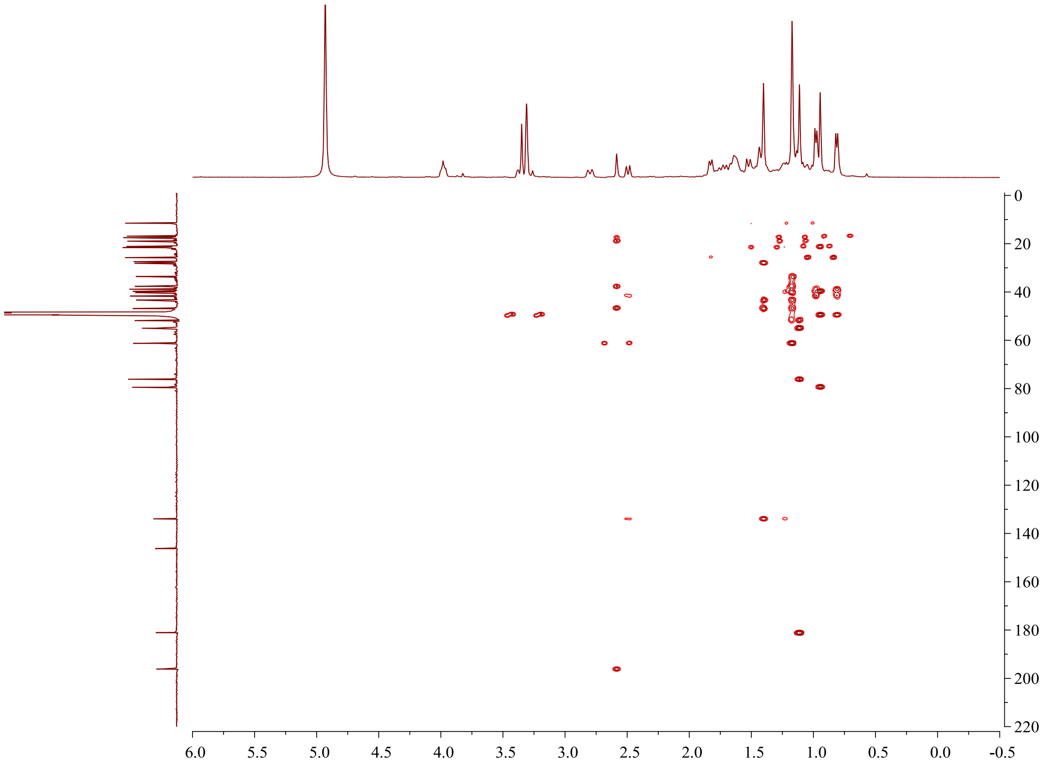

Fig. S78. HMBC spectrum of compound **8**–expansion.

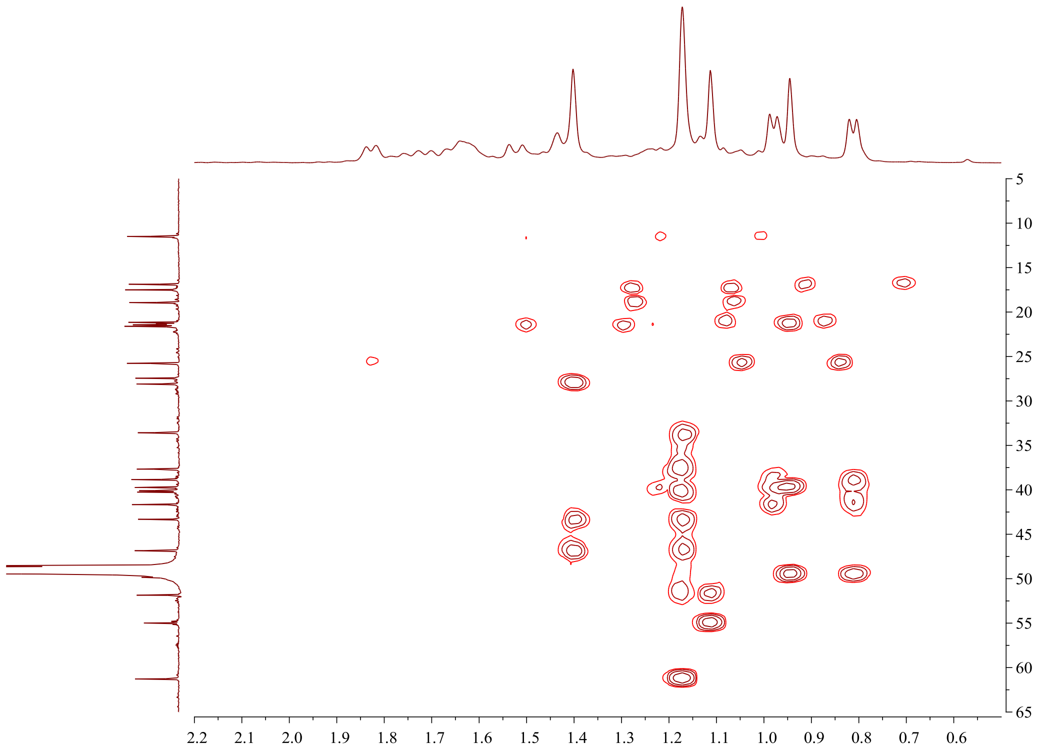

Fig. S79. ^{1}H NMR spectrum of compound **8** in C_5D_5N (600 MHz).

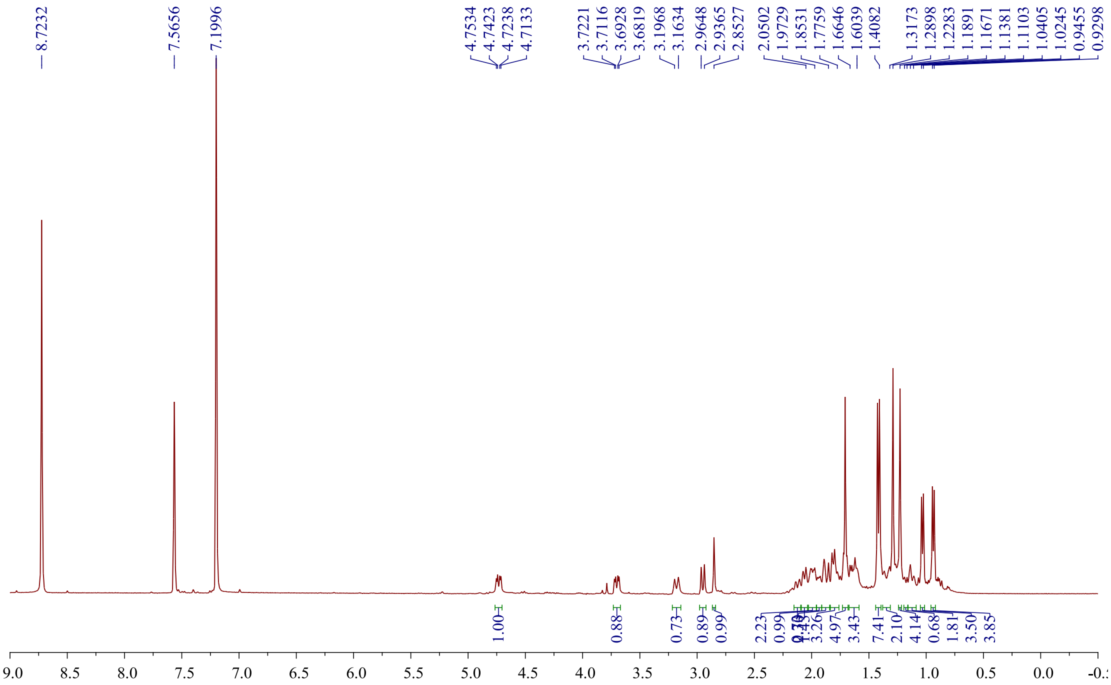

Fig. S80. ROESY spectrum of compound **8** in C_5D_5N (600 MHz).

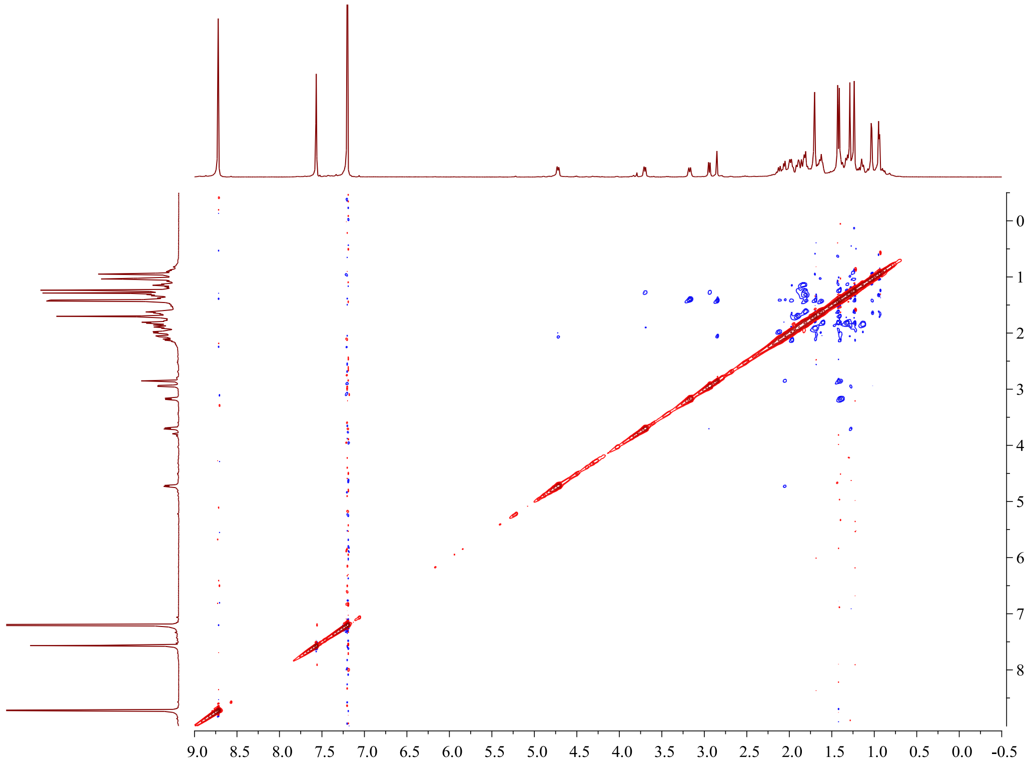

Fig. S81. HRESIMS report of compound **8**.

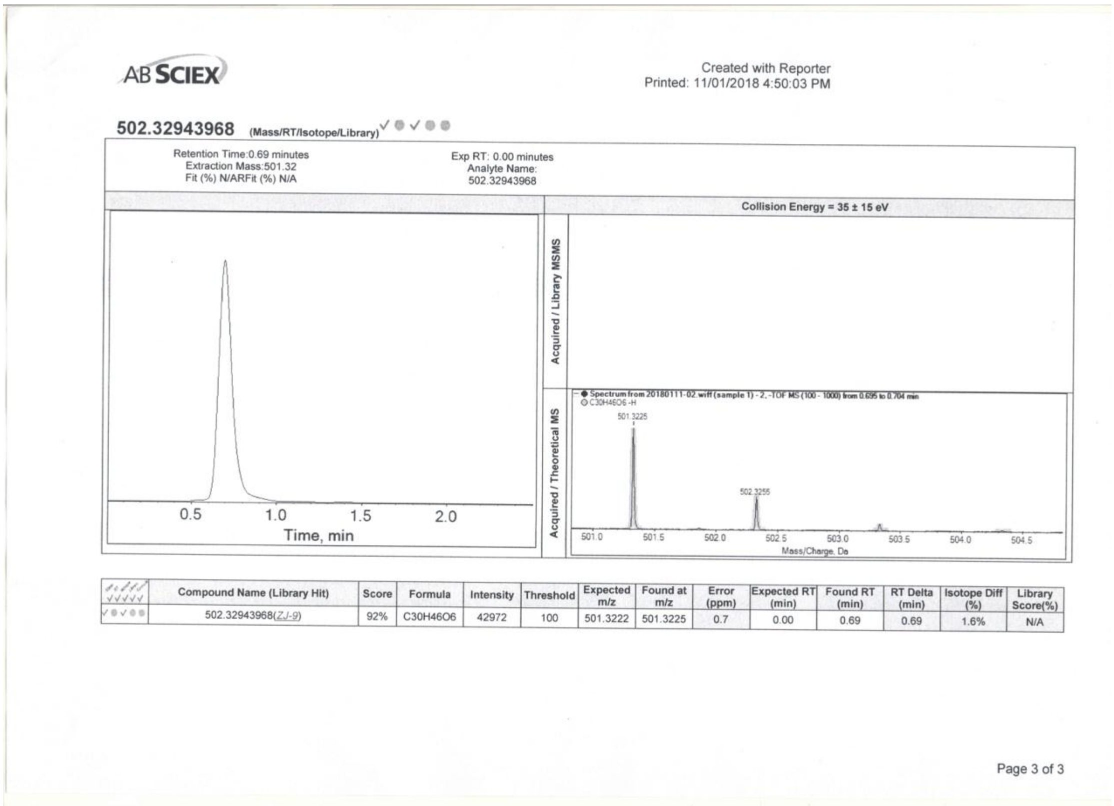
AB SCIEX

Created with Reporter
Printed: 11/01/2018 4:50:03 PM

502.32943968 (Mass/RT/Isotope/Library)

Retention Time:0.69 minutes
Extraction Mass:501.32
Fit (%) N/ARFit (%) N/A

Exp RT: 0.00 minutes
Analyte Name:
502.32943968

Collision Energy = 35 ± 15 eV

	Compound Name (Library Hit)	Score	Formula	Intensity	Threshold	Expected m/z	Found at m/z	Error (ppm)	Expected RT (min)	Found RT (min)	RT Delta (min)	Isotope Diff (%)	Library Score(%)
	502.32943968(ZJ-9)	92%	C30H46O6	42972	100	501.3222	501.3225	0.7	0.00	0.69	0.69	1.6%	N/A

Page 3 of 3

Fig. S82. ^{1}H NMR spectrum of compound **9** in CD_3OD (400 MHz).

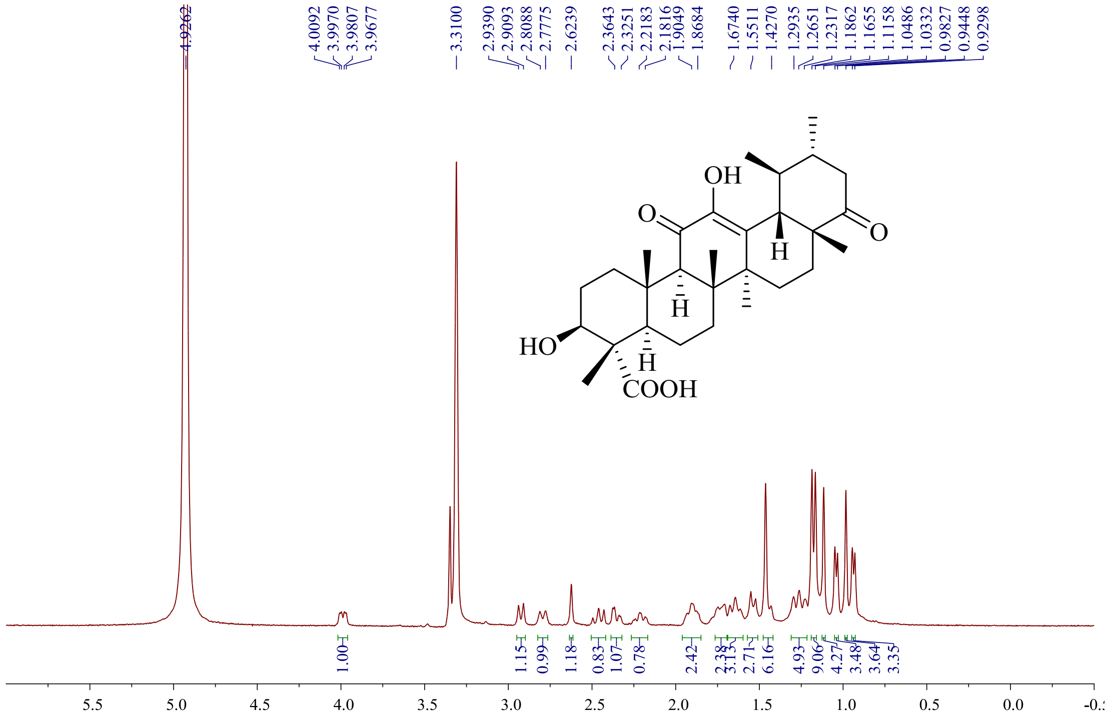

Fig. S83. ^{1}H NMR spectrum of compound **9**-expansion.

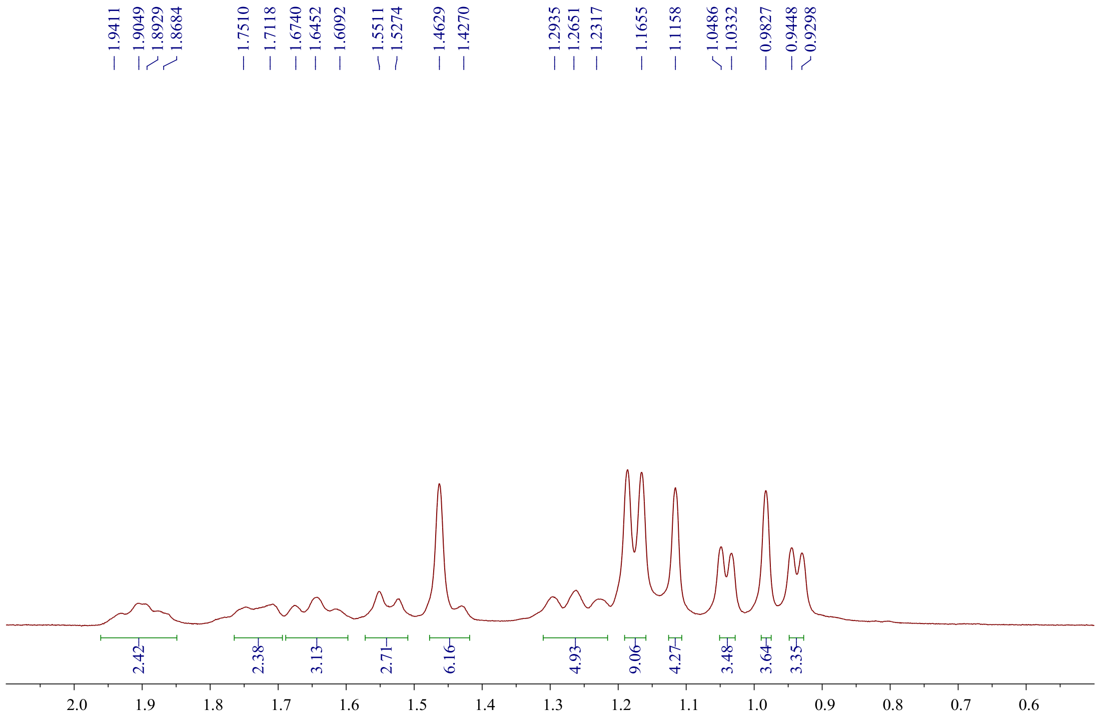

Fig. S84. ^{13}C NMR spectrum of compound **9** in CD3OD (150 MHz).

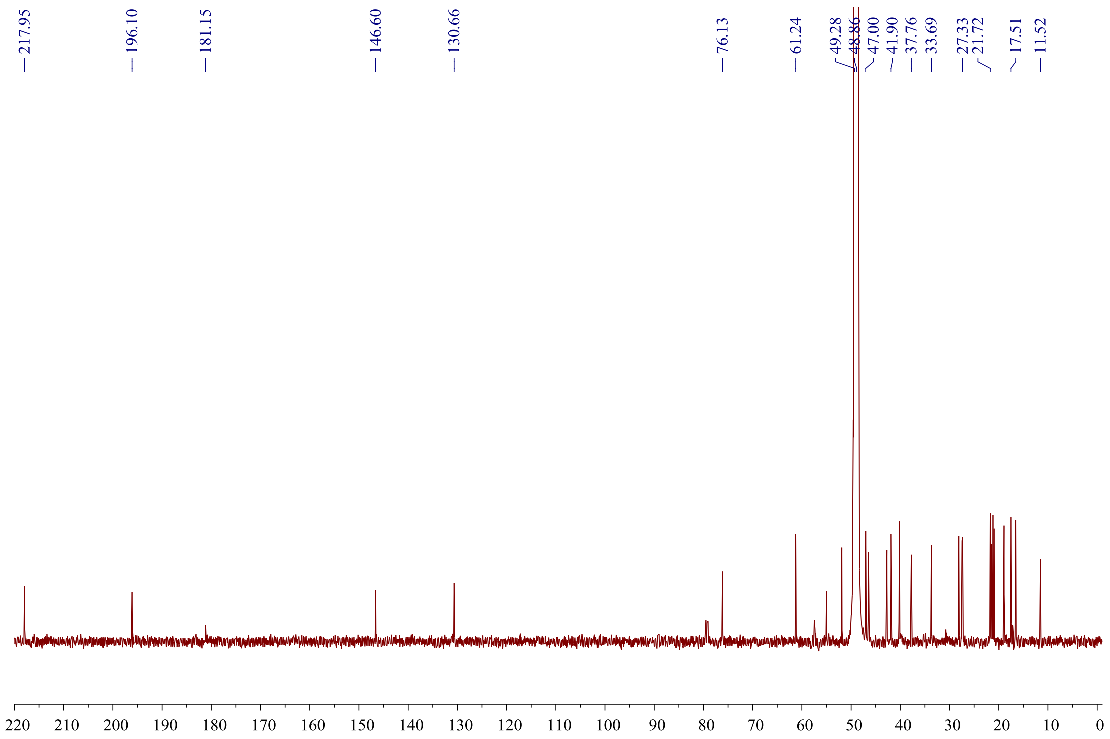

Fig. S85. ^{13}C NMR spectrum of compound **9**-expansion.

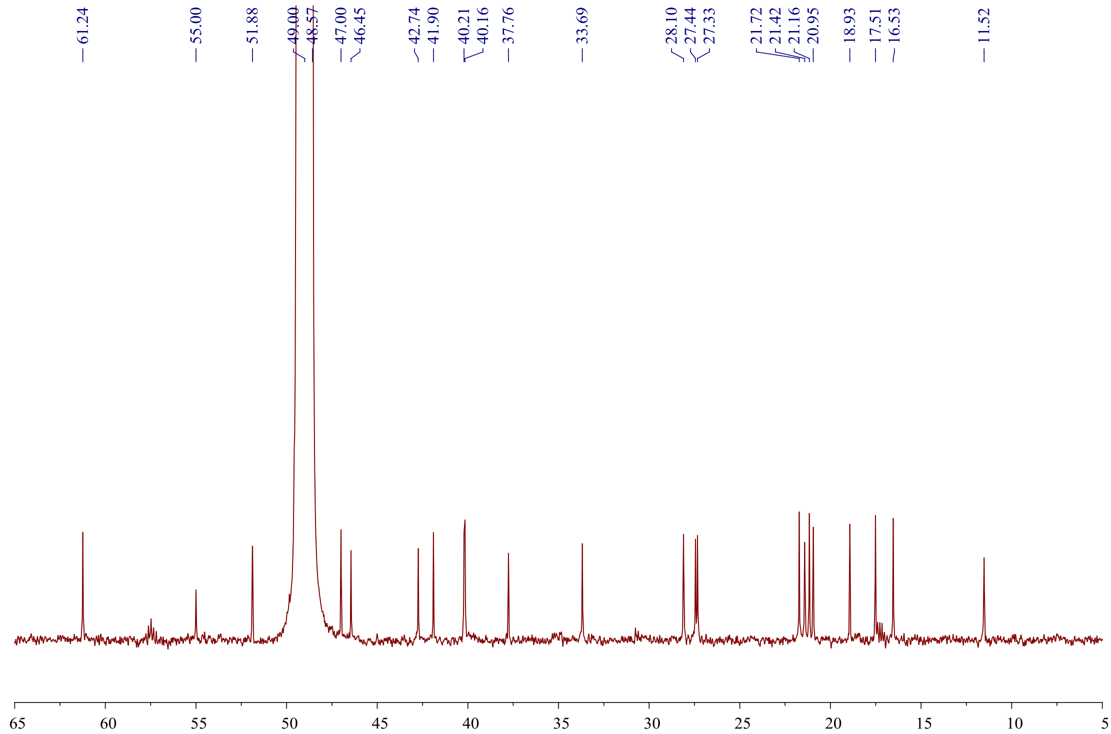

Fig. S86. HMBC spectrum of compound **9** in CD_3OD (600 MHz).

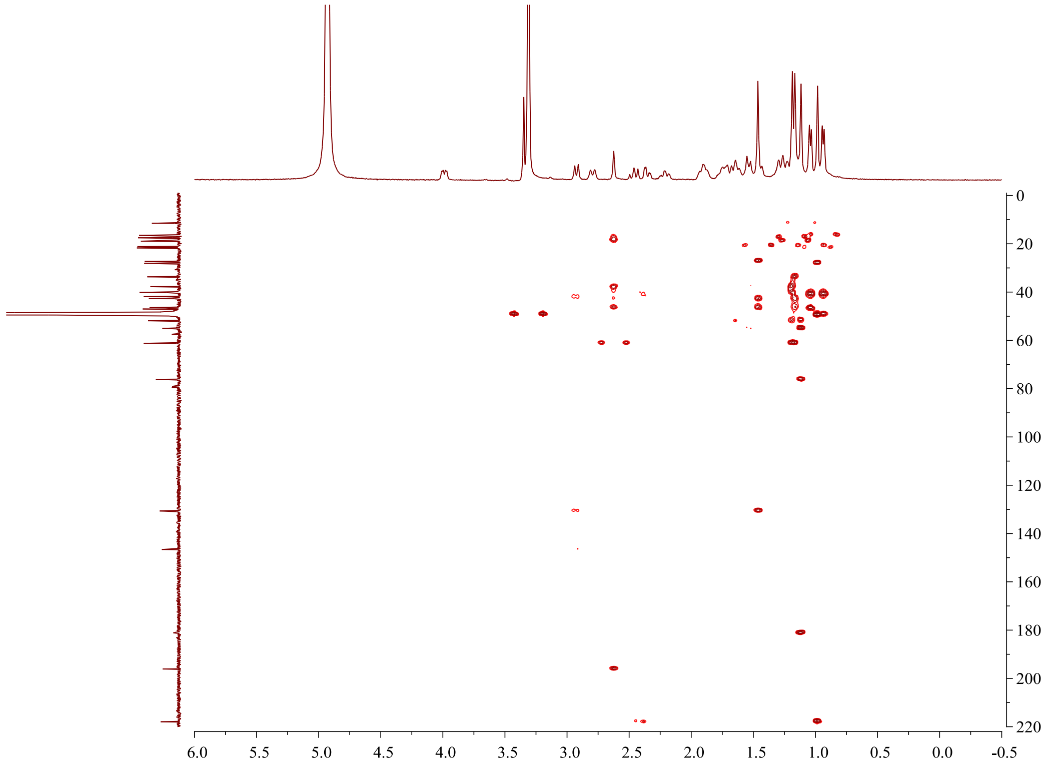

Fig. S87. HMBC spectrum of compound **9**-expansion.

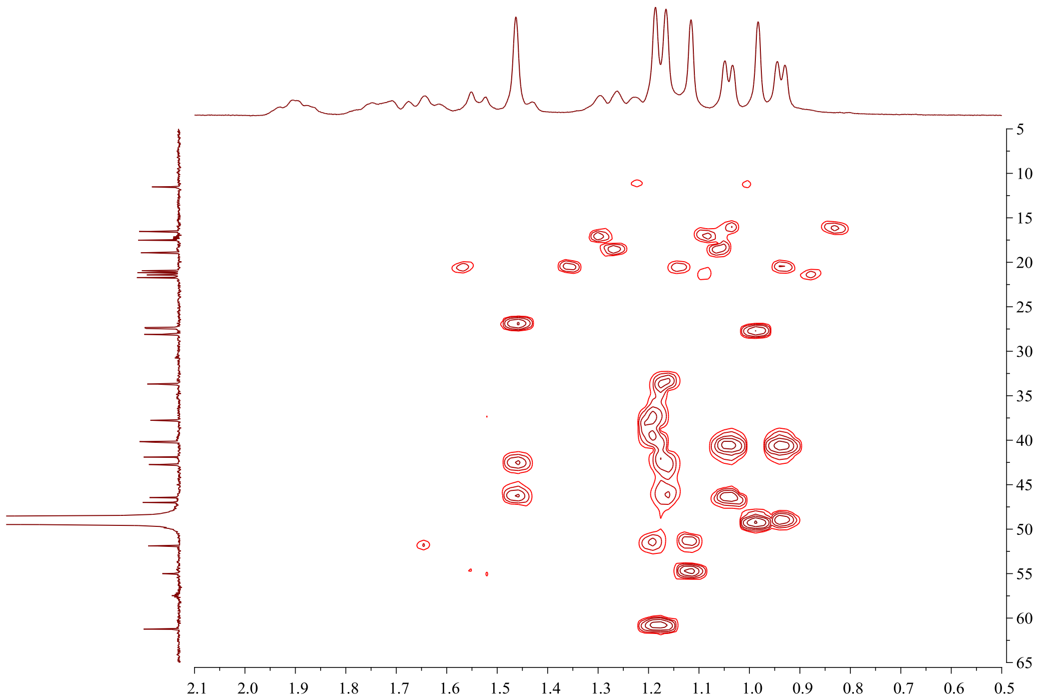

Fig. S88. ROESY spectrum of compound **9** in CD_3OD (600 MHz).

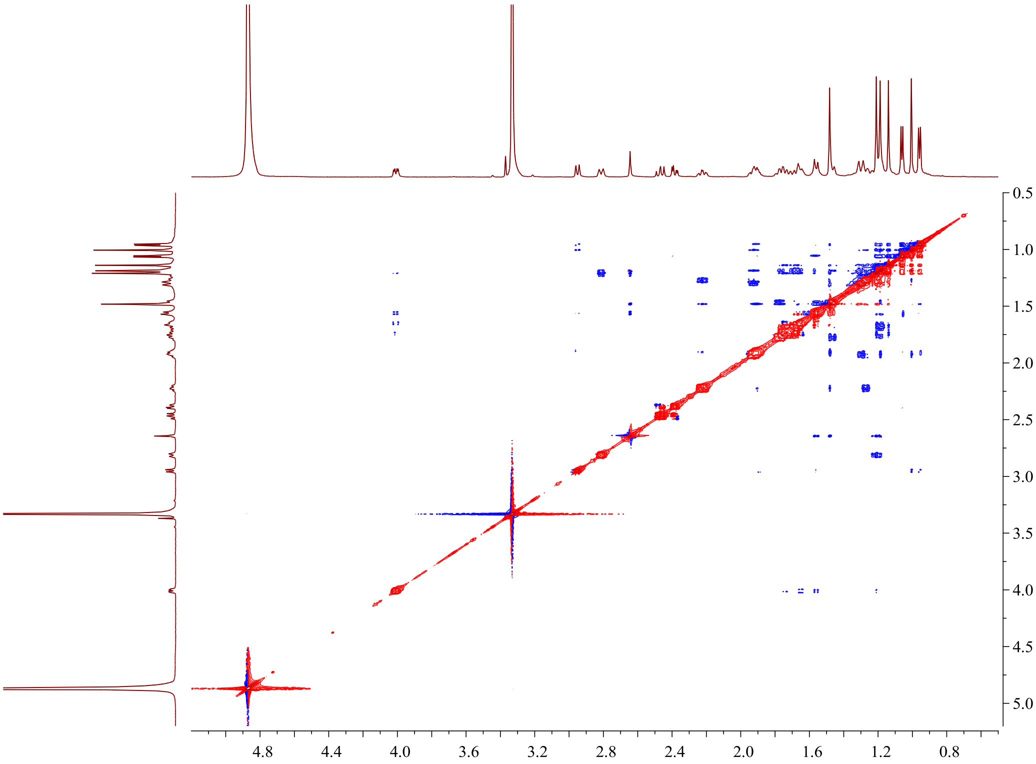

Fig. S89. HRESIMS report of compound **9**.

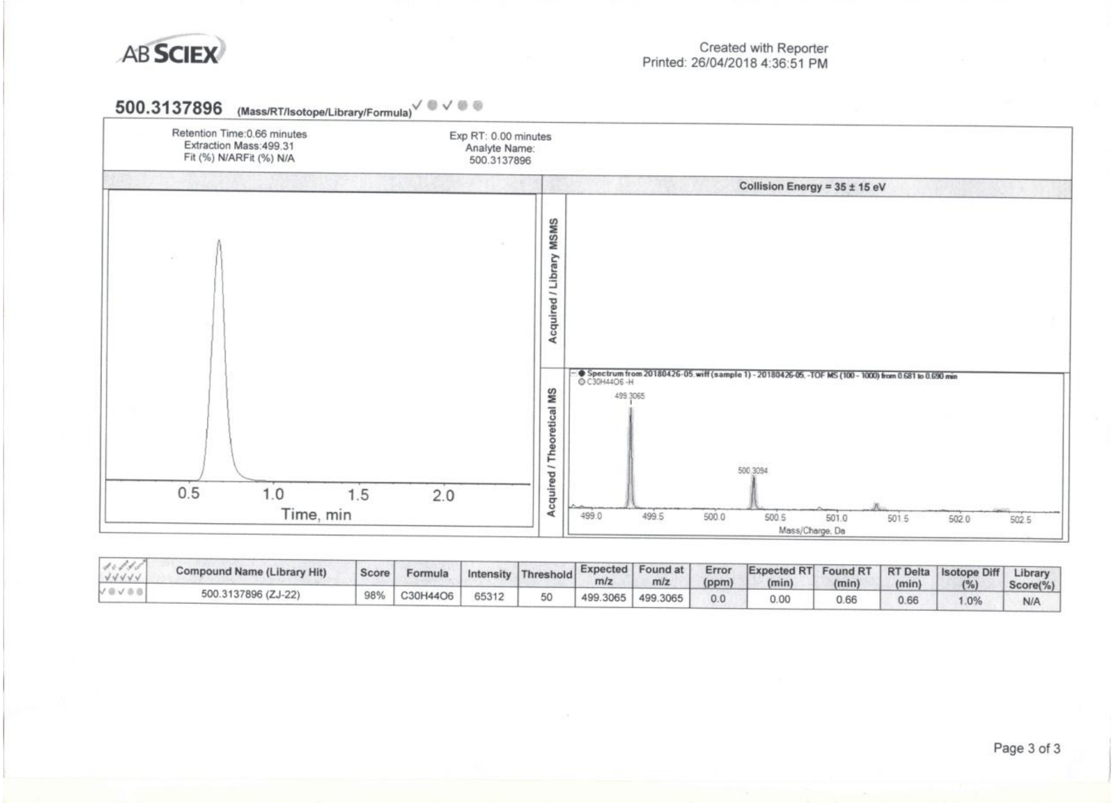

AB SCIEX

Created with Reporter
Printed: 26/04/2018 4:36:51 PM

500.3137896 (Mass/RT/Isotope/Library/Formula)

Retention Time:0.66 minutes
Extraction Mass:499.31
Fit (%) N/ARFit (%) N/A

Exp RT: 0.00 minutes
Analyte Name:
500.3137896

Collision Energy = 35 ± 15 eV

	Compound Name (Library Hit)	Score	Formula	Intensity	Threshold	Expected m/z	Found at m/z	Error (ppm)	Expected RT (min)	Found RT (min)	RT Delta (min)	Isotope Diff (%)	Library Score(%)
	500.3137896 (ZJ-22)	98%	C30H44O6	65312	50	499.3065	499.3065	0.0	0.00	0.66	0.66	1.0%	N/A

Page 3 of 3

Fig. S90. ^{1}H NMR spectrum of compound **10** in CD3OD (400 MHz).

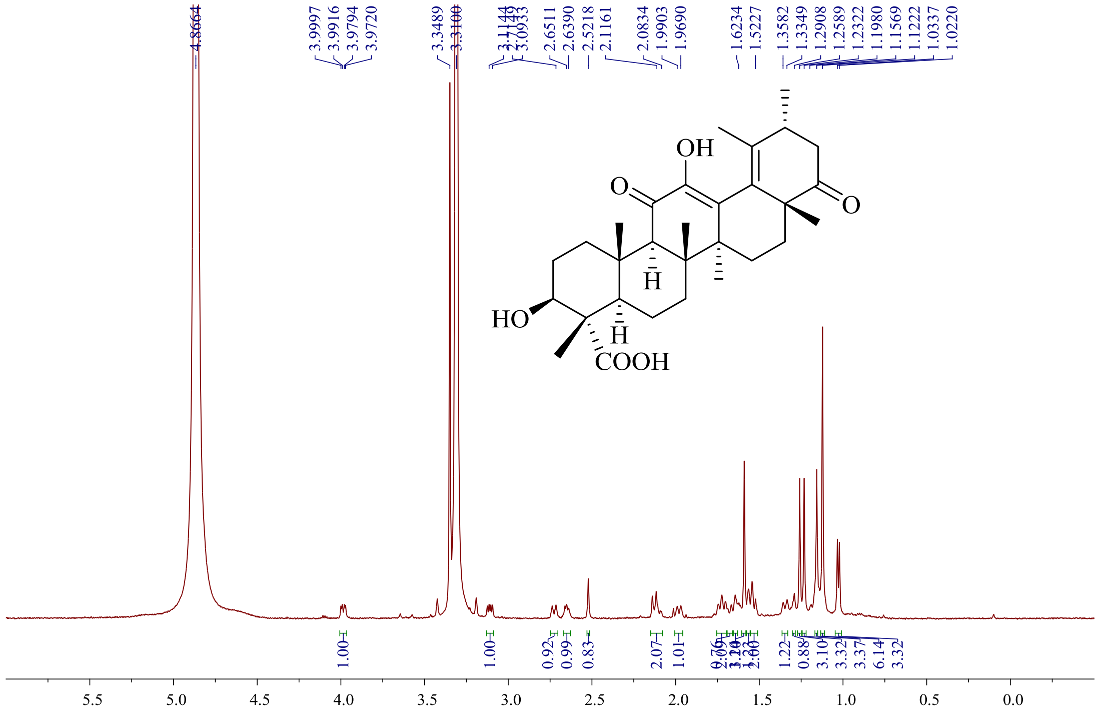

Fig. S91. ^{1}H NMR spectrum of compound **10**-expansion.

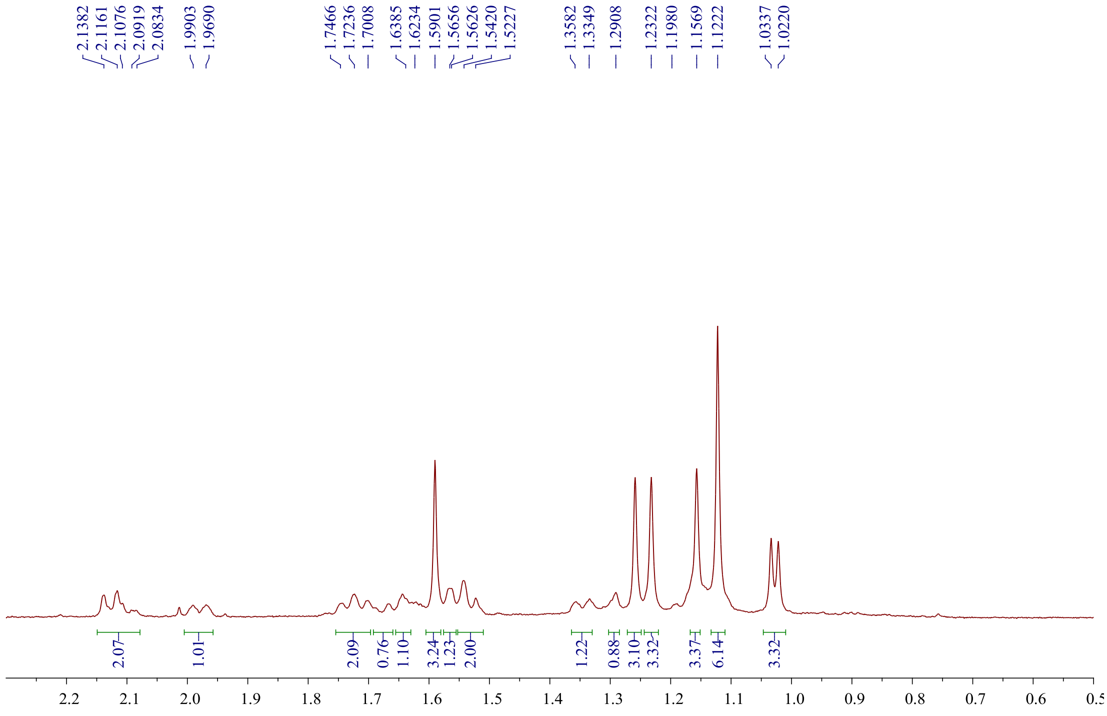

Fig. S92. ^{13}C NMR spectra of compound **10** in CD_3OD (150 MHz).

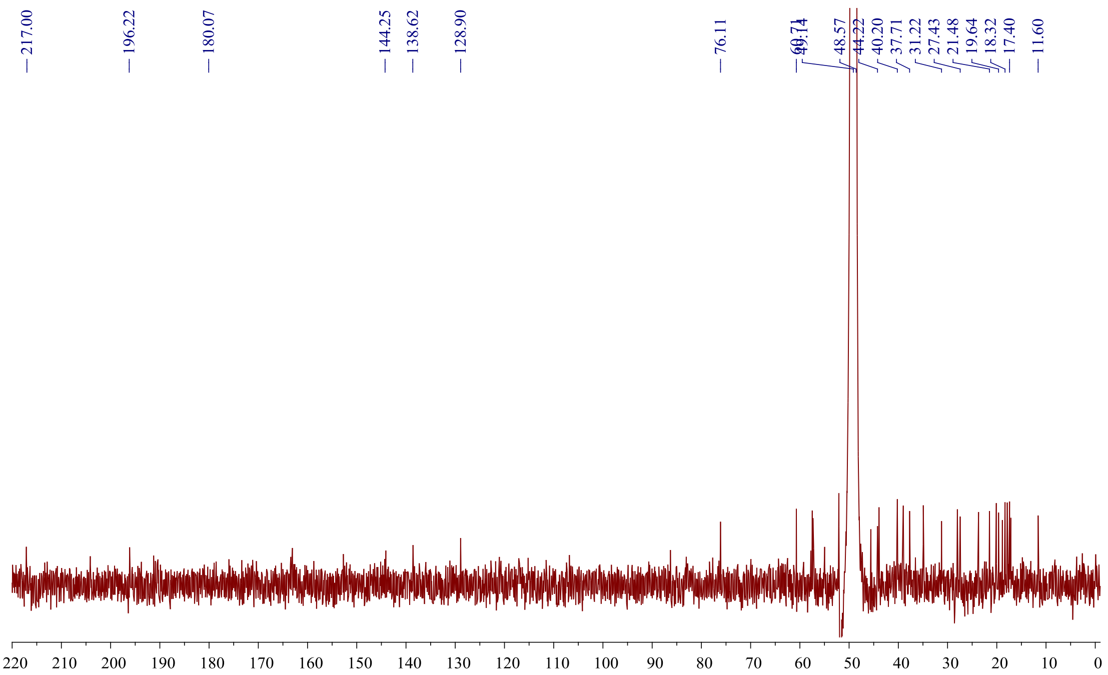

Fig. S93. HSQC spectrum of compound **10** in CD_3OD (600 MHz).

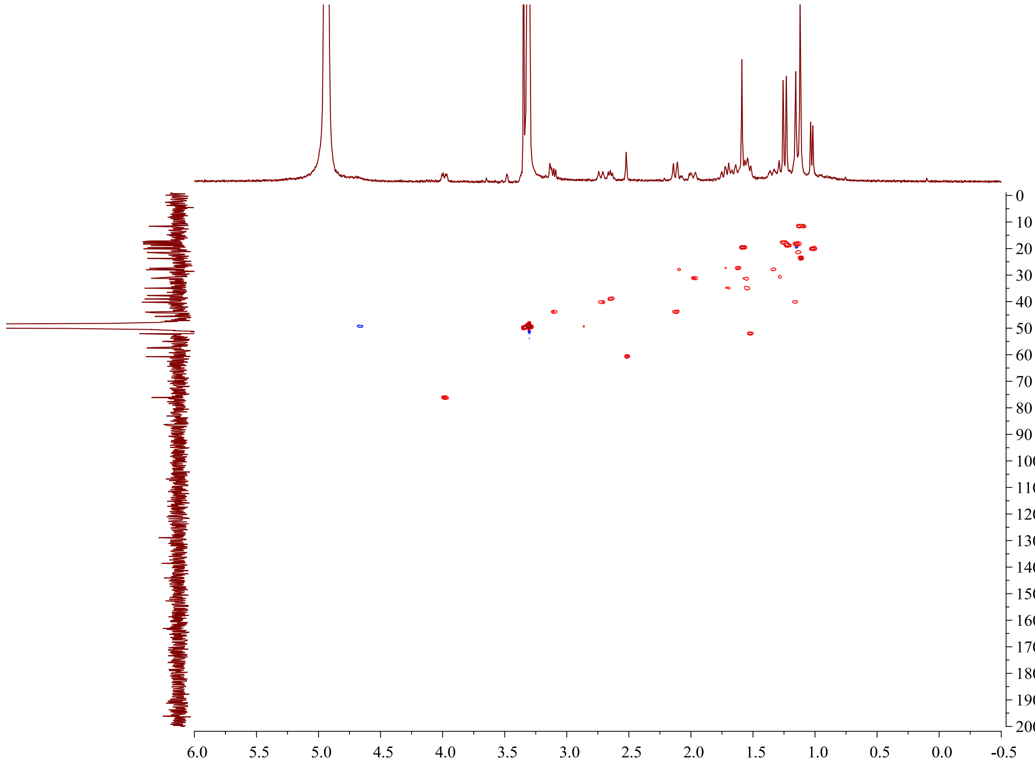

Fig. S94. HMBC spectrum of compound **10** in CD3OD (600 MHz).

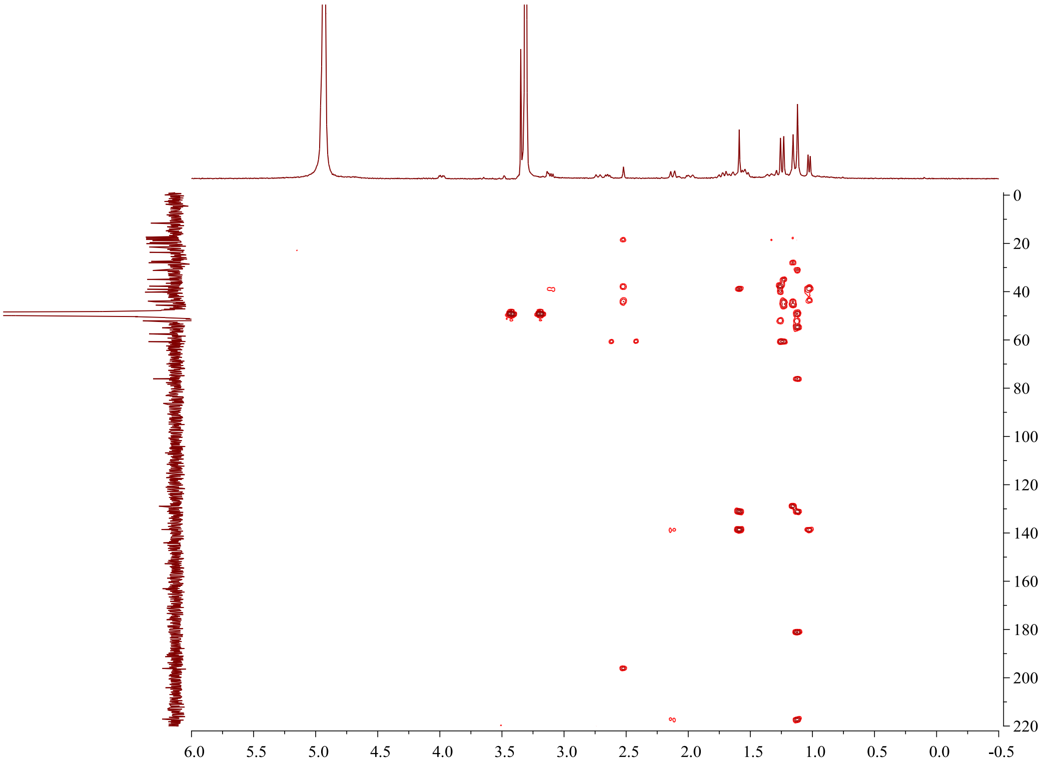

Fig. S95. HMBC spectrum of compound **10**–expansion.

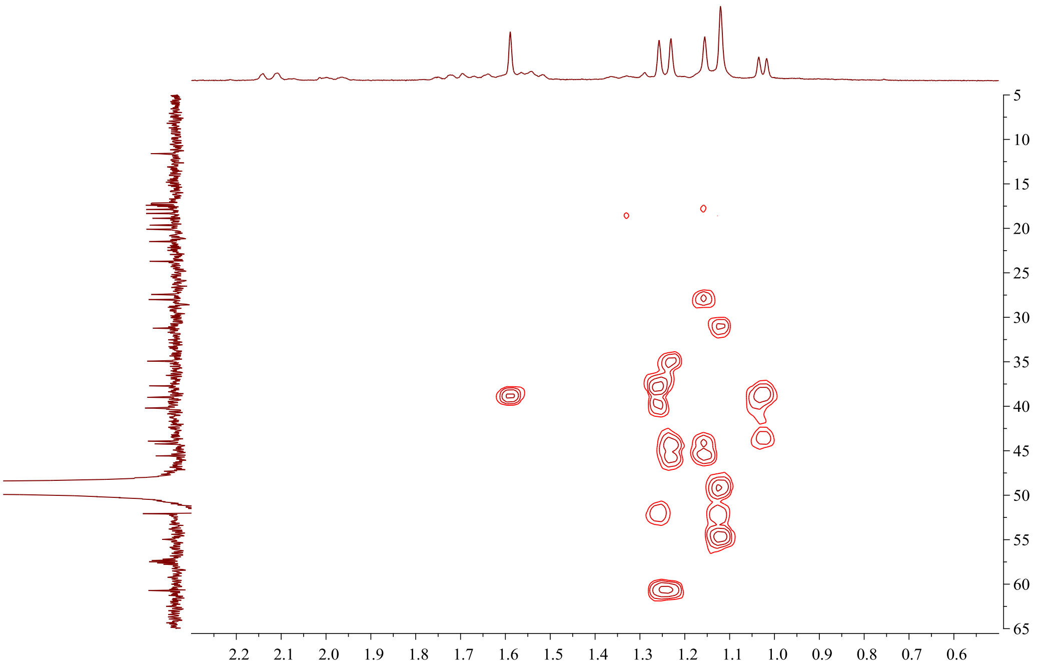

Fig. S96. ROESY spectrum of compound **10** in CD_3OD (600 MHz).

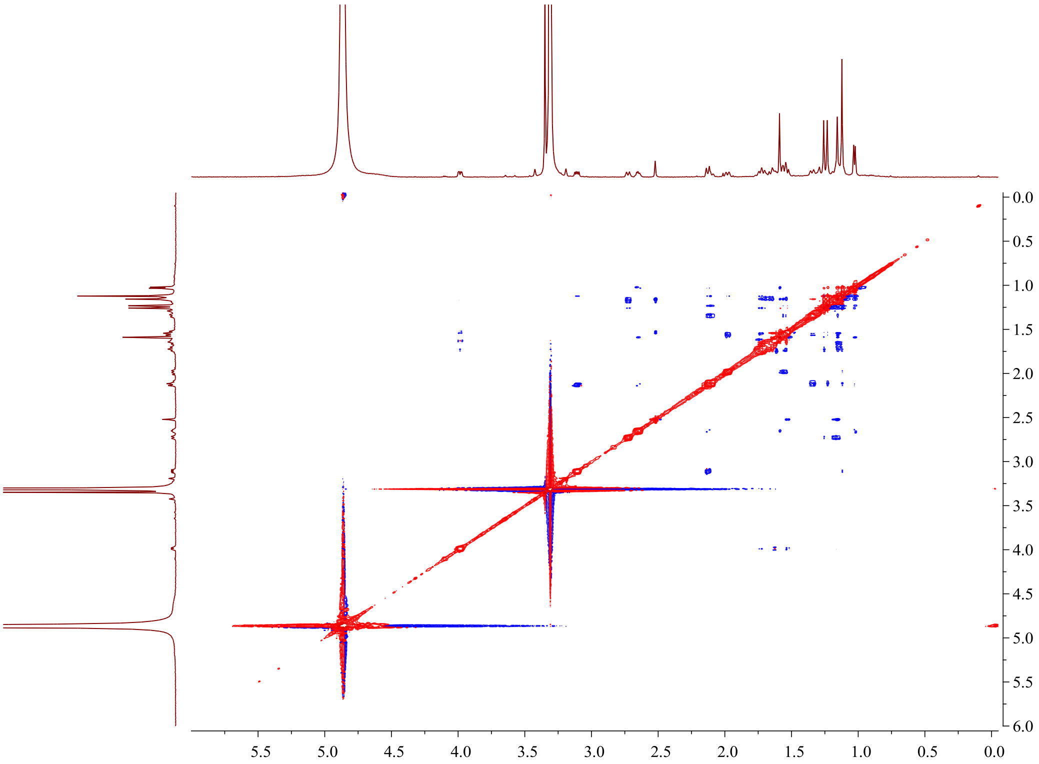

Fig. S97. HRESIMS report of compound **10**.

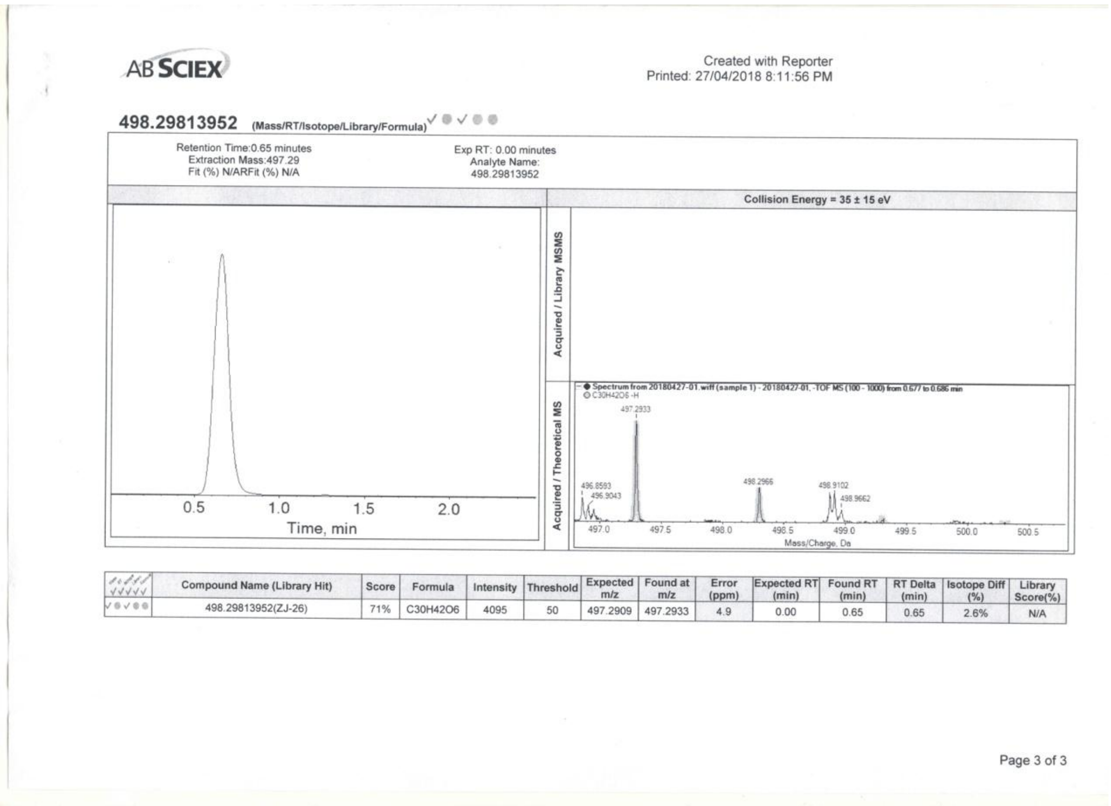

	Compound Name (Library Hit)	Score	Formula	Intensity	Threshold	Expected m/z	Found at m/z	Error (ppm)	Expected RT (min)	Found RT (min)	RT Delta (min)	Isotope Diff (%)	Library Score(%)
	498.29813952(ZJ-26)	71%	C30H42O6	4095	50	497.2909	497.2933	4.9	0.00	0.65	0.65	2.6%	N/A

Fig. S98. ^{1}H NMR spectrum of compound **11** in C_5D_5N (400 MHz).

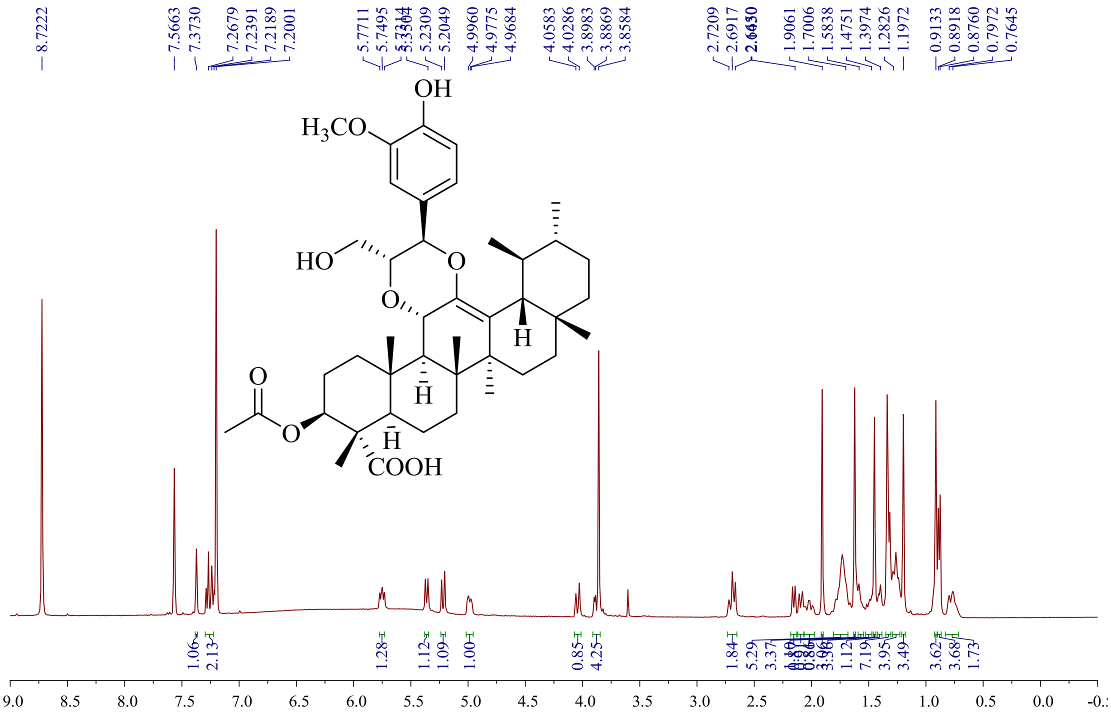

Fig. S99. ^{1}H NMR spectrum of compound **11**-expansion.

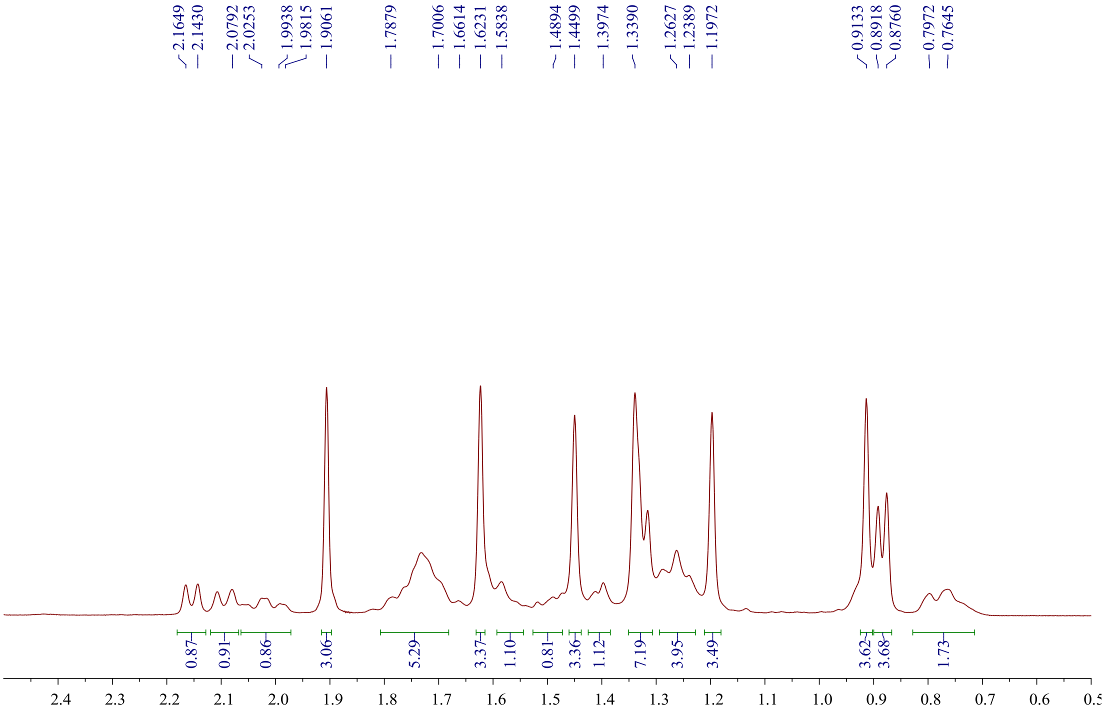

Fig. S100. ^{13}C NMR spectrum of compound **11** in C_5D_5N (150 MHz).

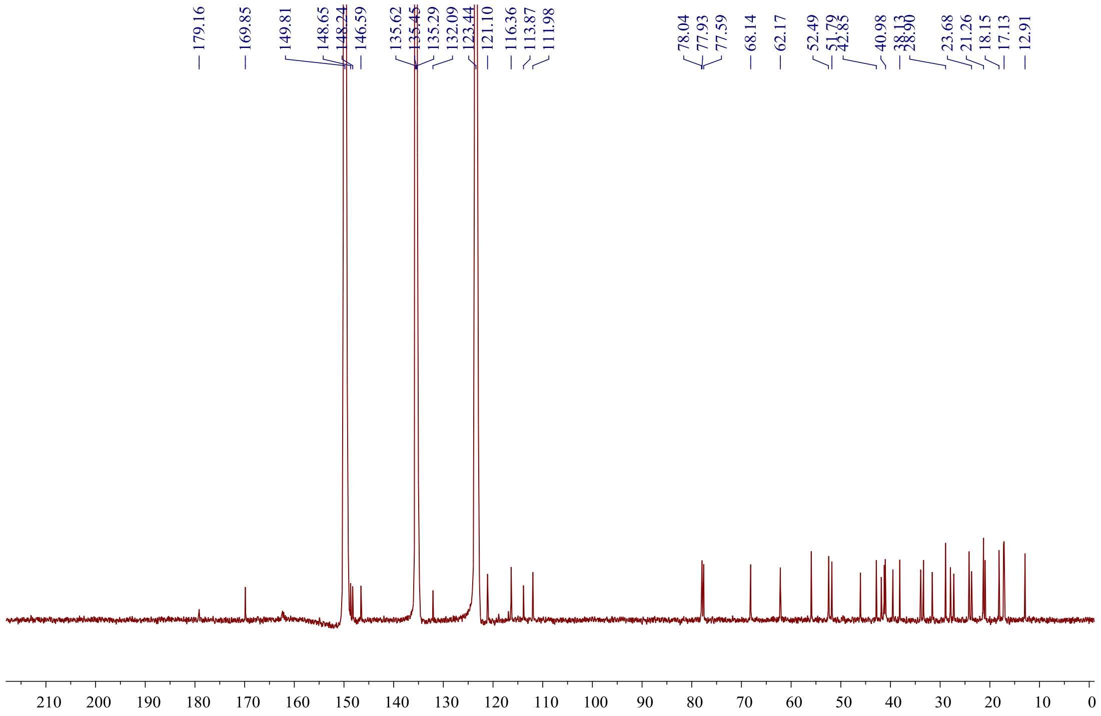

Fig. S101. ^{13}C NMR spectrum of compound **11**-expansion.

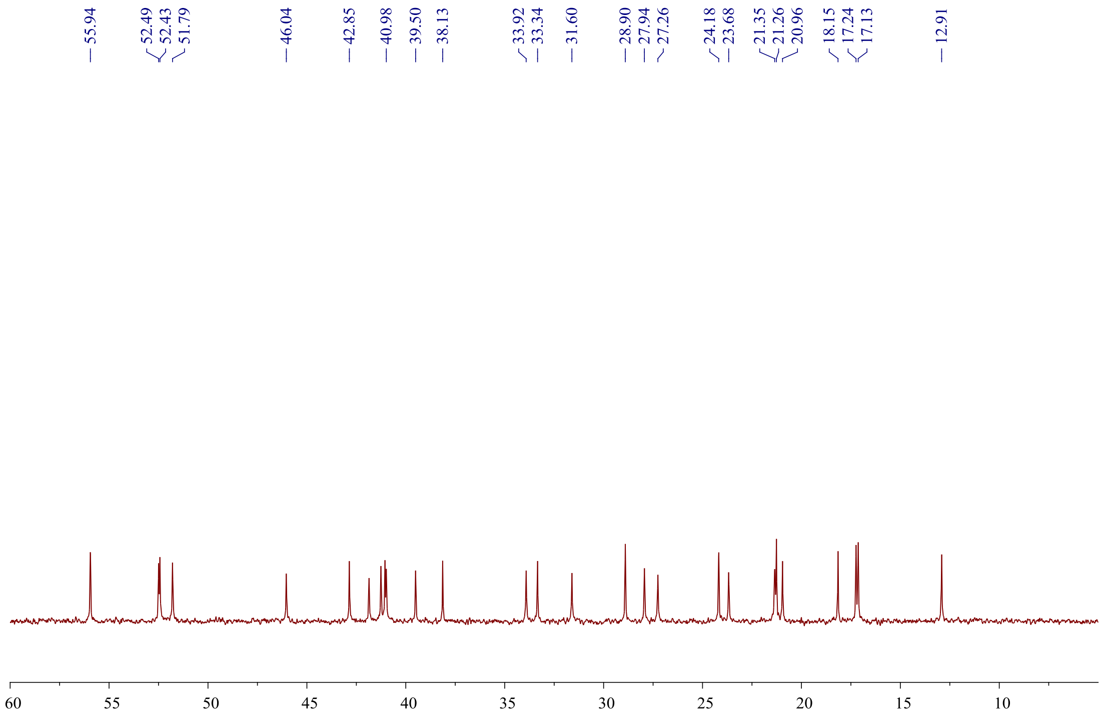

Fig. S102. HSQC spectrum of compound **11** in C_5D_5N (600 MHz).

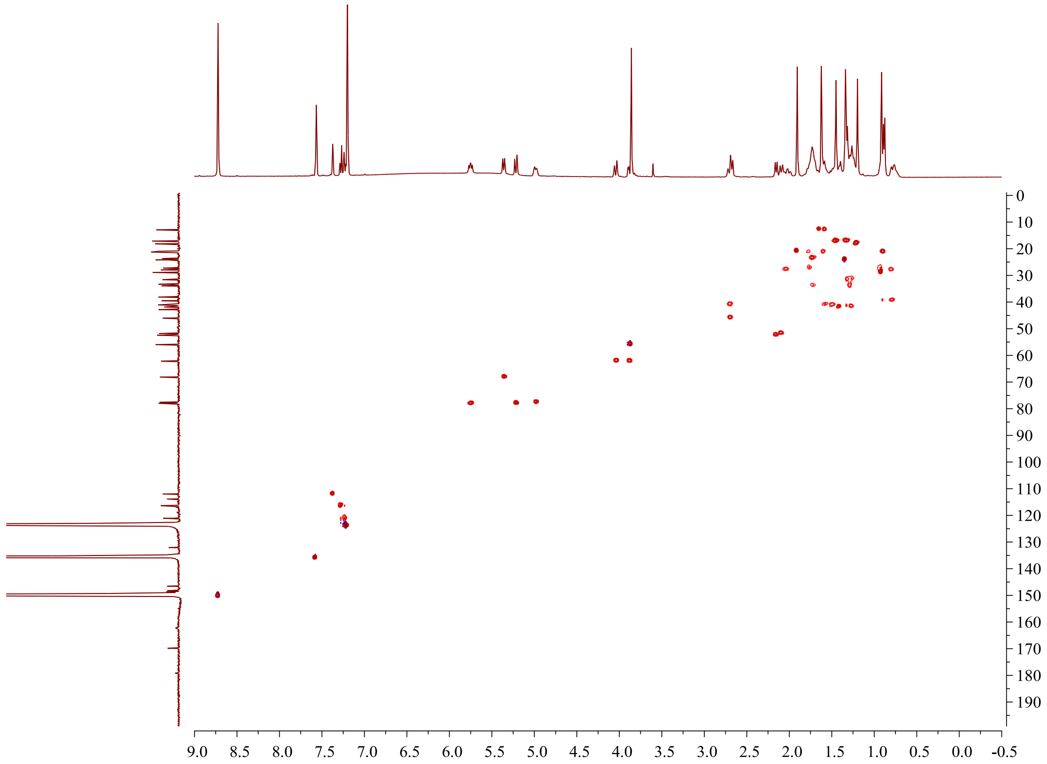

Fig. S103.^{1}H-^{1}H COSY spectrum of compound **11** in C_5D_5N (600 MHz).

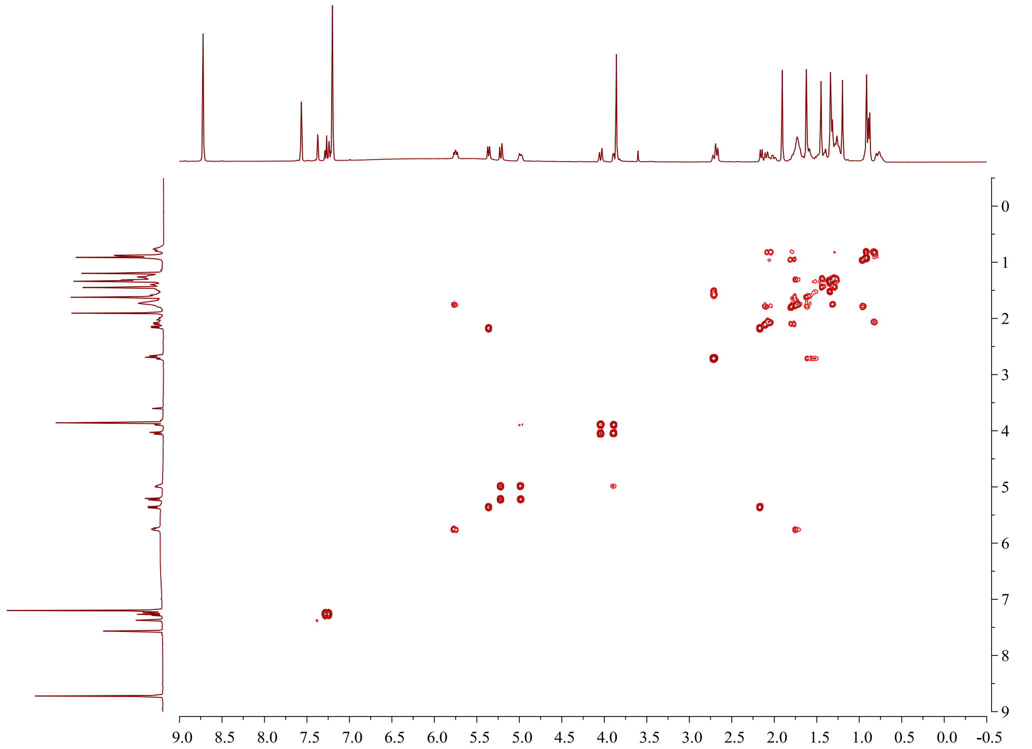

Fig. S104. HMBC spectrum of compound **11** in C_5D_5N (600 MHz).

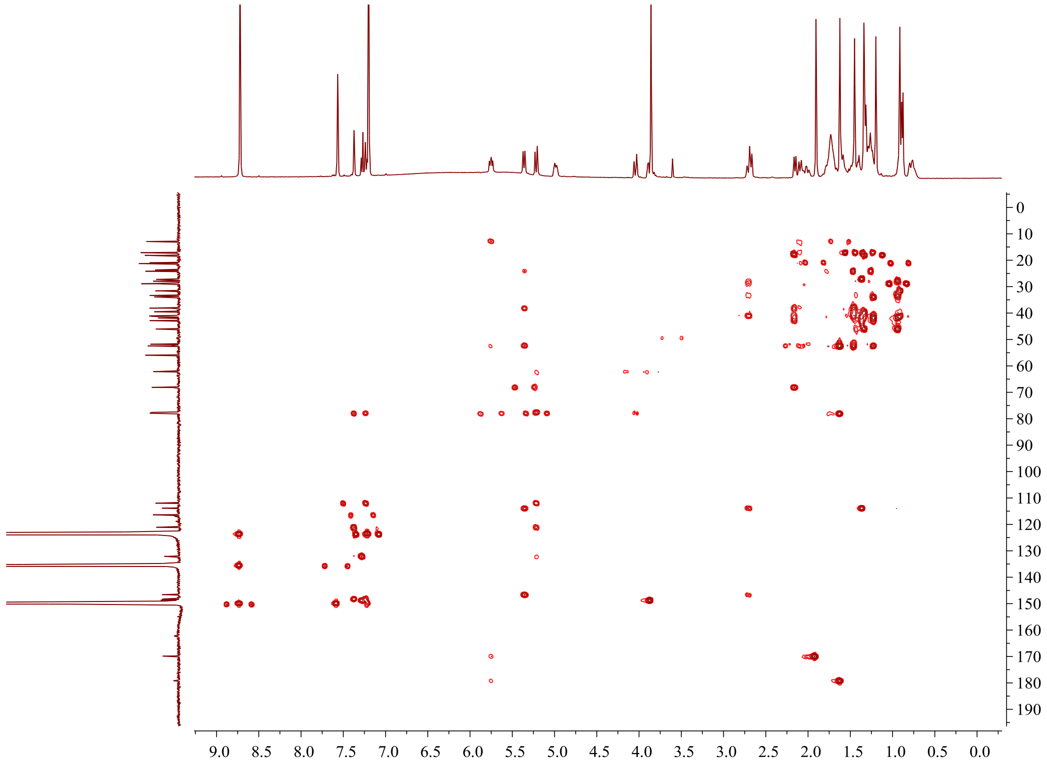

Fig. S105. HMBC spectrum of compound **11** –expansion.

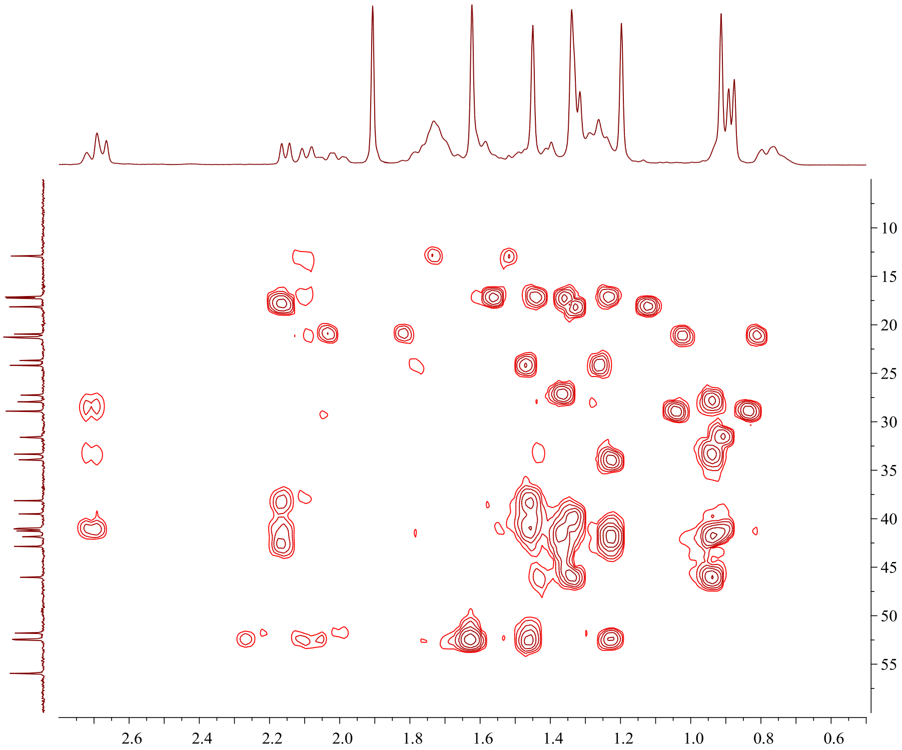

Fig. S106. ROESY spectrum of compound **11** in C_5D_5N (600 MHz).

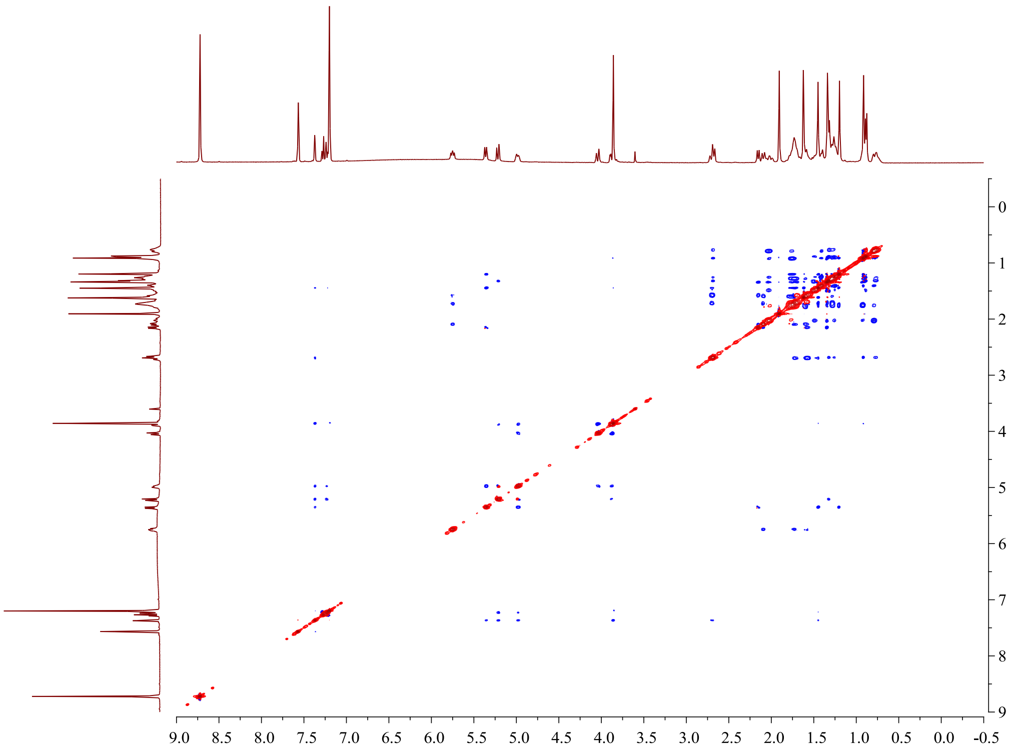

Fig. S107. ROESY spectrum of compound **11** –expansion.

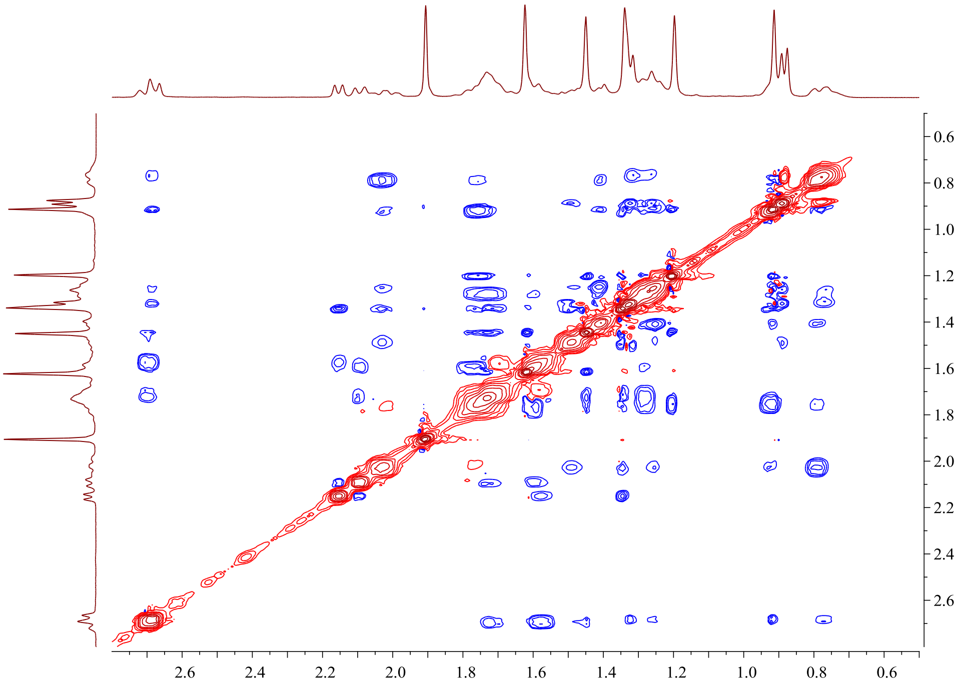

Fig. S108. HRESIMS report of compound **11**.

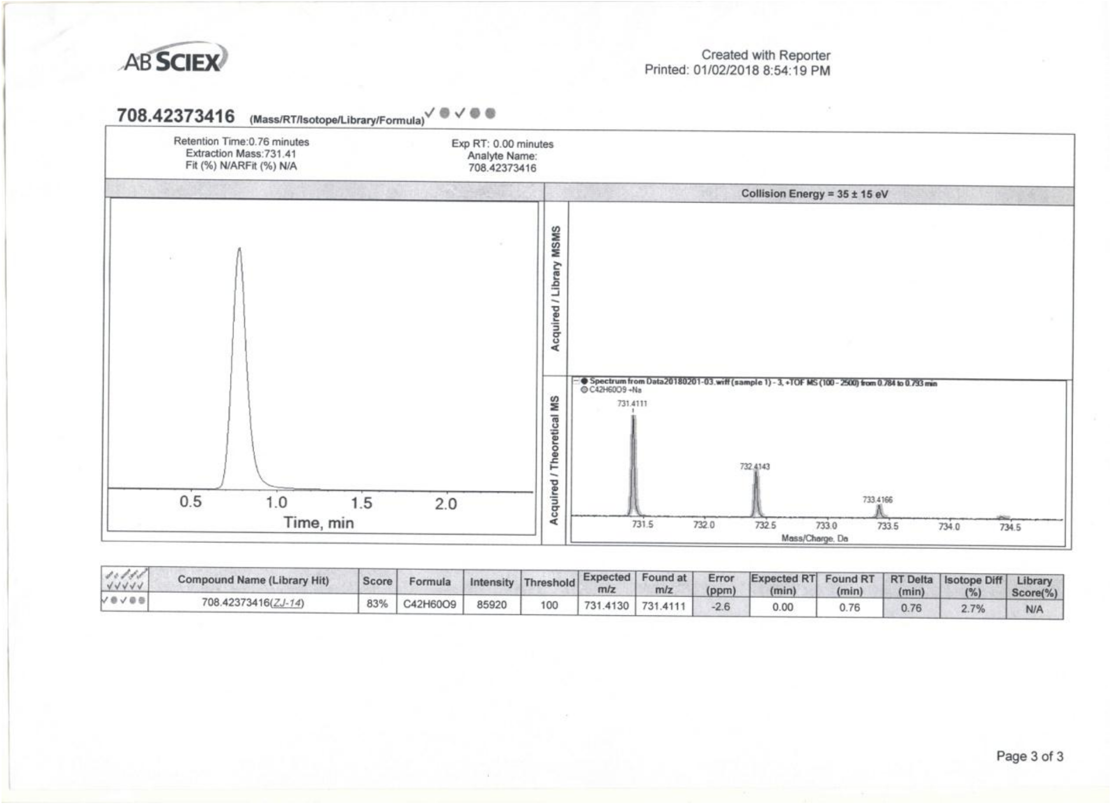

	Compound Name (Library Hit)	Score	Formula	Intensity	Threshold	Expected m/z	Found at m/z	Error (ppm)	Expected RT (min)	Found RT (min)	RT Delta (min)	Isotope Diff (%)	Library Score(%)
	708.42373416(ZJ-14)	83%	C42H60O9	85920	100	731.4130	731.4111	-2.6	0.00	0.76	0.76	2.7%	N/A

Fig. S109. ^{1}H NMR spectrum of compound **12** in C_5D_5N (400 MHz).

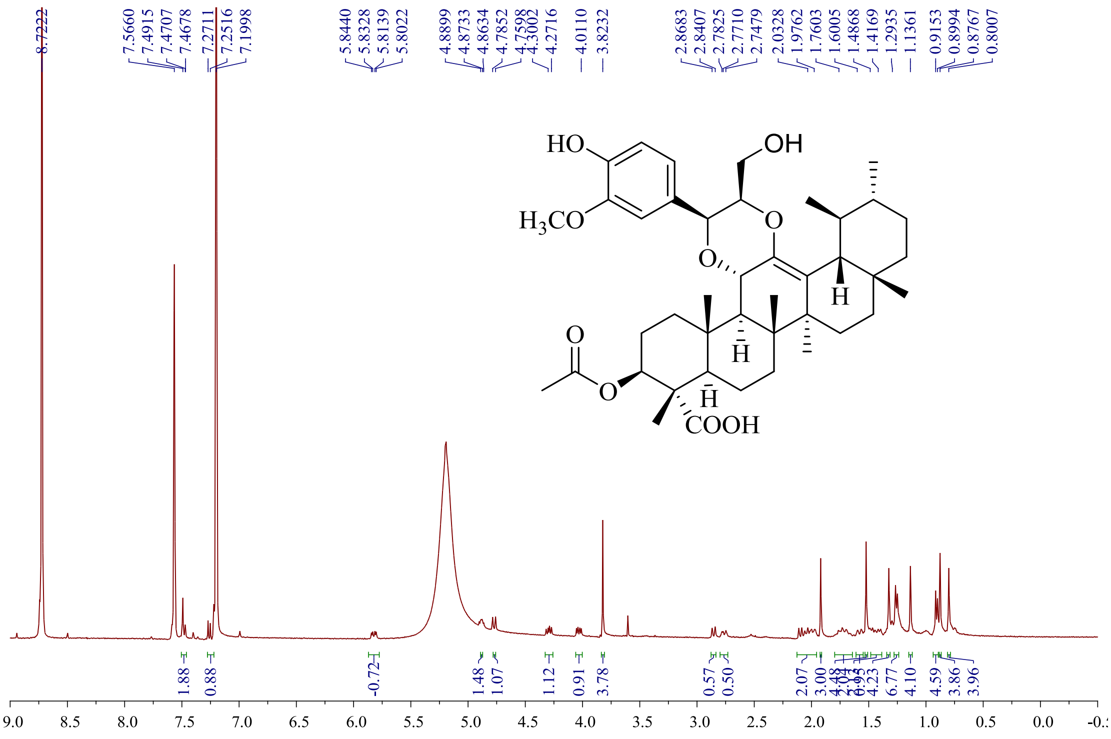

Fig. S110. ^{1}H NMR spectrum of compound **12**–expansion.

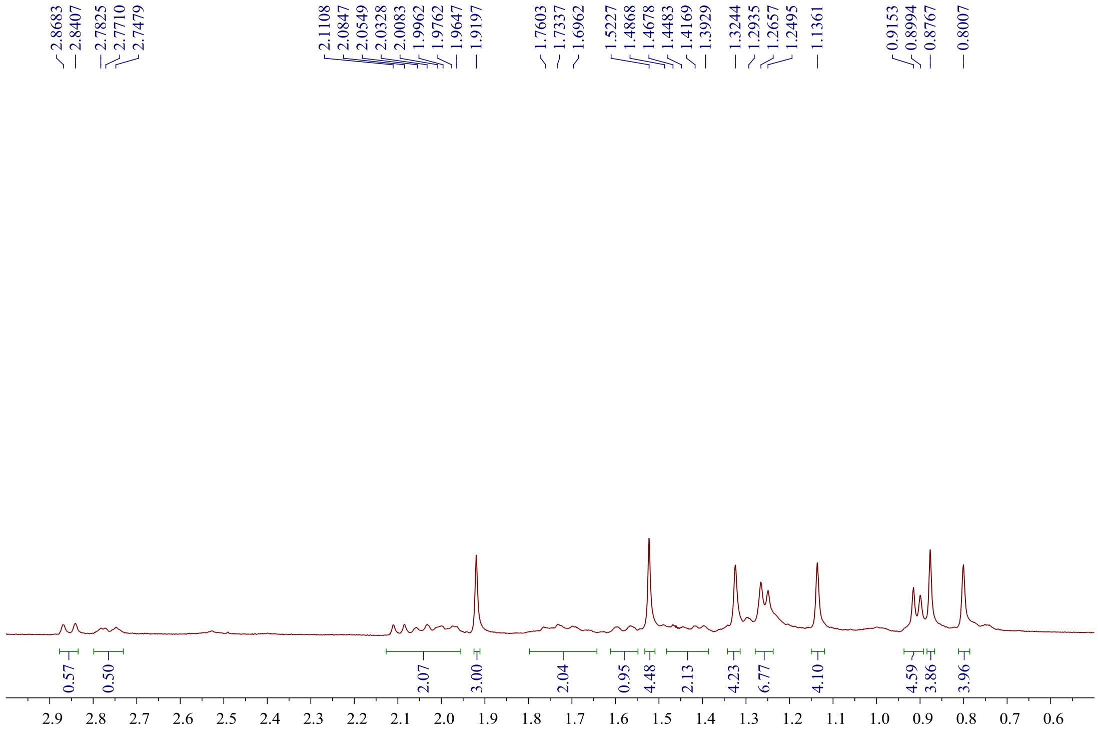

Fig. S111. ^{13}C NMR spectrum of compound **12** in C_5D_5N (150 MHz).

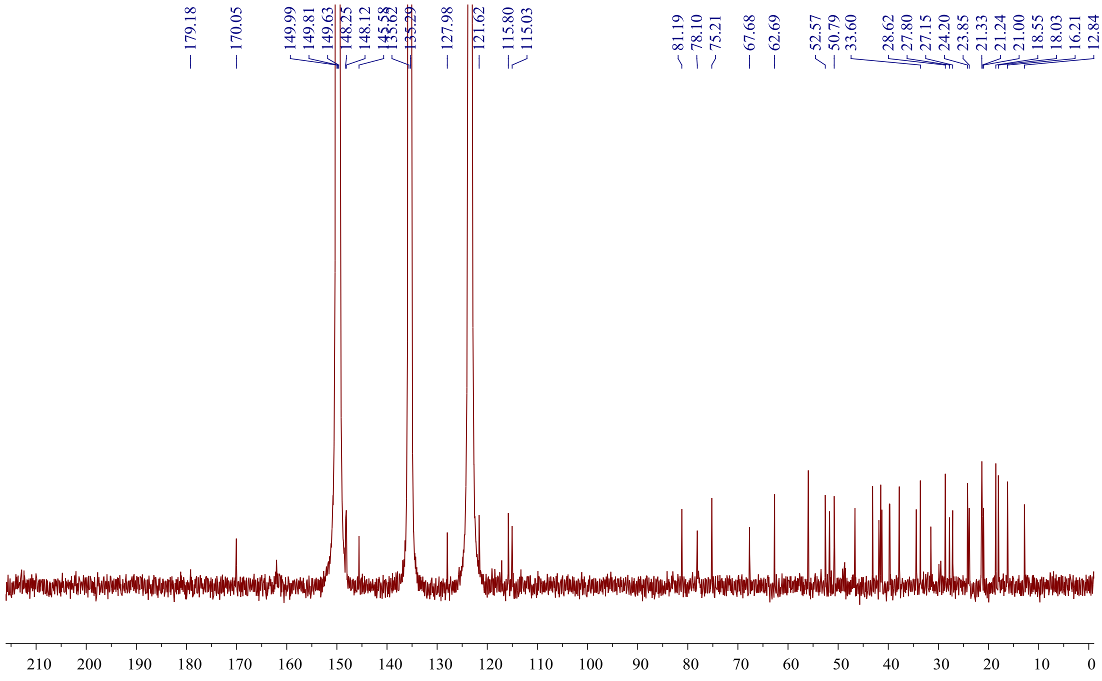

Fig. S112. ^{13}C NMR spectrum of compound **12**–expansion.

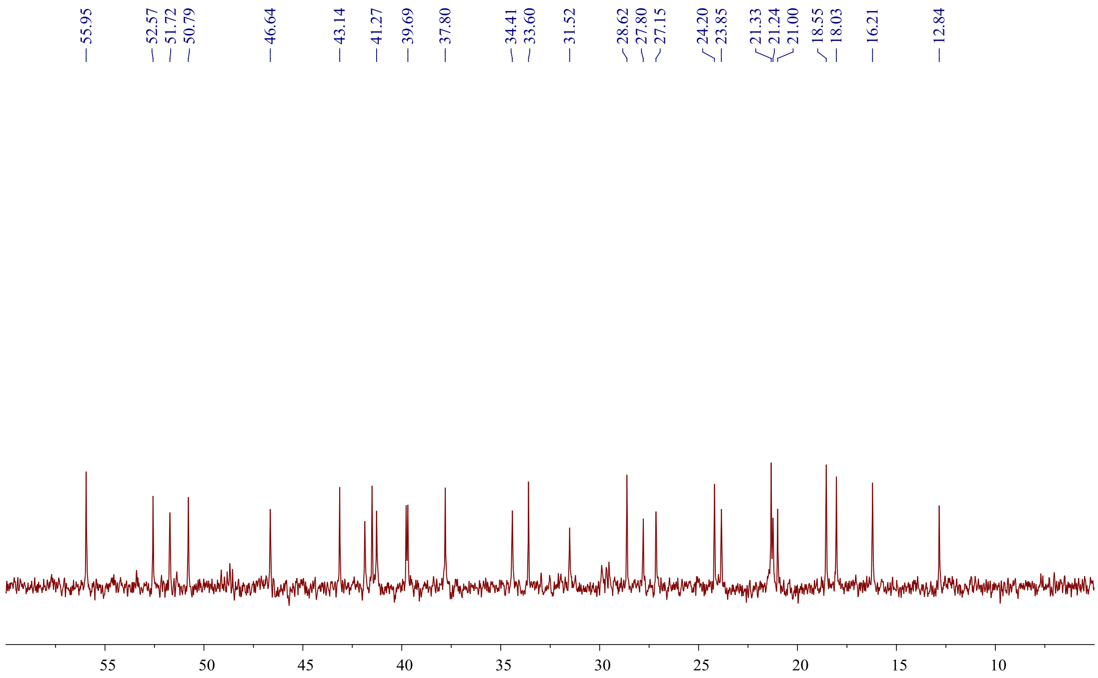

Fig. S113. ^{1}H-^{1}H COSY spectrum of compound **12** in C_5D_5N (600 MHz).

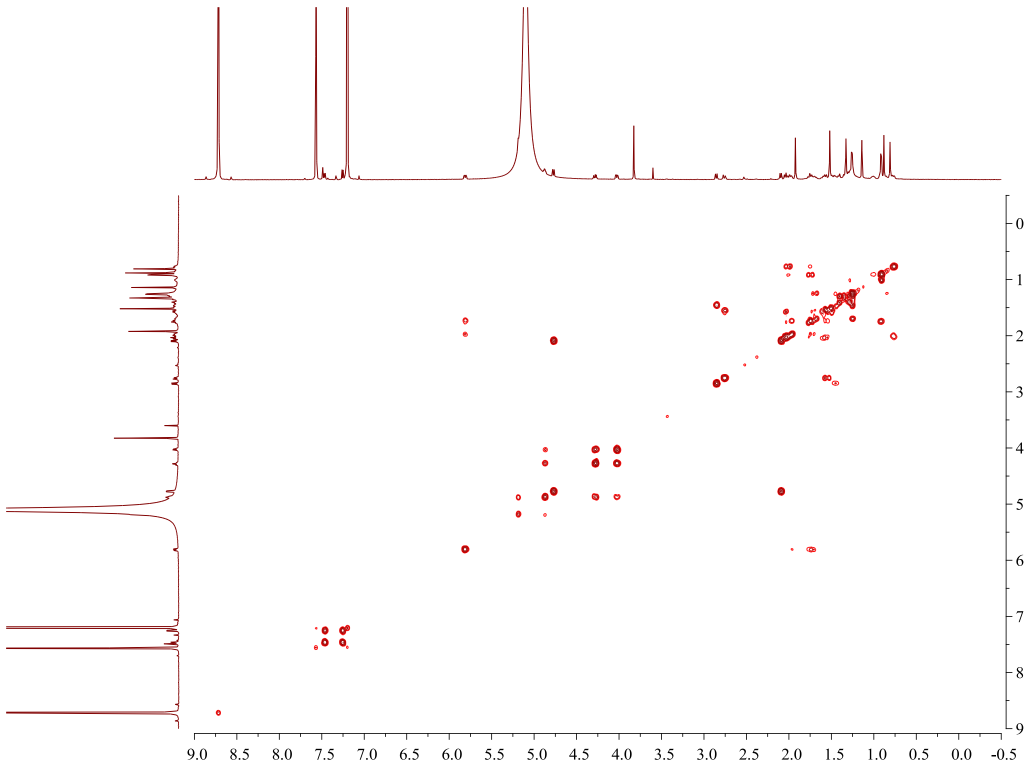

Fig. S114. HMBC spectrum of compound **12** in C_5D_5N (600 MHz).

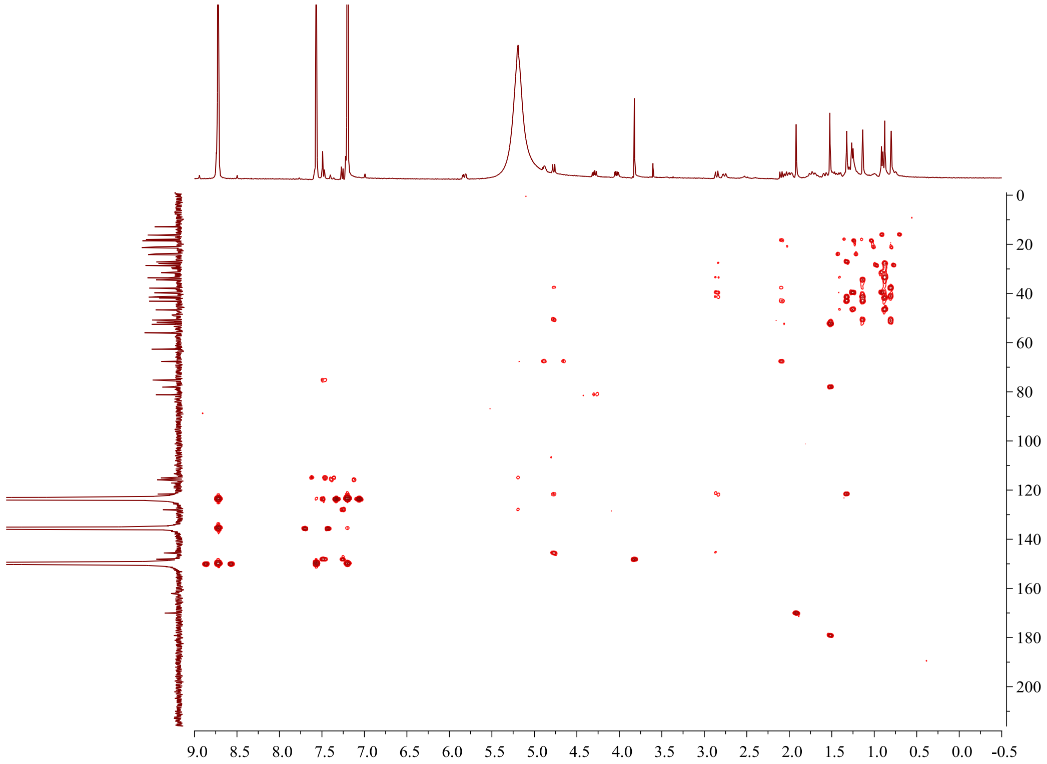

Fig. S115. ROESY spectrum of compound **12** in C_5D_5N (600 MHz).

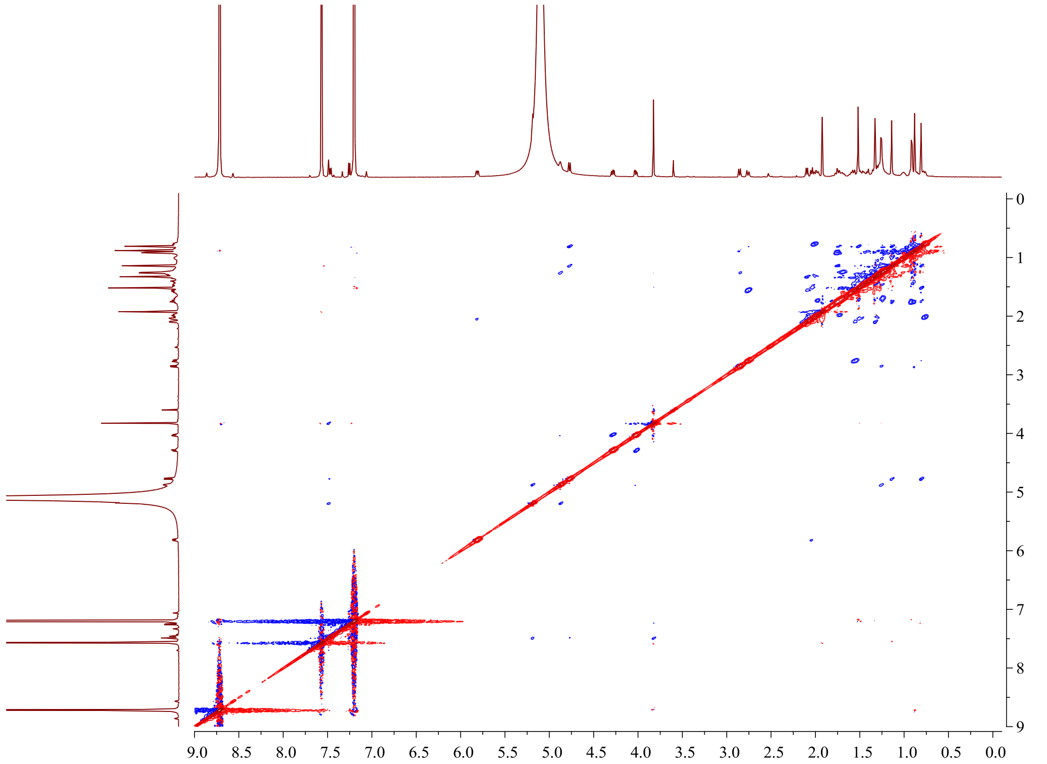

Fig. S116. HRESIMS report of compound **12**.

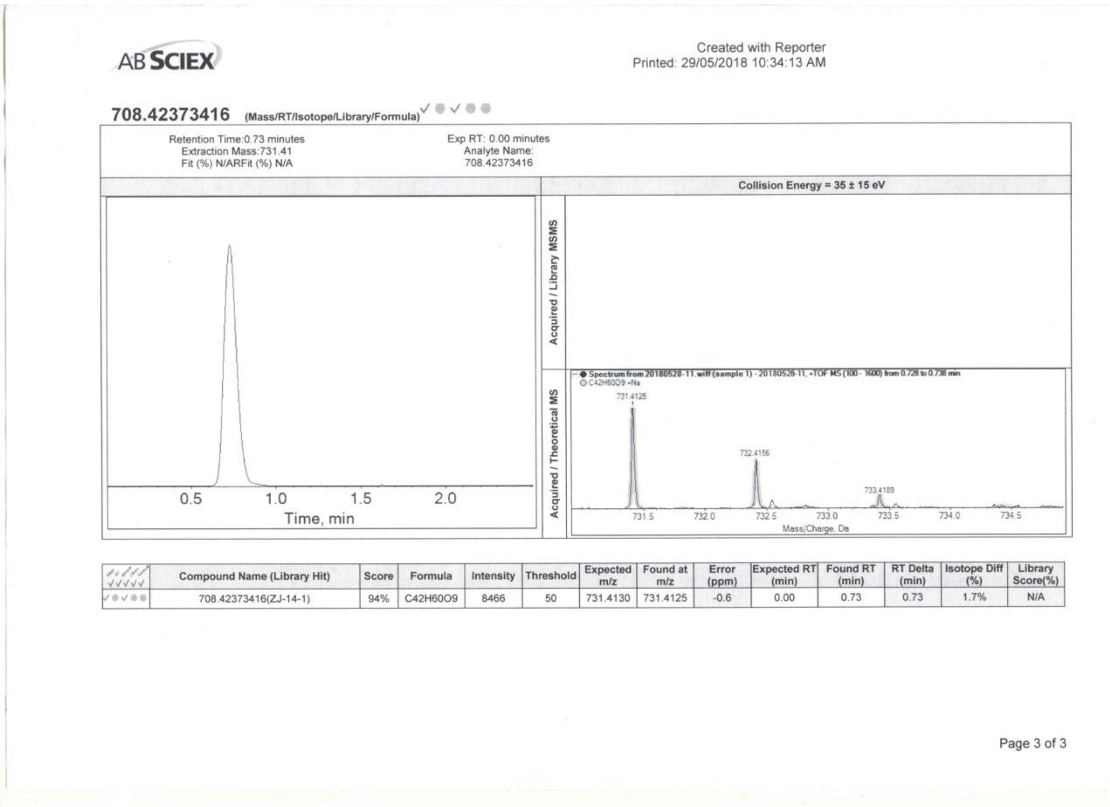

Fig. S117. ^{1}H NMR spectrum of compound **13** in CD3OD (400 MHz)

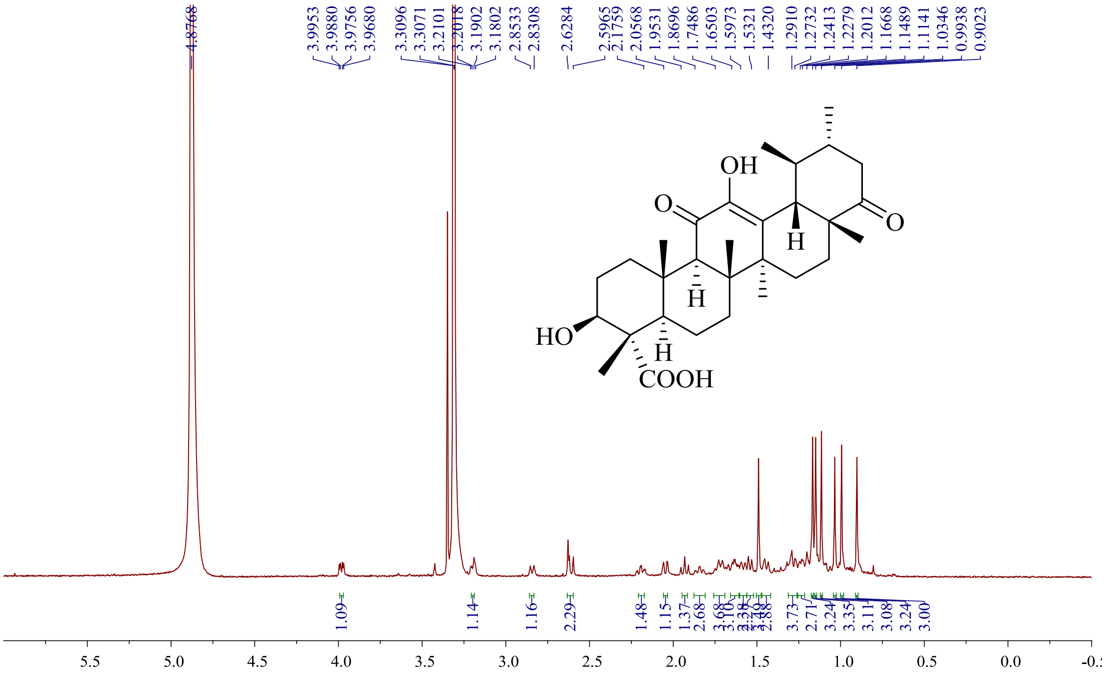

Fig. S118. ^{1}H NMR spectrum of compound **13** –expansion

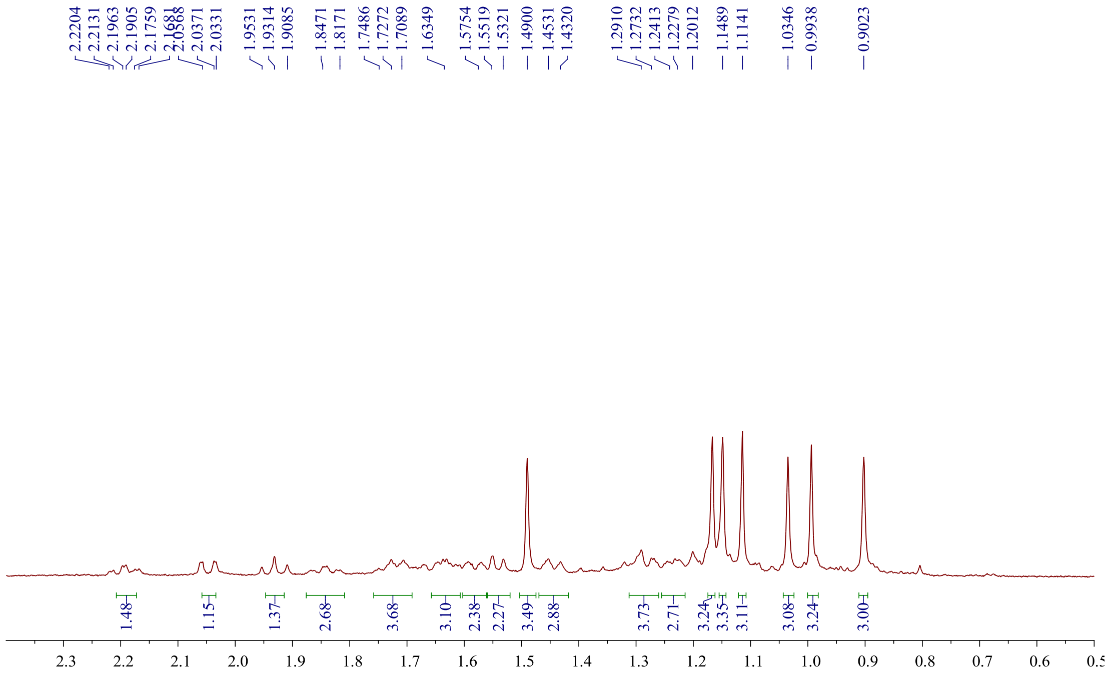

Fig. S119. ^{13}C NMR spectrum of compound **13** in CD_3OD (150 MHz)

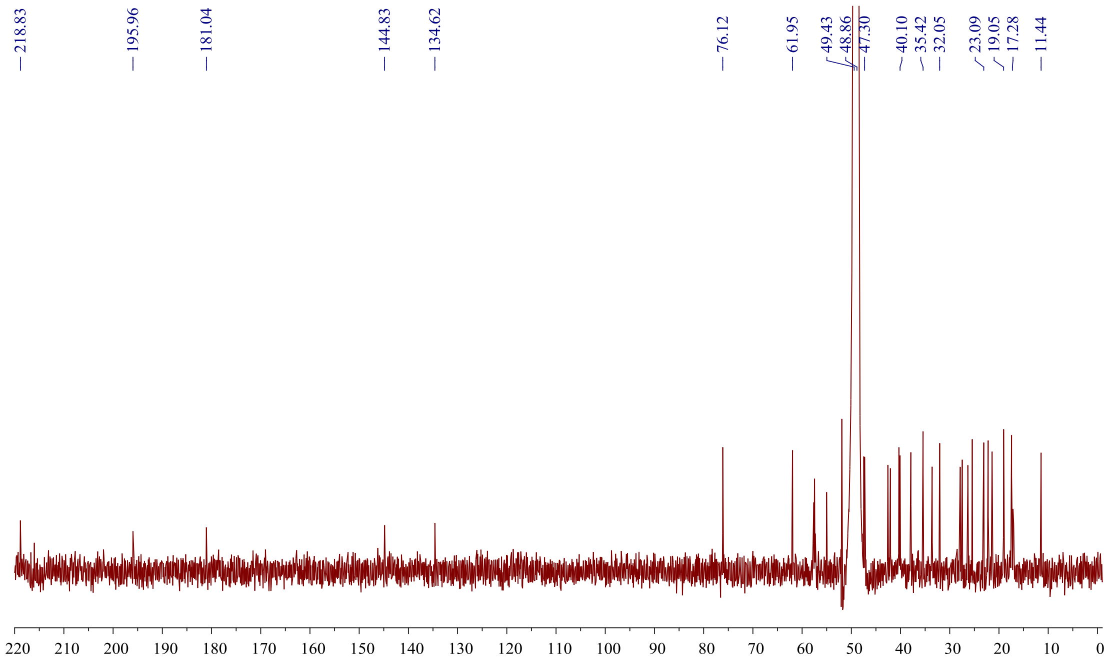

Fig. S120. ^{13}C NMR spectrum of compound **13** –expansion

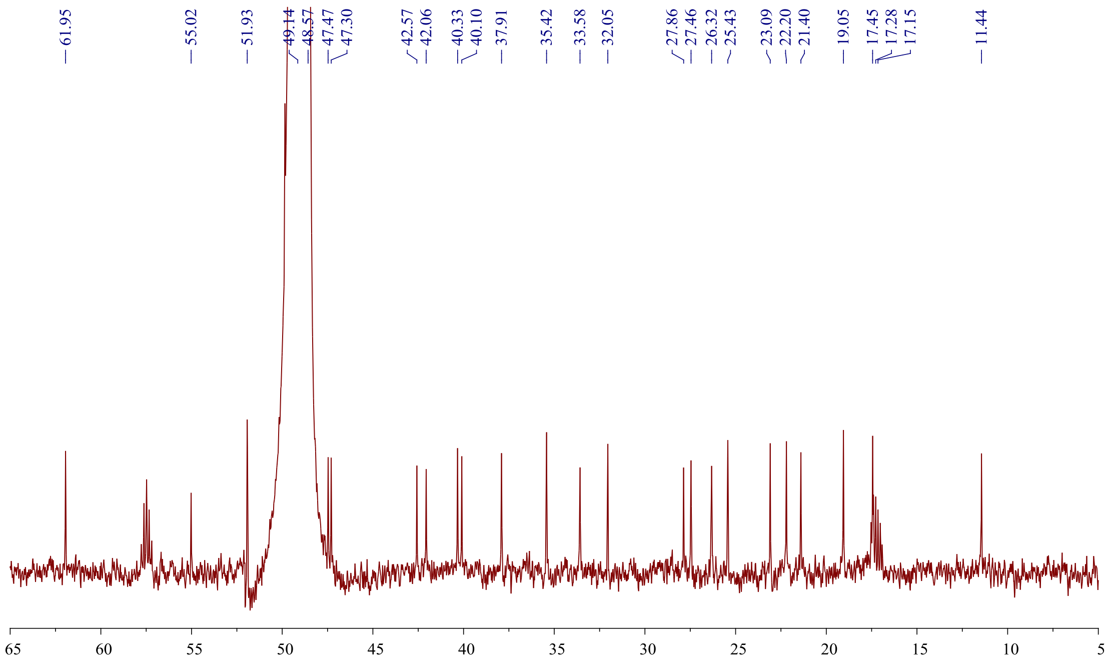

Fig. S121. HMBC spectrum of compound **13** in CD3OD (600 MHz)

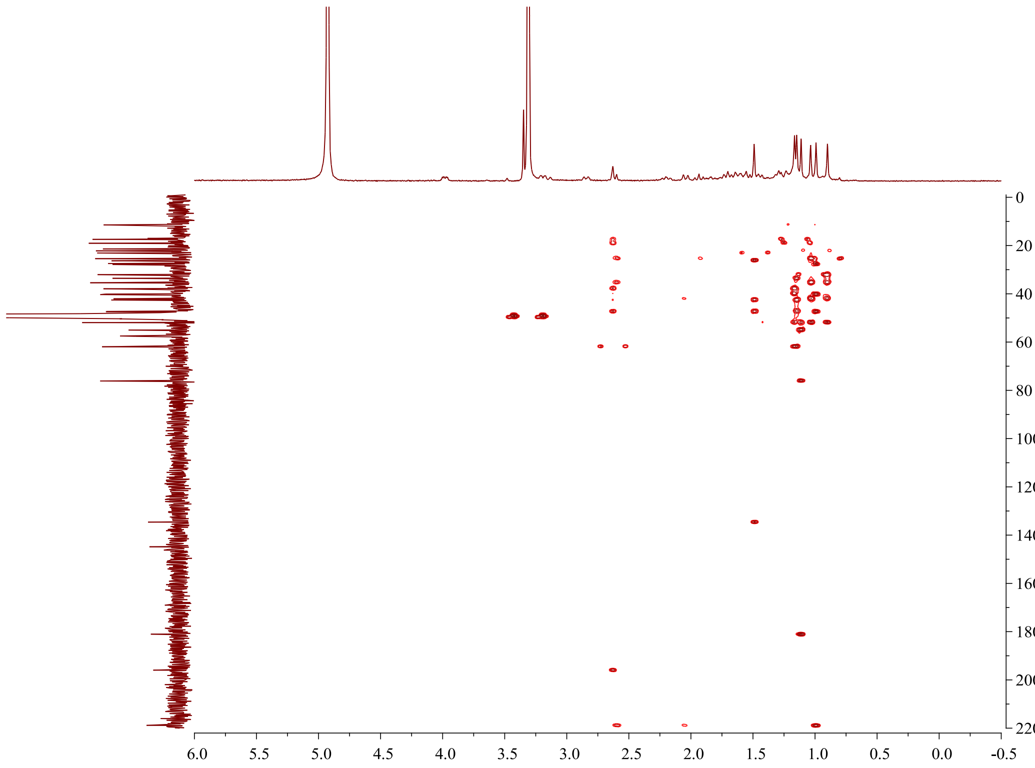

Fig. S122. HMBC spectrum of compound **13**-expansion

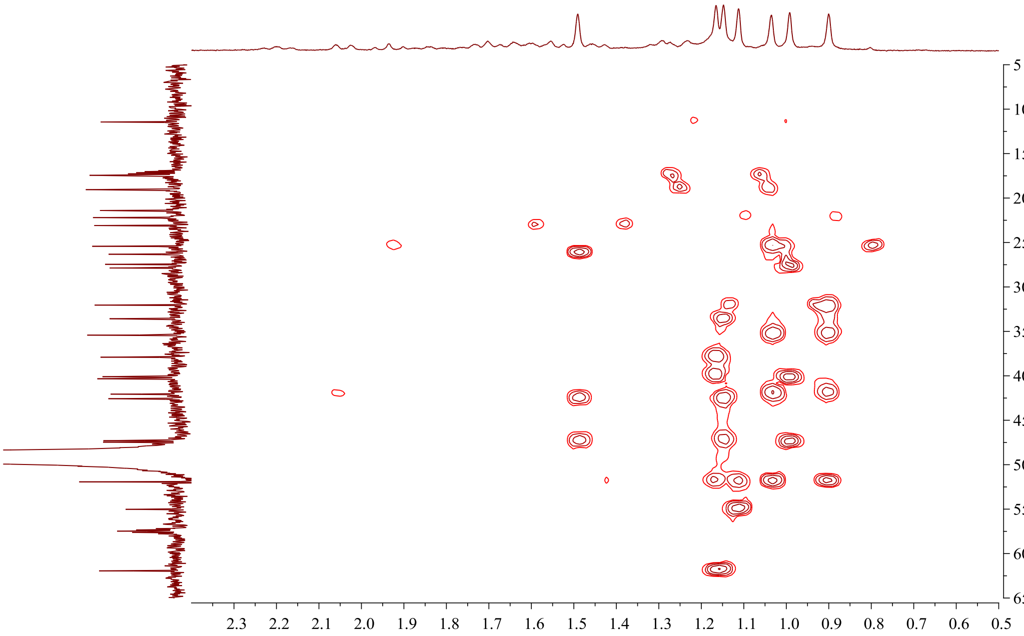

Fig. S123. ROESY spectrum of compound **13** in CD_3OD (600 MHz)

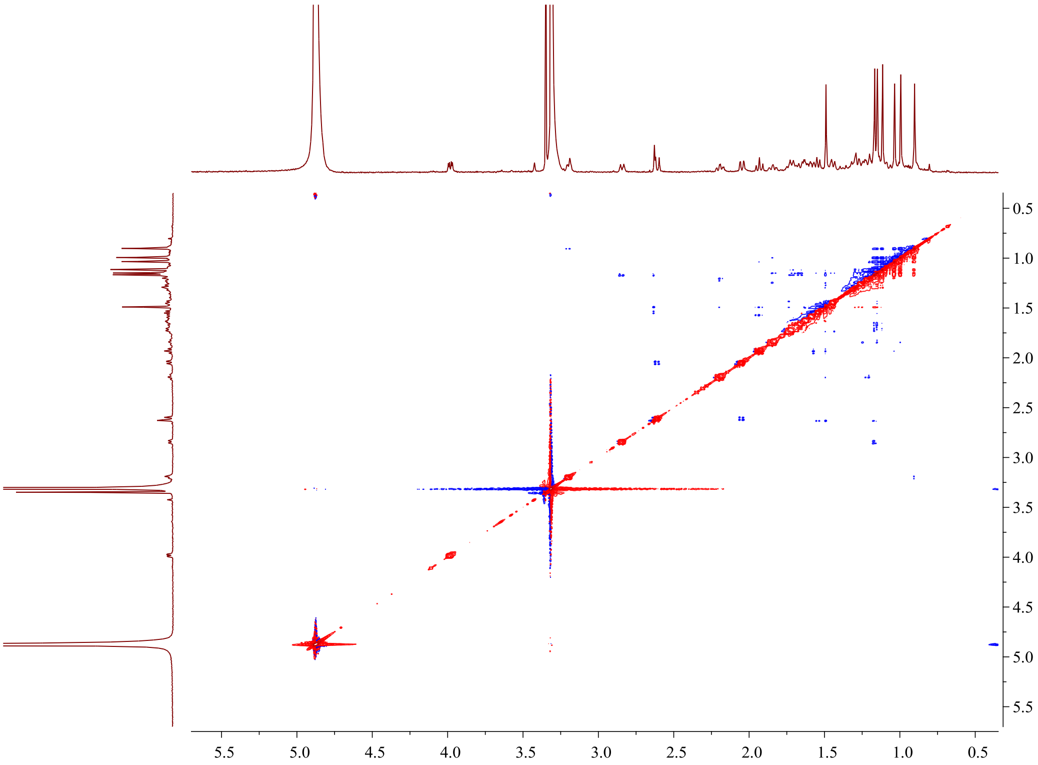

Fig. S124. HR-ESIMS report of compound **13**

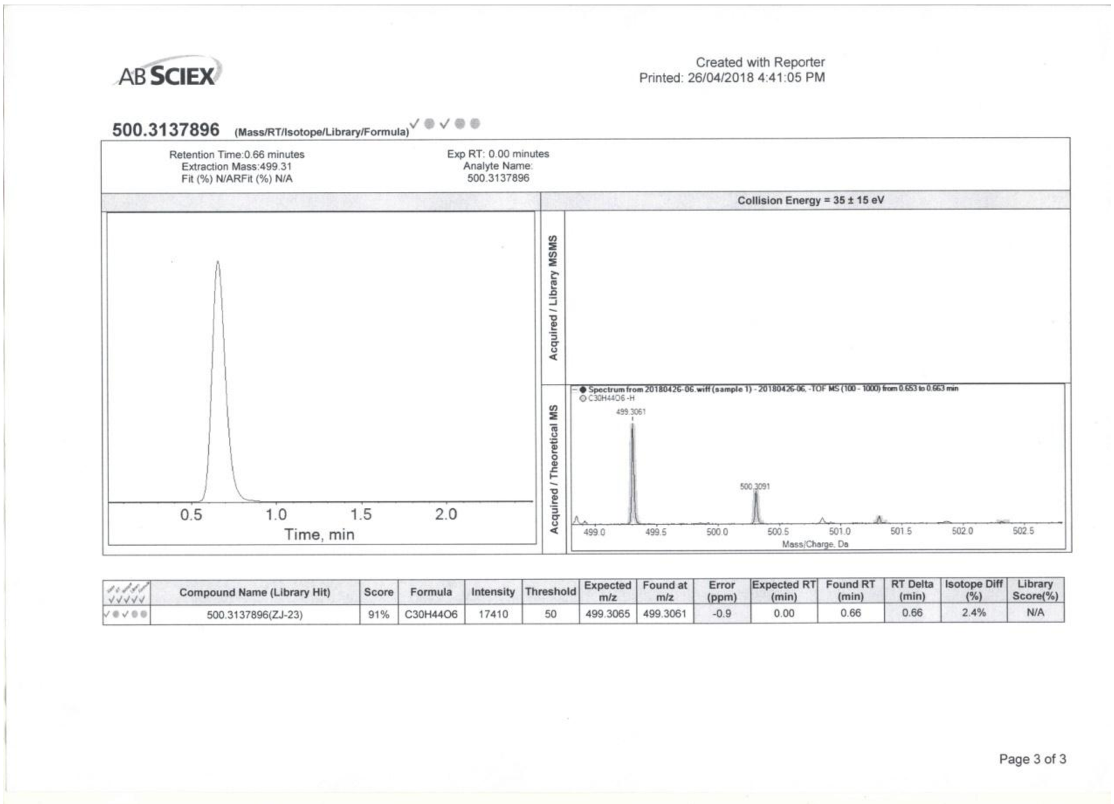

	Compound Name (Library Hit)	Score	Formula	Intensity	Threshold	Expected m/z	Found at m/z	Error (ppm)	Expected RT (min)	Found RT (min)	RT Delta (min)	Isotope Diff (%)	Library Score(%)
	500.3137896(ZJ-23)	91%	C30H44O6	17410	50	499.3065	499.3061	-0.9	0.00	0.66	0.66	2.4%	N/A

Fig. S125. ^{1}H NMR spectrum of compound **14** in C_5D_5N (400 MHz).

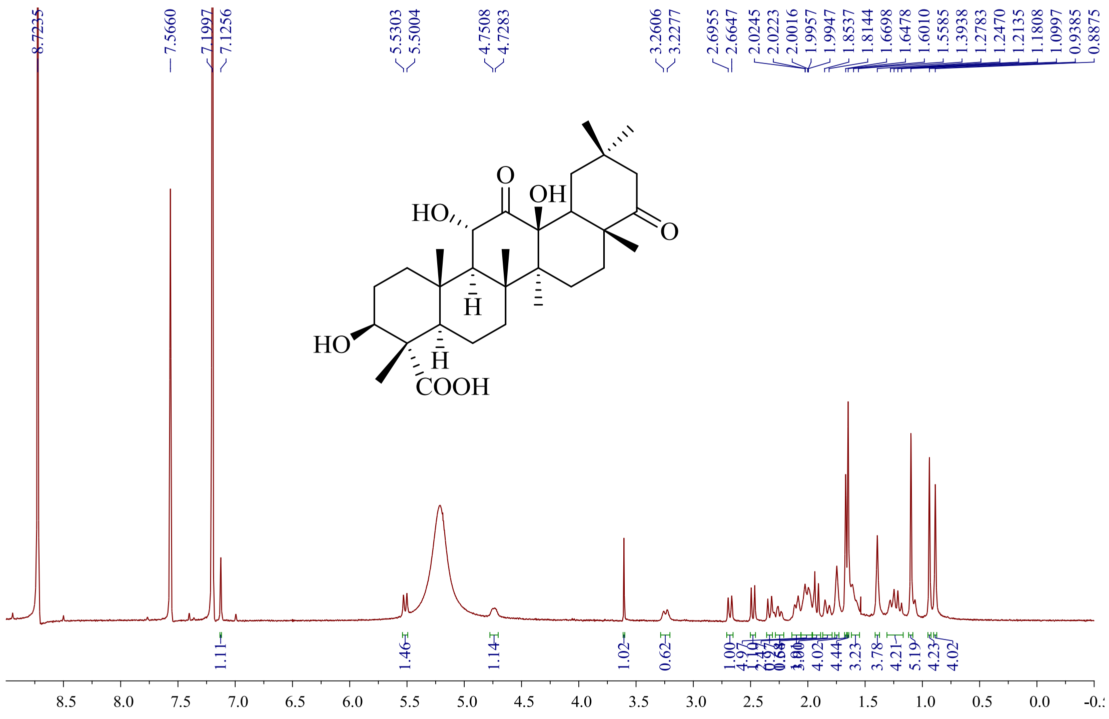

Fig. S126. ^{1}H NMR spectrum of compound **14** –expansion.

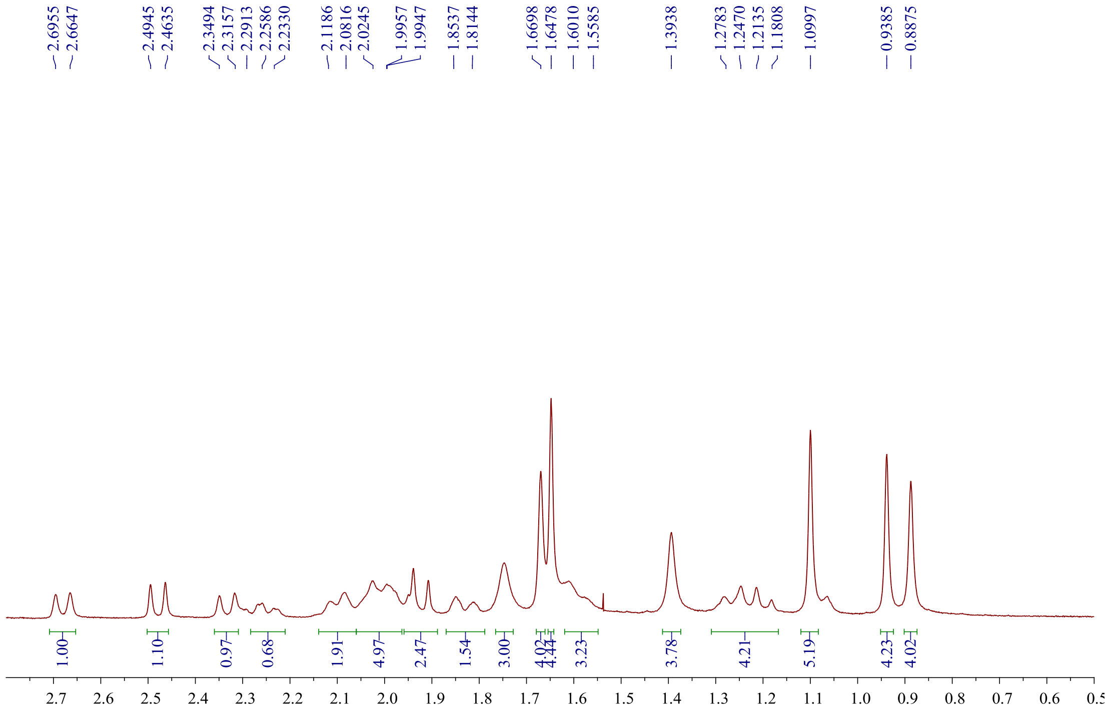

Fig. S127. ^{13}C NMR spectrum of compound **14** in C_5D_5N (150 MHz).

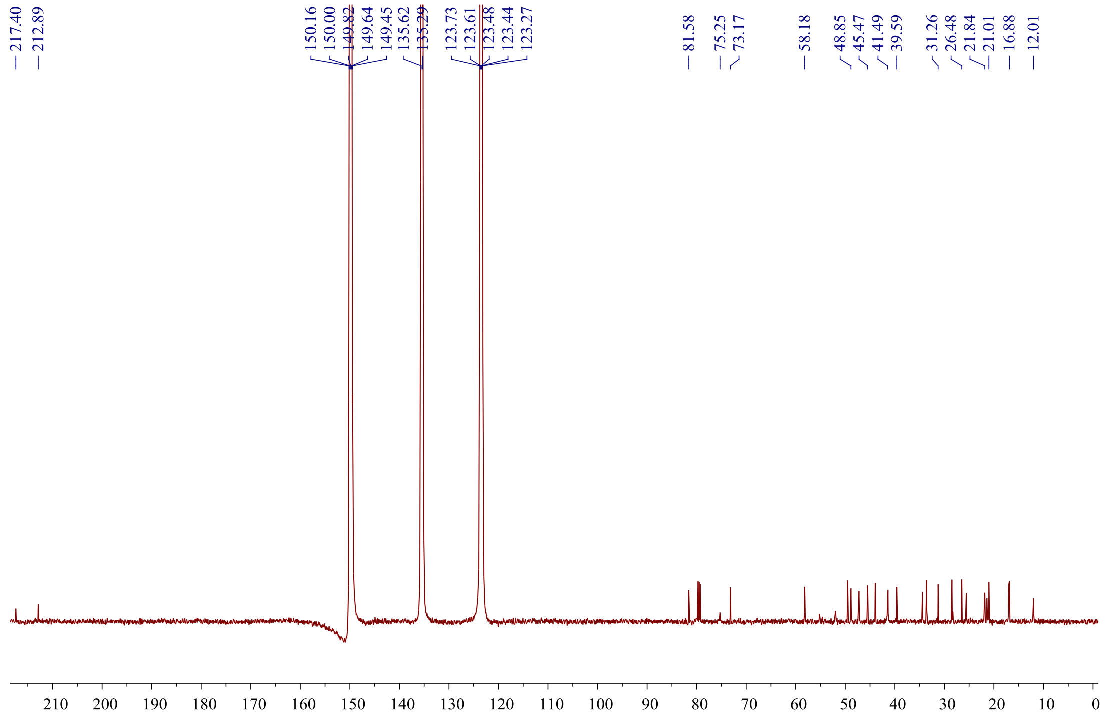

Fig. S128. ^{13}C NMR spectrum of compound **14**-expansion.

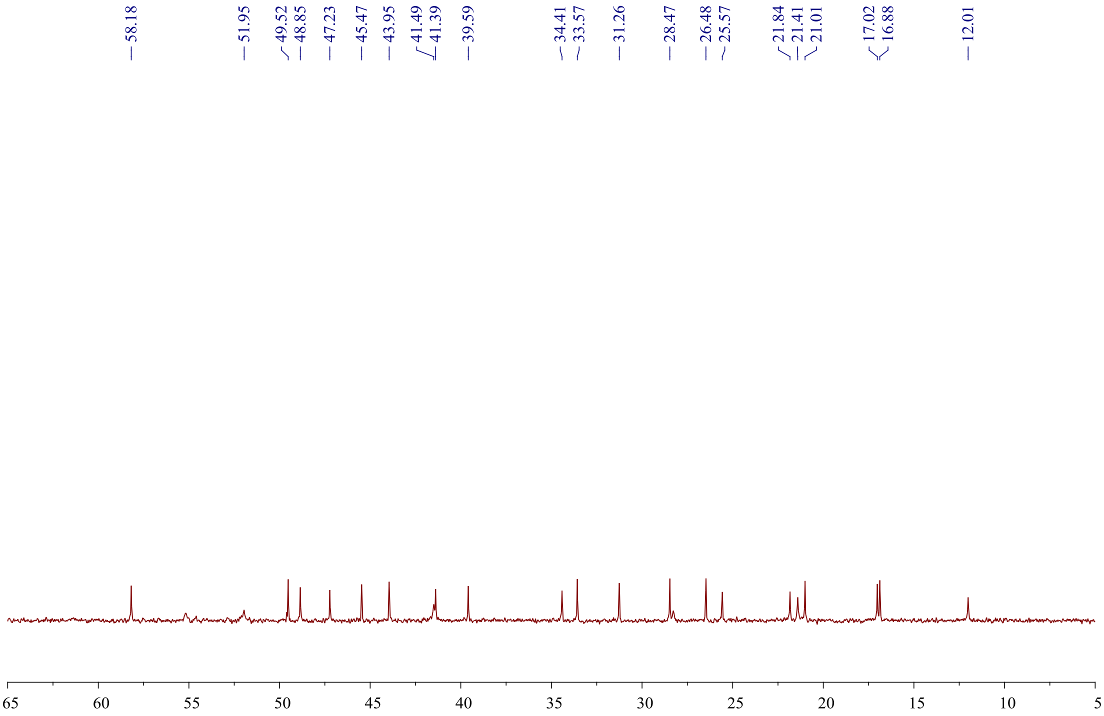

Fig. S129. HMBC spectrum of compound **14** in C_5D_5N (600 MHz).

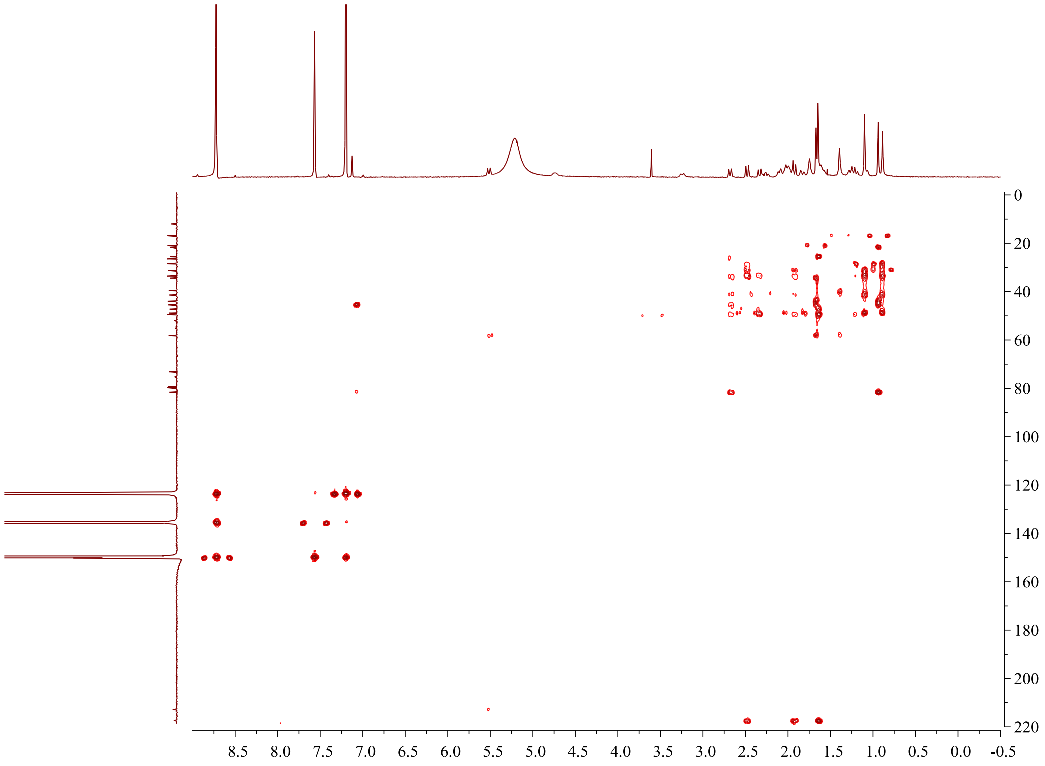

Fig. S130. HMBC spectrum of compound **14**-expansion.

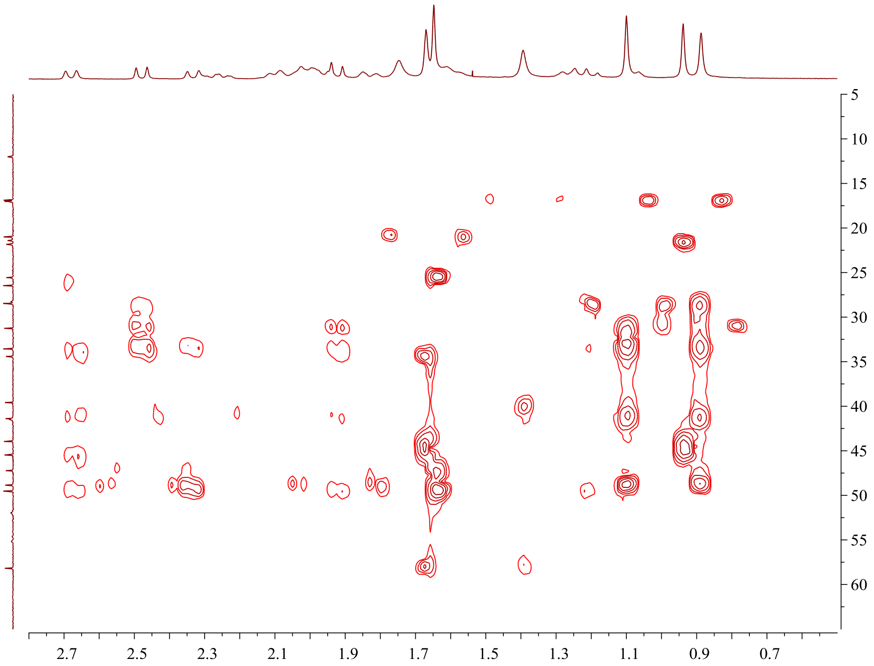

Fig. S131. ROESY spectrum of compound **14** in C_5D_5N (600 MHz).

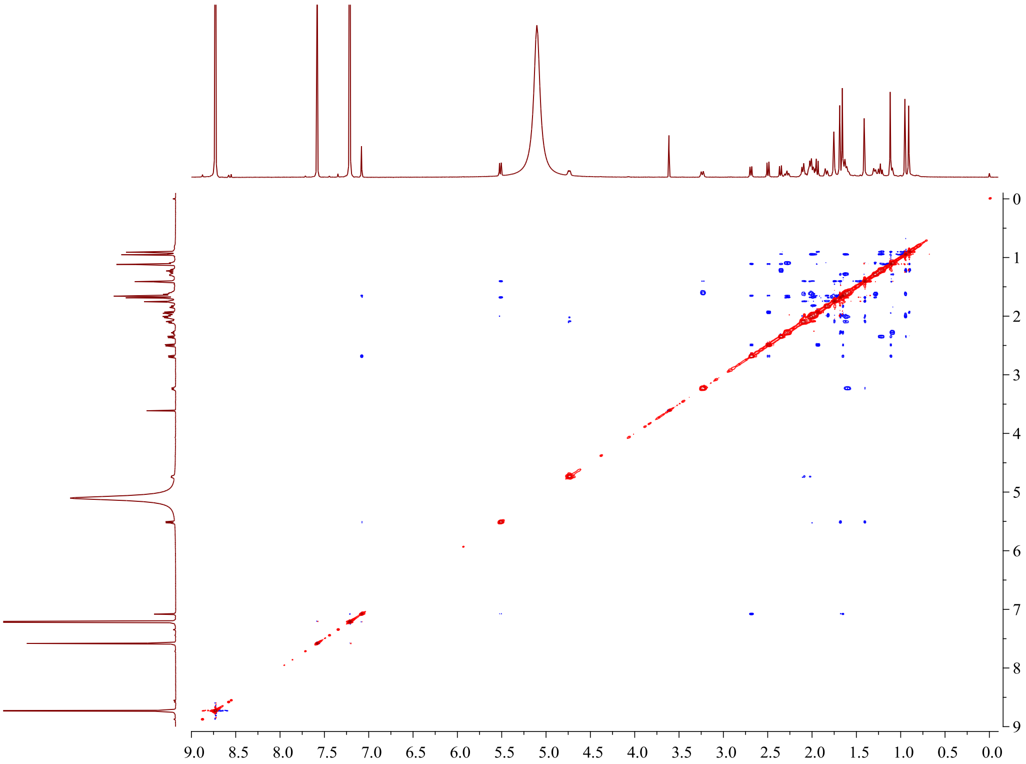

Fig. S132. HRESIMS report of compound **14**.

AB SCIEX

Created with Reporter
Printed: 26/06/2018 4:51:43 PM

518.32435432 (Mass/RT/Isotope/Library/Formula)

Retention Time:0.49 minutes
Extraction Mass:541.31
Fit (%) N/ARFit (%) N/A

Exp RT: 0.00 minutes
Analyte Name:
518.32435432

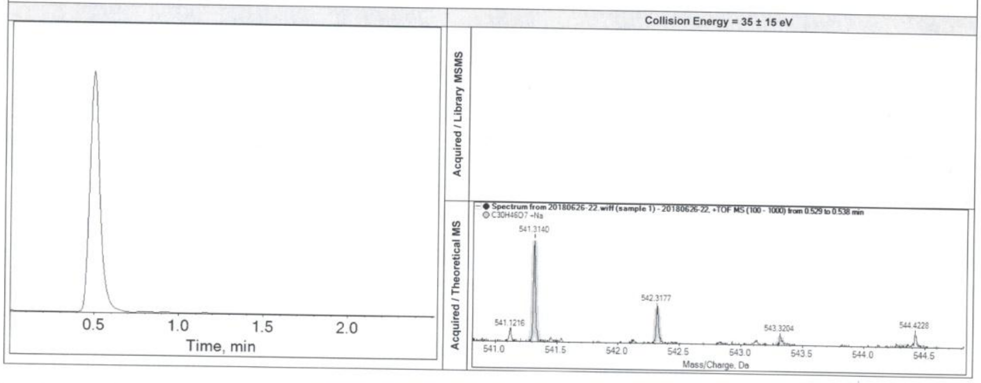

	Compound Name (Library Hit)	Score	Formula	Intensity	Threshold	Expected m/z	Found at m/z	Error (ppm)	Expected RT (min)	Found RT (min)	RT Delta (min)	Isotope Diff (%)	Library Score(%)
	518.32435432 (ZJ-16)	83%	C30H46O7	92112	50	541.3136	541.3140	0.9	0.00	0.49	0.49	5.2%	N/A

Page 3 of 3

Table S1. Crystal data and structure refinement for stewartiacid A (**1**)

Identification code	mjr17084_0m	
Empirical formula	$C_{30}H_{46}O_6$	
Formula weight	502.67	
Temperature	170 K	
Wavelength	1.34139 Å	
Crystal system	Monoclinic	
Space group	P 1 21 1	
Unit cell dimensions	a = 14.2747(19) Å	α = 90 °
	b = 6.4197(9) Å	β = 105.303(9) °
	c = 14.897(2) Å	γ = 90 °
Volume	1316.7(3) $Å^3$	
Z	2	
Density (calculated)	1.268 Mg/m^3	
Absorption coefficient	0.444 mm^{-1}	
F(000)	548	
Crystal size	0.05 x 0.02 x 0.01 mm^3	
Theta range for data collection	3.319 to 55.147 °	
Index ranges	-17<=h<=17, -7<=k<=7, -16<=l<=18	
Reflections collected	14495	
Independent reflections	4786 [R(int) = 0.0521]	
Completeness to theta =	99.2%	
Absorption correction	Semi-empirical from equivalents	
Max. and min. transmission	0.7508 and 0.4336	
Refinement method	Full-matrix least-squares on F^2	
Data / restraints / parameters	4786 / 1 / 343	
Goodness-of-fit on F^2	1.061	
Final R indices [I>2sigma(I)]	R1 = 0.0683, wR2 = 0.1556	
R indices (all data)	R1 = 0.0866, wR2 = 0.1718	
Absolute structure parameter	0.19(17)	
Extinction coefficient	0.0051(14)	
Largest diff. peak and hole	0.323 and -0.320 e.$Å^{-3}$	

Table S2. Crystal data and structure refinement for stewartiacid C (**3**)

Identification code	191209wj_0m	
Empirical formula	$C_{30}H_{50}O_9$	
Formula weight	554.70	
Temperature	169.98 K	
Wavelength	1.34139 Å	
Crystal system	Orthorhombic	
Space group	$P2_12_12_1$	
Unit cell dimensions	a = 8.0649(6) Å	α = 90 °.
	b = 11.3973(8) Å	β = 90 °.
	c = 32.416(2) Å	γ = 90 °.
Volume	2979.6(4) Å^3	
Z	4	
Density (calculated)	1.237 Mg/m^3	
Absorption coefficient	0.471 mm^{-1}	
F (000)	1208	
Crystal size	0.15 x 0.1 x 0.008 mm^3	
Theta range for data collection	3.576 to 55.060 °.	
Index ranges	-9<=h<=7, -11<=k<=13, -39<=l<=39	
Reflections collected	21220	
Independent reflections	5558 [R (int) = 0.0705]	
Completeness to theta = 53.594 °	98.5 %	
Absorption correction	Semi-empirical from equivalents	
Max. and min. transmission	0.7508 and 0.3675	
Refinement method	Full-matrix least-squares on F^2	
Data / restraints / parameters	5558 / 0 / 372	
Goodness-of-fit on F^2	1.031	
Final R indices [I>2 sigma (I)]	R1 = 0.0949, wR2 = 0.2521	
R indices (all data)	R1 = 0.1371, wR2 = 0.2937	
Absolute structure parameter	-0.01(19)	
Extinction coefficient	0.029(4)	
Largest diff. peak and hole	0.292 and -0.310 e.Å^{-3}	

Table S3. Crystal data and structure refinement for **s**tewartiacid D (**4**).

Identification code	mjl18283_0m	
Empirical formula	$C_{30}H_{46}O_6$	
Formula weight	502.67	
Temperature	170.0 K	
Wavelength	1.34139 Å	
Crystal system	Monoclinic	
Space group	P 1 21 1	
Unit cell dimensions	a = 13.9664(4) Å	α = 90 °.
	b = 6.5552(2) Å	β = 104.366(2) °.
	c = 15.1284(5) Å	γ = 90 °.
Volume	1341.73(7) Å^3	
Z	2	
Density (calculated)	1.244 Mg/m^3	
Absorption coefficient	0.436 mm^{-1}	
F (000)	548	
Crystal size	0.1 x 0.02 x 0.01 mm^3	
Theta range for data collection	2.841 to 54.870 °.	
Index ranges	-17<=h<=17, -7<=k<=7, -18<=l<=18	
Reflections collected	20123	
Independent reflections	5048 [R(int) = 0.0530]	
Completeness to theta = 53.594 °	99.8 %	
Absorption correction	Semi-empirical from equivalents	
Max. and min. transmission	0.7508 and 0.5858	
Refinement method	Full-matrix least-squares on F^2	
Data / restraints / parameters	5048 / 1 / 335	
Goodness-of-fit on F^2	1.038	
Final R indices [I>2 sigma(I)]	R1 = 0.0534, wR2 = 0.1558	
R indices (all data)	R1 = 0.0595, wR2 = 0.1613	
Absolute structure parameter	-0.11(13)	
Extinction coefficient	n/a	
Largest diff. peak and hole	1.397 and -0.327 e.Å^{-3}	

Table S4. Crystal data and structure refinement for **s**tewartiacid F (**6**).

Identification code	mjl18275_0m	
Empirical formula	$C_{31}H_{52}O_7$	
Formula weight	536.72	
Temperature	169.98 K	
Wavelength	1.34139 Å	
Crystal system	Monoclinic	
Space group	C 1 2 1	
Unit cell dimensions	a = 30.9220(6) Å	α= 90 °.
	b = 13.9294(3) Å	β= 107.9170(10) °.
	c = 14.7361(3) Å	γ= 90 °.
Volume	6039.4(2) Å^3	
Z	8	
Density (calculated)	1.181 Mg/m^3	
Absorption coefficient	0.421 mm^{-1}	
F(000)	2352	
Crystal size	0.15 x 0.1 x 0.02 mm^3	
Theta range for data collection	3.054 to 55.014 °.	
Index ranges	-37<=h<=37, -16<=k<=16, -17<=l<=18	
Reflections collected	48612	
Independent reflections	11459 [R(int) = 0.0507]	
Completeness to theta = 53.594 °	99.6 %	
Absorption correction	Semi-empirical from equivalents	
Max. and min. transmission	0.7508 and 0.5610	
Refinement method	Full-matrix least-squares on F^2	
Data / restraints / parameters	11459 / 13 / 710	
Goodness-of-fit on F^2	1.038	
Final R indices [I>2 sigma(I)]	R1 = 0.0875, wR2 = 0.2556	
R indices (all data)	R1 = 0.0955, wR2 = 0.2669	
Absolute structure parameter	0.10(8)	
Extinction coefficient	0.0029(7)	
Largest diff. peak and hole	1.152 and -0.472 e.Å^{-3}	
